# Epigenetically silenced apoptosis-associated tyrosine kinase (AATK) facilitates a decreased expression of *Cyclin D1* and *WEE1*, phosphorylates TP53 and reduces cell proliferation in a kinase-dependent manner

**DOI:** 10.1038/s41417-022-00513-x

**Published:** 2022-07-28

**Authors:** Michelle L. Woods, Astrid Weiss, Anna M. Sokol, Johannes Graumann, Thomas Boettger, Antje M. Richter, Ralph T. Schermuly, Reinhard H. Dammann

**Affiliations:** 1 Institute for Genetics, Justus-Liebig-University Giessen, 35392 Giessen, Germany; 2 Department of Internal Medicine, Justus-Liebig-University Giessen, 35392 Giessen, Germany; 3 German Center for Lung Research (DZL), Giessen, Germany; 4 Scientific Service Group Biomolecular Mass Spectrometry, Max-Planck Institute for Heart and Lung Research, 61231 Bad Nauheim, Germany; 5 Max-Planck Institute for Heart and Lung Research, 61231 Bad Nauheim, Germany; 6 German Center for Lung Research (DZL), Universities of Giessen and Marburg Lung Center, 35392 Giessen, Germany; 7 Present Address: Institute for Translational Proteomics, Department of Medicine, Philipps-University, 35037 Marburg, Germany

**Keywords:** Gene regulation, Molecular biology

## Abstract

Silencing of the *Apoptosis associated Tyrosine Kinase* gene (*AATK*) has been described in cancer. In our study, we specifically investigated the epigenetic inactivation of *AATK* in pancreatic adenocarcinoma, lower grade glioma, lung, breast, head, and neck cancer. The resulting loss of *AATK* correlates with impaired patient survival. Inhibition of DNA methyltransferases (DNMTs) reactivated *AATK* in glioblastoma and pancreatic cancer. In contrast, epigenetic targeting via the CRISPR/dCas9 system with either EZH2 or DNMT3A inhibited the expression of *AATK*. Via large-scale kinomic profiling and kinase assays, we demonstrate that AATK acts a Ser/Thr kinase that phosphorylates TP53 at Ser366. Furthermore, whole transcriptome analyses and mass spectrometry associate AATK expression with the GO term ‘regulation of cell proliferation’. The kinase activity of AATK in comparison to the kinase-dead mutant mediates a decreased expression of the key cell cycle regulators *Cyclin D1* and *WEE1*. Moreover, growth suppression through AATK relies on its kinase activity. In conclusion, the Ser/Thr kinase AATK represses growth and phosphorylates TP53. Furthermore, expression of *AATK* was correlated with a better patient survival for different cancer entities. This data suggests that AATK acts as an epigenetically inactivated tumor suppressor gene.

## Introduction

With nearly 10 million deaths worldwide in 2020, cancer is a public health burden and a leading cause of death [1]. In the USA, costs for cancer care amounted for US$208.9 billion in 2020 [2]. Moreover, cancer patients are confronted with potential impoverishment due to insufficient health care coverage or indirectly through non-medical costs as a result of productivity losses because of morbidity [3]. These economic and socio-economic aspects highlight the need for continued research into underlying cause as well as the prognostic determinants of cancer.

Epigenetic silencing of tumor suppressor genes has been studied extensively and is thought to be a driving event in early oncogenesis [4, 5]. One hallmark of epigenetic silencing is the hypermethylation of CpG island promoters which leads to transcriptional repression of the corresponding gene [6]. DNMTs are responsible for the aberrant modification and maintenance of 5-methyl-cytosine at CpG sites. In turn, these serve as binding sites for methyl-CpG binding domain proteins, an event that leads to chromatin condensation [7]. Previously, we have identified multiple epigenetically silenced tumor suppressor genes in human carcinogenesis [8, 9].

AATK was first discovered as induced during apoptosis in mouse myeloid precursor cells [10]. It has since been implicated in neuronal differentiation, axon outgrowth, and dendrite formation [11, 12]. In neuronal cells, interaction with the cytoskeleton and regulation of endosome trafficking have been described [13, 14]. Initial evidence for potential epigenetic silencing of *AATK* has come from lung and breast cancer as well as pancreatic ductal adenocarcinoma [15, 16]. AATK suppresses growth of melanoma cells, lung and cervix cancer cells [16, 17]. Silencing of *AATK* leads to proliferation of pancreatic ductal cells and resistance to radiotherapy of lung cancer cells [15, 18]. However, the functional mechanism underlying the tumor suppressive function of AATK in respect to its kinase activity has not been analyzed in detail.

AATK belongs to the LMTK family of kinases. To date, only LMTK2 has been identified as Ser/Thr kinase [19]. Based on homology, AATK has also been presumed to convey a Ser/Thr kinase activity [20]. High throughput kinomic profiling can be used to determine the global kinase activity in whole cell lysates [21]. The PamStation, fluorescent kinase activity assay, for example, measures the capabilities of kinases to phosphorylate specific peptides and differentiates between Tyr kinase and Ser/Thr kinase [22]. This system was utilized, to assess the kinase activity of AATK in a broad network.

In the present work, we analyzed the function and regulation of *AATK* in several cancer entities. We demonstrate that AATK acts a Ser/Thr kinase and that it phosphorylates TP53. We further find the cell cycle drivers cyclin D1 (*CCND1)* and *WEE1* to be repressed by AATK induction and identify the loss of *AATK* via epigenetic silencing as correlated to impaired overall patient survival in various cancers.

## Materials and methods

### DNA methylation analysis

**Fig. 1:**
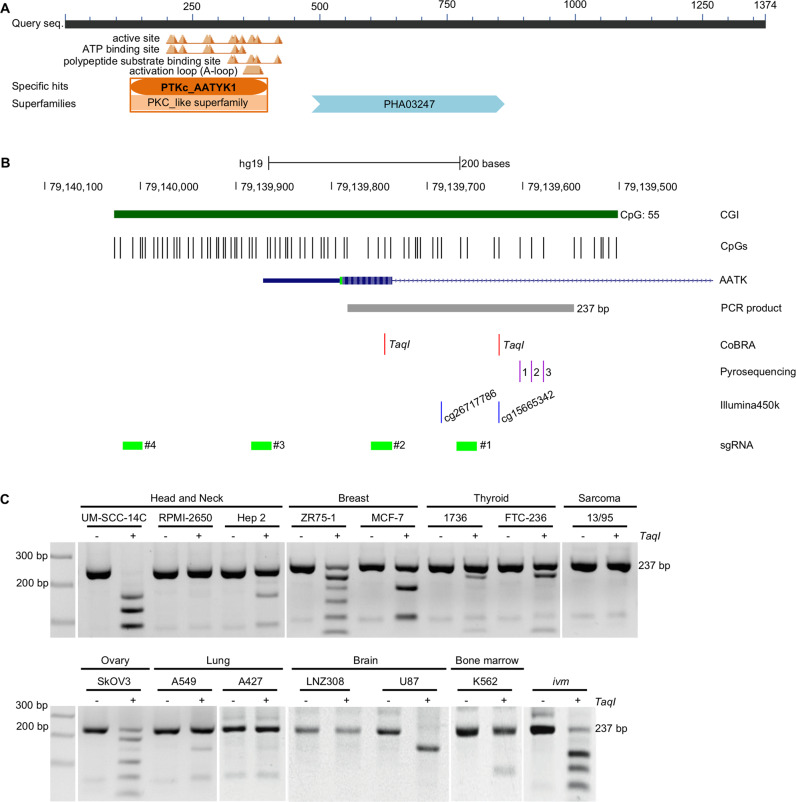
Structure of AATK and hypermethylation of its CpG island promoter in cancer. **A** AATK is characterized by a tyrosine kinase domain (NCBI tool for conserved domain search) [76]. **B** *AATK* is located on chromosome 17q25.3. A 537 bp CpG island (CGI) (green) overlaps the transcription start site [40]. The frequency of the individual CpG sites is depicted (black lines). For the methylation analyses via combined bisulfite restriction analysis (CoBRA) and pyrosequencing, a 237 bp PCR product is generated (gray bar). Two *TaqI* restriction sites for CoBRA (red), three CpGs for pyrosequencing (purple), and two CpGs (cg26717786 and cg15665342) for Illimuna450k array (blue) are marked. For epigenetic editing, four gRNAs (#1–#4) covering the CGI were generated (light green). **C** A panel of 14 cancer cell lines with three head and neck, two breast, two thyroid, one sarcoma, one ovary, two brain cancer cell lines, and an in vitro methylated (ivm) sample were analyzed via CoBRA. PCR products were *mock* (−) and *TaqI* (+) digested.

The promoter region of *AATK* was analyzed by bisulfite-based DNA methylation assays. The precise promoter region was chosen for CpG content and presence of *TaqI* restriction sites for CoBRA analysis [23]. Genomic DNA was bisulfite treated as previously described [16]. Primers for methylation analysis of the AATK promoter are listed in Table S1. PCR product is 237 bp with *TaqI* sites at position 39 and 158 (Fig. 1). PCR products were either digested with *TaqI* (Thermo Fisher Scientific) 2% TBE gel together with mock control or pyrosequencing was performed according with PyroMark Q24 System (Qiagen). In vitro methylation of genomic DNA was generated using CpG methyltransferase *M.SssI* (NEB).

### RNA expression analysis

RNA was isolated from human cell culture using Trizol-RNA lysis procedure (Thermo Fisher Scientific). RNA was *DNaseI* (Thermo Fisher Scientific) treated and then reversely transcribed by MMLV (Promega). Quantitative RT–PCR was performed in triplicate with SYBR select (Thermo Fisher Scientific) using Rotor-Gene 3000 (Qiagen) and normalized to *GAPDH/ACTB* level. Primers for RT–PCR are listed in Table S1.

We performed RNA microarrays (Clariom S human) according to manufacturer’s protocol (P/N 703174 Rev. 2) with 200 ng of total RNA. Reagents/equipment were GeneChip wt PLUS Reagent Kit, P/N: 902280; GeneChip Hybridization, Wash, and Stain Kit P/N 900720, GeneChip Scanner, GeneChip Fluidics Station 450, GeneChip Hybridization Oven 640, Bioanalyzer 2100 (Agilent), and RNA600 NanoKit (Agilent).

### Cell lines and transfections

The cell lines TREx293, HEK293T, U343, and MCF-7 were cultivated in DMEM or RPMI with 10% FCS and 1% Penicillin-Streptomycin at 37 °C under 5% CO_2_. HCT116 p53^+/+^ and HCT116 p53^−/−^ were obtained from Thorsten Stiewe (University Marburg, Germany) and cultivated in DMEM [24]. The cells were transfected at a confluency of 60–80% in serum-free media (Optimem, GIBCO) or at 40,000 cells (96-well plates) in serum-free media (Optimem, GIBCO) with 0.16, 4, or 7 µg DNA (96-well, 6-well, 10 cm plates respectively). HEK293T cells were transfected using PEI (Sigma). TREx293 and MCF-7 cells were transfected using Turbofect (Thermo Fisher Scientific). U343 and HCT116 cells were transfected using X-tremGENE HP (Roche). Carcinomas and matching normal tissue samples were obtained from patients of the University of Halle-Wittenberg and were previously described [25]. The local committee of medical ethics approved the use and all patients gave their consent.

### Generation of AATK wt and AATK KD (kinase dead) stable cell lines

AATK coding sequence was kindly provided by Ma and Rubin [16] and cloned into, pEYFP-C2 (Clontech) and pCMVTag1 (Flag; Agilent). The ATP binding pocket of AATK was mutated (Ala>Lys) by site-directed mutagenesis (Agilent). DNA of AATK wt or AATK KD were cloned into pcDNATo/Myc/His vector (Thermo Fisher Scientific). TREx293 cells, that stably express the Tet repressor (Thermo Fisher Scientific), were transfected with the expression vector pcDNA4TO-AATK wt or pcDNA4TO-AATK KD and selected with Zeocin (Thermo Fisher Scientific). The cells were cultivated in DMEM with 10% tetracycline-free serum (Biochrom) and 1% penicillin and streptomycin (GIBCO). The selection of the clones was performed using blasticidin (5 µg/ml, Roth) and Zeocin (500 µg/ml, Thermo Fisher Scientific). The induction of AATK wt or AATK KD was performed using doxycycline (2 µg/ml, Thermo Fisher Scientific).

### *AATK* knockdown

Knockdown of *AATK* was performed with siRNA (Dharmacon). MCF-7, HEK293T, and Sk-Mel13 cells were transiently transfected with either 50 pmol of a non-targeting siRNA control pool or with 50 pmol of the siRNA for *AATK* (SMARTpool) using the Lipofectamine RNAiMAX (Thermo Fisher Scientific). The cells were harvested after 2 d for RNA isolation.

For UV irradiation, knockdown of *AATK* MCF-7 cells was performed as described. Cells were starved for 72 h after transfection, exposed to 40 J/m^2^, and recovered in full media for 30 min at 37 °C. Following recovery, the cells were lysed as described above and lysates were subjected to Western blot analysis.

### Cell viability assay (MTS-assay)

Cells were seeded at 20,000 cells per well and grown for 24 h. MCF-7, HEK293T, HCT116 p53^+/+^, and HCT116 p53^−/−^ were transfected as described above. Clone pools were induced as described above. 24 h post transfection or induction, the CellTiter 96 AQ_ueous_ Non-Radioactive cell proliferation assay was performed according to manufacturer’s protocol (Promega).

### Binding partner identification using GFP Trap and mass spectrometry

AATK-EYFP vs. EYFP-empty were overexpressed in HEK293T cells (24 h), and pulldown was performed by GFP Trap (ChromoTek). Peptide/protein group identification & quantitation was performed using the MaxQuant suite of algorithms [26, 27] (v. 1.6. 5.0) against the human uniprot database (canonical and isoforms; downloaded on 01/23/2019; 191,406 entries). Relevant MaxQuant settings and instrument parameters extracted using MARMoSET are summarized and included in the supplement [28].

### Kinome analysis

TREx inducible clone pools (AATK wt and AATK KD) were cultured in 10-cm dishes until they reached 80% confluency and starved for 24 h. The next day, exogenous AATK expression was induced with doxycycline (2 µg/ml, Thermo Fisher Scientific) for 24 h. Description of cell lysis, the peptide-based activity assay and its analysis have been explained in detail previously [22, 29–31]. Kinase activity measurement was performed using the PamStation12 platform (PamGene International) with Evolve 12 software.

### Epigenetic editing via CRISPR-dCas9

CRISPR-Cas9 vector px549 was obtained from Lienhard Schmitz (Giessen, Germany) and adapted for epigenetic editing by inactivation of Cas9 by site-directed mutation (dCas9). AATK guide oligos were cloned into *BbsI* sites of px549-Cas9 or px549-dCas9. Epigenetic modifier plasmids were ordered from Addgene: pcDNA-dCas9-p300 Core (61357), pdCas9-DNMT3A (100090), Ezh2[SET]-dCas9 (100087). Epigenetic editing of endogenous *AATK* in HEK293T. Guided oligos for AATK are #1 CACGGCCCCCGGCCCG, #2 TCAGCTCGCACTTCGACCCC, #3 CGGCCGCTGGGTGATGCGGC, #4 ACTGCACCAGCGGAGCCCCG and are positioned relative to TSS at −372 #1, −282 #2, −155 #3, −21 #4. Primers for genomic analysis are listed in Table S1. Sanger sequences are depicted as an original dataset in the supplement.

### Western blot and antibodies

The cell lysates were loaded on 10% SDS gels and blotted onto a PVDF membrane (Amersham). Santa Cruz antibodies: anti-GAPDH (FL-335, 1:1000), anti-p53 (Do-I, 1:1000), and anti-CytochromeC (13156, 1:500). Abcam antibody: anti-AATK (ab56625). BidScientific antibody: anti-p53-pSer366 (E-AB-21221, 1:500). Anti-GFP rabbit polyclonal serum (1:1000) was kindly provided by Jörg Leers (Giessen, Germany).

### Immunofluorescent staining

Cells were grown on coverslips for 24 h. HEK293T were transfected with AATK wt-EYFP, AATK KD-EYFP or empty control, and AATK wt or AATK KD expression was induced with doxycycline in the TREx clone pools. After 24 h, cells were washed, fixed in 3.7% formaldehyde–PBS solution, permeabilized with 0.5% Triton X-100–PBS solution, and blocked with 1% BSA-PBS solution. For HEK293T, incubation with TP53Ser366 (#A8053 Assay Biotech) at 1:50 and subsequent incubation with anti-rabbit Alexa568 was performed. For the TREx clone pools, simultaneous incubation with primary antibodies against TP53Ser366 (#A8053 Assay Biotech) at 1:50 and Anti-FLAG M2 (#F1804 Sigma-Aldrich) at 1:100 in 1% BSA-PBS solution, subsequent simultaneous incubation with secondary fluorescent antibodies (anti-mouse Alexa568 and anti-rabbit Alexa488) were performed. Thereafter, cells were stained with DAPI (Invitrogen) and mounted with Mowiol. Images were acquired using an Axio Observer.Z1 inverted microscope (Carl Zeiss) equipped with Zeiss Zen 3.1 (blue edition) software and the Axiocam 506 mono system (Carl Zeiss). Processing of the images was performed with Fiji/ImageJ (version 1.51n). Bean plots were generated via http://shiny.chemgrid.org/boxplotr/.

### Immunoprecipitation and subsequent in vitro kinase assay

For the immunoprecipitation, HEK293T cells were transiently transfected either with 7 µg of pCMV-Flag-AATK wt or Flag-AATK KD and empty vector control or GFP-p53. The lysis was performed with either FLAG- or GFP Trap-lysis buffer supplemented with PMSF, pepstatin, aprotanin, leupeptin. 1/10 of the lysates were referred to as input. The cell lysates were incubated overnight with anti-flag M2 agarose beads (Sigma) or with anti-GFP agarose beads (Chromotek) for 1 h. Beads were washed twice with cold TBS and washed twice with GFP-Trap dilution/wash buffer before denaturation. The final washing step was performed with 1xkinase buffer (#9802 Cell Signaling). Beads with the bound substrate were resuspended in 50 µl 1xkinase buffer supplemented with 200 µM ATP. The 50 µl resuspended substrate was added to the beads with the bound kinase or bound tag without kinase and incubated for 30 min at 30 °C. The assay was stopped by adding loading buffer and boiling at 95 °C for 5 min. Cell lysates (input) and IPs were separated by SDS-PAGE and subjected to Western blot analysis with TP53 Ser366 phosphorylation antibody.

### Further analysis of publicly accessed data

Gene expression, promoter methylation correlation, and Kaplan–Meier calculations were performed using R2 Genomics Analysis and Visualization Platform [32], Wanderer [33], KM Plotter [34, 35], MethSurv [36], and SMART App [37]. Public gene category analyses were performed using R2 [32]. One-way ANOVA was performed.

## Results

### *AATK* is epigenetically inactivated in various cancers and its loss is associated with impaired prognosis

**Fig. 2:**
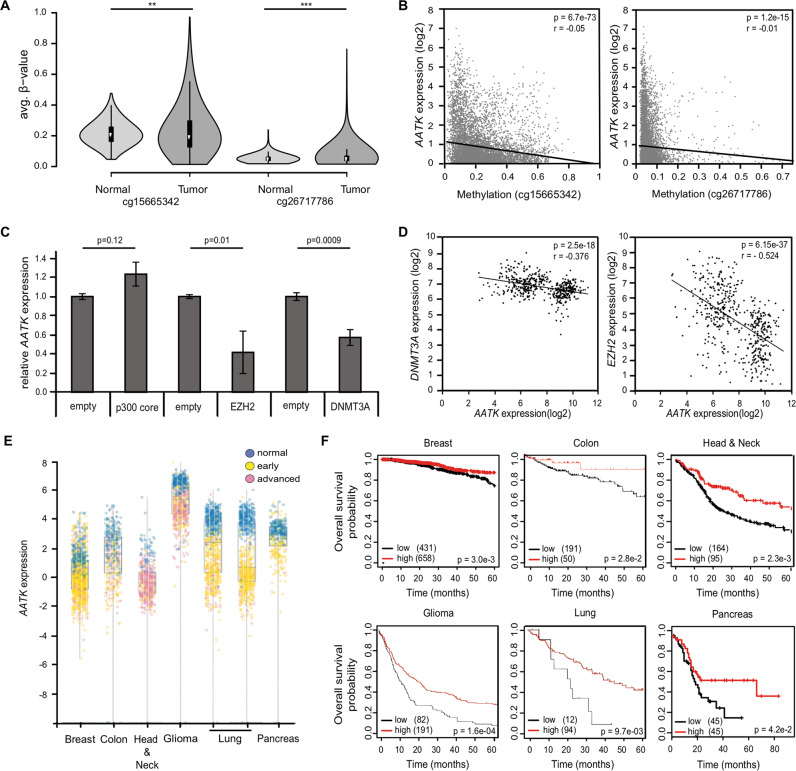
*AATK* expression is epigenetically downregulated in cancer. **A** Via Infinium Human Methylation 450 BeadChip (ilmnhm450K array) and visualized by the SMART app [37], the methylation levels of *AATK* at two CpG sites within the CpG island (cg1566342 and cg26717786) in normal tissues (*n* = 711) compared to tumor samples (*n* = 8893) from the TCGA project are plotted as beta-value (1 = 100% methylation) and the *p*-values are calculated by one-way ANOVA. **B** Correlation analysis of methylation (beta-value) and expression of *AATK* (log2). Methylation of two CpG sites (cg26717786 and cg1566342) is plotted for *AATK* expression (ENST00000417379.5) in various tumor samples (*n* = 8411) from the TCGA project. The data obtained from the SMART app [37] for all tumor entities are plotted together. The R-values of linear regression and the *p*-values of correlation are given by regression statics via Excel. **C** Epigenetic editing of the *AATK* promoter via the CRISPR/dCas9 system. The relative *AATK* expression is normalized to GAPDH and is displayed after co-transfection of four gRNAs targeting the *AATK* promoter with effector proteins, p300 core domain, EZH2, DNMT3A, or empty vector in HEK293T cells for 48 h. **D** Correlation analysis of *AATK* (205986_at) and *DNMT3A* (222640_at) or *EZH2* (203358_s_at) expression in normal tissues (GSE7307). **E** *AATK* expression throughout tumor progression in breast cancer, head and neck squamous cell carcinoma, glioma, lung cancer (left: lung adenocarcinoma, right: lung squamous cell carcinoma) [44]. **F** Overall survival probability in correlation with expression of *AATK* was analyzed via km plotter in breast and stage four HNSCC [34], in colon adenocarcinoma with unknown KRAS mutation status (TCGA ID: COAD with date: 2000-01-01), glioma (GSE16011) and lung cancer (GSE3141) via R2 Genomics Analysis and Visualization Platform [32] and in pancreatic adenocarcinoma via GEPIA [45].

AATK has mainly been described in the context of neuronal development [11, 38, 39]. The AATK protein has been associated with apoptosis and reduced proliferative activity [16, 17] and is characterized by a kinase domain (Fig. 1A). The *AATK* gene is located on chromosome 17q25.3 and a 537 bp large promoter-associated CpG island (CGI) overlaps the transcription start site (Fig. 1B) [40]. The promoter methylation of the *AATK* promoter was analyzed via CoBRA in various cancer cell lines from head and neck, breast, thyroid, sarcoma, ovary, lung, brain, and bone marrow (Fig. 1C). The CGI of *AATK* was methylated in 10 out of 14, i.e., 71% of the analyzed cancer cell lines. Thus, we further analyzed the methylation of two 450 K array probes (cg15665342 and cg26717786) within the CGI of *AATK* in 711 normal tissues and 8893 tumor samples from the TCGA project (Fig. 2A) [37]. The increase in scattering of the promoter methylation of both CpGs is evident when comparing normal with tumor tissues (Fig. 2A). In addition, the expression of *AATK* and the corresponding methylation at these CpGs significantly correlates in these tumor samples (Fig. 2B). A lower expression of *AATK* is found in samples with high methylation levels.

In respect to the observed results, we analyzed the methylation of the CGI of *AATK* in eight glioblastoma cell lines (LN229, U343, U118, U87MG, T98G, A172, U251, LNZ308), one glioma (A764) and one astrocytoma (SNB19) cell line (Fig. S1). 78% of the glioblastoma cell lines were methylated and reduced expression of *AATK* was observed in 7 out of 8 cell lines. The glioma cell line was also strongly methylated and *AATK* expression was as entirely undetectable (Fig. S1A, B). Upon pharmacological inhibition of DNMTs with 5-Aza-2′-deoxycytidine in the glioblastoma cell line U87MG, a slight decrease in methylation of *AATK* (Fig. S1D) as well as re-expression of *AATK* were observed (Fig. S1E). Via Geo2R [41], we analyzed the promoter CGI (cg15665342, cg26717786) and eight CpG sites in the shore regions of *AATK* in normal brain (Corpus callosum) and glioma (GII) samples [42]. A significant increase in methylation from normal brain to glioma samples was seen for both annotated CpGs within the CGI (Fig. S2A). Therefore, we analyzed the overall survival probability in relation to the methylation of cg15665342 and found that a decreased methylation at this CpG site coincided with a higher survival probability of glioma patients [36] (Fig. S2B).

In addition, we determined that the CGI of *AATK* was methylated in 100% of seven pancreatic cancer cell lines (Hup-T3, Capan-1, PATU-S, Capan-2, PATU-T, PaCa2, PATU-02) and pharmacologically inhibiting DNMTs with 5-Aza-2′-deoxycytidine led to demethylation and re-expression of *AATK* (Fig. S3A–D). Further, via pyrosequencing, we found that the mean methylation of the promoter CGI of *AATK* was significantly higher in pancreatic adenocarcinoma (PAAD > 22%) and acute pancreatitis (>20%) compared to normal tissues (10%) (Fig. S3E). Via Wanderer [33], we observed that cg15665342 is methylated significantly higher in PAAD in comparison to normal pancreatic tissue (Fig. S4A). Furthermore, a significantly higher overall survival probability coincides with a reduced methylation of cg15665342 in patients with pancreatic PAAD (Fig. S4B) [36].

In head and neck squamous cell carcinoma (HNSCC), we analyzed the *AATK* methylation in matching normal and tumor samples, finding 36% of the tumor samples significantly higher methylated than the matching normal sample (Fig. S5). Utilizing Wanderer [33], we observed that cg15665342 is methylated significantly higher in HNSCC in comparison to normal tissues and higher methylation at this CpG site is linked to poorer overall survival of HNSCC patients (Fig. S6) [36].

To further pinpoint the regulatory mechanisms underlying the expression of *AATK*, we utilized deactivated CAS9 (dCAS9) fused to epigenetic modulators. We targeted the *AATK* promoter directly with four sgRNAs (Fig. 1B). The generated sgRNAs effectively target *AATK* genomically (Fig. S7A, B, Dataset 1). Transcription factor ChipSeq Clusters (161 factors) from ENCODE display the binding potential of EZH2 and p300 to the *AATK* promoter region (Fig. S7C). When targeting the endogenous *AATK* promoter in HEK293T via the p300 HAT core, there was not a significant change in expression (Fig. 2C). However, a significant reduction in *AATK* expression was achieved by tethering DNMT3A or EZH2 to its CGI (Fig. 2C). This finding is corroborated by the inverse correlation between *AATK* expression and *DNMT3A* or *EZH2* levels in various normal tissues (Fig. 2D).

Grippingly, downregulation of *AATK* expression can be found throughout tumor progression in breast cancer (BRCA), colon adenocarcinoma (COAD), HNSCC, lung cancer, and PAAD (Fig. 2E) [43, 44]. Furthermore, in BRCA and stage four HNSCC [35], in COAD, glioma (LGG), lung cancer [32], and PAAD [45], higher expression of *AATK* coincides with an increased overall survival probability (Fig. 2F).

### Expression of *AATK* is associated with negative regulation of cell proliferation

**Fig. 3:**
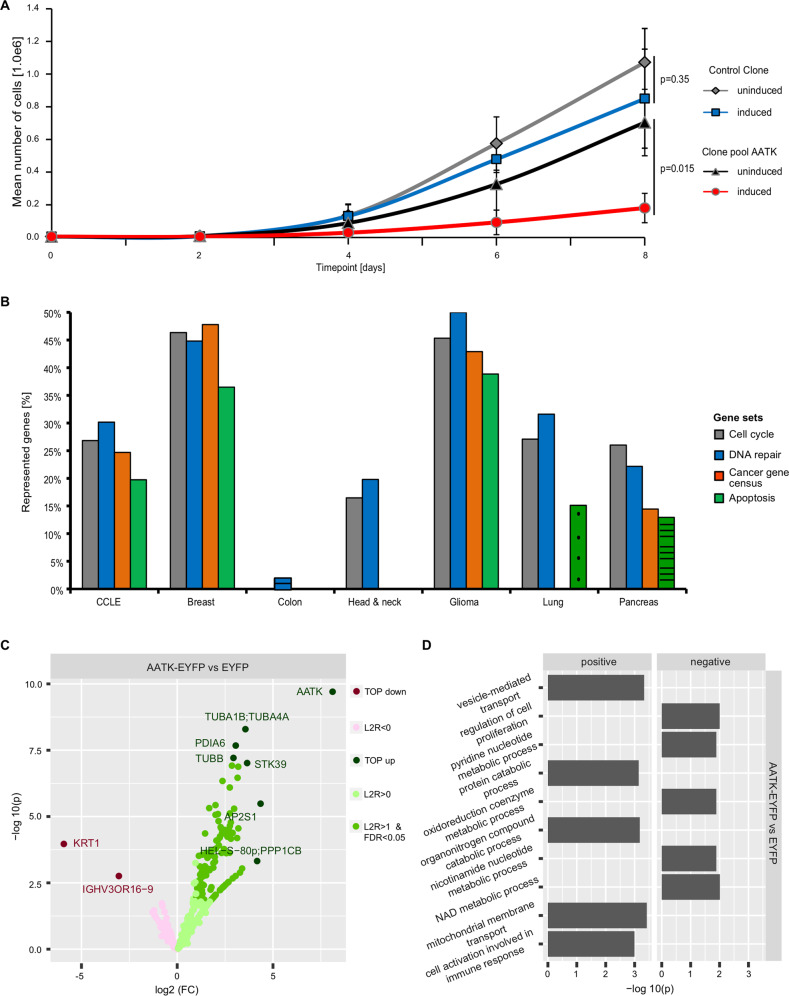
Exogenous AATK expression inhibits cell growth and is associated with negative regulation of cell proliferation. **A** Using the TREx293 cell system, growth curves of a control clone pool and an *AATK* expressing clone pool with doxycycline (induced) and without (uninduced) were generated. Cells were grown for eight days and counted every two days (displayed as mean of three independent experiments with SD, unpaired two-sided *t*-test). **B** Using the R2 Genomics Analysis and Visualization Platform [31], gene sets (public gene categories) negatively correlated to *AATK* expression in various cancer cell lines (GSE363133), breast cancer (tcgaBRCA1097), colon cancer (tcgaCOAD286), head and neck cancer (tcgaHNSC520), glioma (tcgaLGG516), lung cancer (GSE3141), as well as pancreatic cancer (tcgaPAAD178) were determined and are shown with color code. Solid: *p* < 0.001, striped: *p* < 0.01; dotted: *p* < 0.05. **C** Binding partners of AATK were detected in HEK293T cells after transfection of EYFP-tagged AATK and EYFP empty and subsequent pulldown with GFP-Trap and mass spectrometry. Enrichment of interaction partners of control samples was determined from three replicate experiments using the limma-based analysis package autonomics [77]. **D** Overrepresentation analysis of enriched interaction partners of AATK-EYFP vs. EYFP was performed against categories from the Kyoto Encyclopedia of Genes and Genomes [45] and Gene Ontology [46]. The contrast AATK-EYFP to EYFP displays the (sub)ontology GObp in the protein groups data sets. Data is facetted by regulation direction in the context of contrast. Significances were determined with a Fisher Exact Test.

Strikingly, *AATK* expression is epigenetically regulated in cancer cell lines and reduced expression of *AATK* is a common molecular phenotype in carcinogenesis associated to poor disease outcome. Consequently, the effect of exogenous expression of AATK on cell growth as a potential therapeutic avenue was explored. To this end, we generated inducible clone pools expressing AATK in a doxycycline-dependent manner (Fig. 3). Growth curves of the inducible AATK expressing cells and control cells were generated after eight days of doxycycline induction (Fig. 3A). In three independent experiments, we could see a significantly slower growth of AATK expressing cells in comparison to the uninduced clone pool as well as to the uninduced and induced control cells. To rationalize this finding, we used the platform R2 [31] to perform gene ontology analyses focusing on gene categories in cancer cell lines, BRCA, COAD, HNSCC, glioma, lung cancer, and PAAD data sets (Fig. 3B). The gene category “DNA repair” was found as significantly associated with reduced expression of *AATK* in all data sets. Categories “Cell cycle” (6 out of 7 data sets), “Apoptosis” (5/7), and “Cancer gene census” (4/7) were also correlated with reduced *AATK* expression (Fig. 3B).

Using AATK-EYFP pulldown assays analyzed by quantitative mass spectrometry to identify its binding partners, we followed up on the cell cycle theme. Besides previously described interaction partners such as STK39 [46], we also identified new interaction partners such as tubulins (Fig. 3C). Overrepresentation analysis (ORA) of enriched interaction partners of AATK-EYFP vs. EYFP was performed against categories from KEGG [47] and GO [48]. In this context, significantly up- and downregulated (sub)ontology GO Biological processes (GOBP) are displayed (Fig. 3D). While the upregulation of GOBP “vesicle-mediated transport” validates earlier reports [47], the enrichment of the term “regulation of cell proliferation” further underlines the findings derived from transcriptomic analysis (Fig. 3B).

### Downregulation of *WEE1* and *CCND1* expression is dependent on the kinase activity of AATK

**Fig. 4:**
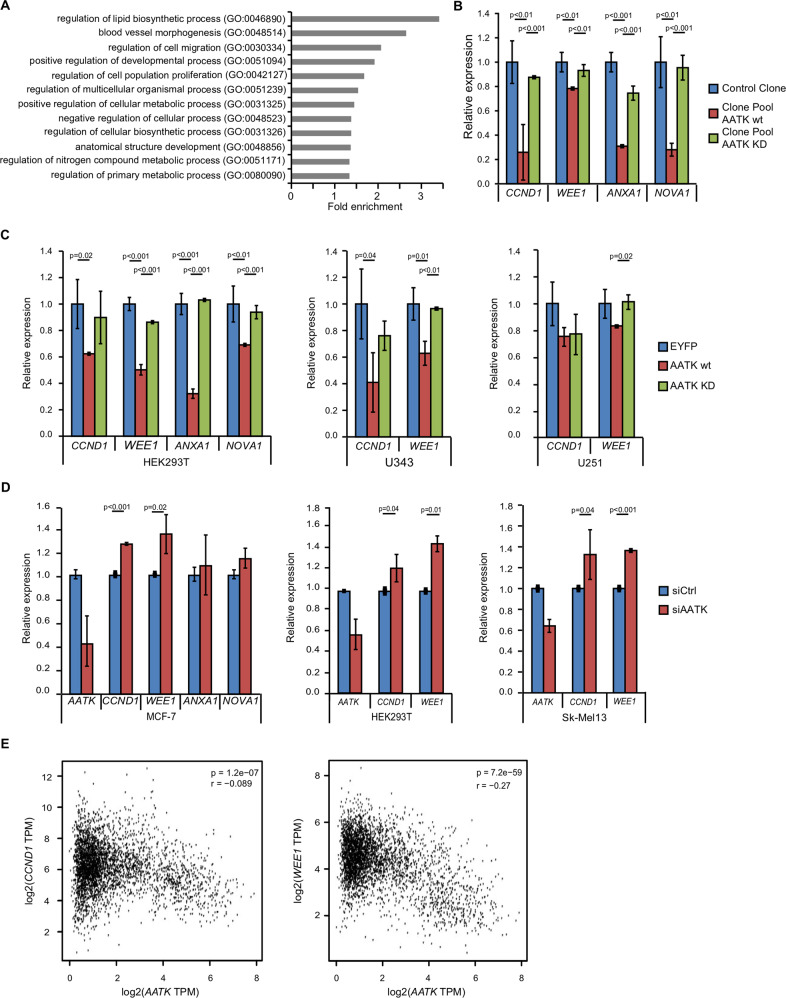
Expression of *CCND1* and *WEE1* is negatively regulated by AATK. **A** Whole transcriptome analysis via affymetrix array of a control clone pool, an AATK wt (wild type) expressing clone pool and an AATK KD (kinase dead) expressing clone pool was performed. After eliminating lowly expressed (linear values ≤100) genes, logFC (≤−0.6, 0.6≥) with respect to AATK wt expressing sample and control clone were determined. Thereafter, the top 500 deregulated genes were subjected to GO term analysis via PANTHER [78]. All displayed GO terms are significantly enriched (Fisher’s exact test with FDR correction). **B** A panel of cell cycle regulatory genes differentially expressed after induction of AATK wt expression is displayed as relative expression to induced control clone and normalized to *GAPDH* (SD, unpaired two-sided *t*-test). **C** Various cell lines (HEK, U343, U251) were transfected with EYFP-tagged AATK wt, AATK KD, or EYFP empty. Relative expression to EYFP and normalized to β-Actin with SD (unpaired two-sided *t*-test). **D** A knockdown of *AATK* was performed for 48 h in various cell lines (MCF-7, HEK, and Sk-Mel13). Relative expression of various cell cycle regulatory genes normalized to *β-Actin* with SD (unpaired two-sided *t*-test). **E** Pearson correlation of *AATK* and *CCND1* or *WEE1* expression in breast cancer, colon adenocarcinoma, glioma, lung cancer (adeno- and squamous cell carcinoma), and pancreatic adenocarcinoma was analyzed via GEPIA [45]. TPM = transcripts per million.

For further insight into the mechanism of the suppression of cell proliferation by AATK re-expression, we analyzed its effect on gene subsets with the TREx inducible system and explored whether AATK kinase activity impacts those effects. To that end, we generated an inducible clone pool for an AATK kinase-dead (KD) variant in the TREx system, in which the ATP binding pocket was mutated to obliterate ATP binding. The genome-wide differential transcriptomes of three inducible clone pools i.e., control clone, AATK wt (wild type), and AATK KD (kinase dead) were analyzed (Fig. S8). The 500 most up- and downregulated genes (DEGs) in the control clone and the AATK wt expressing cells were subjected to GO term analysis via PANTHER [49] and highly significantly enriched GOBP are depicted (Fig. 4A). Interestingly, “regulation of cell population proliferation” was detected, among others. Furthermore, a clustering of the transcriptomes of AATK KD and the control cells was revealed (Fig. S8). Further exploring these findings, we identified genes for which expression was altered by expression of AATK compared to AATK KD. Hereby, we detected significant decreases in expression of *cyclin D1 (CCND1)* and *WEE1*, *ANXA1* and *NOVA1* after induction of AATK wt but neither in the control clone nor in AATK KD expressing cells (Fig. 4B). The expression of *CCND1* is decreased in HEK293T and the glioblastoma cell line U343 after overexpression of EYFP-tagged AATK wt in comparison to EYFP-tagged AATK KD or EYFP empty (Fig. 4C). *WEE1* was decreased in HEK293T cells and both glioblastoma cell lines (Fig. 4C). To further evaluate whether the decreased expression of *CCND1* and *WEE1* was directly attributed to the expression of *AATK*, we performed RNAi for *AATK* loss of function studies (Fig. 4D). The knockdown of *AATK* expression caused a significant increase in *CCND1* and *WEE1* expression in the breast cancer cell line MCF-7, HEK293T and the melanoma cell line Sk-Mel13 (Fig. 4D). It did not affect the expression levels of *NOVA1* or *ANXA1*. Next, we observed a significant negative correlation between *AATK* and *WEE1* or *CCND1* expression in BRCA, COAD, LGG, lung cancer (adeno- and squamous cell carcinoma), and PAAD via GEPIA [45] (Fig. 4E). In addition, survival analyses indicate a higher survival probability for low *CCND1* and high *AATK* expression in HNSCC, pancreatic ductal adenocarcinoma (PDA) and lung cancer (lung squamous cell carcinoma and adenocarcinoma) (Fig. S9). Similarly, survival analyses for low *WEE1* and high *AATK* expression indicate a higher survival probability in BRCA and lung cancer, HNSCC, and PDA (Fig. S10).

### The Ser/Thr kinase AATK phosphorylates TP53 at Ser366

**Fig. 5:**
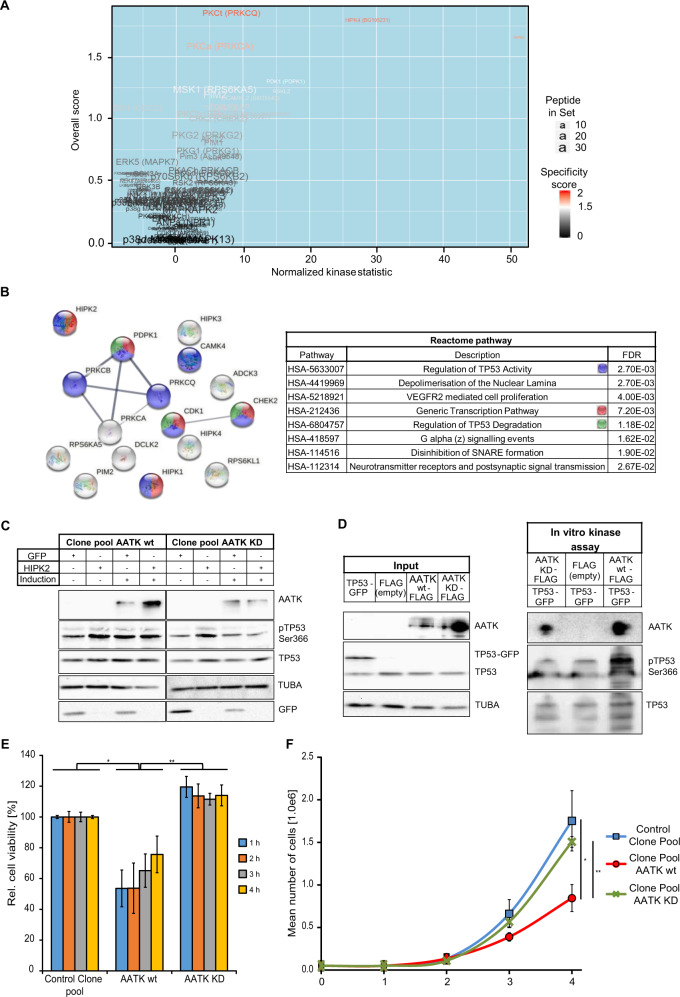
The Ser/Thr kinase AATK. **A** Peptide-based kinase activity assays were performed with an AATK wt and AATK KD expressing clone pool with doxycycline for 24 h. Computational upstream kinase analysis uncovered the basal kinomic activity profile Ser/Thr kinases. Due to the differential pattern of peptide phosphorylation, an increased activity of certain upstream kinases was predicted and these are represented by a volcano plot. **B** The significantly over-activated upstream kinases that compare to the activity profile of AATK wt were subjected to STRING analysis (left) [50] and the significantly (FDR = false discovery rate) represented Reactome pathways are displayed (right). Bubbles represent kinases, lines highlight interactions. **C** Lysates of AATK wt or KD expressing clone pools with overexpression of HIPK2-GFP or GFP empty were subjected to immunoblotting. **D** Input (left) and in vitro kinase assay (right) with immunoprecipitated TP53-GFP added to immunoprecipitated FLAG (empty), AATK wt-FLAG or AATK KD-FLAG to determine the phosphorylation status of TP53 at Ser366. **E** Cell viability was determined using MTS assay. 24 h post induction of AATK wt or AATK KD expression. MTS absorbance was measured at 490 nm with reference at 650 nm hourly for 4 h. **F** Via the TREx293 cell system, growth curves of a control clone pool, an AATK wt and an AATK KD expressing clone pool were generated. Cells were grown 4 days and counted every day (displayed as mean of three independent experiments with SD, unpaired two-sided *t*-test).

To further characterize, the function of AATK, we performed a peptide-based kinase activity profile [22]. We utilized the induced AATK wt and AATK KD expressing clone pools for phospho-tyrosine kinase (PTK) and serine/threonine kinase (STK) assays. The individual basal activity profiles of all three replicates for AATK wt and AATK KD in the PTK assay was very heterogeneous (Fig. S11A). In contrast, the activity profiles from the STK assay comparing the AATK wt and AATK KD expressing clone pools depicted a clearly diverging activity profile (Fig. S11B). Distinct peptides are phosphorylated by AATK wt but unphosphorylated in the AATK KD samples and vice versa. Upstream kinase predictions based on the phosphorylated peptides, identified a subset of Ser/Thr kinases that are highly active in samples expressing AATK wt as compared to the kinase-dead mutant (Fig. 5A). Among others, all members of the Homeodomain-interacting protein kinases (HIPK1-4), MSK1 and CHEK2 showed putatively higher and CDK1 putatively lower activity. On this basis, the significantly determined kinases were subjected to STRING analysis [50] and enriched Reactome pathways were viewed (Fig. 5B and Fig. S12). Interestingly, “Regulation of TP53 activity”, “Generic transcription pathway” and “Regulation of TP53 degradation” are significantly enriched (Fig. 5B). Since Ou et al. [51] demonstrated that phosphorylation of TP53 at Ser366 was correlated with its activity and stability, the phosphorylation of TP53 at Ser366 after exogenous expression of AATK wt or AATK KD with and without exogenous HIPK2 expression in the respective clone pools was analyzed (Fig. 5C). Induction of AATK wt led to phosphorylation of TP53 at Ser366 to an extend equivalent to exogenous overexpression of HIPK2. Furthermore, induction of AATK KD reduced the basal phosphorylation of TP53 at Ser366. In addition, the induction of TP53 phosphorylation at Ser366 through exogenous expression of HIPK2 was inhibited through the induction of AATK KD. In the next step, MCF-7 cells expressing AATK wt or KD exogenously were subjected to UV irradiation (40 J/m^2^) to induce DNA damage (Fig. S13). In comparison to the control cells, an increase in phosphorylation was seen upon AATK wt expression and a decrease in phosphorylation after UV irradiation was detected in the AATK KD expressing cells (Fig. S13). Further, we performed RNAi for *AATK* in MCF-7 cells which were subjected to UV irradiation (40 J/m^2^). The loss of *AATK* reduced the UV-induced phosphorylation of TP53 at Ser366 (Fig. S14). Building on these findings, in vitro phosphorylation of TP53-GFP through AATK wt was evaluated. In contrast to the controls and AATK KD a clearly increased phosphorylation level of TP53 at Ser366 was identified (Fig. 5D). Furthermore, exogenous expression of AATK wt in HEK293T resulted in a significant increase of TP53 phosphorylation at Ser366 in comparison to EYFP empty and AATK KD expression (Fig. S15A, B). The increase in fluorescence signal intensity was also observed in the inducible clone pool expressing AATK wt in a doxycycline-dependent manner in comparison to the control clone pool and the AATK KD expressing clone pool (Fig. S16A, B).

Based on these findings, the expression of *CCND1* and *WEE1* was evaluated in *p53*-deficient and *p53wt* HCT116 cells in combination with exogenous expression of AATK wt and KD (Fig. S17). In the HCT116-p53wt cells, a significant decrease in expression of *CCND1* was observed in samples expressing AATK wt. In contrast, in the HCT116-Δp53 cells an upregulation of *CCND1* expression was detected after AATK wt overexpression. The expression of *WEE1* was not significantly altered. In addition, we assessed cell viability via MTS assay in these cells after exogenous expression of AATK wt and KD (Fig. S18). In general, the HCT116-Δp53 cells were more viable than the HCT116-p53wt cells. This effect was increased through AATK wt and even more through AATK KD (Fig. S18). Since the loss of *p53* in HCT116 cells had a significant impact on the cell viability, we took another approach and pharmacologically inhibited TP53 in AATK wt and KD expressing clone pools and MCF-7 cells. We observed that the AATK wt-dependent reduction in cell growth of the clone pool is overcome by inhibition of TP53 through Pifithrinα (PFTα) (Fig. S19A). Further, the overall cell growth is significantly higher in the PFTα-treated clone pools compared to solvent-treated cells. This is not seen in the MCF-7 cells after PFTα treatment (Fig. S20B).

To further pinpoint the mechanism of action of AATK, we performed MTS assays for cell viability of HEK293T and MCF-7 cells after exogenous expression of AATK wt and AATK KD (Fig. S20). In comparison to the control, AATK wt significantly decreased the cell viability in HEK293T cells (Fig. S20A). In MCF-7, AATK wt expressing cells were significantly less viable compared to the AATK KD expressing cells (Fig. S20B). Interestingly, the cell viability was significantly increased through AATK KD expression in MCF-7 and HEK293T. These findings were also observed in the AATK wt and AATK KD expressing clone pools (Fig. 5E). The relative cell viability in the AATK wt expressing clone pool was reduced by more than 20% compared to the control clone and nearly 40% compared to the AATK KD expressing clone pool (Fig. 5E).

**Fig. 6:**
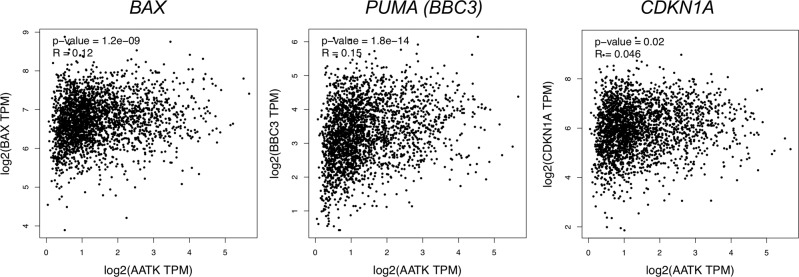
Expression of TP53 target genes is positively correlated with AATK expression in cancer. Pearson correlation of *AATK* and *BAX, BBC3 (PUMA)* or *CDKN1A (p21)* expression in breast cancer, colon adenocarcinoma, lung cancer (adeno- and squamous cell carcinoma) and pancreatic adenocarcinoma was analyzed via GEPIA [45] (TPM = transcripts per million).

Consequently, we generated growth curves of the induced clone pools expressing AATK wt, AATK KD, and the control clone pool (Fig. 5F). The cell counts of the control cells and the AATK KD expressing cells were very similar. In comparison, the AATK wt expressing cells grew significantly slower in comparison to the control cells as well as the AATK KD expressing cells (Fig. 5F). To analyze, the expression of classic TP53 target genes that are associated with *AATK* expression, we performed correlation analysis in BRCA, COAD, lung cancer (adeno- and squamous cell carcinoma), and PAAD via GEPIA [45] (Fig. 6). Here, we observed a positive correlation between the expression of *AATK* and *BAX*, *BBC3 (PUMA)* and *CDKN1A* (Fig. 6) and this was highly significant for the proapoptotic targets *BAX* an*d PUMA* (*p* = 1.2 × 10^−9^ and *p* = 1.8 × 10^−14^, respectively).

## Discussion

Multiple studies have primarily focused on AATK in the nervous system [11, 38, 39, 52], although it was first described being upregulated during apoptosis of myeloid cells [10]. In the past years, AATK has moved into the focus of tumorigenesis. To date, epigenetic inactivation of *AATK* via promoter hypermethylation has been reported in pancreatic ductal adenocarcinoma, lung and breast cancer [15, 16]. In this study, we characterize the loss of *AATK* expression due to promoter hypermethylation (Figs. 1 and 2) and the coinciding poorer overall survival probability in breast, colon, head and neck, lung, and pancreatic adenocarcinoma (Fig. 2). In part, this type of DNA methylation is sufficient to predict an impaired overall survival probability (Figs. S2, S4 and S6). Although we show that *AATK* is epigenetically downregulated in various cancers, further mechanisms of downregulation have been described. Previously, Gao et al. have described post-transcriptional downregulation of AATK via MiR−196a-3p. The expression of AATK was directly negatively regulated by MiR−196a-3p [53]. In the past, research has focused more on MiR in cancer progression [54]. Therefore, this aspect of expression should not be neglected in the future.

Utilizing CRISPR-dCAS9 epigenetic editing, we modulated the promoter activity and expression of *AATK*, thereby further verifying the epigenetic inactivation (Fig. 2). Motivated by the various cancer types with reduced *AATK* expression throughout tumor progression, we analyzed the function of *AATK*. Here, we report that loss of *AATK* expression in various cancer types correlates with gene set enrichments of categories: cell cycle, DNA damage, cancer gene census, and apoptosis (Fig. 3). In addition, we demonstrate that exogenous expression of AATK leads to inhibition of cell growth (Fig. 3A). These observations are in accordance with earlier reports on inhibition of colony formation of a lung cancer and melanoma cell lines, as well as apoptosis induction in pancreatic cancer cell lines through exogenous AATK expression [15–17]. Further, loss of *AATK* expression has been linked to resistance to radiotherapy in the lung cancer cell line A549 [18]. Interestingly our results suggest that AATK induces TP53 phosphorylation in breast cancer cells upon UV radiation (Fig. S13). Conversely, loss of *AATK* decreases UV-induced TP53 phosphorylation (Fig. S14). DNA damage induction via radiotherapy, UV-treatment, and chemotherapeutics has been widely used for treatment of various cancer types [55]. Accordingly, the DNA damage response in cancer cells has been researched extensively and the complex network of action in DNA repair have been focused on [56]. A key regulator of DNA damage response is TP53 and loss of function in various tumors has been linked to resistance to chemotherapy and poor prognosis [57, 58].

Interactome analysis of AATK (Fig. 3), revealed among others previously described binding partners of AATK such as STK39 and PPP1CB [46, 59]. The resulting protein network indicates exogenous expression of AATK to be linked to “vesicle-mediated transport” (Fig. 3C), which we did not follow up on, as this aspect has been researched extensively in neuronal cells [60]. However, the network also linked expression of AATK to “regulation of cell proliferation” (Fig. 3D), which we found corroborated by whole transcriptome analysis (Fig. 4A). Among others, we identified that the key cell cycle regulators *CCND1* and *WEE1* are downregulated (Fig. 4B). We validated their downregulation through overexpression of AATK in a kidney cell line and two glioblastoma cell lines and found their expression induced by RNAi of *AATK* in a breast cancer, a skin cancer and a kidney cell line (Fig. 4C, D). Remarkably, AATK mediates the decrease in the expression of *CCND1* and *WEE1* (Fig. 4E). Interestingly, both are well-characterized drivers of cell cycle progression [61, 62]. In a multitude of cancers, *CCDN1* overexpression is linked to poorer survival [62–65]. *WEE1* is a de facto pan-cancer essential gene [66] associated with negative prognostic factors, including lymph node involvement, induction of metastasis, increased proliferation markers, and resistance to cancer treatments [67–70]. Furthermore, we demonstrated that decreased expression of *CCND1* and *WEE1* mediated through AATK depends on its kinase activity.

In an attempt to validate kinase activity, as well as to characterize the signaling network and downstream targets of AATK, we performed peptide-based activity assays for tyrosine kinases (PTK) and serine/threonine kinases (STK). Based on homology to other members of the Lemur tyrosine kinase family which are known Ser/Thr kinases [18, 64], AATK was presumed to be a Ser/Thr kinase. This assumption was experimentally validated by the identification of a number of natural-protein-derived substrate peptides as phosphorylated at serine and/or threonine residues upon exposure to AATK wt as compared to AATK KD (Fig. S11). Further, GO term analysis of the STRING network, revealed a significant role of AATK in the regulation of cell proliferation (Fig. S12). This finding was further corroborated by overrepresentation analysis in our AATK interactome dataset (Fig. 3D). Taken together, these findings demonstrate that AATK is involved in cell proliferation and that exogenous reconstitution of AATK expression leads to inhibition of cell growth (Fig. 3A). The activity of AATK as a Ser/Thr kinase was further analyzed in detail because to the best of our knowledge, no substrate phosphorylated by AATK has so far been found. With the kinomic profile of AATK, we had the opportunity to compare the kinase activity of AATK with well-characterized kinases (Fig. 5A). Its kinase profile revealed Reactome pathways which included “Regulation of TP53 activity” and “Regulation of TP53 degradation” as well as “Generic transcription pathway” (Fig. 5B). This finding indicates that AATK mimics the activity of kinases involved in the regulation of TP53. Our results also suggest that bona fide TP53 target genes like *BAX*, *PUMA*, and *CDKN1A* are positively associated with AATK expression (Fig. 6).

A multitude of TP53 phosphorylation sites have been detected that affect its activity and degradation [71, 72]. Among others, phosphorylation of TP53 at Ser366 by CHEK2 has been described in the context of TP53 stabilization and transactivation [51]. In vivo, we observed an increase of TP53Ser366 phosphorylation through exogenous AATK expression and a decrease through the kinase-dead mutant of AATK (Fig. 5C). In vitro kinase assay supported that AATK is a Ser/Thr kinase phosphorylating TP53 at Ser366 (Fig. 5D). We further demonstrated that the kinase activity of AATK is required for cell viability (Fig. 5E and Fig. S20) and growth inhibition (Fig. 5F) likely as a result of TP53 phosphorylation. Interestingly, decreased *CCND1* expression is linked to increased stability of TP53 [73].

Deregulated G1 checkpoint in cancers often leads to a strong dependency on G2/M checkpoint for prevention of mitotic catastrophe due to DNA damage [56]. Interestingly, WEE1 is the gatekeeper of the G2/M checkpoint [74]. Thus a high reliance on G2/M checkpoint is required in cancer cells with deregulated G1 checkpoint [74]. Newest research on WEE1, has once more pinpointed the efficacy of WEE1 inhibitors in combination with genotoxic stress as approach in cancer treatment [75]. This therapeutic strategy leads to activation of CDK1 and rapid cell cycle progression without proper DNA damage response. Consequently, mitotic catastrophe due to DNA damage cannot be prevented [70].

On the one hand, CCND1 is required for G1/S transition and overexpression is an early event in tumorigenesis [63]. On the other hand, overexpression of WEE1 in cancer strengthens G2/M checkpoint for DNA damage response and thereby supports cell proliferation [70]. Considering, that the expression of *AATK* correlates with DNA damage (Fig. 3), UV-induced TP53 phosphorylation is dependent on *AATK* expression (Fig. S14) and the kinase activity of AATK (Fig. S13) and that it inhibits cell growth (Fig. 5E, F), the necessity for further research on AATK is emphasized.

In summary, we demonstrate for the first time that AATK acts as a Ser/Thr kinase and that TP53 is its direct target. In addition, the inhibition of cell cycle progression and the decrease of *CCND1* and *WEE1* expression facilitated by AATK is dependent on its kinase activity.

In conclusion, the loss of *AATK* via epigenetic silencing in various cancers drives uncontrolled cell growth by interfering with a hypothesized signaling cascade including TP53 phosphorylation and subsequent reduction in expression of the key cell cycle regulators *CCND1* and *WEE1*. Loss of *AATK* expression is observed in multiple tumor entities and correlates with reduced patient survival. Based on this observation and given our findings, in the future AATK should be considered a biomarker for the characterization of tumors and in the decision of adequate treatment options. Detection of *AATK* silencing (e.g., by promoter hypermethylation) may thus be of value to precision medicine.

## Supplementary information

Supplemental Table S1

Supplemental figures

LC-MSMS Parametrization

Dataset original Western blots

Dataset Sanger sequences

Dataset original qPCR

## Data Availability

Microarray data are available in the ArrayExpress database (www.ebi.ac.uk/arrayexpress) under accession number E-MTAB-11198.
