# Supplementary material for: Epigenetically silenced apoptosis-associated tyrosine kinase (AATK) facilitates a decreased expression of Cyclin D1 and WEE1, phosphorylates TP53 and reduces cell proliferation in a kinase-dependent manner
Source: Cancer Gene Ther. 2022 Jul 28;29(12):1975–87. doi: 10.1038/s41417-022-00513-x (PMC9750878; doi:10.1038/s41417-022-00513-x)
Supplement: Supplementary file 1 — Supplemental Table S1 [file 41417_2022_513_MOESM1_ESM.pdf]

Supplementary table S1: List of primers

CoBRA-PCR	
1. Upper	GTTGGGTGATGYGGYGGTT
2. Upper	GTTTTTTTTTAATTTTAGTTTYGTTTTTAGTT
1. and 2.Lower	ATTTATACTAAAACCCAAAACCTACCC
**Pyrosequencing -PCR**	
1. Upper	GTTGGGTGATGYGGYGGTT
2. Upper	GTTTTTTTTTAATTTTAGTTTYGTTTTTAGTT
1. and 2. Lower (with biotin tag)	ATTTATACTAAAACCCAAAACCTACCC
**Expression analysis**	
ANXA1	GCAAGAAGGTAGAGATAAAGACACT
	GCGACATCCGAGGATGGATT
NOVA1	GGACCAATACGGGCGAAGAC
	AGACAGCTTGATGGTGGCTC
CCND1	AAGTTGCAAAGTCCTGGAGCC
	GATGGTTTCCACTTCGCAGC
WEE1	CACACGCCCAAGAGTTTGC
	CACTTGAGGAGTCTGTCGCA
GAPDH	TGGAGAAGGCTGGGGCTCAT
	GACCTTGGCCAGGGGTGCTA
β-ACTIN	CCTTCCTTCCTGGGCATGGAGTC
	CGGAGTACTTGCGCTCAGGAGGA
AATK	TGGCCTGGCTCACTGCAAGTACAG
	CCCAGATGGTCACGCCCAGG
**Genomic region targeted via CRISPR/CAS9**	
gDNA AATK	GGGCGGGGAAAGGGTTAATTTC
	CTCCAAGCCCCCTTCTGTGTC

Primer sequences are listed in 5'-3' direction
