# Supplementary material for: Epigenetically silenced apoptosis-associated tyrosine kinase (AATK) facilitates a decreased expression of Cyclin D1 and WEE1, phosphorylates TP53 and reduces cell proliferation in a kinase-dependent manner
Source: Cancer Gene Ther. 2022 Jul 28;29(12):1975–87. doi: 10.1038/s41417-022-00513-x (PMC9750878; doi:10.1038/s41417-022-00513-x)
Supplement: Supplementary file 2 — Supplemental figures [file 41417_2022_513_MOESM2_ESM.pdf]

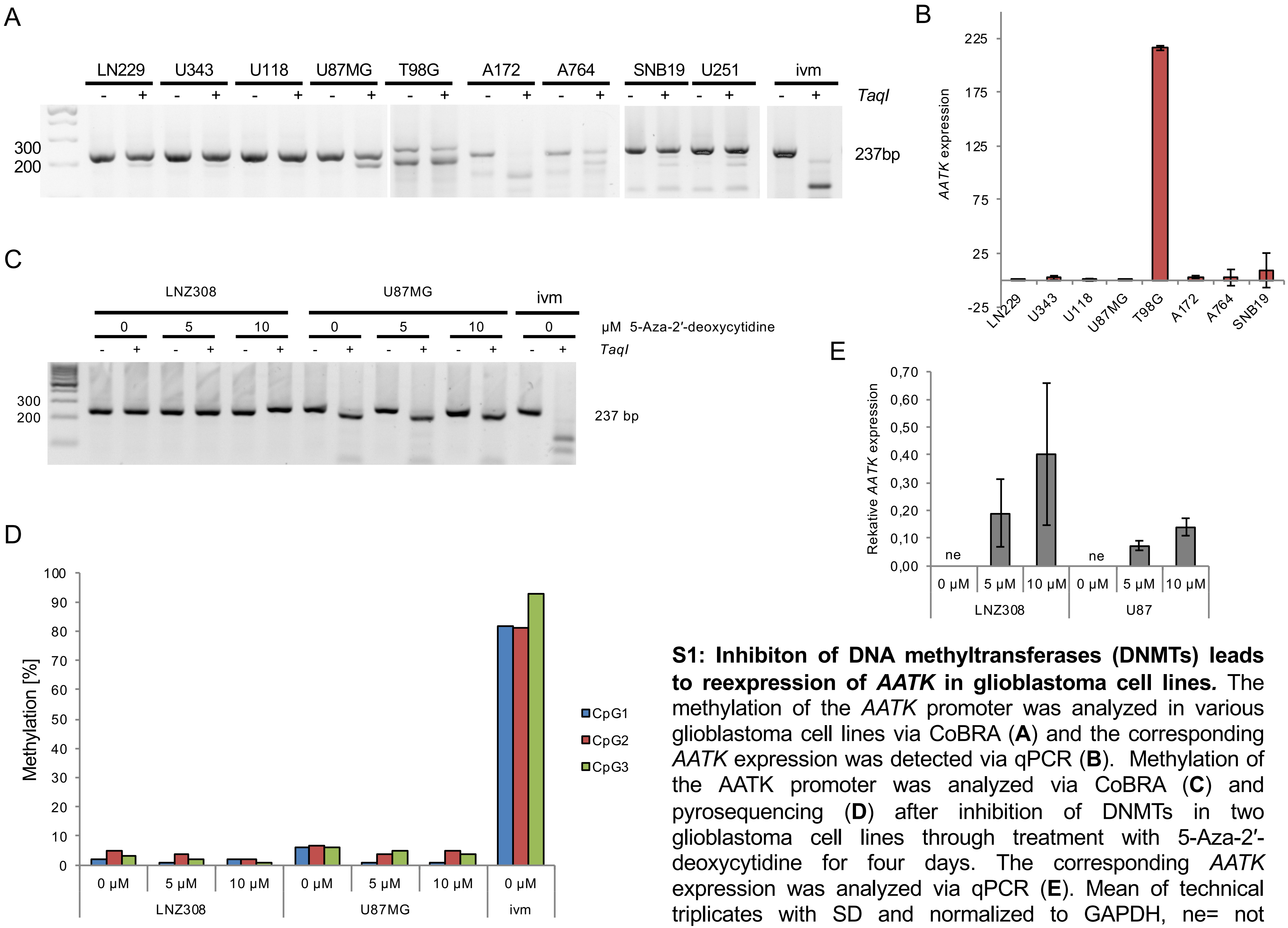

S1: Inhibiton of DNA methyltransferases (DNMTs) leads to reexpression of *AATK* in glioblastoma cell lines. The methylation of the *AATK* promoter was analyzed in various glioblastoma cell lines via CoBRA (**A**) and the corresponding *AATK* expression was detected via qPCR (**B**). Methylation of the AATK promoter was analyzed via CoBRA (**C**) and pyrosequencing (**D**) after inhibition of DNMTs in two glioblastoma cell lines through treatment with 5-Aza-2′-deoxycytidine for four days. The corresponding *AATK* expression was analyzed via qPCR (**E**). Mean of technical triplicates with SD and normalized to GAPDH, ne= not expressed.

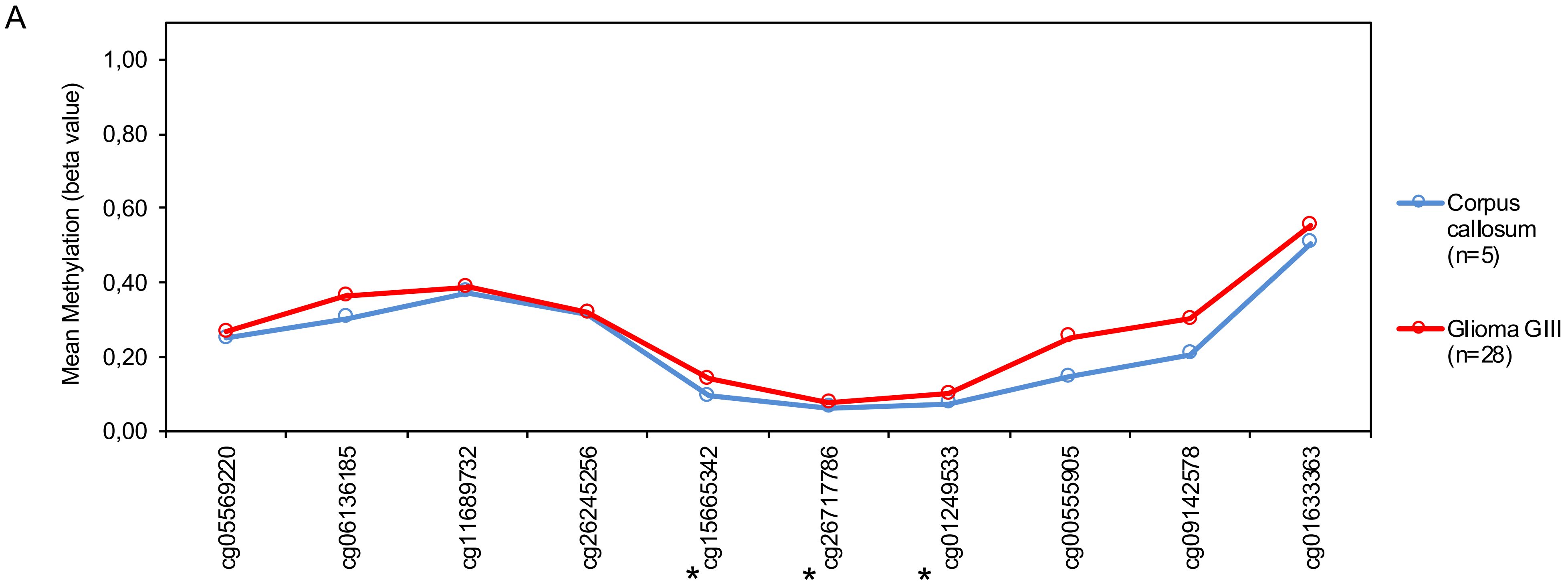

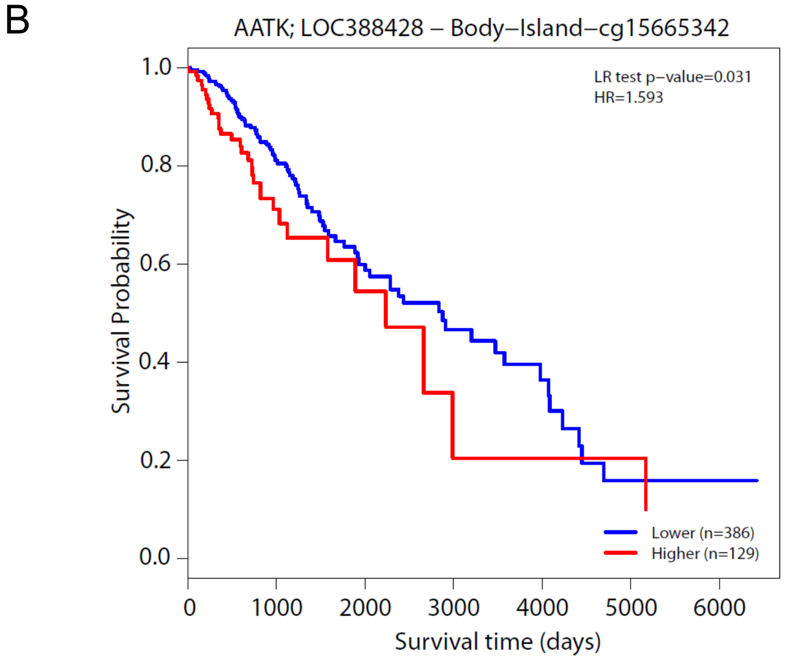

S2: Methylation of cg15665342 correlates with poorer overall survival in glioma. A Mean methylation of normal brain (corpus callosum) compared to grade II glioma is displayed. Via Geo2R single values of seven probes for the shores and CGI of AATK were extracted for tha data set GSE123678. Significance via one-way Anova with *$p < 0.05$. **B** *AATK* methylation-dependent survival (cg15665342) of glioma patients (red: high methylation, blue: low methylation) (https://biit.cs.ut.ee/methsurv/).

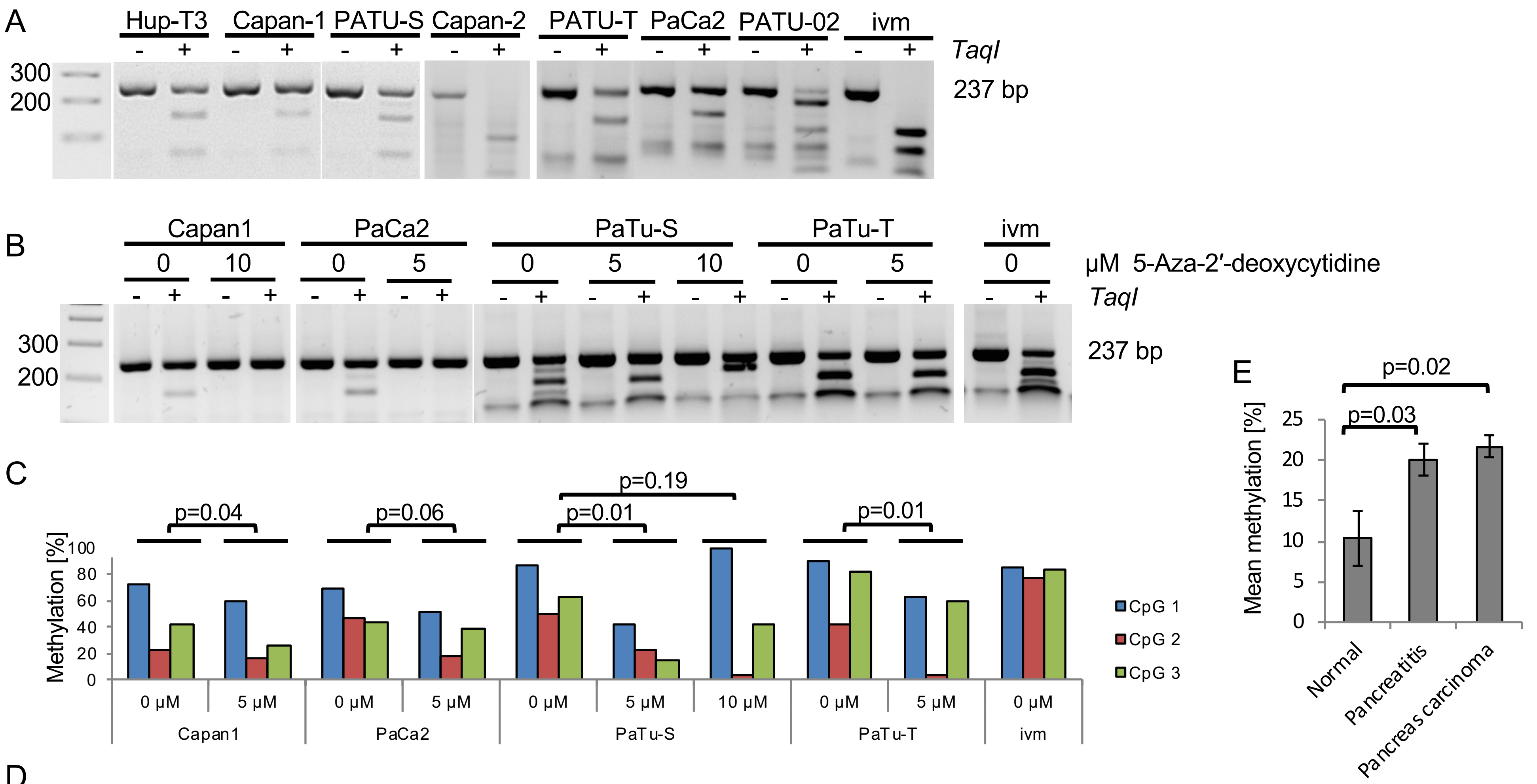

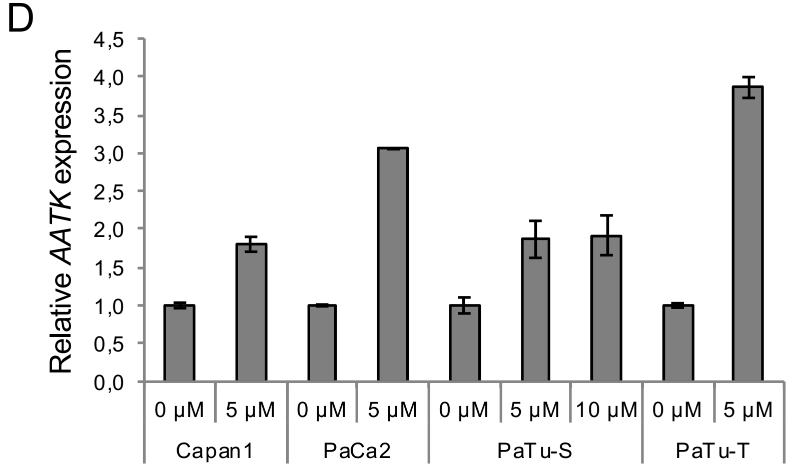

S3: Methylation of *AATK* in various pancreatic cancer cell lines is reversed by treatment with 5-Aza-2′-deoxycytidine. A CoBRA of eight pancreatic cancer cell lines. CoBRA (**B**) and pyrosequencing (mean methylation of all CpG dinucleotides; unpaired, two-tailed t-test) (**C**) of *AATK* methylation and qRT-PCR (**D**) for AATK expression after treatment with 5-Aza-2′-deoxycytidine. **E** *AATK* methylation-dependent of normal pancreatic tissues (n=4), pancreatitis samples (n=18) and pancreatic carcinoma samples (n=45). Mean methylation of all CpG dinucleotides with SD; unpaired, two-tailed t-test,

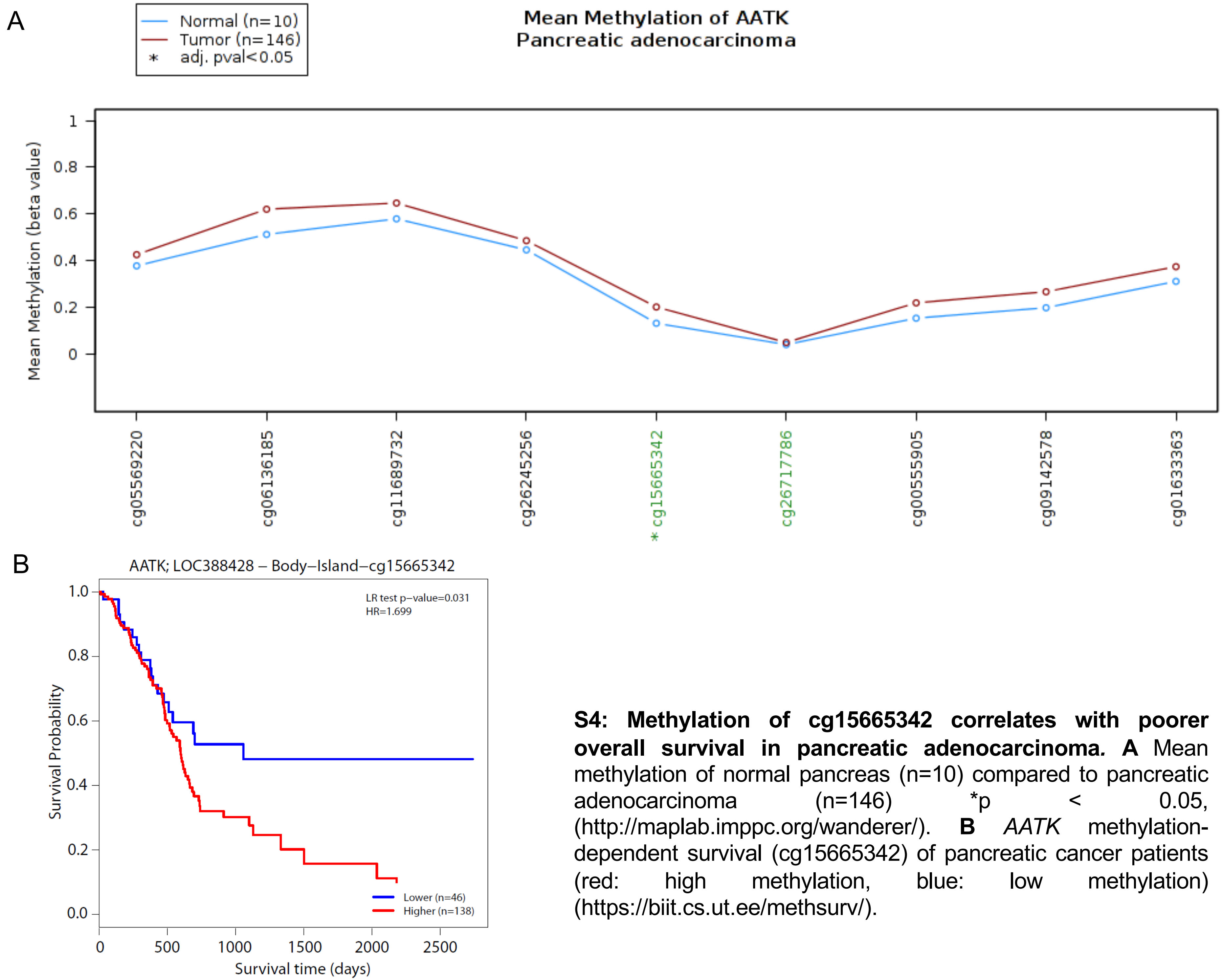

S4: Methylation of cg15665342 correlates with poorer overall survival in pancreatic adenocarcinoma. A Mean methylation of normal pancreas (n=10) compared to pancreatic adenocarcinoma (n=146) *p < 0.05, (http://maplab.imppc.org/wanderer/). **B** *AATK* methylation-dependent survival (cg15665342) of pancreatic cancer patients (red: high methylation, blue: low methylation) (https://biit.cs.ut.ee/methsurv/).

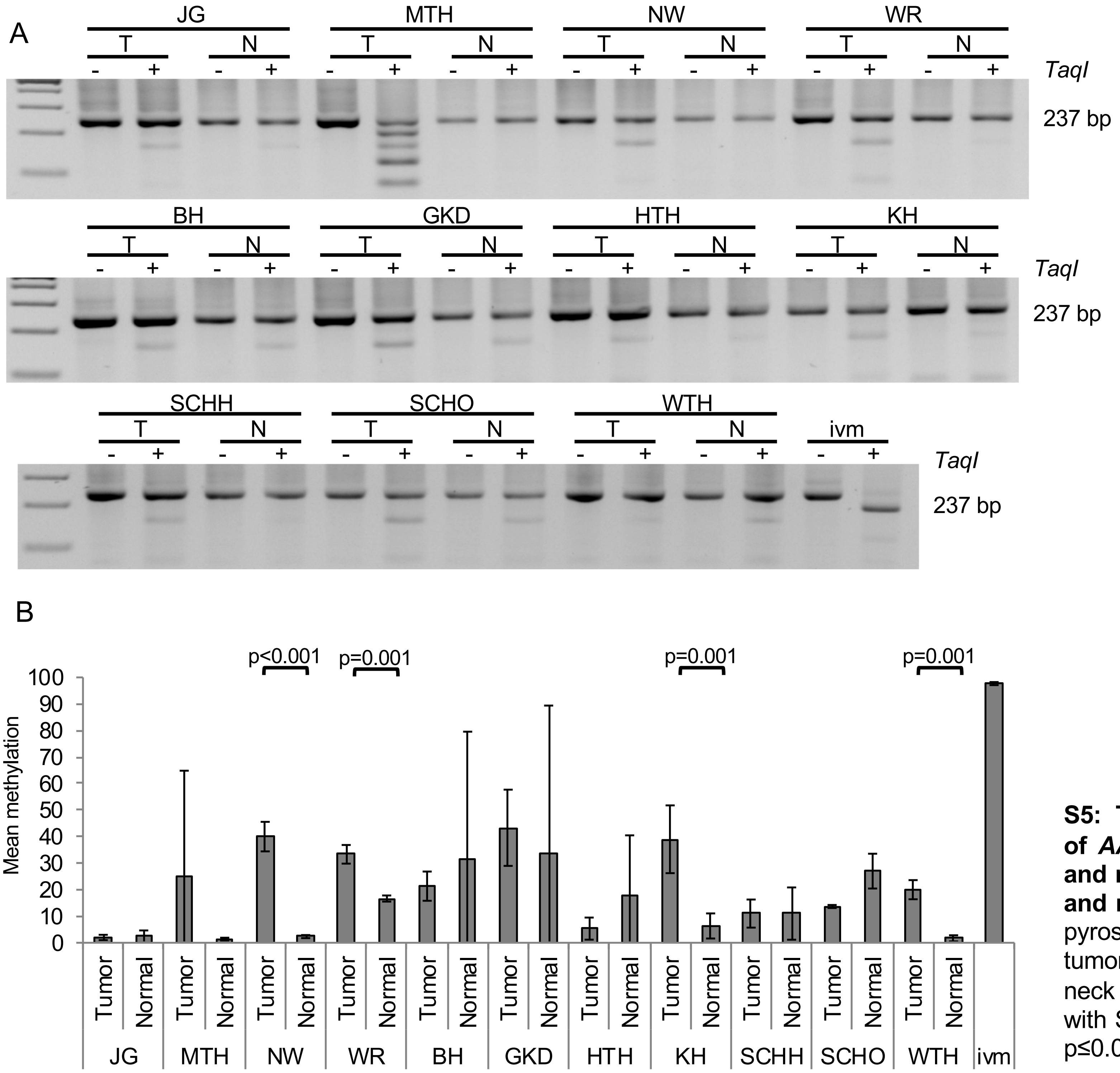

S5: Tumor-specific methylation of *AATK* in matching tumor (T) and normal (N) samples of head and neck cancer . CoBRA (**A**) an pyrosequencing (**B**) of matching tumor and normal samples of head neck cancer. Mean methylation with SD; unpaired two-tailed t-test; p≤0.01.

A

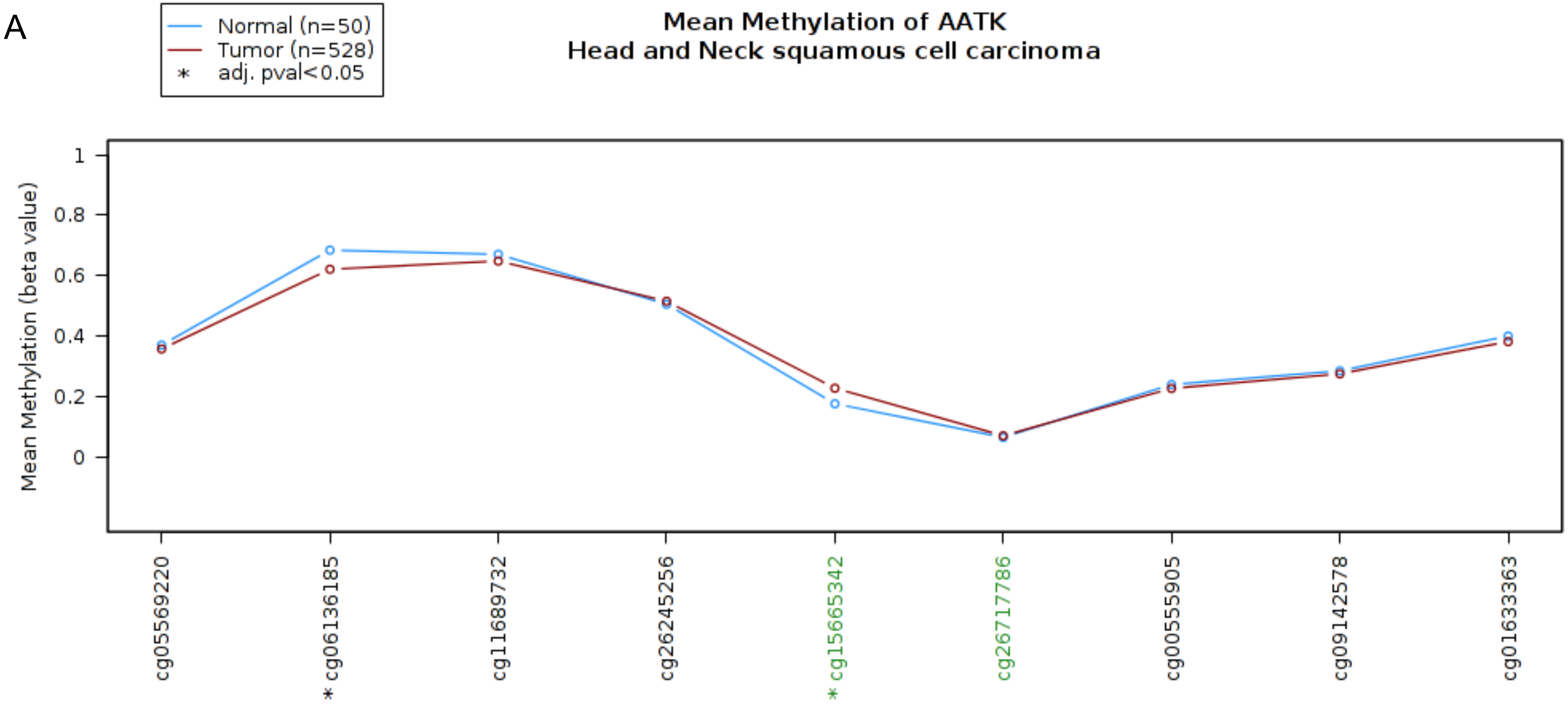

B

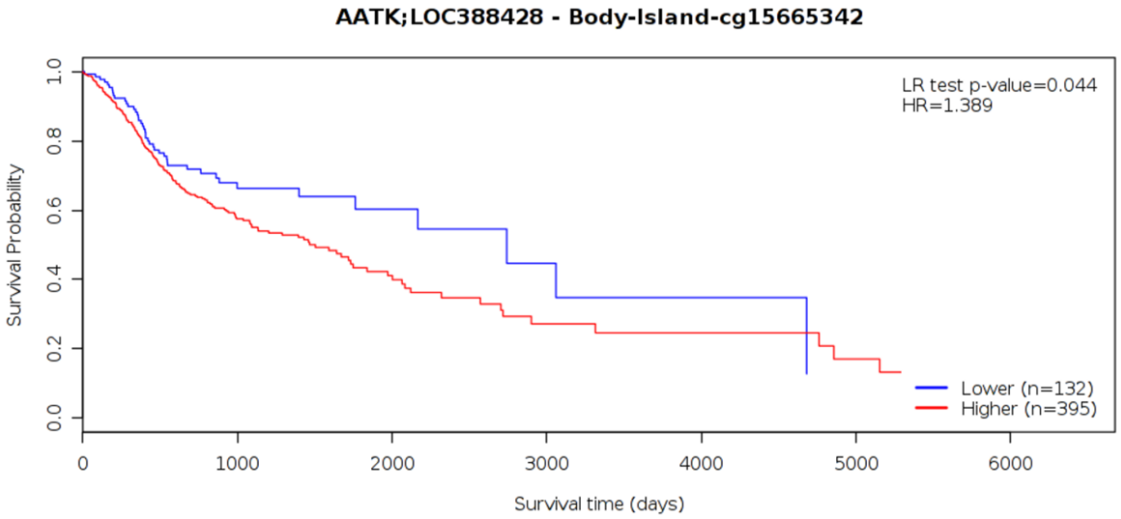

S6: Methylation of cg15665342 correlates with poorer overall survival in head and neck squamous cell carcinoma. A Mean methylation of normal head and neck samples (n=50) compared to head and neck squamous cell carcinoma (n=528) *p < 0.05, (http://maplab.imppc.org/wanderer/). **B** *AATK* methylation-dependent survival (cg15665342) of head and neck squamous cell cancer patients (red: high methylation, blue: low methylation) (https://biit.cs.ut.ee/methsurv/).

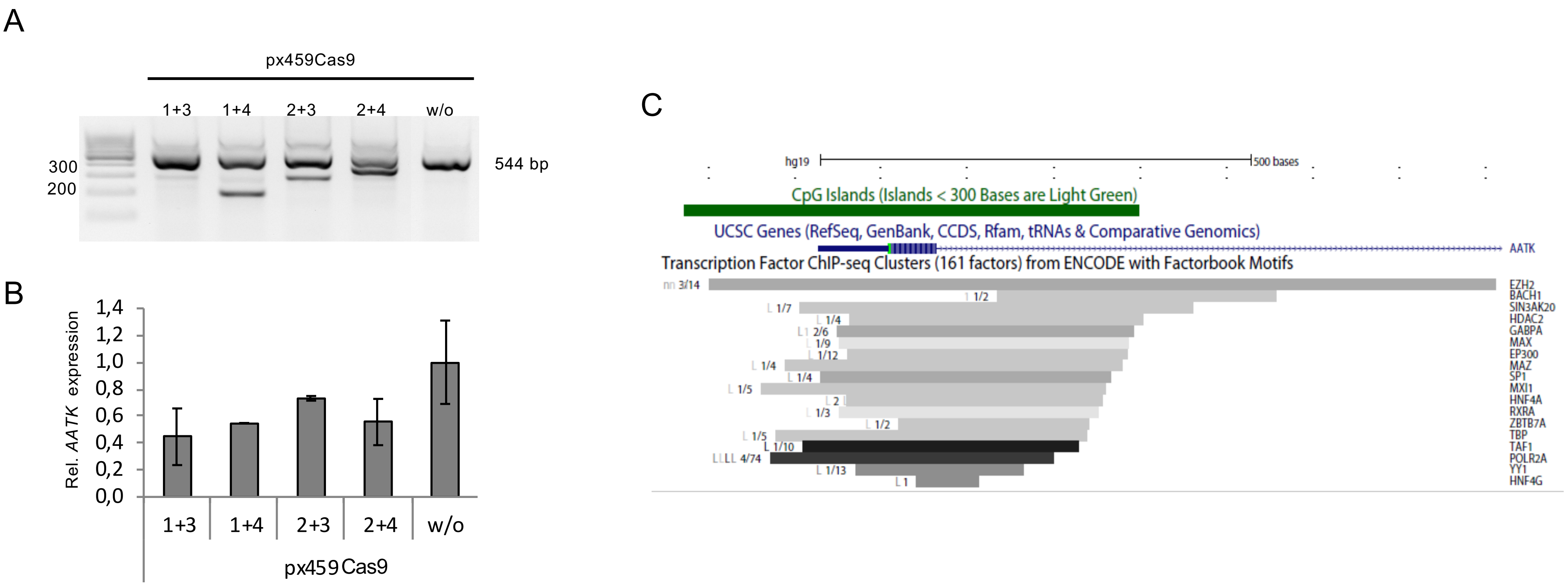

S7: Targeting the genomic region of *AATK* with Crispr AATK guide RNA oligos. A PCR of targeted genomic region of *AATK* with expected genomic deletion and corresponding relative expression of *AATK.* PCR product were sequenced and Sanger sequences are depicted as an original data set in the supplement. **B** 24 h after combined transfection with *AATK* guide RNAs *AATK.* **C** Transcription factor ChipSeq Clusters (161 factors) from ENCODE with factorbook motifs display the binding potential of EZH2 and EP300 to the *AATK* promoter region.

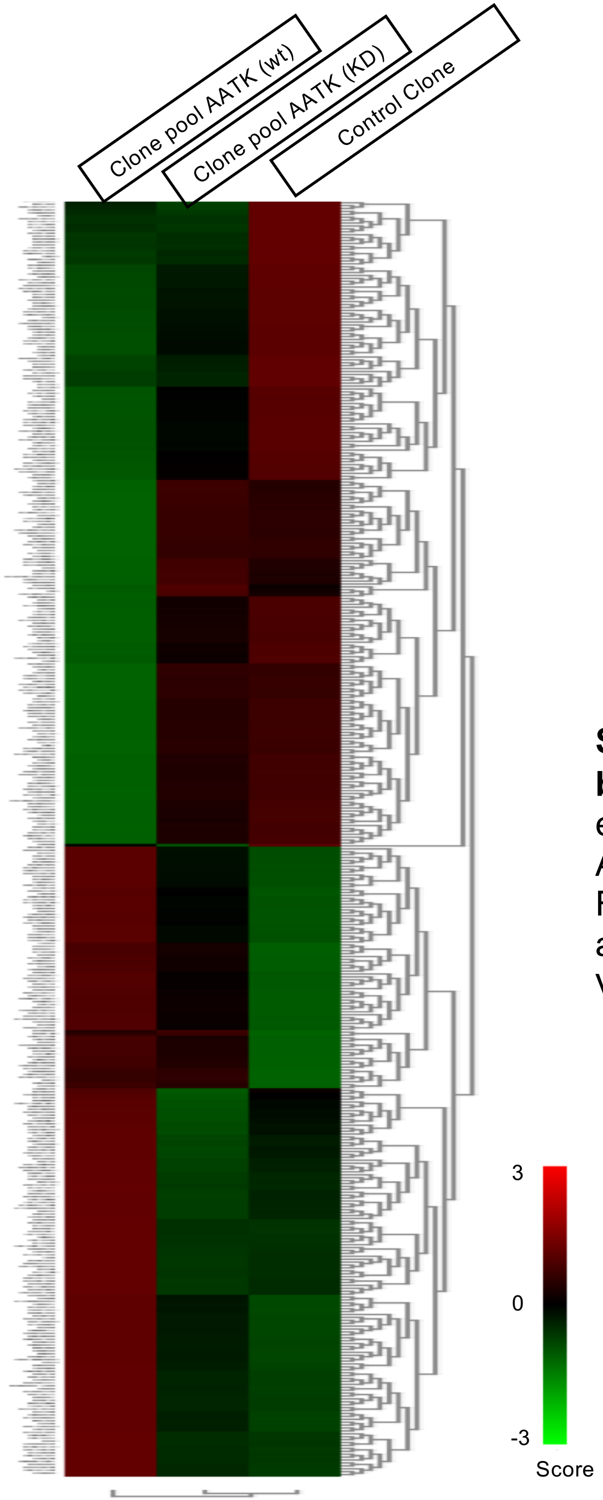

S8: Heatmap of deregulated genes (DEGs) detected by whole transcriptome analysis. Clustering of gene expression of the 500 genes deregulated via induction of AAKT wt expression in comparison to the control clone. Furthermore the AATK KD (kinase dead) clone pool is also displayed. Visualized via the Genomics Analysis and Visualization Platform R2 (Molenaar, Koster et al. 2012).

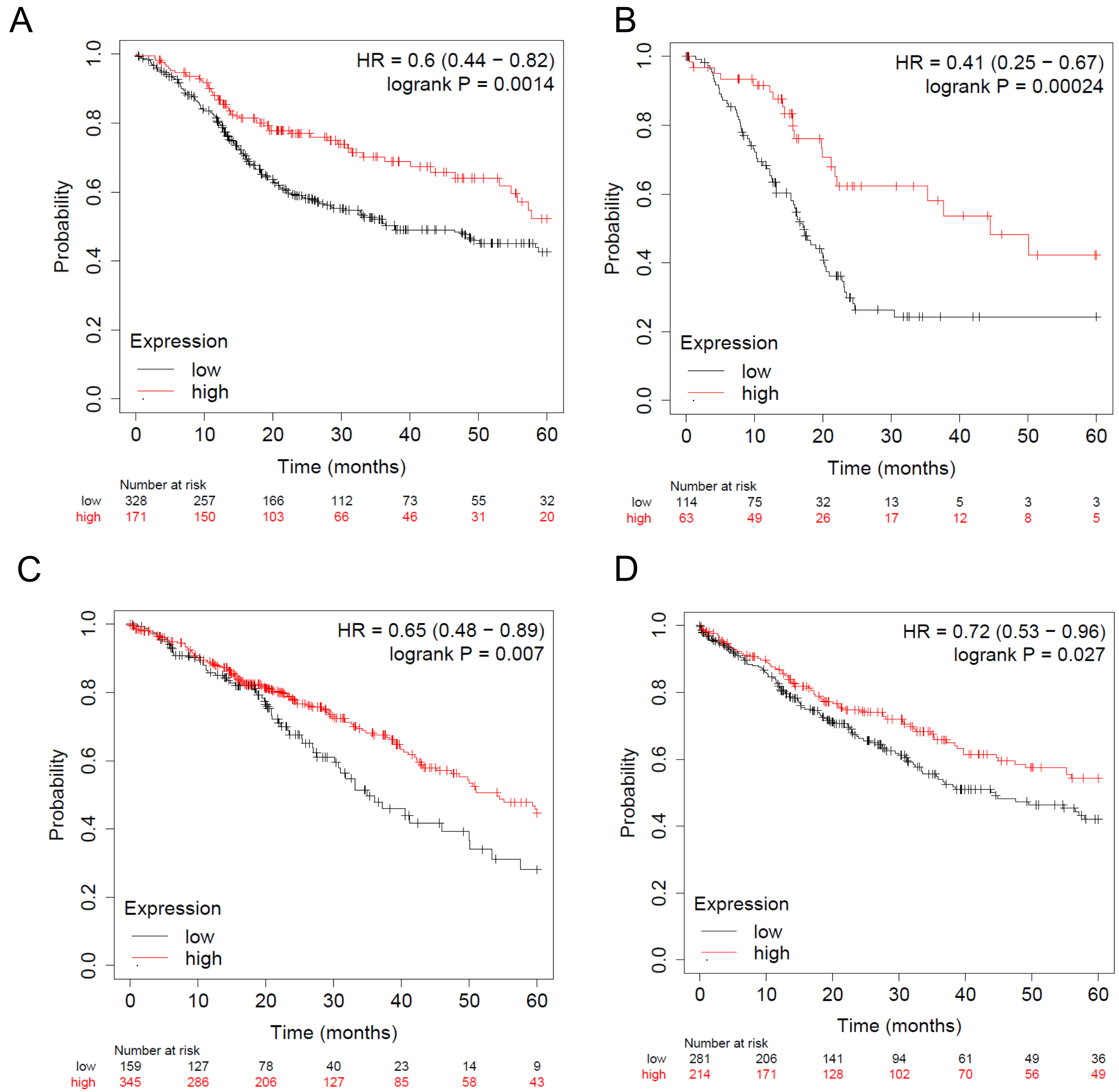

S9: Overall survival probability depends on high *AATK* and low *CCND1* expression in various cancers. Kaplan-meier plots of head and neck squamous cell carcionma (n=499) (**A**), pancreatic ductal carcinoma (n=177) (**B**), lung adenocarcinoma (n=504) (**C**) and lung squamous cell carcinoma (n=495) (**D**) with high *AATK* and low *CCND1* expression (red) or high *CCND1* expression and low *AATK* (black). (Nagy A, Munkacsy G, Gyorffy B: (Pancancer survival analysis of cancer hallmark genes, Sci Rep. 2021 Mar 15;11(1):6047)).

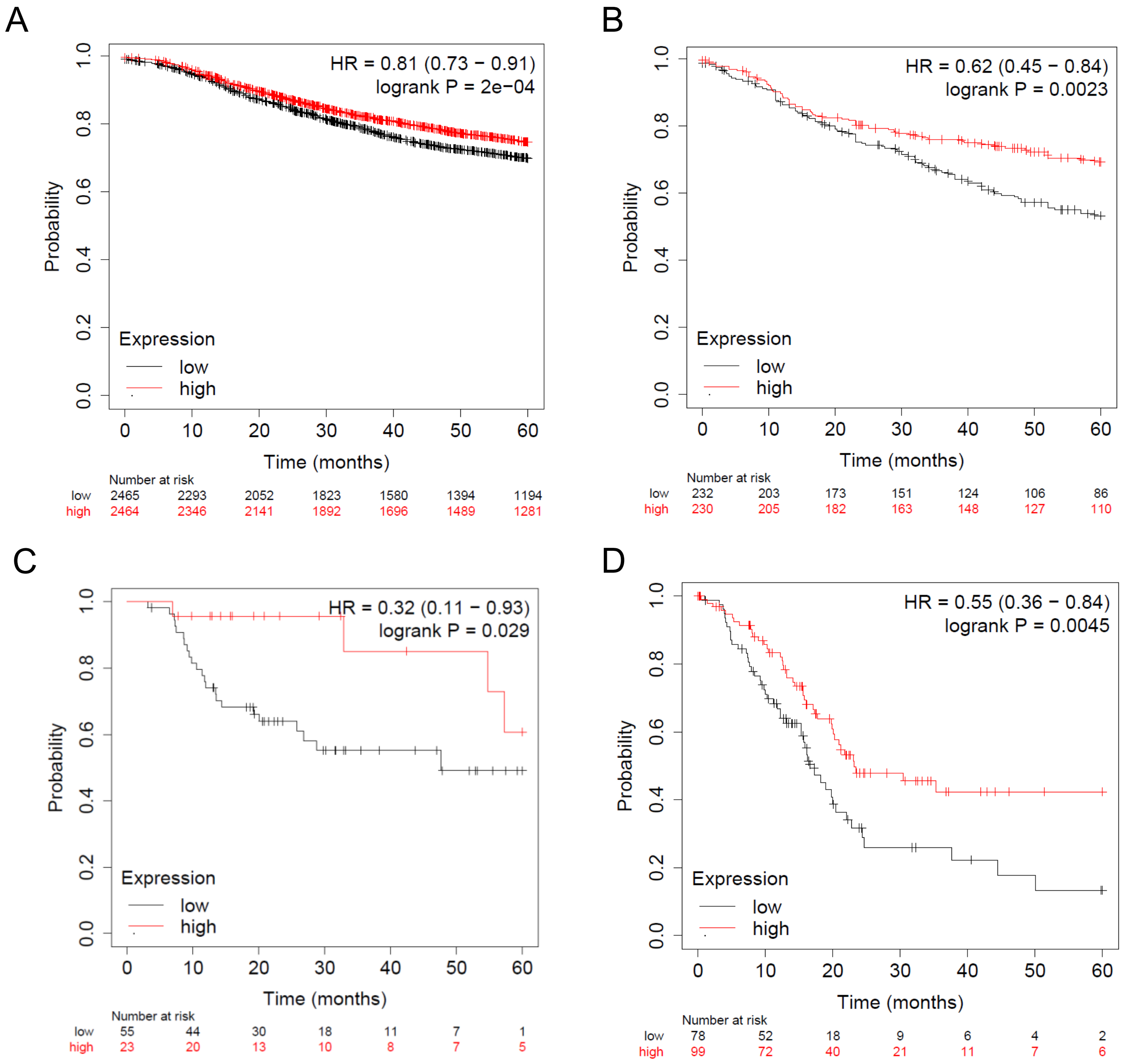

S10: Overall survival probability depends on high *AATK* and low *WEE1* expression in various cancers. Kaplan-meier plots of breast cancer (n=4929) (**A**) (Győrffy B. Survival analysis across the entire transcriptome identifies biomarkers with the highest prognostic power in breast cancer, Computational and Structural Biotechnology Journal, 2021, https://doi.org/10.1016/j.csbj.2021.07.014), squamous cell lung cancer (n=462) (**B**) (Gyorffy B, Surowiak P, Budczies J, Lanczky A. Online survival analysis software to assess the prognostic value of biomarkers using transcriptomic data in non-small-cell lung cancer, PLoS One, 2013 Dec 18;8(12):e82241. doi: 10.1371/journal.pone.0082241), head and neck squamous cell carcinoma (stg.3) (n=78) (**C**) and pancreatic ductal carcinoma (n=177) (**D**) (Nagy A, Munkacsy G, Gyorffy B: (Pancancer survival analysis of cancer hallmark genes, Sci Rep. 2021 Mar 15;11(1):6047) with high *AATK* expression and low *WEE1* (red) and high WEE1 and low *AATK* expression (black).

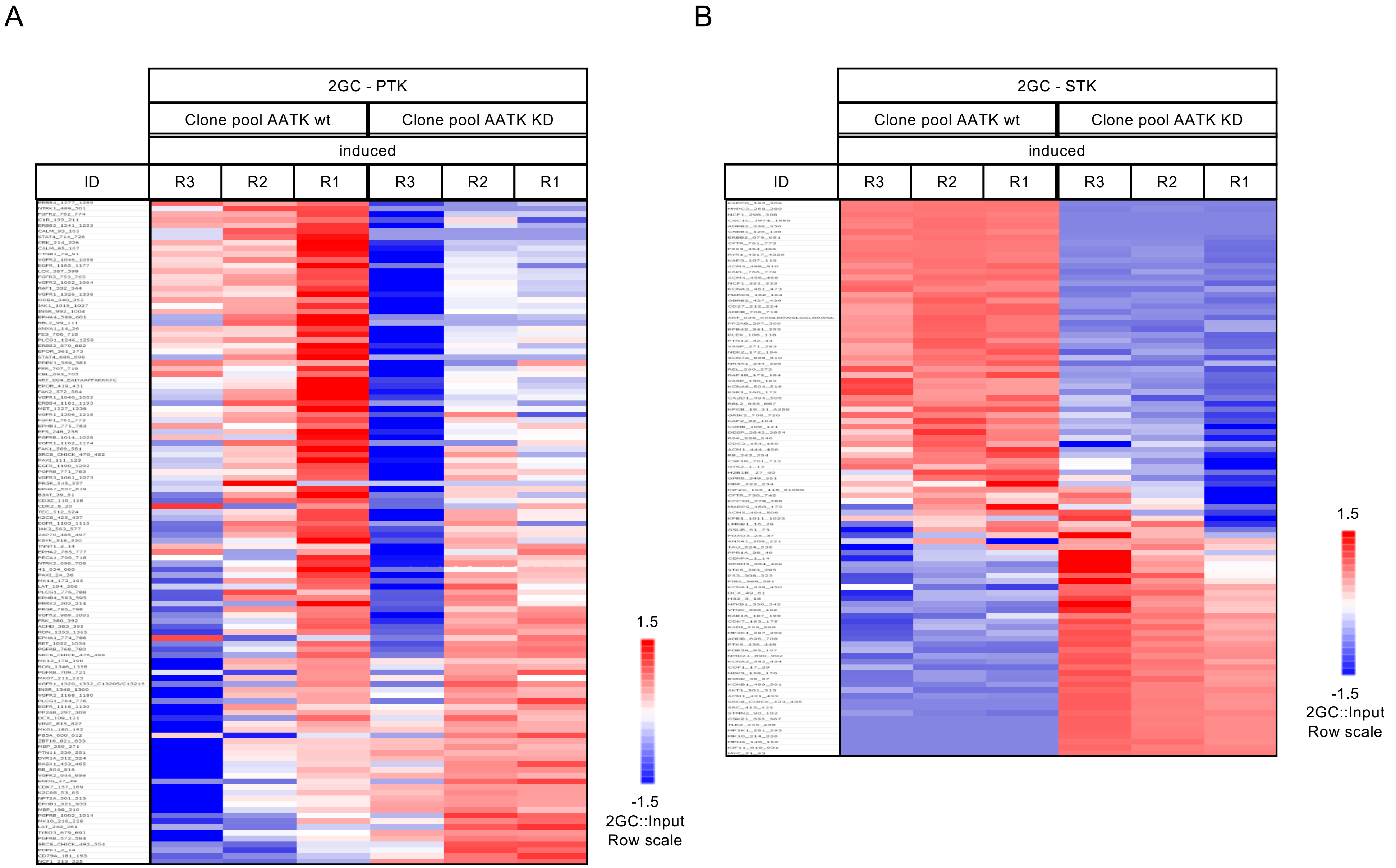

S11: AATK is a Ser/Thr kinase. Peptide-based kinase activity assays were performed with an AATK wt and AATK KD expressing clone pool with doxycycline for 24 h. The basal kinomic activity profiles are displayed from three independent experiments (R1-R3). Unsupervised hierarchical clustering of basal **A** Tyr (PTK) and **B** Ser/Thr (STK) kinomic profiles displaying log transformed slope-exposure for peptides. Red indicates relative increased signal. Blue indicates the opposite.

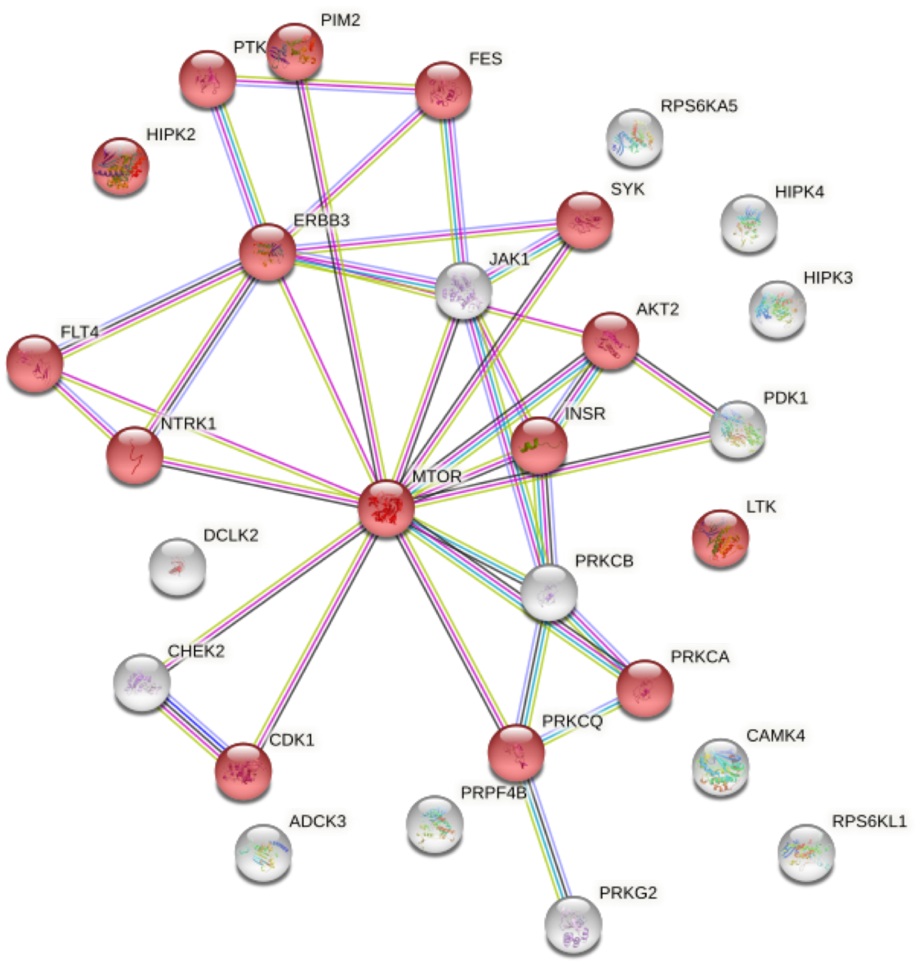

Biological process (Gene ontology)		
GO-Term	Description	FDR
GO:0006468	Protein phosphorylation	4.54E-33
GO:0018193	Peptidyl-amino acid modification	2.76E-23
GO:0018105	Peptidyl-serine phosphorylation	4.14E-20
GO:0046777	Protein autophosphorylation	3.37E-14
GO:0035556	Intracellular signal transduction	7.04E-09
GO:0018107	Peptidyl-threonine phosphorylation	1.06E-08
GO:0042327	Positive regulation of phosphorylation	3.84E-08
GO:0007169	Transmembrane receptor protein tyrosine kinase signaling pathway	4.44E-08
GO:0007167	Enzyme linked receptor protein signaling pathway	7.16E-08
GO:0019220	Regulation of phosphate metabolic process	1.79E-07
GO:0001934	Positive regulation of protein phosphorylation	2.05E-07
GO:0042325	Regulation of phosphorylation	4.32E-07
GO:0042127	Regulation of cell population proliferation	4.80E-07
GO:0043066	Negative regulation of apoptotic process	6.37E-07
GO:0016572	Histone phosphorylation	6.88E-07
GO:0001932	Regulation of protein phosphorylation	1.12E-06
GO:0007165	Signal transduction	1.72E-06
GO:0042981	Regulation of apoptotic process	2.21E-06
GO:0012501	Programmed cell death	3.25E-06
GO:0051094	Positive regulation of developmental process	6.03E-06
GO:0006915	Apoptotic process	8.83E-06
GO:2000026	Regulation of multicellular organismal development	9.18E-06
GO:0051240	Positive regulation of multicellular organismal process	9.76E-06

S12: Network of kinases from upstream kinase analysis of PTK and STK . Peptide-based kinase activity assays were performed with an AATK wt and AATK KD expressing clone pool with doxycycline for 24 h. The basal kinomic activity profiles of PTK and STK arrays were analyzed in an upstream kinase analysis. The significantly determined upstream kinases that compare to the activity profile of AATK wt were subjected to STRING analysis (left) and GO-terms of biological processes highly significantly enriched are displayed (right). Bubbles represent kinases, lines highlight interactions and red depicts kinases which are found in GO-term „regulation of cell population proliferation“.

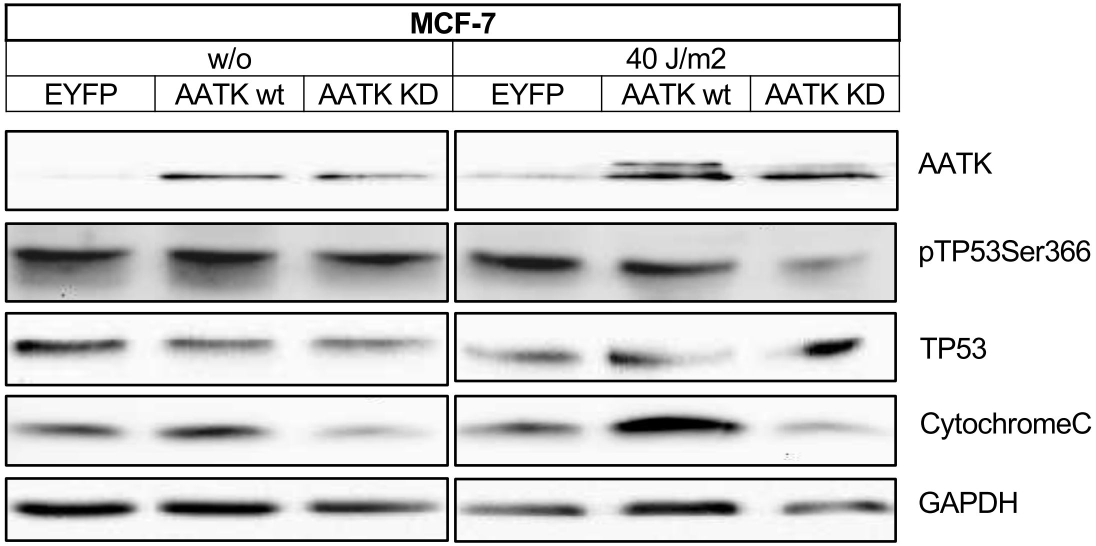

S13: Activation of TP53 via UV irradation via phosphorylation at Ser366 in MCF-7 is increased through AATK wt. Starved MCF-7 cells were transfected with EYFP (empty control), AATK wt and AATK KD for 24 h. Thereafter the cells were subjected to 40 J/m^2 UV irradiation and incubation with full media for 30 min. Immunoblotting of cell lysate for pTP53Ser366 and total TP53 protein levels are displayed.

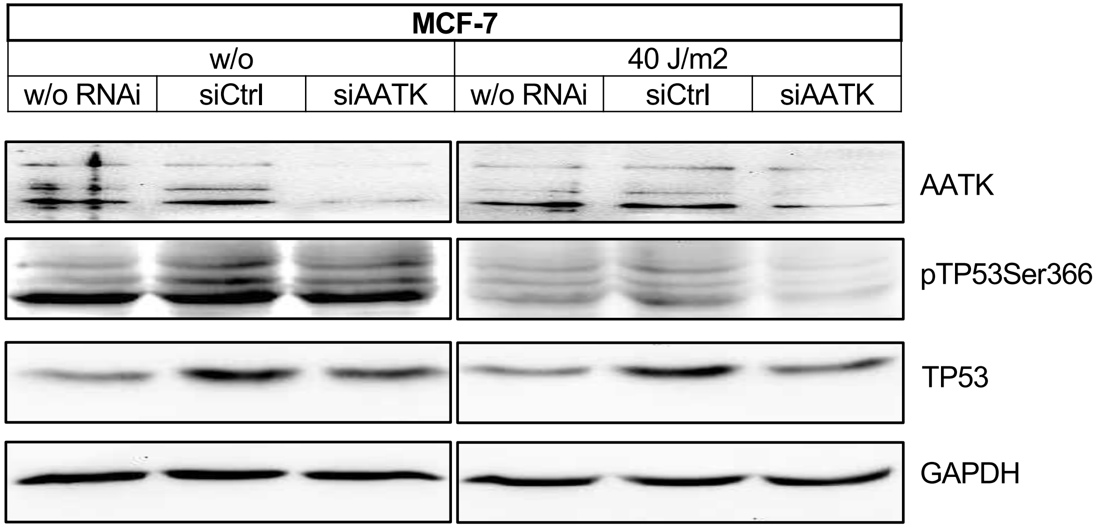

S14: Activation of TP53 via UV irradation via phosphorylation at Ser366 in MCF-7 is decreased through knockdown of *AATK*. Starved MCF-7 cells were transfected without RNAi, with siCtrl or siAATK for 72 h. Thereafter the cells were subjected to 40 J/m^2 UV irradiation and incubation with full media for 30 min. Immunoblotting of cell lysate for pTP53Ser366 and total TP53 protein levels are displayed.

A

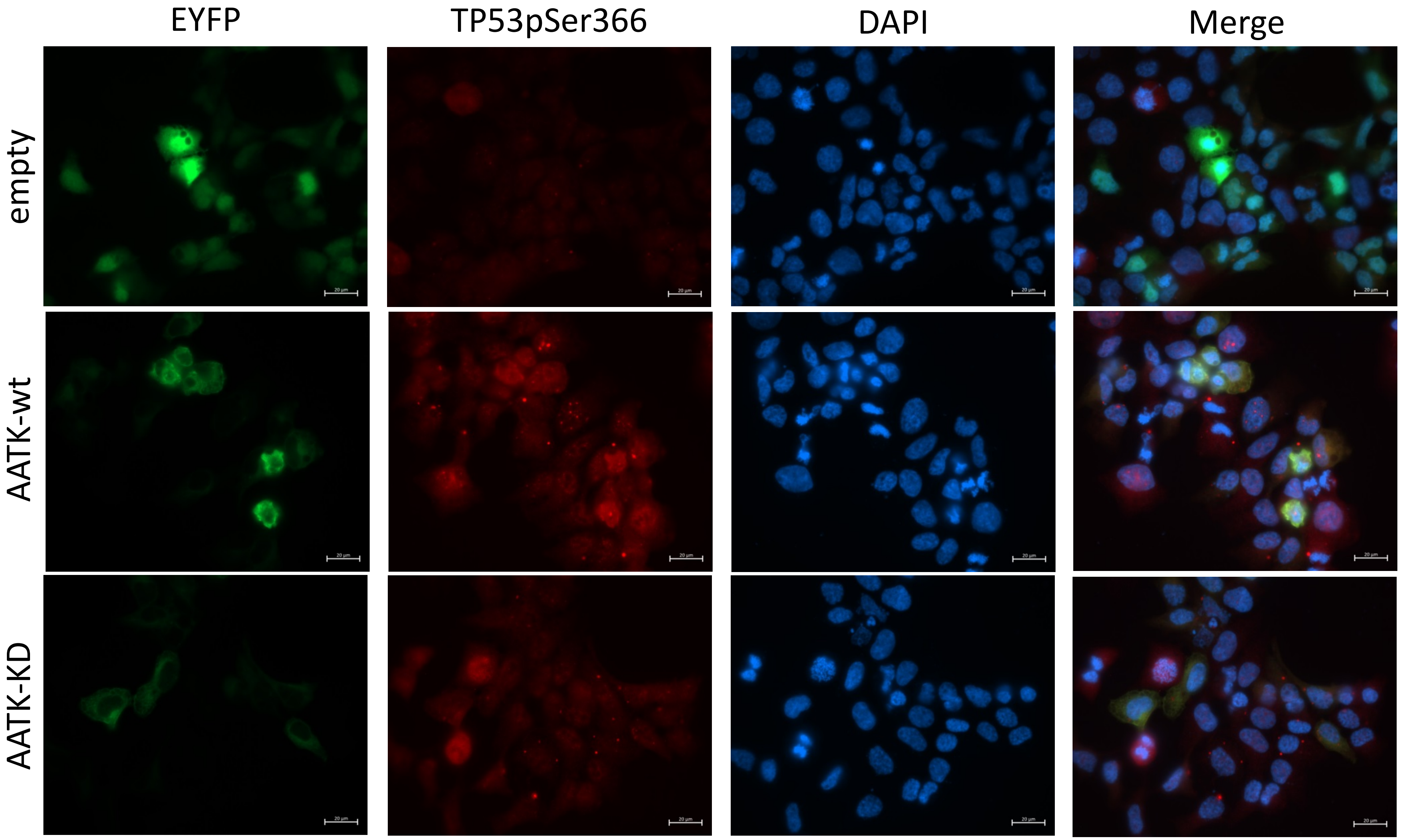

S15A: Exogenous AATK expression leads to an increase TP53Ser366 phosphorylation. HEK293T cells expressing EYFP, AATK wt or AATK KD exogenously for 24 h were fixed, stained for TP53Ser366 phosphorylation and counterstained DAPI. **A** Example panel for immunofluorescent staining. Scale bar = 20 µm.

B

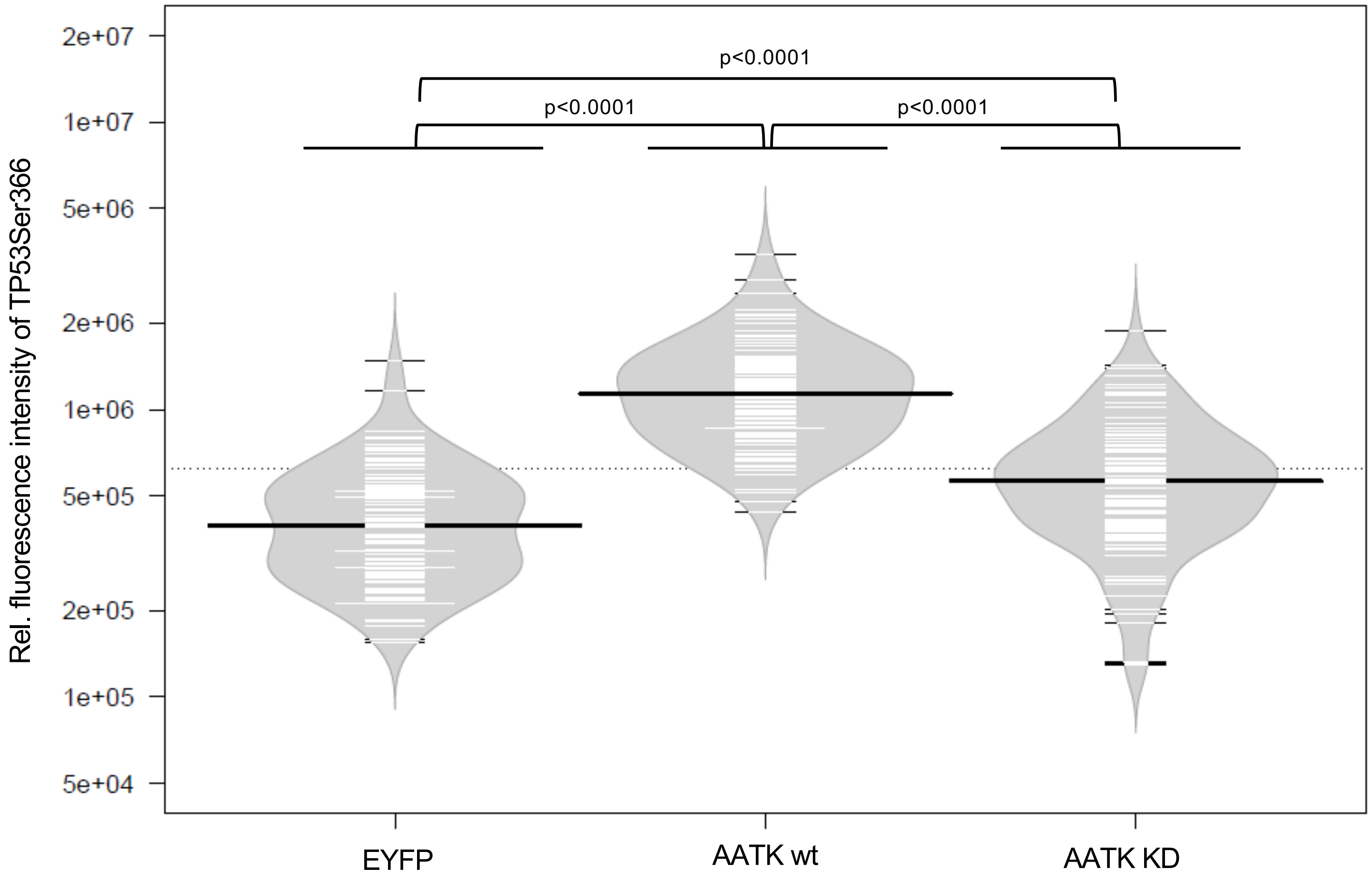

S15B: Exogenous AATK expression leads to an increase TP53Ser366 phosphorylation. HEK293T cells expressing EYFP, AATK wt or AATK KD exogenously for 24 h were fixed, stained for TP53Ser366 phosphorylation and counterstained DAPI. **B** Quantification of fluorescent intensity of TP53Ser366 phosphorylation in cells positive for expression of EYFP, AATK wt or AATK KD. Median CTCF (Integrated Density – (Area of selected cell X Mean fluorescence of background readings) representing fluorescence intensity (M. Fitzpatrick, 2014) of 115 cells, respectively; Black lines represent the medians, white lines individual data points and polygons the estimated density of the data, One-way ANOVA.

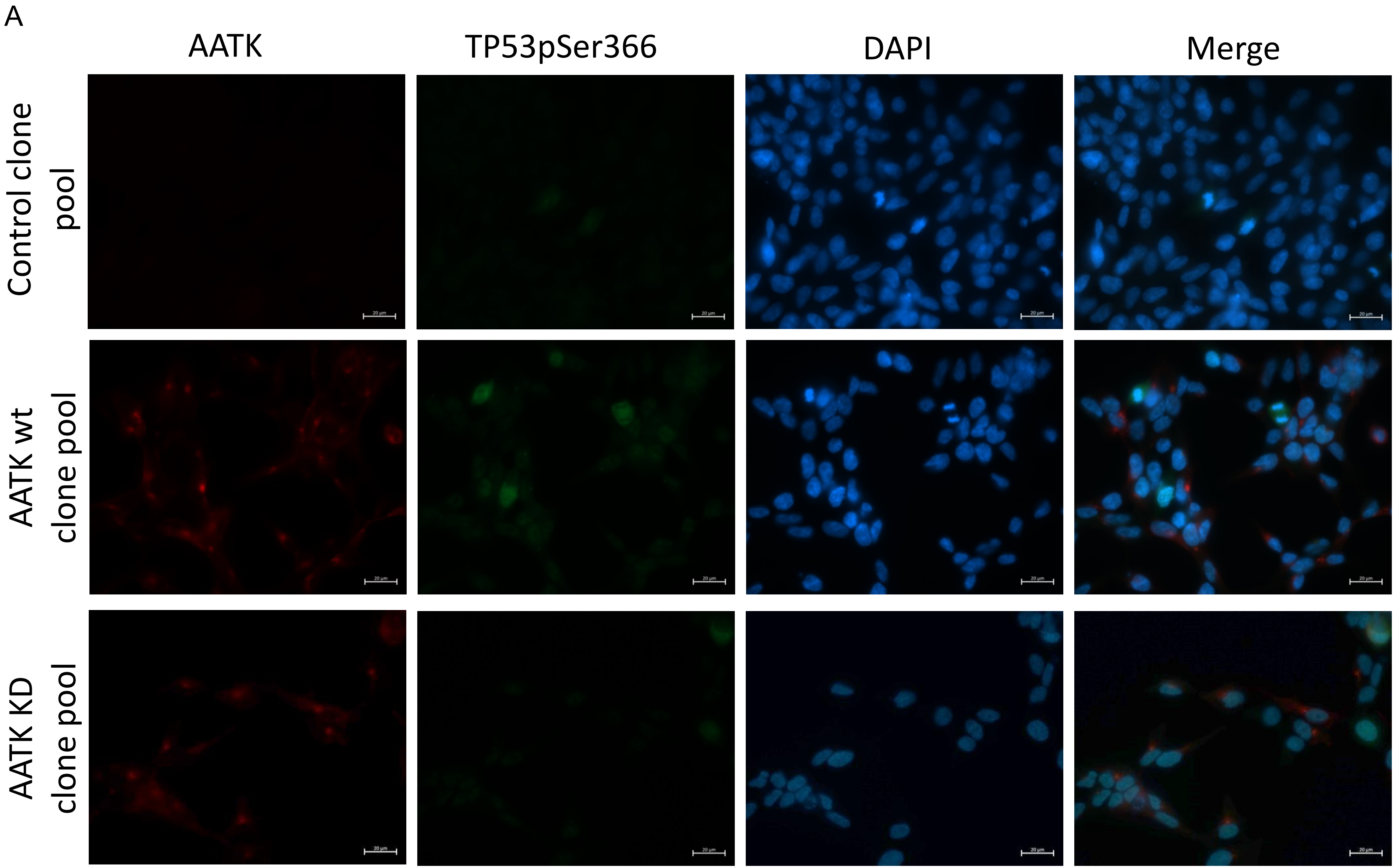

S16A: Exogenous AATK expression increases TP53 phosphorylation at Ser366. Inducibe clone pools for expression of AATK wt or AATK KD and a control clone pool were induced for 24 h, fixed, stained for AATK, TP53Ser366 phosphorylation and counterstained with DAPI. **A** Example panel of immunofluorescent staining. Scale bar = 20 µm.

B

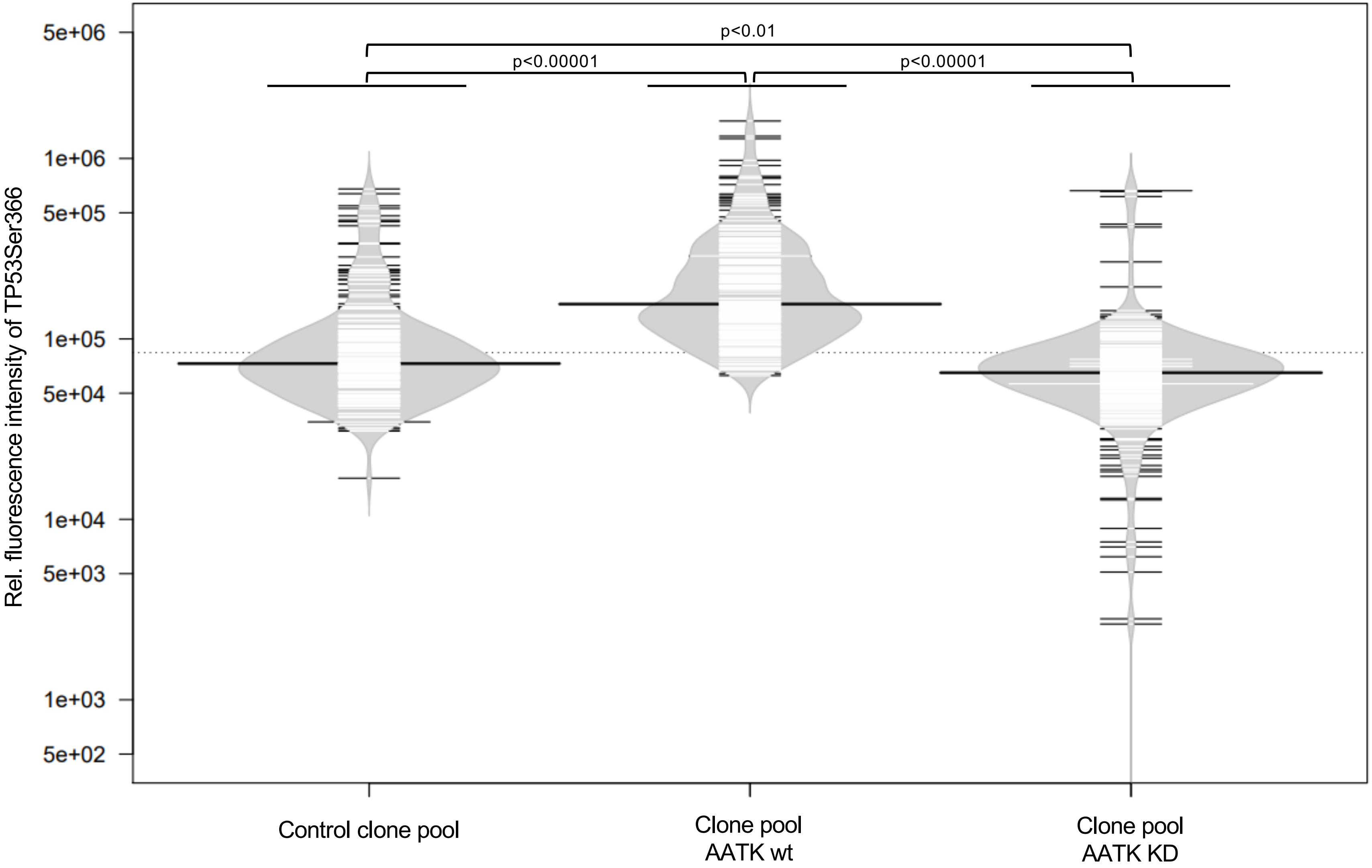

S16B: Exogenous AATK expression increases TP53 phosphorylation at Ser366. Inducibe clone pools for expression of AATK wt or AATK KD and a control clone pool were induced for 24 h, fixed, stained for AATK, TP53Ser366 phosphorylation and counterstained with DAPI. **B** Quantification of fluorescent intensity of TP53Ser366 phosphorylation in cells positive for expression of AATK wt or AATK KD. Median CTCF (Integrated Density – (Area of selected cell X Mean fluorescence of background readings) representing fluorescence intensity (M. Fitzpatrick, 2014) with n=198, n=219, n=257; Black lines represent the medians, white lines individual data points and polygons the estimated density of the data, One-way ANOVA.

.

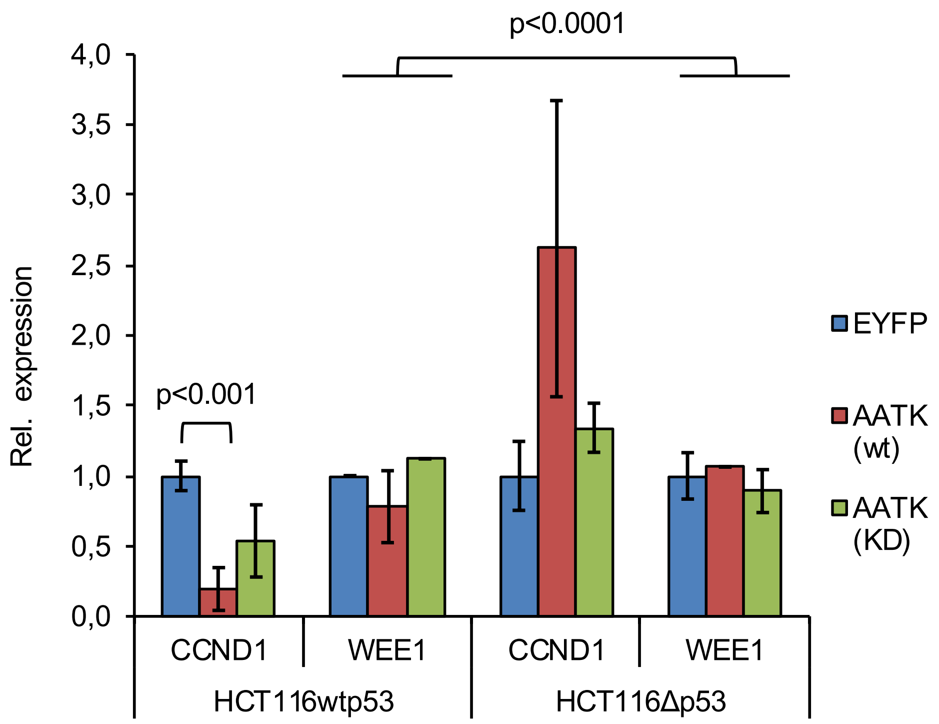

S17: TP53 is required for downregulation of *CCND1* expression via AATK wt. Relative *CCND1* and *WEE1* expression normalized to *β-ACTIN* in HCT116wtp53 and HCT116Δp53 (via CRISPR/Cas9) cells with exogeneous expression of AATK wt and KD or empty control for 24 h (displayed as mean of three replicates with SD, unpaired two-sided t-test). ANOVA of group HCT116wtp53 and HCT116Δp53.

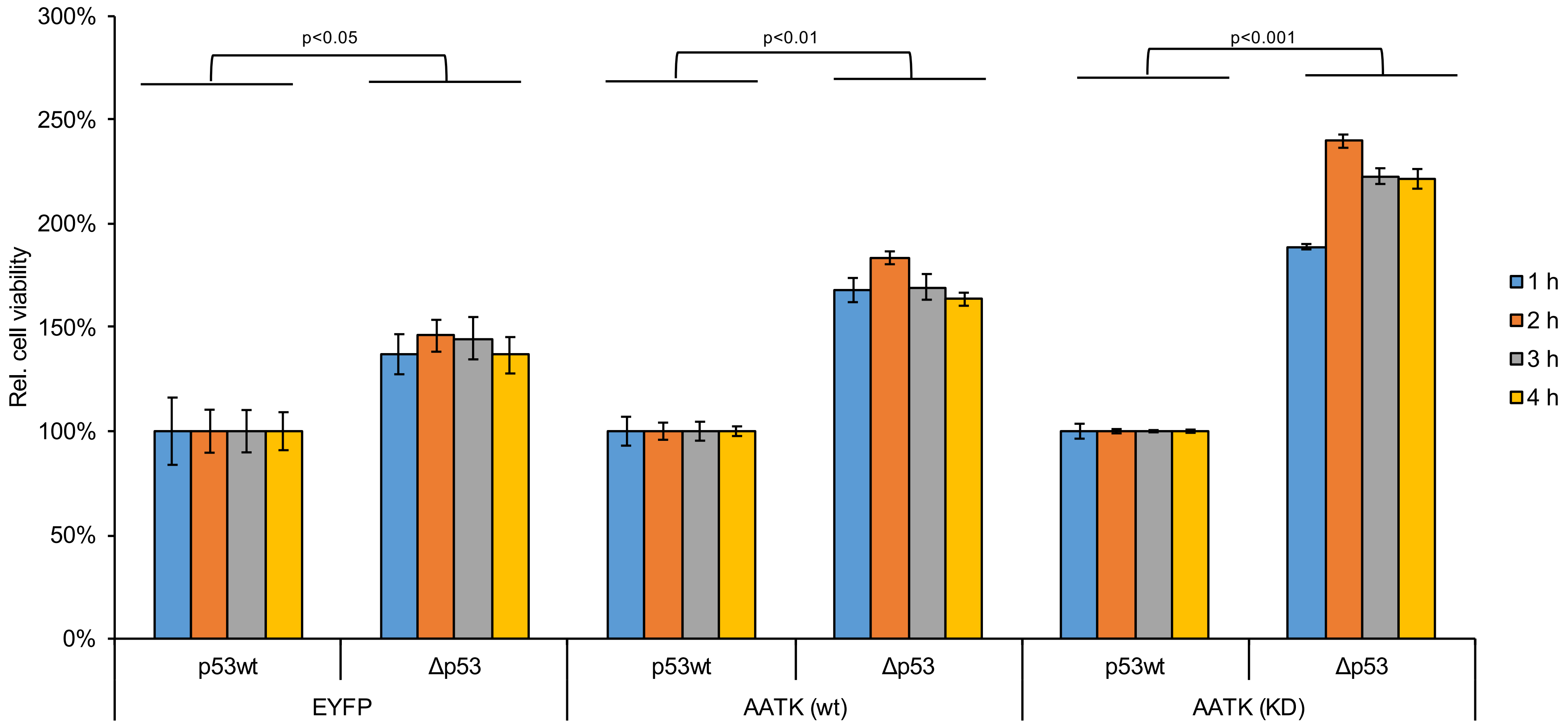

S18: Induced cell viability through loss of p53 in HCT116 cells is increased through AATK (wt and KD). Cell viability of HCT116wtp53 and HCT116Δp53 cells expressing AATK wt and KD or empty control for 24 h was determined via MTS assay at an absorbance of 490 nm with a reference at 650 nm (displayed as mean of three replicates with SD, ANOVA of group HCT116wtp53 and HCT116Δp53 respectively).

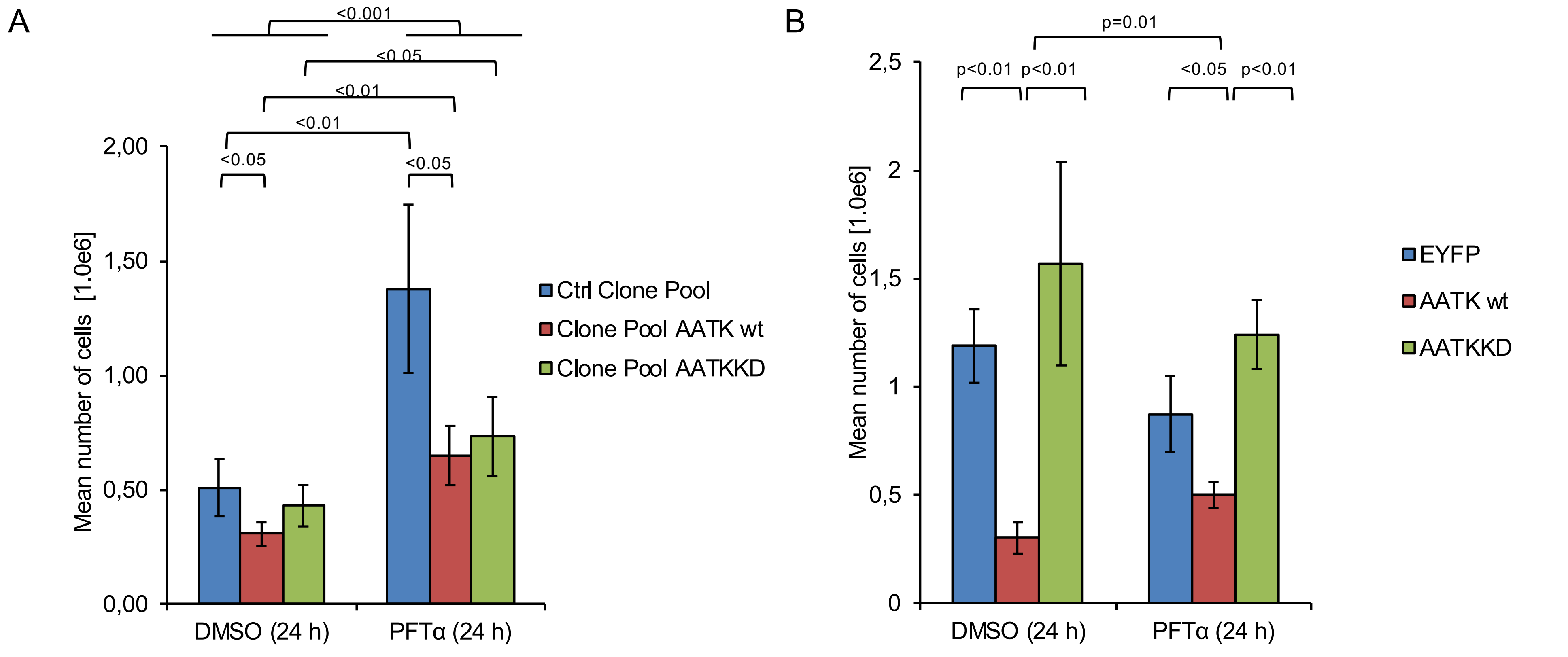

S19: Reduced cell growth via exogenous AATK expression is overcome by inhibiton of TP53. A Inducible clone pools expressing AATK wt or AATK KD in a doxycycline-dependent matter and a control clone pool or (**B**) MCF-7 expressing EYFP, AATK wt or AATK KD exogenously were seeded at 2.4e5 cells and grown for three days. 24 h prior to cell counting the cells were either treated with DMSO or 20 µM Pifithrinα (PFTα) (displayed as mean of three replicates with SD, unpaired one-sided t-test; One-way ANOVA for group DMSO vs. group PFTα).

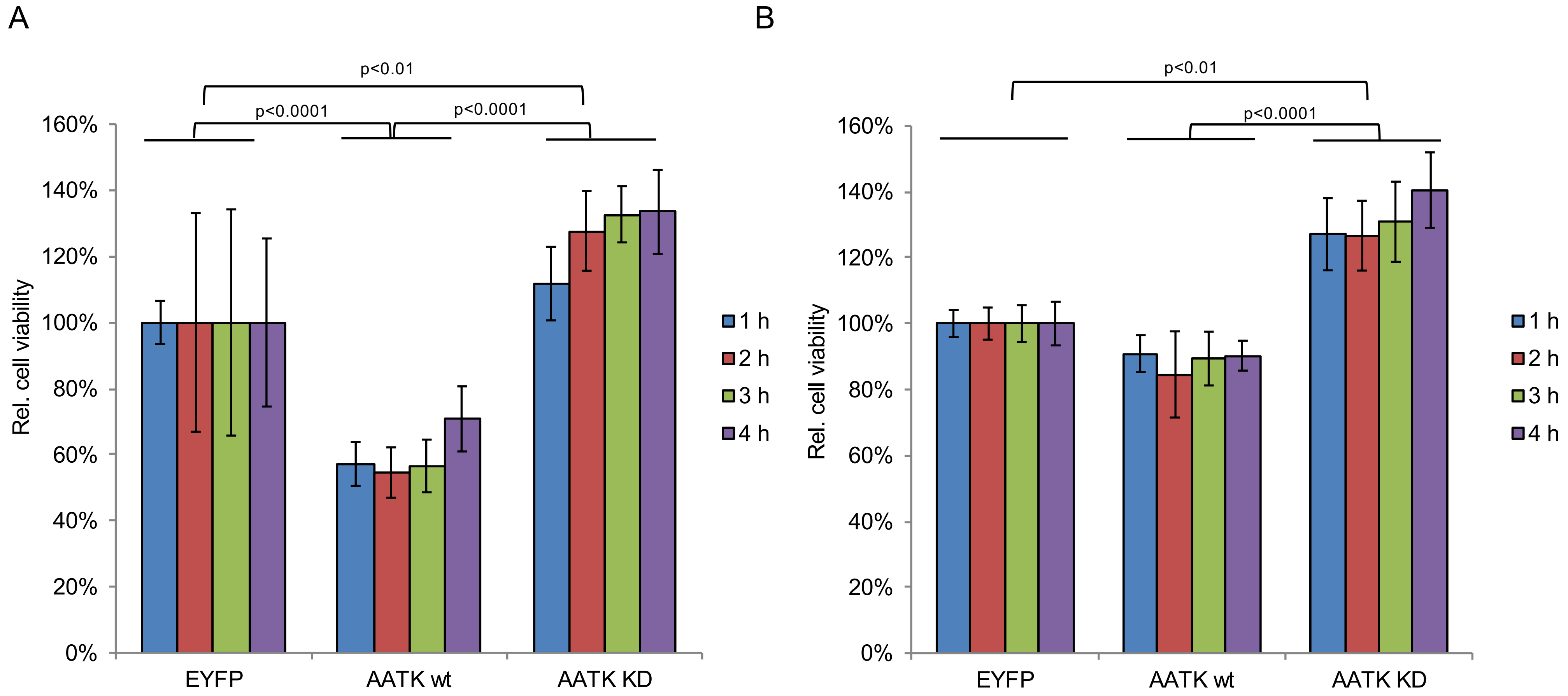

S20: Exogenous expression of AATK KD induces cell viability. MTS-assay of (**A**) HEK293 or (**B**) MCF-7 expressing EYFP, AATK-wt or AATK-KD exogenously for 24 h. Absorbance at 490 nm (reference at 650 nm) was measured every hour for four hours (displayed as mean of three replicates with SD, One-way ANOVA).
