# Supplementary material for: Epigenetically silenced apoptosis-associated tyrosine kinase (AATK) facilitates a decreased expression of Cyclin D1 and WEE1, phosphorylates TP53 and reduces cell proliferation in a kinase-dependent manner
Source: Cancer Gene Ther. 2022 Jul 28;29(12):1975–87. doi: 10.1038/s41417-022-00513-x (PMC9750878; doi:10.1038/s41417-022-00513-x)
Supplement: Supplementary file 3 — LC-MSMS Parametrization [file 41417_2022_513_MOESM3_ESM.pdf]

AAKT Interactomics (AMS114)

LC-MSMS Parametrization

This file is produced using R and the package openxlsx .

Tab Index

LC-MSMS Parameters:	LC-MS/MS parameters extracted from ThemeroFisher Scientific RAW files using (Kiweler, Looso, and Graumann, 2019).
LC Gradient:	Chromatography gradient(s) extracted from ThermoFisher Scientific RAW files using (Kiweler, Looso, and Graumann, 2019).
MaxQuant Parameters:	Parametrization of the MaxQuant suite of algorithms (Cox and Mann, 2008; Cox, Hein, Luber, Paron, Nagaraj, and ann, Matthias, 2014; Cox, Neuhauser, Michalski, Scheltema, Olsen, and Mann, 2011) as provided in the paramters.txt file.

Bibliography

[1] J. Cox, M. Y. Hein, C. A. Luber, et al. “Accurate Proteome-wide Label-free Quantification by Delayed Normalization and Maximal Peptide Ratio Extraction, Termed MaxLFQ”. In: _Molecular & Cellular Proteomics_ 13.9 (2014), pp. 2513-2526. DOI: 10.1074/mcp.M113.031591.

[2] J. Cox and M. Mann. “MaxQuant enables high peptide identification rates, individualized p.p.b.-range mass accuracies and proteome-wide protein quantification”. In: _Nature Biotechnology_ 26.12 (2008), pp. 1367-1372. DOI: 10.1038/nbt.1511.

[3] J. Cox, N. Neuhauser, A. Michalski, et al. “Andromeda: A Peptide Search Engine Integrated into the MaxQuant Environment”. In: _Journal of Proteome Research_ 10.4 (2011), pp. 1794-1805. DOI: 10.1021/pr101065j.

[4] M. Kiweler, M. Looso, and J. Graumann. “MARMoSET – Extracting Publication-Ready Mass Spectrometry Metadata from RAW Files”. In: _Molecular & Cellular Proteomics_ (Jan. 01, 2019), p. mcp.TIR119.001505. DOI: 10.1074/mcp.TIR119.001505.

Term	Value
High Performance Liquid Chromatography (HPLC) Instrument	Thermo EASY-nLC
HPLC Vendor	Thermo Scientific
Injected Sample Volume	4.00
Mass Spectrometer (MS)	Q Exactive HF - Orbitrap MS
MS Vendor	Thermo Scientific
MS Model	QExactive HF
MS Instrument Software	Xcalibur
MS Instrument Software Version	2.9-290033/2.9.0.2926
MS Ionization Type	Electrospray Ionization (ESI)
MS Electrospray Voltage	2000
MS Heated Capillary Temperature	310
MS Analyzer Precursor Ion Spectrum (MS1)	orbitrap
MS Data Type Precursor Ion Spectrum (MS1)	Profile
MS Micro Spectra Precursor Ion Spectrum (MS1)	1
MS Resolution Precursor Ion Spectrum (MS1)	60,000
MS Resolution m/z Precursor Ion Spectrum (MS1)	200 m/z
MS Mass Range Precursor Ion Spectrum (MS1)	300 to 1650 m/z
MS Ion Target Value Precursor Ion Spectrum (MS1)	3e6
MS Max. Injection Time Precursor Ion Spectrum (MS1)	15 ms
MS Analyzer Fragment Ion Spectrum (MS2)	orbitrap
MS Data Type Fragment Ion Spectrum (MS2)	Profile
MS Micro Spectra Fragment Ion Spectrum (MS2)	1
MS Fixed First m/z Fragment Ion Spectrum (MS2)	-
MS Isolation Window Width Fragment Ion Spectrum (MS2)	1.2 m/z
MS Isolation Window Offset Fragment Ion Spectrum (MS2)	0.0 m/z
MS Fragmentation Mode (MS2)	HCD
MS Normalized Collision Energy Fragment Ion Spectrum (MS2)	nce: 28
MS Resolution Fragment Ion Spectrum (MS2)	15,000
MS Resolution m/z Fragment Ion Spectrum (MS2)	200 m/z
MS Ion Target Value Fragment Ion Spectrum (MS2)	5e5
MS Max. Injection Time Fragment Ion Spectrum (MS2)	15 ms
MS Loop Count (Top n) Fragment Ion Spectrum (MS2)	20
MS Charge States Excluded from Fragmentation	unassigned, 1, >8
MS Minimum Peak Intensity for Fragmentation	1.0e5
MS Fragmentation Trigger on Peak Apex	-
MS Dynamic Exclusion Length	20.0 s
MS Isotopologue Exclusion	on
MS Peptide Model for Excluded Isotope Clusters	preferred

Time[mm:ss]	Duration[mm:ss]	Flow[nl/min]	Mixture[%B]
00:00	00:00	400	5
215:00	215:00	400	30
220:00	05:00	400	60
225:00	05:00	400	95
230:00	05:00	400	95
235:00	05:00	400	5
240:00	05:00	400	5

Parameter	Value
Version	1.6.5.0
User name	asokol
Machine name	KI-S0114
Date of writing	05/21/2019 13:05:55
Include contaminants	True
PSM FDR	0.01
PSM FDR Crosslink	0.01
Protein FDR	0.01
Site FDR	0.01
Use Normalized Ratios For Occupancy	True
Min. peptide Length	7
Min. score for unmodified peptides	0
Min. score for modified peptides	40
Min. delta score for unmodified peptides	0
Min. delta score for modified peptides	6
Min. unique peptides	0
Min. razor peptides	1
Min. peptides	1
Use only unmodified peptides and	True
Modifications included in protein quantification	Oxidation (M);Acetyl (Protein N-term)
Peptides used for protein quantification	Razor
Discard unmodified counterpart peptides	True
Label min. ratio count	2
Use delta score	False
iBAQ	False
iBAQ log fit	False
Match between runs	True
Matching time window [min]	0.7
Match ion mobility window [indices]	0.05
Alignment time window [min]	20
Alignment ion mobility window [indices]	1
Find dependent peptides	False
Fasta file	\\ki-s0147.kerckhoff.mpg.de\AG-Proteomics$\Fasta 2019\20190123\Human_9606_canonical+isoforms
Decoy mode	revert
Include contaminants	True
Advanced ratios	True
Fixed andromeda index folder	
Temporary folder	
Combined folder location	
Second peptides	True
Stabilize large LFQ ratios	True
Separate LFQ in parameter groups	False
Require MS/MS for LFQ comparisons	True
Calculate peak properties	False
Main search max. combinations	200
Advanced site intensities	True

Write msScans table	False
Write msmsScans table	True
Write ms3Scans table	True
Write allPeptides table	True
Write mzRange table	True
Write pasefMsmsScans table	True
Write accumulatedPasefMsmsScans table	True
Max. peptide mass [Da]	4600
Min. peptide length for unspecific search	8
Max. peptide length for unspecific search	25
Razor protein FDR	True
Disable MD5	False
Max mods in site table	3
Match unidentified features	False
Epsilon score for mutations	
Evaluate variant peptides separately	True
Variation mode	None
MS/MS tol. (FTMS)	20 ppm
Top MS/MS peaks per Da interval. (FTMS)	12
Da interval. (FTMS)	100
MS/MS deisotoping (FTMS)	True
MS/MS deisotoping tolerance (FTMS)	7
MS/MS deisotoping tolerance unit (FTMS)	ppm
MS/MS higher charges (FTMS)	True
MS/MS water loss (FTMS)	True
MS/MS ammonia loss (FTMS)	True
MS/MS dependent losses (FTMS)	True
MS/MS recalibration (FTMS)	False
MS/MS tol. (ITMS)	0.5 Da
Top MS/MS peaks per Da interval. (ITMS)	8
Da interval. (ITMS)	100
MS/MS deisotoping (ITMS)	False
MS/MS deisotoping tolerance (ITMS)	0.15
MS/MS deisotoping tolerance unit (ITMS)	Da
MS/MS higher charges (ITMS)	True
MS/MS water loss (ITMS)	True
MS/MS ammonia loss (ITMS)	True
MS/MS dependent losses (ITMS)	True
MS/MS recalibration (ITMS)	False
MS/MS tol. (TOF)	40 ppm
Top MS/MS peaks per Da interval. (TOF)	10
Da interval. (TOF)	100
MS/MS deisotoping (TOF)	True
MS/MS deisotoping tolerance (TOF)	0.01
MS/MS deisotoping tolerance unit (TOF)	Da
MS/MS higher charges (TOF)	True
MS/MS water loss (TOF)	True
MS/MS ammonia loss (TOF)	True

MS/MS dependent losses (TOF)	True
MS/MS recalibration (TOF)	False
MS/MS tol. (Unknown)	0.5 Da
Top MS/MS peaks per Da interval. (Unknown)	8
Da interval. (Unknown)	100
MS/MS deisotoping (Unknown)	False
MS/MS deisotoping tolerance (Unknown)	0.15
MS/MS deisotoping tolerance unit (Unknown)	Da
MS/MS higher charges (Unknown)	True
MS/MS water loss (Unknown)	True
MS/MS ammonia loss (Unknown)	True
MS/MS dependent losses (Unknown)	True
MS/MS recalibration (Unknown)	False
Site tables	Oxidation (M)Sites.txt
