# Supplementary material for: Epigenetically silenced apoptosis-associated tyrosine kinase (AATK) facilitates a decreased expression of Cyclin D1 and WEE1, phosphorylates TP53 and reduces cell proliferation in a kinase-dependent manner
Source: Cancer Gene Ther. 2022 Jul 28;29(12):1975–87. doi: 10.1038/s41417-022-00513-x (PMC9750878; doi:10.1038/s41417-022-00513-x)

Blot 5C AATK TP53

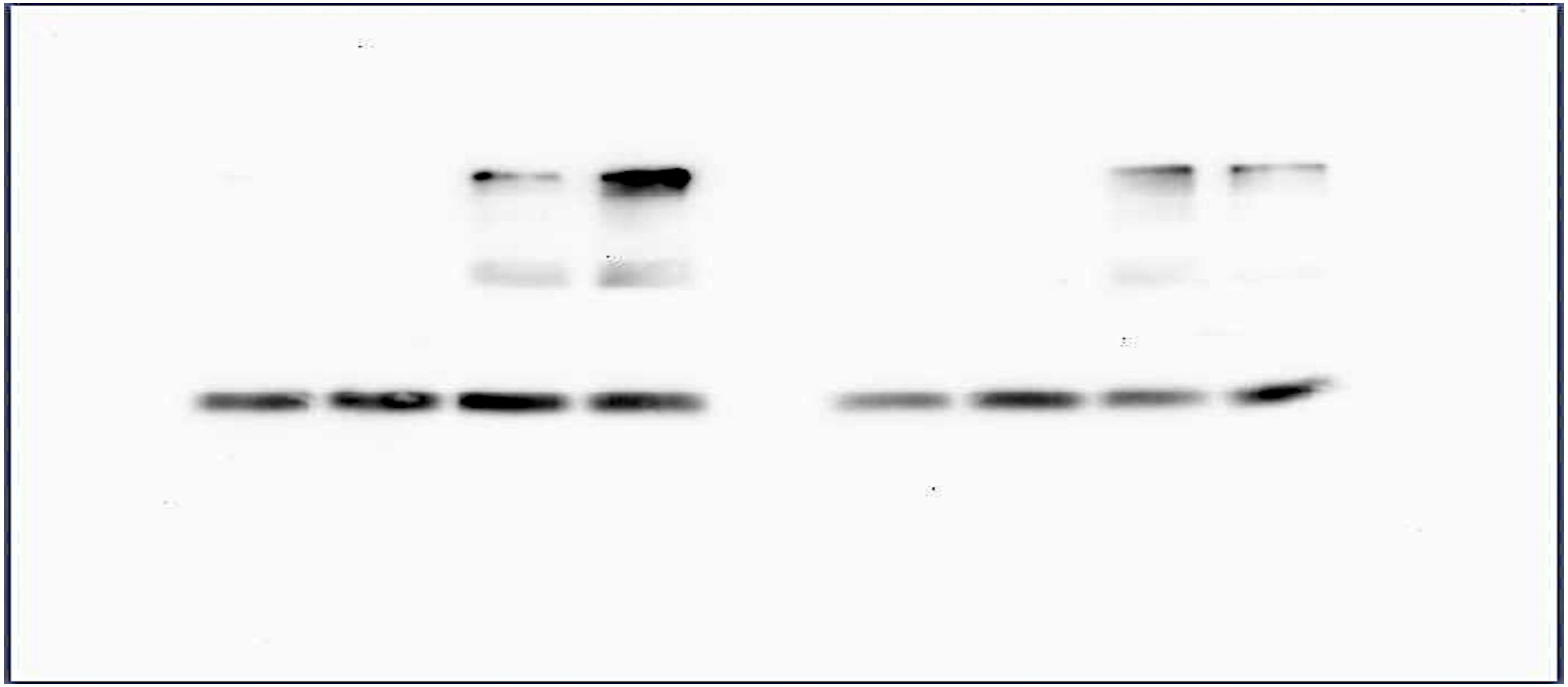

Blot 5C

pTP53 Ser366

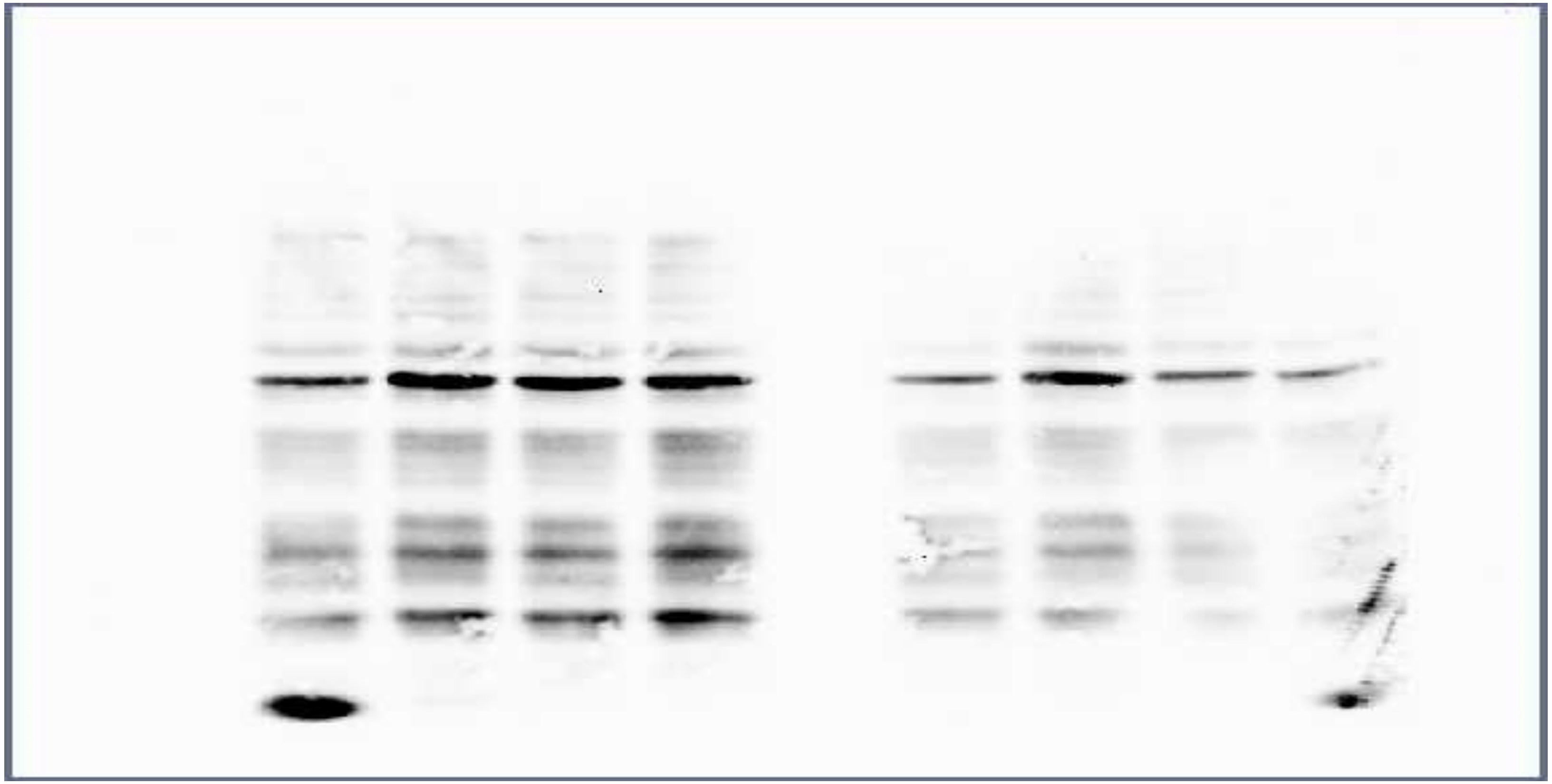

Blot 5C αTubulin

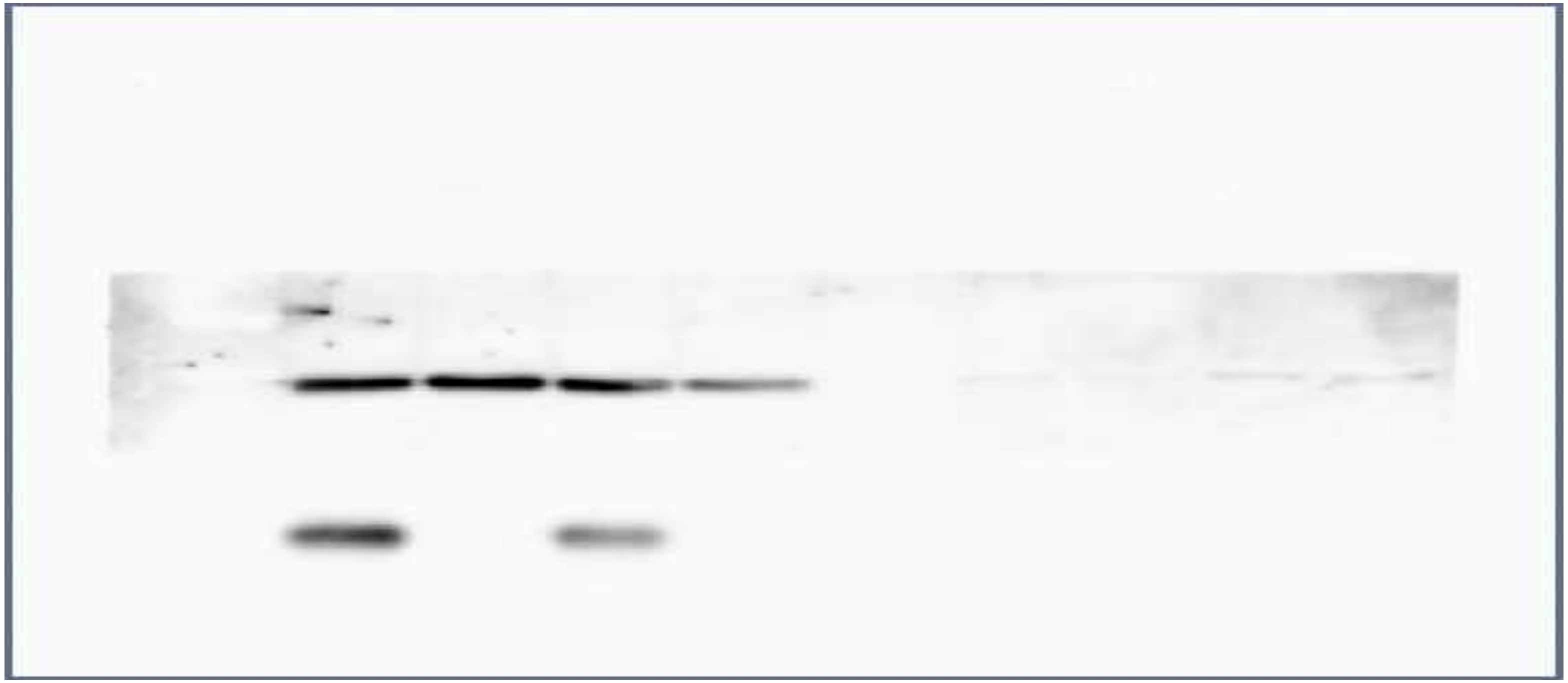

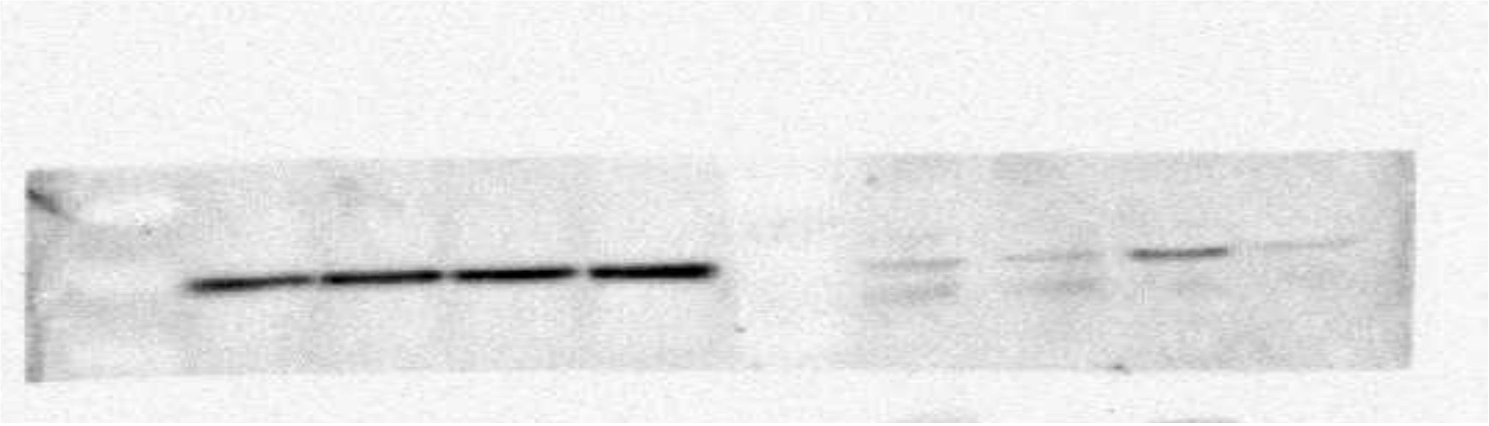

Blot 5C GFP

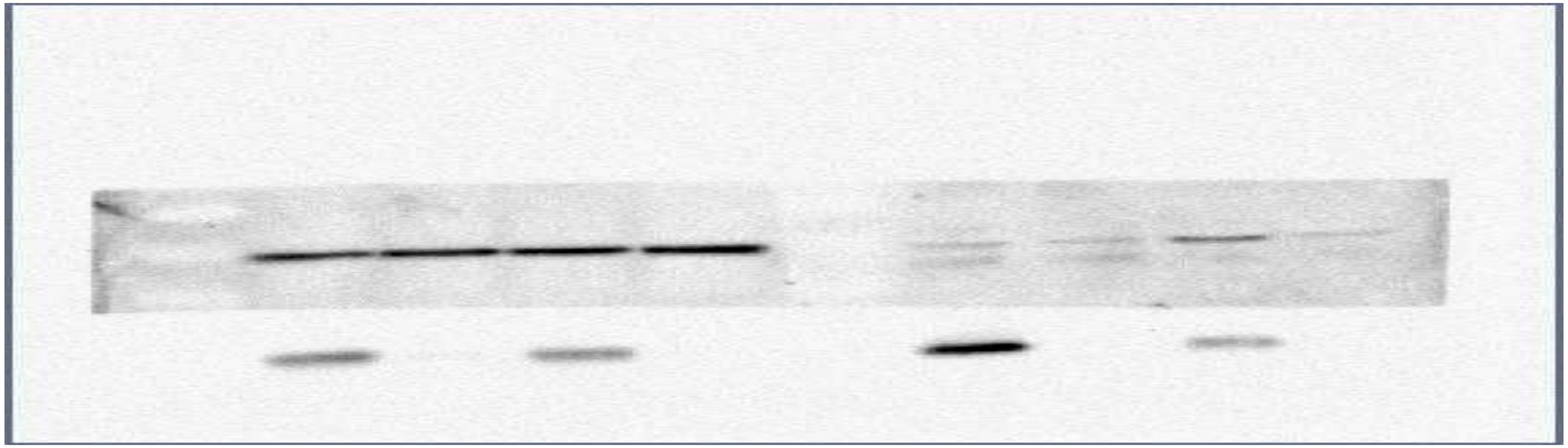

Blot 5D Input

AATK

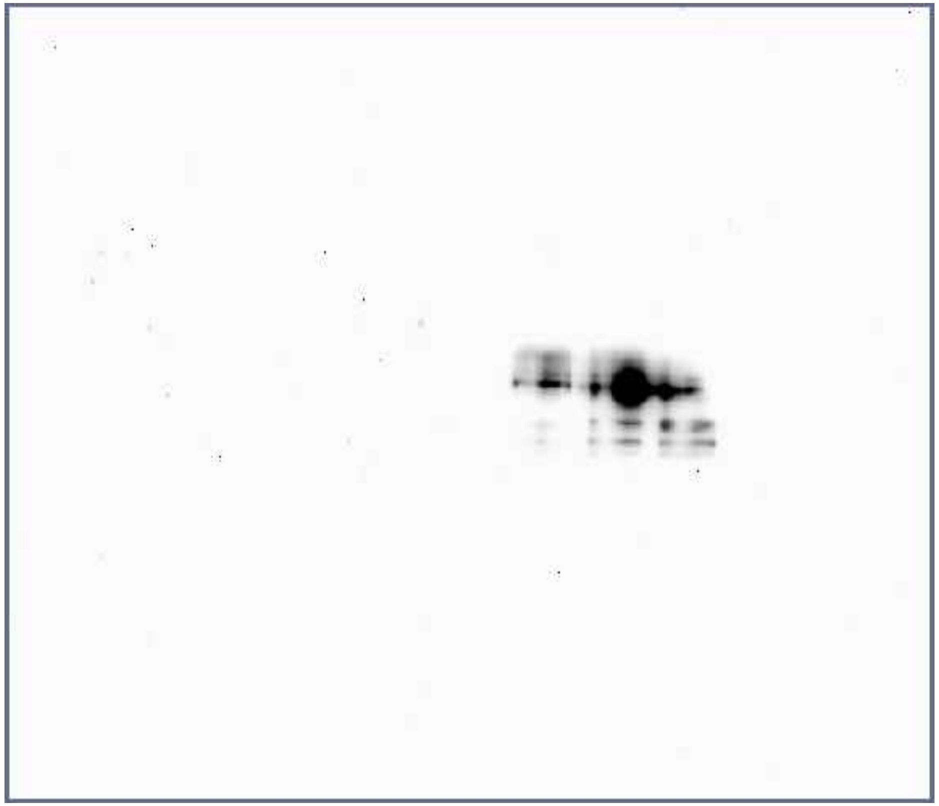

αTubulin

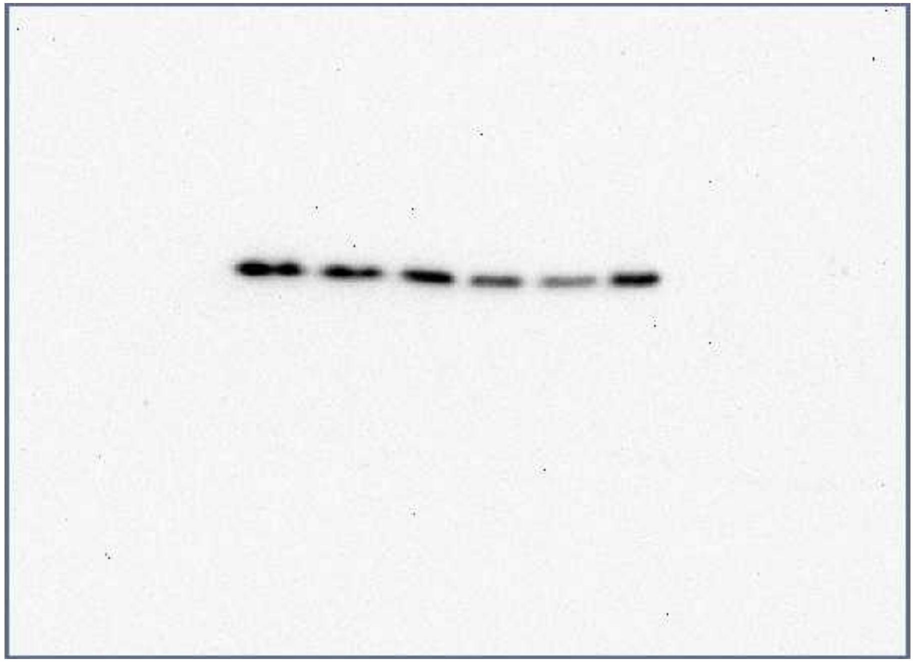

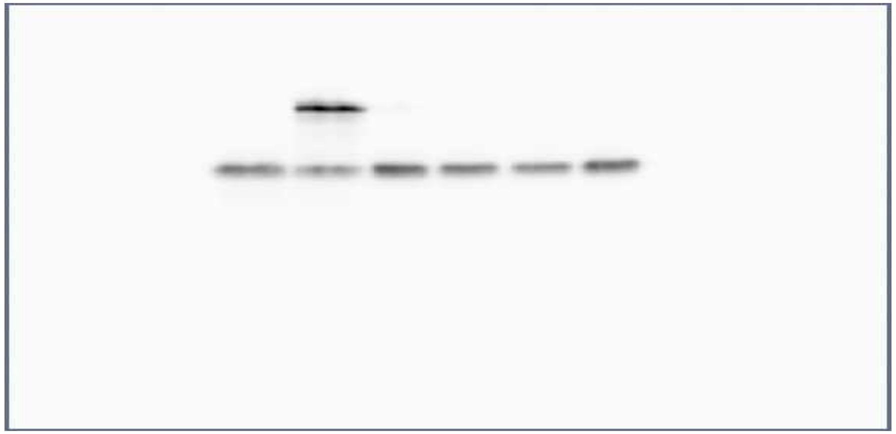

TP53-GFP

TP53

Blot 5D in vitro kinase assay

AATK

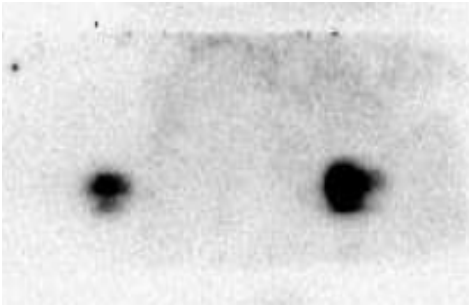

pTP53-Ser366

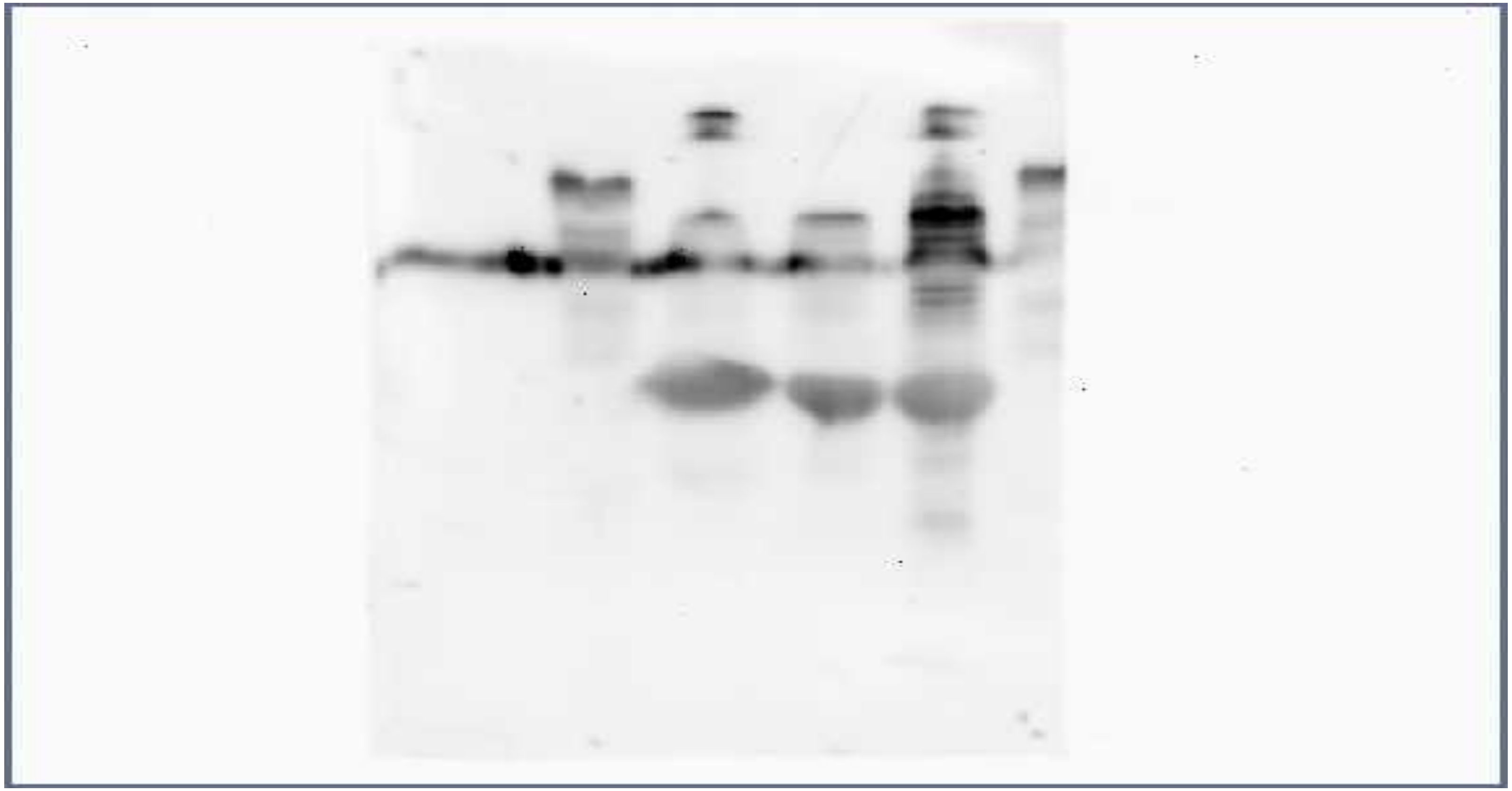

Blot 5D in vitro kinase assay

TP53

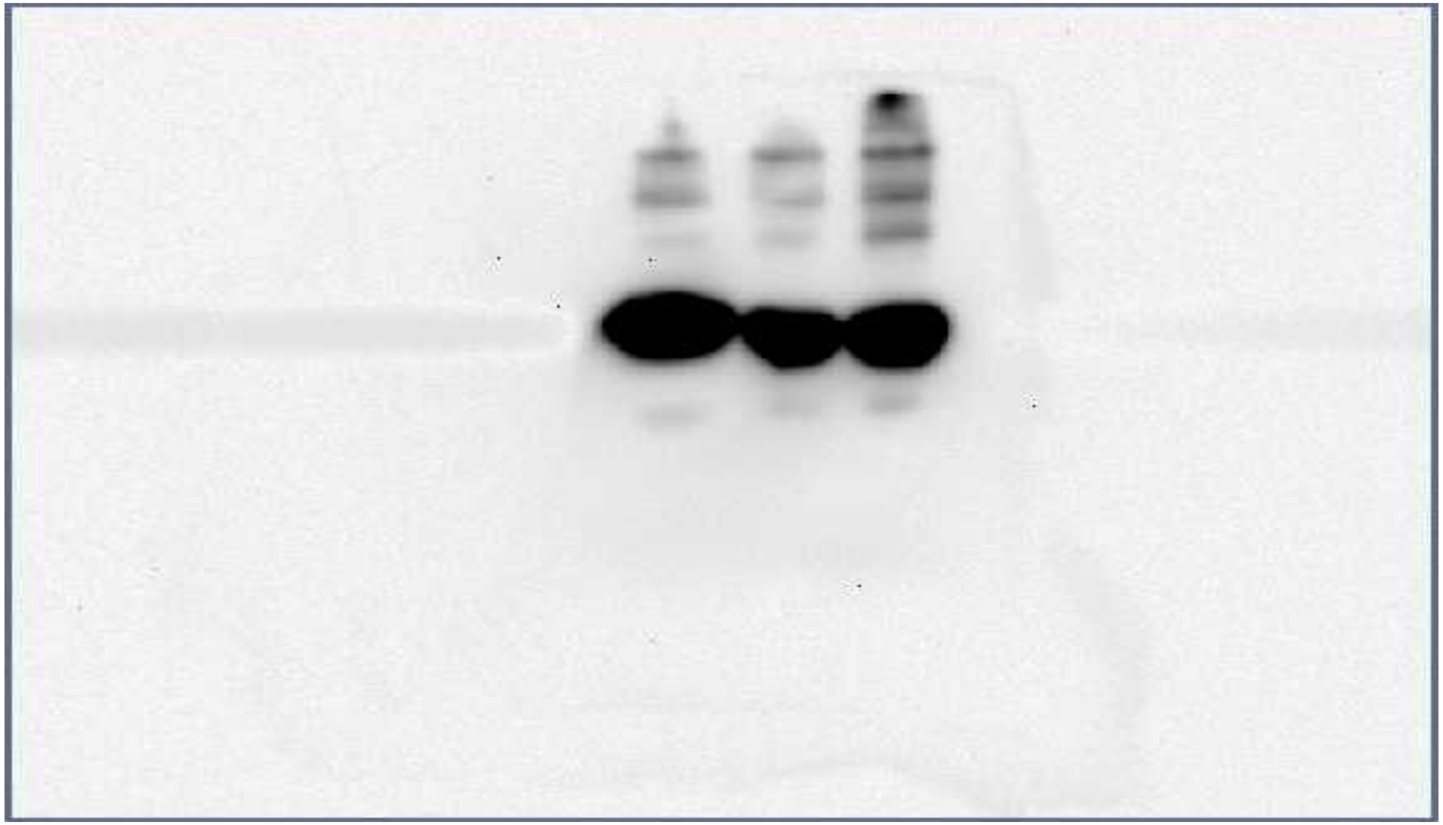

Supplement: Supplementary file 4 — Dataset original Western blots [file 41417_2022_513_MOESM4_ESM.pdf]
