# Supplementary material for: Epigenetically silenced apoptosis-associated tyrosine kinase (AATK) facilitates a decreased expression of Cyclin D1 and WEE1, phosphorylates TP53 and reduces cell proliferation in a kinase-dependent manner
Source: Cancer Gene Ther. 2022 Jul 28;29(12):1975–87. doi: 10.1038/s41417-022-00513-x (PMC9750878; doi:10.1038/s41417-022-00513-x)

File: 1+3.ab1

Geospiza
www.geospiza.com

Sample Name: 24 3_AATKgDNAF1
Mobility: KB_3730_POP7_BDTv3.mob
Spacing: 14.3174
Comment: 42422218

Signal Strengths: A = 552, C = 1157, G = 962, T = 302
Lane/Cap#: 32
Matrix: n/a
Direction: Native

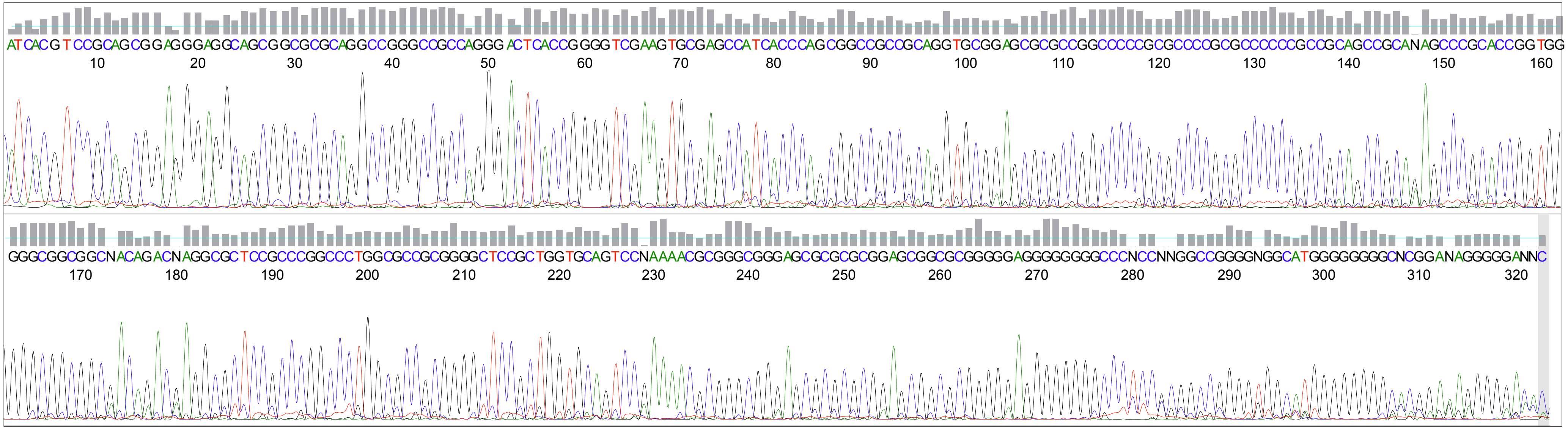

File: 1+4.ab1

Sample Name:	48 1_AATKgDNAF1	Signal Strengths:	A = 565, C = 1270, G = 1056, T = 284
Mobility:	KB_3730_POP7_BDTv3.mob	Lane/Cap#:	22
Spacing:	14.4785	Matrix:	n/a
Comment:	42422221	Direction:	Native

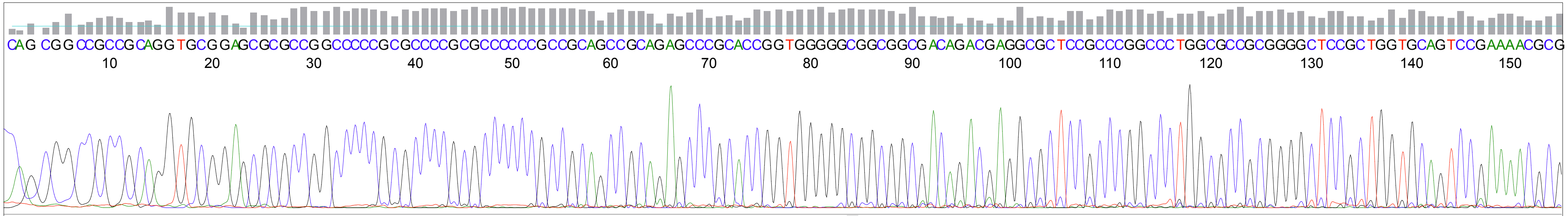

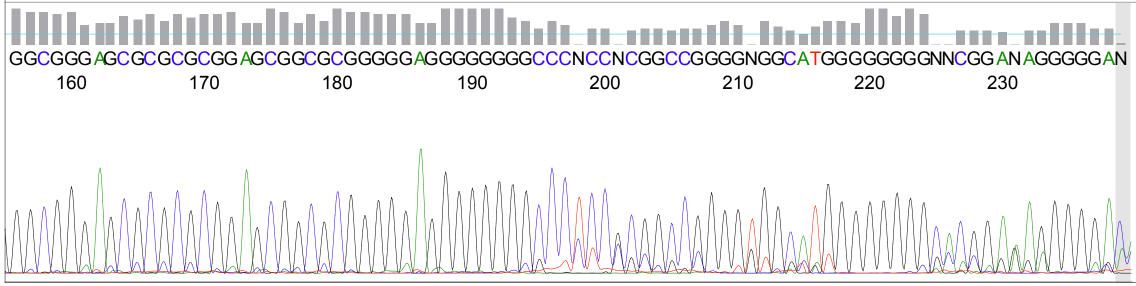

File: 2+3.ab1

Sample Name: 24 5_AATKgDNAF1
Mobility: KB_3730_POP7_BDTv3.mob
Spacing: 14.3699
Comment: 42422220

Signal Strengths: A = 418, C = 823, G = 699, T = 236
Lane/Cap#: 45
Matrix: n/a
Direction: Native

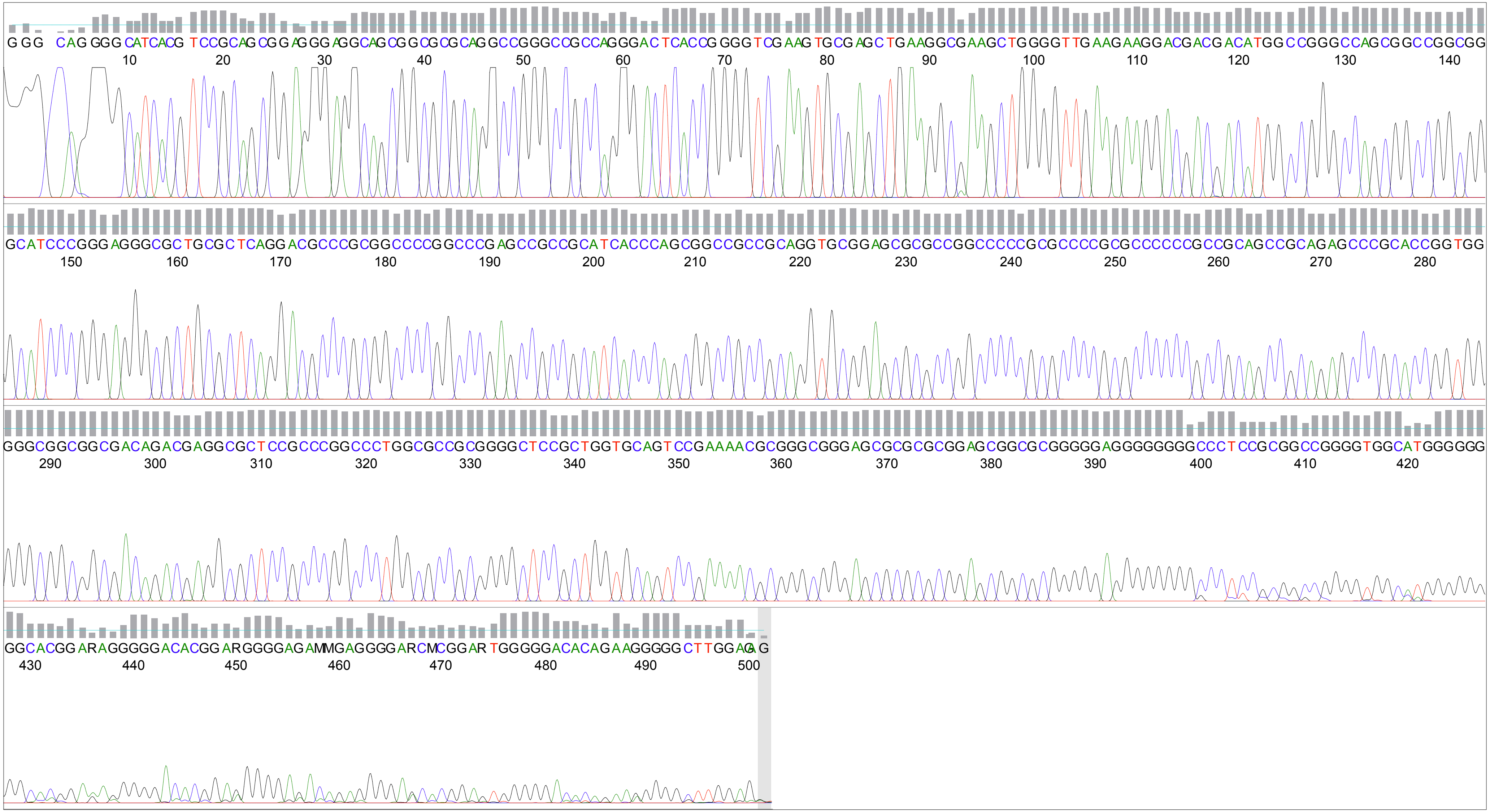

File: 2+4.ab1

Geospiza
www.geospiza.com

Sample Name:	48 3_AATKgDNAF1	Signal Strengths:	A = 1646, C = 3704, G = 2891, T = 884
Mobility:	KB_3730_POP7_BDTv3.mob	Lane/Cap#:	30
Spacing:	14.2645	Matrix:	n/a
Comment:	42422223	Direction:	Native

GCATCACGTCCGCAGCGGAGGGAGGCAGCGGCGCGCAGGCCGGGCCGCCAGGGACTCACCGGGGTCGAAGTGCGAGCCATCACCCAGCGGCCGCCGCAGGTGCGGAGCGCGCCGGCCCCCGCGCCCCGCGCCCCCCGCCGCAGCCGCANAGC

10 20 30 40 50 60 70 80 90 100 110 120 130 140 150

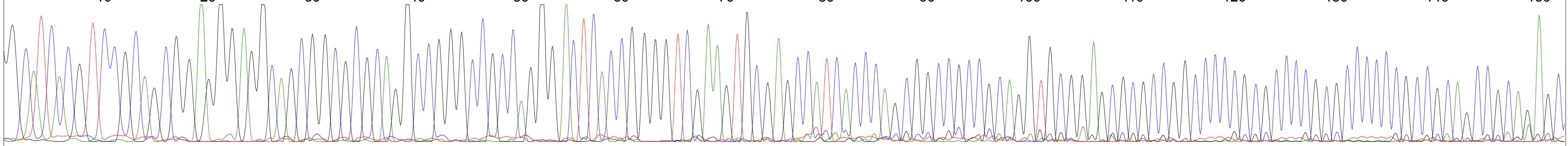

CCGCACCGGTGGGGGCGGCGGCGACAGACNAGGCGCTCCGCCCGGCCCTGGCGCCGCGGGGCTCCGCTGGTGCAGTCCRAAAACGCGGGCGGGAGCGCGCGCGGAGCGGCGCGGGGAGGGGGGGCCCNCCNNGGCCGGGGNGGCATGGGGG

160 170 180 190 200 210 220 230 240 250 260 270 280 290 300

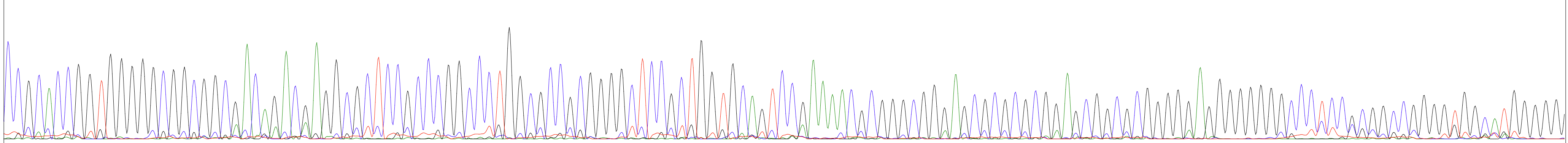

GGGCNCGGANAGGGGGACNCGGAGGGGGANACANAGGGGANCNCGGAGTGGGGGACNCANAAGGGGGCTTGGAG

310 320 330 340 350 360 370

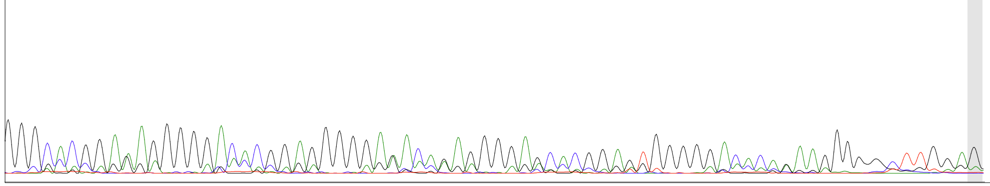

File: w:o.ab1

Geospiza
www.geospiza.com

Sample Name: 24 1_AATKgDNAF1
Mobility: KB_3730_POP7_BDTv3.mob
Spacing: 14.3239
Comment: 42422216

Signal Strengths: A = 230, C = 494, G = 417, T = 135
Lane/Cap#: 43
Matrix: n/a
Direction: Native

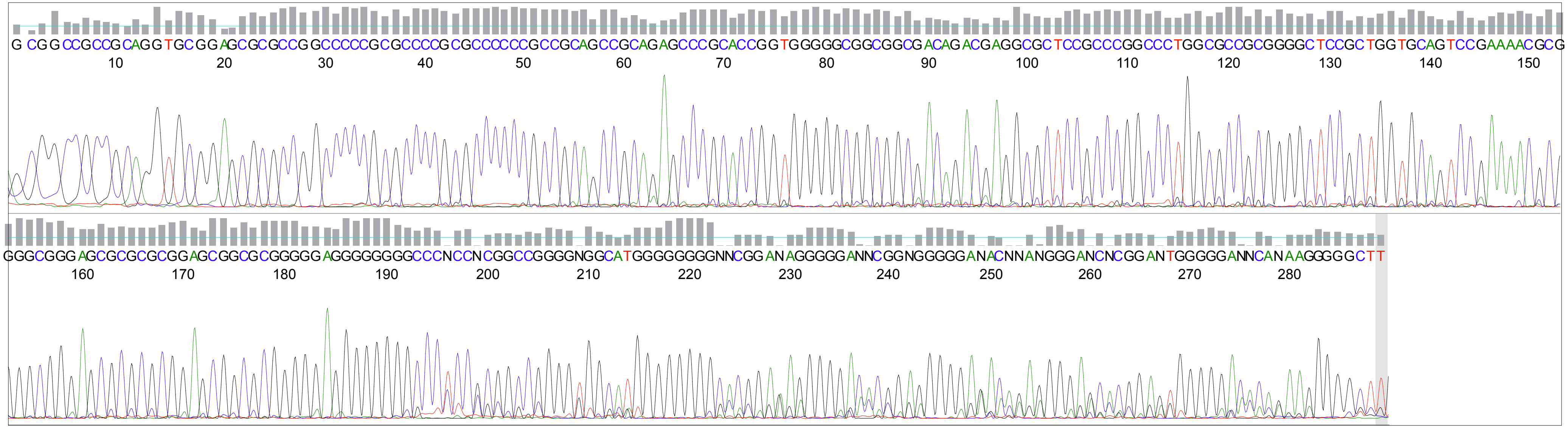

Supplement: Supplementary file 5 — Dataset Sanger sequences [file 41417_2022_513_MOESM5_ESM.pdf]
