# Supplementary material for: Epigenetically silenced apoptosis-associated tyrosine kinase (AATK) facilitates a decreased expression of Cyclin D1 and WEE1, phosphorylates TP53 and reduces cell proliferation in a kinase-dependent manner
Source: Cancer Gene Ther. 2022 Jul 28;29(12):1975–87. doi: 10.1038/s41417-022-00513-x (PMC9750878; doi:10.1038/s41417-022-00513-x)
Supplement: Supplementary file 6 — Dataset original qPCR [file 41417_2022_513_MOESM6_ESM.zip › HEK_ACTB.pdf]

14 Hilly Street Mortlake NSW 2137 Australia
T +61 2 9736 1320
F +61 2 9736 1364
W www.corbettlifescience.com

Comparative Quantitation Report

Experiment Information

Run Name	Run 2020-06-07_b-Act_RNAi HEK (2)_(3);UV HEK (1)_(2)
Run Start	07.06.2020 13:11:27
Run Finish	07.06.2020 14:45:28
Operator	MW
Notes	b-Act RNAi HEK (2) (3), UV HEK (1) (2) triplicate
Run On Software Version	Rotor-Gene 6.1.93
Run Signature	The Run Signature is valid.
Gain FAM	8.
Gain ROX	9.33

Comparative Quantitation Information

Reaction Amplification	1.66
Reaction Amplification Std. Deviation	0.02
Sample Page	Page 1
Control Replicate	(37)

Take off Graph for Cycling A.FAM/Cycling A.ROX

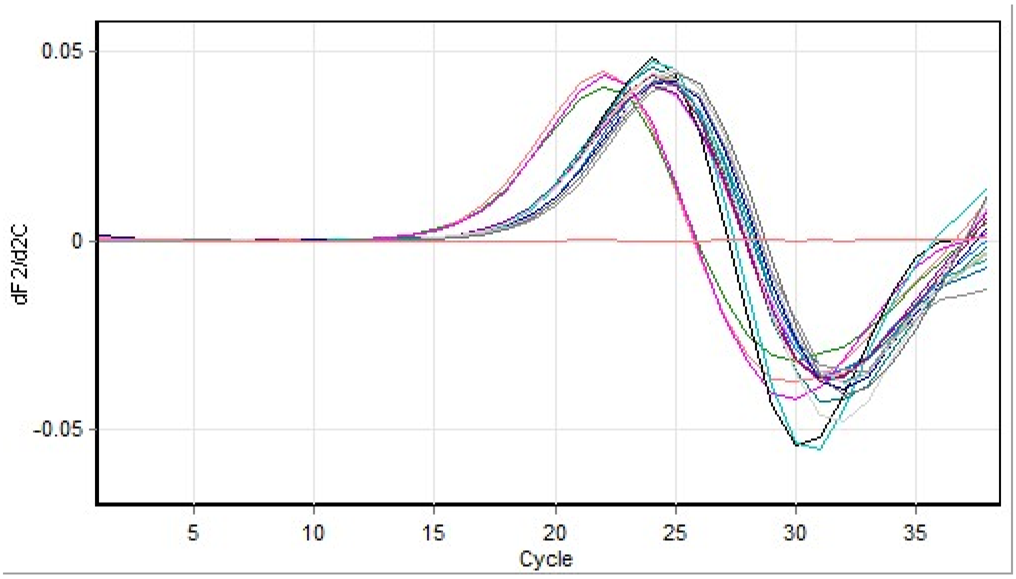

No.	Colour	Name	Take Off	Amplification	Comparative Conc.	Rep. Takeoff	Rep. Takeoff (95% CI)
E5		ohne EY (2)	19.6	1.65	1.03E+00	19.7	[1.$,1.$]
E6		ohne EY (2)	19.7	1.65	9.83E-01		
E7		ohne EY (2)	19.7	1.66	9.83E-01		
E8		ohne B (2)	17.5	1.67	2.99E+00	17.6	[1.$,1.$]
F1		ohne B (2)	17.5	1.67	2.99E+00		
F2		ohne B (2)	17.8	1.68	2.57E+00		
F3		ohne B KD (2)	20.0	1.65	8.45E-01	19.9	[1.$,1.$]
F4		ohne B KD (2)	20.0	1.64	8.45E-01		
F5		ohne B KD (2)	19.8	1.65	9.35E-01		
G7		ohne EY (3)	20.1	1.63	8.03E-01	20.1	[1.$,1.$]
G8		ohne EY (3)	20.0	1.69	8.45E-01		
H1		ohne EY (3)	20.1	1.64	8.03E-01		
H2		ohne B (3)	19.6	1.63	1.03E+00	19.7	[1.$,1.$]
H3		ohne B (3)	19.6	1.70	1.03E+00		
H4		ohne B (3)	19.8	1.66	9.35E-01		
H5		ohne B KD (3)	20.3	1.65	7.26E-01	20.4	[1.$,1.$]
H6		ohne B KD (3)	20.4	1.66	6.90E-01		
H7		ohne B KD (3)	20.4	1.65	6.90E-01		
I8		H2O	33.4	0.00	9.67E-04	33.4	

Quality System
Quality Endorsed Company
ISO 9001 Lic 21313
SAI Global

™ This report generated by Rotor-Gene Real-Time Analysis Software 6.1 (Build 93)
© Corbett Research 2005
® All Rights Reserved
ISO 9001:2000 (Reg. No. QEC21313)
