# Supplementary material for: Epigenetically silenced apoptosis-associated tyrosine kinase (AATK) facilitates a decreased expression of Cyclin D1 and WEE1, phosphorylates TP53 and reduces cell proliferation in a kinase-dependent manner
Source: Cancer Gene Ther. 2022 Jul 28;29(12):1975–87. doi: 10.1038/s41417-022-00513-x (PMC9750878; doi:10.1038/s41417-022-00513-x)
Supplement: Supplementary file 6 — Dataset original qPCR [file 41417_2022_513_MOESM6_ESM.zip › AATK_Capan1.pdf]

14 Hilly Street Mortlake NSW 2137 Australia
T +61 2 9736 1320
F +61 2 9736 1364
W www.corbettlifescience.com

# Comparative Quantitation Report

## Experiment Information

| | |
|---|---|
| Run Name | Run 2016-12-22_AATK_Aza_Pankreas |
| Run Start | 22.12.2016 11:15:27 |
| Run Finish | 22.12.2016 13:11:51 |
| Operator | MW |
| Notes | AATK Aza Pankeas triplicate |
| Run On Software Version | Rotor-Gene 6.1.93 |
| Run Signature | The Run Signature is valid. |
| Gain FAM | 8. |
| Gain ROX | 8. |

## Comparative Quantitation Information

| | |
|---|---|
| Reaction Amplification | 1.82 |
| Reaction Amplification Std. Deviation | 0.03 |
| Sample Page | Page 1 |
| Control Replicate | (1) |

## Take off Graph for Cycling A.FAM

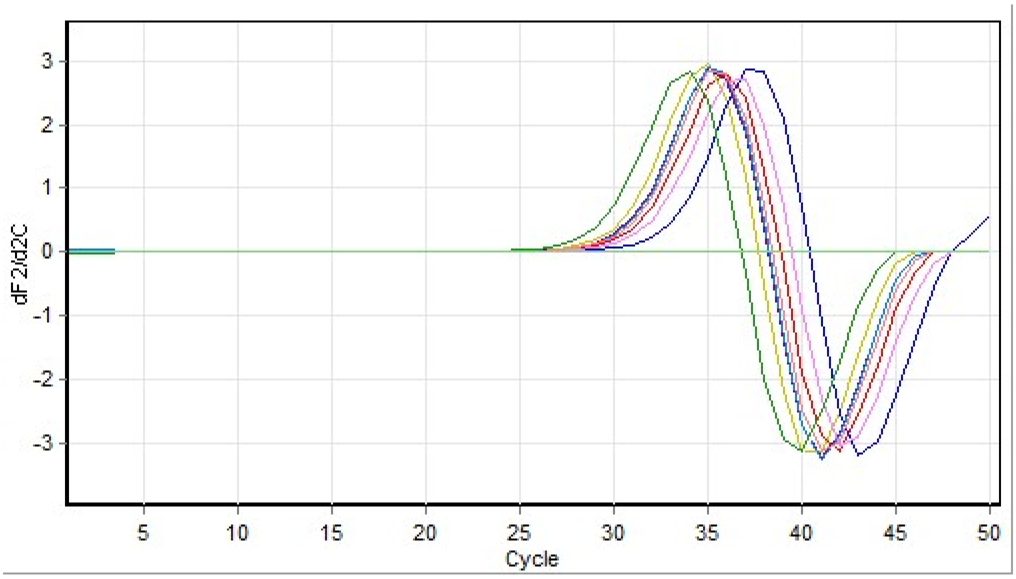


| No. | Colour | Name | Take Off | Amplification | Comparative Conc. | Rep. Takeoff | Rep. Takeoff (95% CI) |
|---|---|---|---|---|---|---|---|
| A1 | | Capan1 0uM | 32.4 | 1.80 | 1.13E+00 | 32.6 | [1.$,1.$] |
| A2 | | Capan1 0uM | 31.4 | 1.83 | 2.05E+00 | | |
| A3 | | Capan1 0uM | 34.0 | 1.86 | 4.33E-01 | | |
| A4 | | Capan1 5uM | 31.8 | 1.80 | 1.61E+00 | 32.2 | [1.$,1.$] |
| A5 | | Capan1 5uM | 32.9 | 1.78 | 8.36E-01 | | |
| A6 | | Capan1 5uM | 31.9 | 1.84 | 1.52E+00 | | |
| A7 | | Capan1 10uM | 30.4 | 1.83 | 3.73E+00 | 30.9 | [1.$,1.$] |
| A8 | | Capan1 10uM | 32.0 | 1.83 | 1.43E+00 | | |
| B1 | | Capan1 10uM | 30.3 | 1.80 | 3.96E+00 | | |
| F6 | | H2O | 17.3 | 0.58 | 9.50E+03 | 17.3 | |

Quality System ™
Quality Endorsed Company
ISO 9001 Lic 21313
SAI Global

This report generated by Rotor-Gene Real-Time Analysis Software 6.1 (Build 93)
© Corbett Research 2005
® All Rights Reserved
ISO 9001:2000 (Reg. No. QEC21313)
