# Supplementary material for: Epigenetically silenced apoptosis-associated tyrosine kinase (AATK) facilitates a decreased expression of Cyclin D1 and WEE1, phosphorylates TP53 and reduces cell proliferation in a kinase-dependent manner
Source: Cancer Gene Ther. 2022 Jul 28;29(12):1975–87. doi: 10.1038/s41417-022-00513-x (PMC9750878; doi:10.1038/s41417-022-00513-x)
Supplement: Supplementary file 6 — Dataset original qPCR [file 41417_2022_513_MOESM6_ESM.zip › HEK_CCND1.pdf]

14 Hilly Street Mortlake NSW 2137 Australia
T +61 2 9736 1320
F +61 2 9736 1364
W www.corbettlifescience.com

Comparative Quantitation Report

Experiment Information

Run Name	Run 2020-06-04_CCND1_HEK-UV_starved_(1)_(2)
Run Start	04.06.2020 09:39:06
Run Finish	04.06.2020 11:34:15
Operator	MW
Notes	CCND1 UV HEK EY OE starved triplicate
Run On Software Version	Rotor-Gene 6.1.93
Run Signature	The Run Signature is valid.
Gain FAM	8.
Gain ROX	9.33

Comparative Quantitation Information

Reaction Amplification	1.69
Reaction Amplification Std. Deviation	0.02
Sample Page	Page 1
Control Replicate	(1)

Take off Graph for Cycling A.FAM/Cycling A.ROX

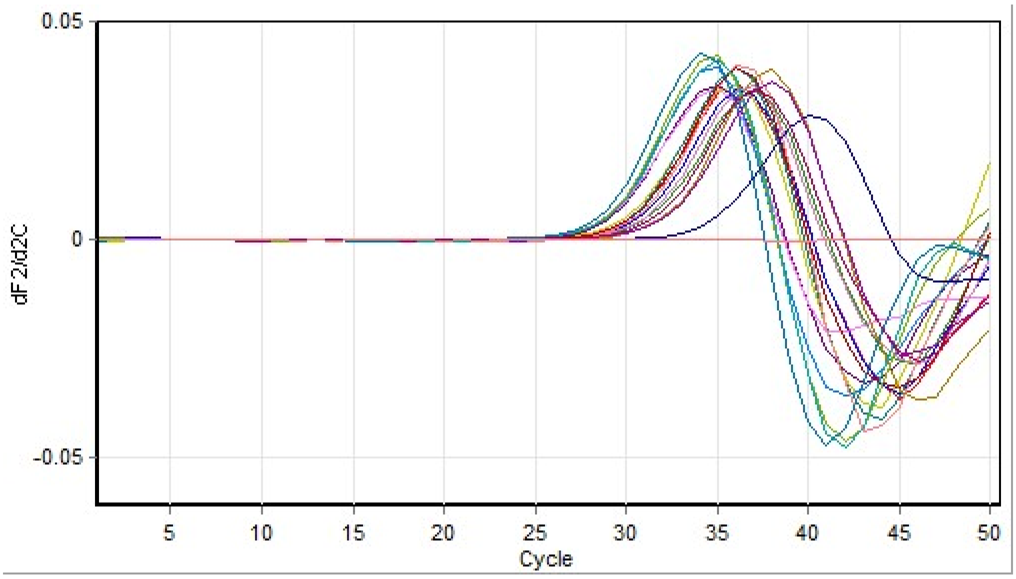

No.	Colour	Name	Take Off	Amplification	Comparative Conc.	Rep. Takeoff	Rep. Takeoff (95% CI)
A1		EY ohne (1)	31.5	1.71	1.02E+00	31.5	[1.$,1.$]
A2		EY ohne (1)	31.2	1.70	1.19E+00		
A3		EY ohne (1)	31.9	1.69	8.24E-01		
A4		B KD ohne (1)	30.3	1.73	1.92E+00	30.3	[1.$,1.$]
A5		B KD ohne (1)	30.2	1.67	2.02E+00		
A6		B KD ohne (1)	30.3	1.69	1.92E+00		
A7		B ohne (1)	31.6	1.75	9.65E-01	32.0	[1.$,1.$]
A8		B ohne (1)	32.1	1.68	7.42E-01		
B1		B ohne (1)	32.4	1.69	6.33E-01		
C3		EY ohne (2)	32.2	1.72	7.04E-01	32.4	[1.$,1.$]
C4		EY ohne (2)	31.8	1.67	8.69E-01		
C5		EY ohne (2)	33.3	1.68	3.94E-01		
C6		B KD ohne (2)	30.3	1.68	1.92E+00	30.2	[1.$,1.$]
C7		B KD ohne (2)	30.4	1.70	1.82E+00		
C8		B KD ohne (2)	30.0	1.70	2.24E+00		
D1		B ohne (2)	35.8	1.67	1.06E-01	33.9	[1.$,1.$]
D2		B ohne (2)	33.3	1.65	3.94E-01		
D3		B ohne (2)	32.6	1.72	5.70E-01		
E8		H2O	32.1	0.00	7.42E-01	32.1	

Quality System
Quality Endorsed Company
ISO 9001 Lic 21313
SAI Global

™ This report generated by Rotor-Gene Real-Time Analysis Software 6.1 (Build 93)
© Corbett Research 2005
® All Rights Reserved
ISO 9001:2000 (Reg. No. QEC21313)
