# Supplementary material for: Epigenetically silenced apoptosis-associated tyrosine kinase (AATK) facilitates a decreased expression of Cyclin D1 and WEE1, phosphorylates TP53 and reduces cell proliferation in a kinase-dependent manner
Source: Cancer Gene Ther. 2022 Jul 28;29(12):1975–87. doi: 10.1038/s41417-022-00513-x (PMC9750878; doi:10.1038/s41417-022-00513-x)
Supplement: Supplementary file 6 — Dataset original qPCR [file 41417_2022_513_MOESM6_ESM.zip › GAPDH_clone pools.pdf]

14 Hilly Street Mortlake NSW 2137 Australia
T +61 2 9736 1320
F +61 2 9736 1364
W www.corbettlifescience.com

Comparative Quantitation Report

Experiment Information

Run Name	Run 2019-02-19_GAPDH_AATK_Klone24h_fürAffy
Run Start	18.02.2019 17:07:51
Run Finish	18.02.2019 19:01:43
Operator	MW
Notes	GAPDH AATK Klone 24 h für Affy triplicate
Run On Software Version	Rotor-Gene 6.1.93
Run Signature	The Run Signature is valid.
Gain FAM	9.33
Gain ROX	9.33

Comparative Quantitation Information

Reaction Amplification	1.70
Reaction Amplification Std. Deviation	0.03
Sample Page	Page 1
Control Replicate	(32)

Take off Graph for Cycling A.FAM/Cycling A.ROX

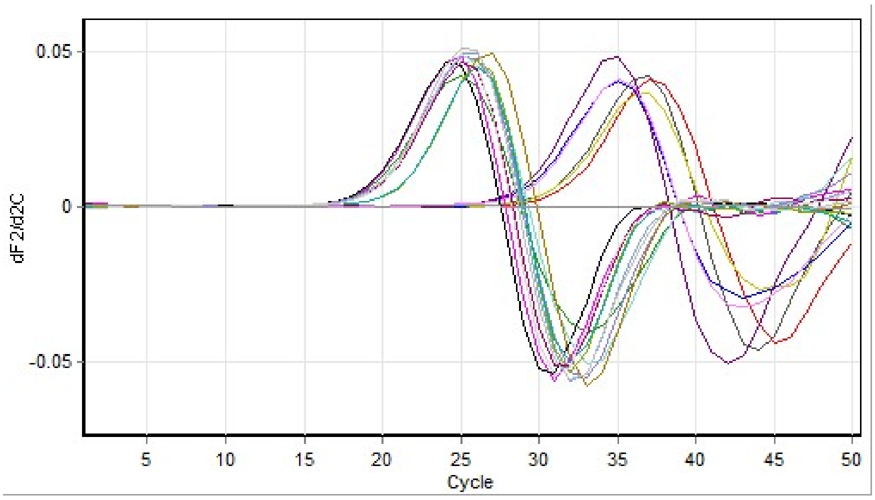

No.	Colour	Name	Take Off	Amplification	Comparative Conc.	Rep. Takeoff	Rep. Takeoff (95% CI)
D8		Control clone pool (1)_AATK	32.2	1.71	1.07E+00	32.3	[1.$,1.$]
E1		Control clone pool (1)_AATK	32.8	1.74	7.81E-01		
E2		Control clone pool (1)_AATK	32.0	1.74	1.19E+00		
E3		Control clone pool (2)_AATK	30.3	1.64	2.93E+00	30.4	[1.$,1.$]
E4		Control clone pool (2)_AATK	30.3	1.66	2.93E+00		
E5		Control clone pool (2)_AATK	30.7	1.67	2.37E+00		
F1		Clone pool AATK (1) _AATK	20.4	1.65	5.53E+02	20.4	[1.$,1.$]
F2		Clone pool AATK (1) _AATK	20.4	1.71	5.53E+02		
F3		Clone pool AATK (1) _AATK	20.3	1.74	5.83E+02		
F7		Clone pool AATK KD (1) _AATK	21.0	1.66	4.02E+02	21.0	[1.$,1.$]
F8		Clone pool AATK KD (1) _AATK	21.1	1.69	3.82E+02		
G1		Clone pool AATK KD (1) _AATK	20.9	1.75	4.24E+02		
G5		Clone pool AATK (2) _AATK	22.5	1.70	1.82E+02	22.4	[1.$,1.$]
G6		Clone pool AATK (2) _AATK	22.4	1.72	1.92E+02		
G7		Clone pool AATK (2) _AATK	22.3	1.71	2.02E+02		
H3		Clone pool AATK KD (2) _AATK	21.0	1.69	4.02E+02	21.1	[1.$,1.$]
H4		Clone pool AATK KD (2) _AATK	21.2	1.68	3.62E+02		
H5		Clone pool AATK KD (2) _AATK	21.1	1.71	3.82E+02		
H6		H2O AATK	42.7	0.00	4.14E-03	42.7	

This report generated by Rotor-Gene Real-Time Analysis Software 6.1 (Build 93)
© Corbett Research 2005
® All Rights Reserved
ISO 9001:2000 (Reg. No. QEC21313)
