# Supplementary material for: Epigenetically silenced apoptosis-associated tyrosine kinase (AATK) facilitates a decreased expression of Cyclin D1 and WEE1, phosphorylates TP53 and reduces cell proliferation in a kinase-dependent manner
Source: Cancer Gene Ther. 2022 Jul 28;29(12):1975–87. doi: 10.1038/s41417-022-00513-x (PMC9750878; doi:10.1038/s41417-022-00513-x)
Supplement: Supplementary file 6 — Dataset original qPCR [file 41417_2022_513_MOESM6_ESM.zip › U251_WEE1.pdf]

corbett
RESEARCH
A Division of Corbett Life Science

14 Hilly Street Mortlake NSW 2137 Australia
T +61 2 9736 1320
F +61 2 9736 1364
W www.corbettlifescience.com

Comparative Quantitation Report

Experiment Information

Run Name	Run 2020-10-16_WEE1_OE-U343_U251_A549_A427_(1)(2)
Run Start	16.10.2020 09:19:03
Run Finish	16.10.2020 11:04:07
Operator	MW
Notes	Wee1 OE u343 u251 a549 a427 (1)(2) triplicate
Run On Software Version	Rotor-Gene 6.1.93
Run Signature	The Run Signature is valid.
Gain FAM	8.
Gain ROX	8.

Comparative Quantitation Information

Reaction Amplification	1.66
Reaction Amplification Std. Deviation	0.03
Sample Page	Page 1
Control Replicate	(19)

Take off Graph for Cycling A.FAM/Cycling A.ROX

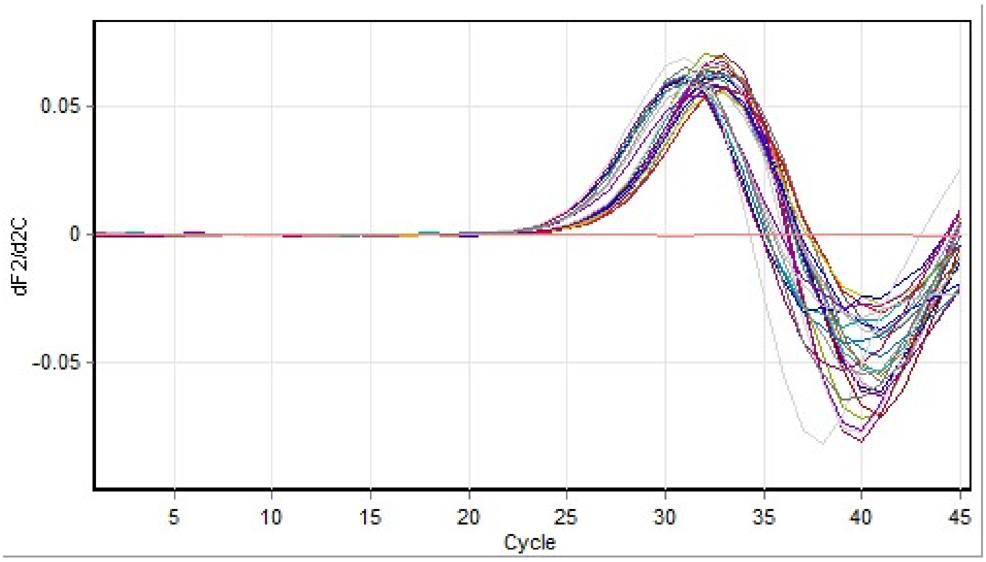

No.	Colour	Name	Take Off	Amplification	Comparative Conc.	Rep. Takeoff	Rep. Takeoff (95% CI)
C3		U251 EY (1)	28.1	1.64	1.03E+00	28.2	[1.$,1.$]
C4		U251 EY (1)	28.3	1.66	9.35E-01		
C5		U251 EY (1)	28.1	1.64	1.03E+00		
C6		U251 B-EY (1)	27.9	1.65	1.14E+00	27.9	[1.$,1.$]
C7		U251 B-EY (1)	27.7	1.62	1.27E+00		
C8		U251 B-EY (1)	28.1	1.67	1.03E+00		
D1		U251 B KD-EY (1)	27.8	1.68	1.20E+00	28.2	[1.$,1.$]
D2		U251 B KD-EY (1)	28.2	1.68	9.83E-01		
D3		U251 B KD-EY (1)	28.5	1.62	8.45E-01		
D4		U251 EY (2)	27.8	1.70	1.20E+00	27.7	[1.$,1.$]
D5		U251 EY (2)	27.5	1.62	1.40E+00		
D6		U251 EY (2)	27.9	1.65	1.14E+00		
D7		U251 B-EY (2)	28.5	1.67	8.45E-01	28.5	[1.$,1.$]
D8		U251 B-EY (2)	28.5	1.62	8.45E-01		
E1		U251 B-EY (2)	28.4	1.59	8.89E-01		
E2		U251 B KD-EY (2)	27.9	1.69	1.14E+00	27.8	[1.$,1.$]
E3		U251 B KD-EY (2)	27.8	1.67	1.20E+00		
E4		U251 B KD-EY (2)	27.7	1.68	1.27E+00		

(Continued on next page)...

No.	Colour	Name	Take Off	Amplification	Comparative Conc.	Rep. Takeoff	Rep. Takeoff (95% CI)
G7		U251 EY (3)	26.3	1.71	2.57E+00	26.3	[1.$,1.$]
G8		U251 EY (3)	26.3	1.64	2.57E+00		
H1		U251 EY (3)	26.3	1.65	2.57E+00		
H2		U251 B KD-EY (3)	26.6	1.62	2.21E+00	26.4	[1.$,1.$]
H3		U251 B KD-EY (3)	26.2	1.70	2.70E+00		

H4		U251 B KD-EY (3)	26.3	1.71	2.57E+00		
H5		U251 B-EY (3)	26.7	1.63	2.10E+00	26.6	[1.$,1.$]
H6		U251 B-EY (3)	26.6	1.68	2.21E+00		
H7		U251 B-EY (3)	26.6	1.65	2.21E+00		
I8		H2O	24.3	0.00	7.05E+00	24.3	

Quality System ™
Quality Endorsed Company
ISO 9001 Lic 21313
SAI Global

This report generated by Rotor-Gene Real-Time Analysis Software 6.1 (Build 93)
© Corbett Research 2005
® All Rights Reserved
ISO 9001:2000 (Reg. No. QEC21313)
