# Supplementary material for: Epigenetically silenced apoptosis-associated tyrosine kinase (AATK) facilitates a decreased expression of Cyclin D1 and WEE1, phosphorylates TP53 and reduces cell proliferation in a kinase-dependent manner
Source: Cancer Gene Ther. 2022 Jul 28;29(12):1975–87. doi: 10.1038/s41417-022-00513-x (PMC9750878; doi:10.1038/s41417-022-00513-x)
Supplement: Supplementary file 6 — Dataset original qPCR [file 41417_2022_513_MOESM6_ESM.zip › Epigen.edit_AATK_1.pdf]

14 Hilly Street Mortlake NSW 2137 Australia
T + 61 2 9736 1320
F + 61 2 9736 1364
W www.corbettlifescience.com

Comparative Quantitation Report

Experiment Information

Run Name	Run 2019-03-01_AATK_HEK_1.Epig.
Run Start	28.02.2019 14:41:37
Run Finish	28.02.2019 16:35:48
Operator	MW
Notes	AATK 1. Epig. ed. HEK triplicate
Run On Software Version	Rotor-Gene 6.1.93
Run Signature	The Run Signature is valid.
Gain FAM	8.
Gain ROX	9.33

Comparative Quantitation Information

Reaction Amplification	1.58
Reaction Amplification Std. Deviation	0.11
Sample Page	Page 1
Control Replicate	(4)

Take off Graph for Cycling A.FAM/Cycling A.ROX

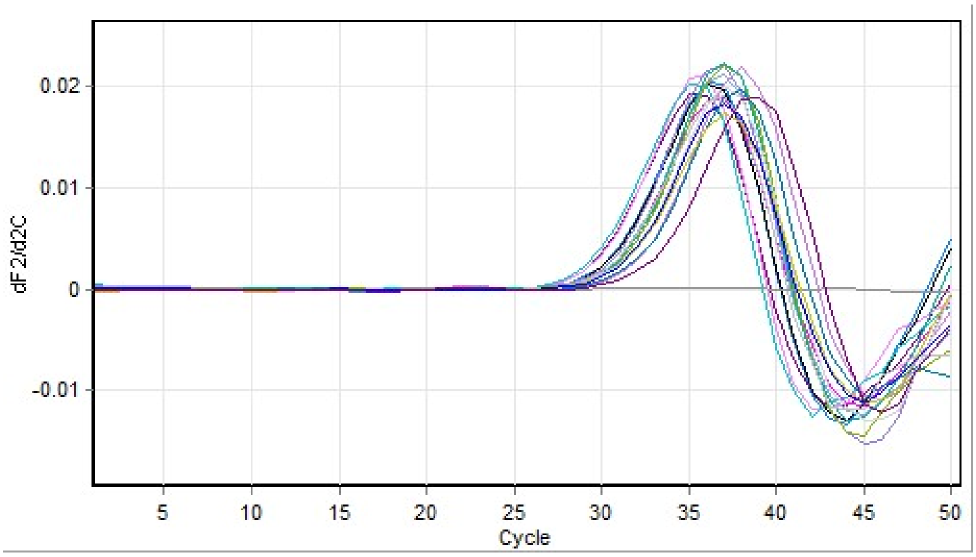

No.	Colour	Name	Take Off	Amplification	Comparative Conc.	Rep. Takeoff	Rep. Takeoff (95% CI)
A4		Oligo Mix pcDNA	30.9	1.62	1.56E+00	31.9	[1.$,1.$]
A5		Oligo Mix pcDNA	31.1	1.66	1.42E+00		
A6		Oligo Mix pcDNA	31.6	1.49	1.13E+00		
B2		Oligo Mix p300	32.0	1.70	9.40E-01	31.5	[1.$,1.$]
B3		Oligo Mix p300	31.8	1.58	1.03E+00		
B4		Oligo Mix p300	30.6	1.48	1.79E+00		
B8		Oligo Mix pcDNA	32.2	1.70	8.58E-01		
C1		Oligo Mix pcDNA	32.0	1.71	9.40E-01		
C2		Oligo Mix pcDNA	33.4	1.69	4.94E-01		
C6		Oligo Mix EZH2	32.5	1.44	7.47E-01	32.7	[1.$,1.$]
C7		Oligo Mix EZH2	32.4	1.41	7.82E-01		
C8		Oligo Mix EZH2	33.2	1.71	5.41E-01		
D4		Oligo Mix pcDNA3	32.6	1.54	7.14E-01	31.2	[1.$,1.$]
D5		Oligo Mix pcDNA3	32.0	1.64	9.40E-01		
D6		Oligo Mix pcDNA3	29.1	0.00	3.57E+00		
E2		Oligo Mix DNMT3A	32.4	1.42	7.82E-01	33.0	[1.$,1.$]
E3		Oligo Mix DNMT3A	32.5	1.47	7.47E-01		
E4		Oligo Mix DNMT3A	34.1	1.67	3.58E-01		

Quality System
Quality Endorsed Company
ISO 9001 Lic 21313
SAI Global

This report generated by Rotor-Gene Real-Time Analysis Software 6.1 (Build 93)
© Corbett Research 2005
® All Rights Reserved
ISO 9001:2000 (Reg. No. QEC21313)
