# Supplementary material for: Epigenetically silenced apoptosis-associated tyrosine kinase (AATK) facilitates a decreased expression of Cyclin D1 and WEE1, phosphorylates TP53 and reduces cell proliferation in a kinase-dependent manner
Source: Cancer Gene Ther. 2022 Jul 28;29(12):1975–87. doi: 10.1038/s41417-022-00513-x (PMC9750878; doi:10.1038/s41417-022-00513-x)
Supplement: Supplementary file 6 — Dataset original qPCR [file 41417_2022_513_MOESM6_ESM.zip › HEK_NOVA1.pdf]

corbett
RESEARCH
A Division of Corbett Life Science

14 Hilly Street Mortlake NSW 2137 Australia
T +61 2 9736 1320
F +61 2 9736 1364
W www.corbettlifescience.com

Comparative Quantitation Report

Experiment Information

Run Name	Run 2020-06-17_NOVA1_RNAi-HEK(2)(3)_UV(1)(2)
Run Start	17.06.2020 10:07:14
Run Finish	17.06.2020 12:06:04
Operator	MW
Notes	NOVA1 HEK RNAi (2) (3), UV (1) (2) triplicate
Run On Software Version	Rotor-Gene 6.1.93
Run Signature	The Run Signature is valid.
Gain FAM	8.
Gain ROX	9.33

Comparative Quantitation Information

Reaction Amplification	1.71
Reaction Amplification Std. Deviation	0.02
Sample Page	Page 1
Control Replicate	(37)

Take off Graph for Cycling A.FAM/Cycling A.ROX

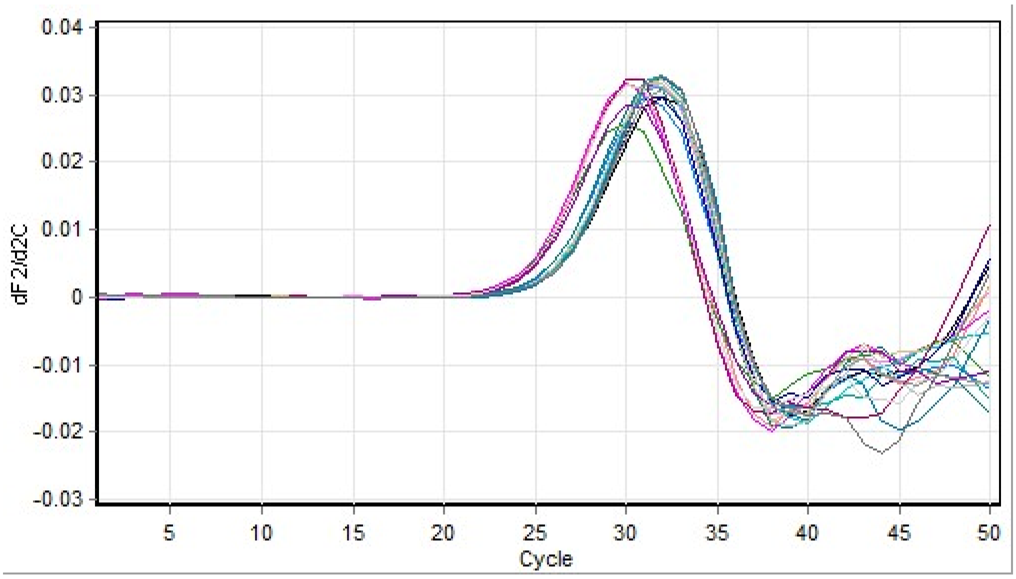

No.	Colour	Name	Take Off	Amplification	Comparative Conc.	Rep. Takeoff	Rep. Takeoff (95% CI)
E5		ohne EY (2)	27.4	1.72	8.35E-01	27.1	[1.$,1.$]
E6		ohne EY (2)	26.8	1.64	1.15E+00		
E7		ohne EY (2)	27.0	1.71	1.04E+00		
E8		ohne B KD (2)	25.8	1.70	1.98E+00	25.7	[1.$,1.$]
F1		ohne B KD (2)	25.5	1.74	2.33E+00		
F2		ohne B KD (2)	25.7	1.68	2.09E+00		
F3		ohne B (2)	27.5	1.69	7.92E-01	27.5	[1.$,1.$]
F4		ohne B (2)	27.4	1.72	8.35E-01		
F5		ohne B (2)	27.5	1.73	7.92E-01		
G7		ohne EY (3)	27.6	1.72	7.50E-01	27.6	[1.$,1.$]
G8		ohne EY (3)	27.6	1.71	7.50E-01		
H1		ohne EY (3)	27.6	1.70	7.50E-01		
H2		ohne B KD (3)	25.9	1.74	1.88E+00	25.9	[1.$,1.$]
H3		ohne B KD (3)	26.0	1.71	1.78E+00		
H4		ohne B KD (3)	25.9	1.62	1.88E+00		
H5		ohne B (3)	27.3	1.74	8.82E-01	27.5	[1.$,1.$]
H6		ohne B (3)	27.6	1.71	7.50E-01		
H7		ohne B (3)	27.7	1.73	7.11E-01		

Quality System Quality Endorsed Company ISO 9001 Lic 21313 SAI Global
™ This report generated by Rotor-Gene Real-Time Analysis Software 6.1 (Build 93)
© Corbett Research 2005
® All Rights Reserved
ISO 9001:2000 (Reg. No. QEC21313)
