# Supplementary material for: Epigenetically silenced apoptosis-associated tyrosine kinase (AATK) facilitates a decreased expression of Cyclin D1 and WEE1, phosphorylates TP53 and reduces cell proliferation in a kinase-dependent manner
Source: Cancer Gene Ther. 2022 Jul 28;29(12):1975–87. doi: 10.1038/s41417-022-00513-x (PMC9750878; doi:10.1038/s41417-022-00513-x)
Supplement: Supplementary file 6 — Dataset original qPCR [file 41417_2022_513_MOESM6_ESM.zip › AATK_PaCa2.pdf]

corbett RESEARCH
A Division of Corbett Life Science

14 Hilly Street Mortlake NSW 2137 Australia
T +61 2 9736 1320
F +61 2 9736 1364
W www.corbettlifescience.com

Comparative Quantitation Report

Experiment Information

Run Name	Run 2017-01-19_AATK_Pankreas_Aza_all
Run Start	19.01.2017 14:18:51
Run Finish	19.01.2017 16:16:28
Operator	MW
Notes	AATK Aza Pankeas all triplicate
Run On Software Version	Rotor-Gene 6.1.93
Run Signature	The Run Signature is valid.
Gain FAM	8.
Gain ROX	9.33

Comparative Quantitation Information

Reaction Amplification	1.83
Reaction Amplification Std. Deviation	0.06
Sample Page	Page 1
Control Replicate	(13)

Take off Graph for Cycling A.FAM

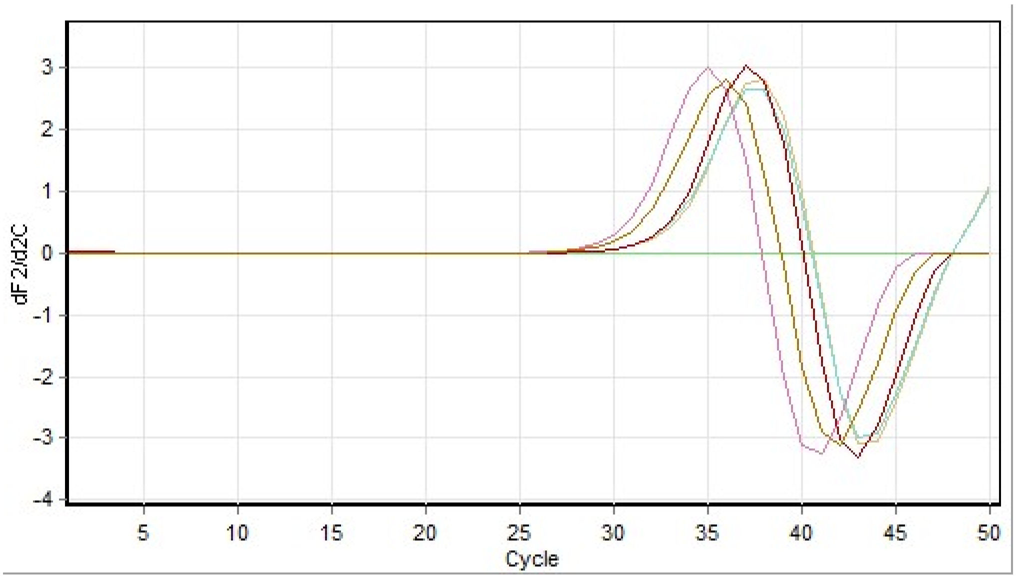

No.	Colour	Name	Take Off	Amplification	Comparative Conc.	Rep. Takeoff	Rep. Takeoff (95% CI)
B5		PaCa2 0 uM	34.1	1.82	5.82E-01	33.2	
B6		PaCa2 0 uM	32.3	1.11	1.72E+00		
B7		PaCa2 0 uM	33.8	1.75	6.97E-01	33.8	
C3		PaCa2 5 uM	31.7	1.87	2.47E+00	32.6	[1.$,1.$]
C4		PaCa2 5 uM	33.8	1.89	6.97E-01		
C5		PaCa2 5 uM	32.3	1.80	1.72E+00		

Quality System™
Quality Endorsed Company
ISO 9001 Lic 21313
SAI Global

This report generated by Rotor-Gene Real-Time Analysis Software 6.1 (Build 93)
© Corbett Research 2005
® All Rights Reserved
ISO 9001:2000 (Reg. No. QEC21313)
