# Supplementary material for: Epigenetically silenced apoptosis-associated tyrosine kinase (AATK) facilitates a decreased expression of Cyclin D1 and WEE1, phosphorylates TP53 and reduces cell proliferation in a kinase-dependent manner
Source: Cancer Gene Ther. 2022 Jul 28;29(12):1975–87. doi: 10.1038/s41417-022-00513-x (PMC9750878; doi:10.1038/s41417-022-00513-x)
Supplement: Supplementary file 6 — Dataset original qPCR [file 41417_2022_513_MOESM6_ESM.zip › U343_CCND1.pdf]

corbett RESEARCH
A Division of Corbett Life Science

14 Hilly Street Mortlake NSW 2137 Australia
T +61 2 9736 1320
F +61 2 9736 1364
W www.corbettlifescience.com

Comparative Quantitation Report

Experiment Information

Run Name	Run 2020-09-18_CCND1_OE_(1)(2)_U343_U251_A549_A427
Run Start	18.09.2020 12:23:09
Run Finish	18.09.2020 14:19:08
Operator	MW
Notes	CCND1 OE EY (1) (2) U343 U251 A549 A427 triplicate
Run On Software Version	Rotor-Gene 6.1.93
Run Signature	The Run Signature is valid.
Gain FAM	8.
Gain ROX	9.33

Comparative Quantitation Information

Reaction Amplification	1.60
Reaction Amplification Std. Deviation	0.10
Sample Page	Page 1
Control Replicate	(1)

Take off Graph for Cycling A.FAM/Cycling A.ROX

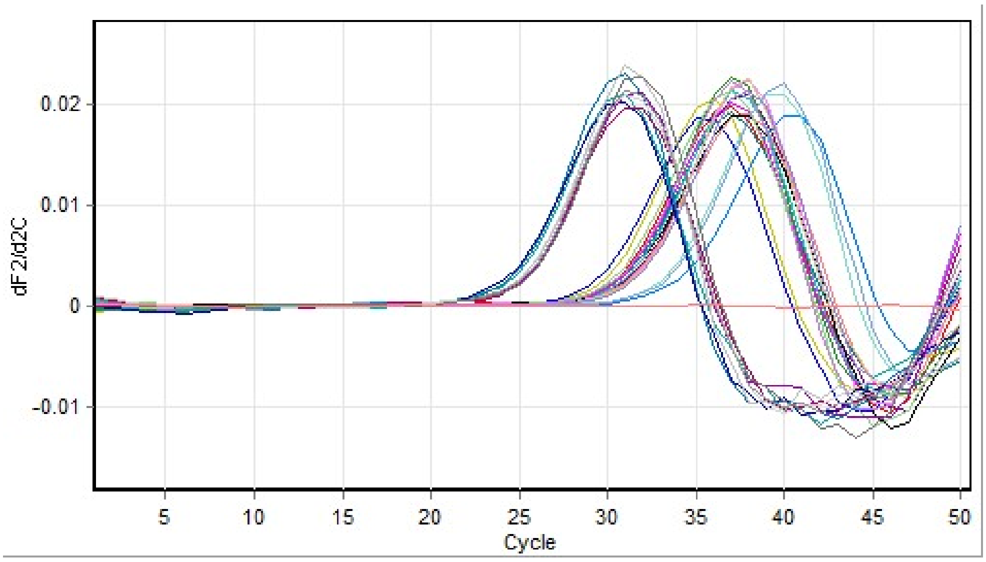

No.	Colour	Name	Take Off	Amplification	Comparative Conc.	Rep. Takeoff	Rep. Takeoff (95% CI)
A1		U343 EY (1)	31.8	1.62	7.66E-01	31.2	[1.$,1.$]
A2		U343 EY (1)	31.2	1.69	1.02E+00		
A3		U343 EY (1)	30.7	1.61	1.28E+00		
A4		U343 B-EY (1)	32.6	1.55	5.26E-01	33.6	[1.$,1.$]
A5		U343 B-EY (1)	33.0	1.66	4.36E-01		
A6		U343 B-EY (1)	35.3	1.62	1.48E-01		
A7		U343 B KD-EY (1)	32.0	1.65	6.98E-01	32.3	[1.$,1.$]
A8		U343 B KD-EY (1)	32.6	1.51	5.26E-01		
B1		U343 B KD-EY (1)	32.3	1.73	6.06E-01		
B2		U343 EY (2)	32.1	1.69	6.66E-01	32.3	[1.$,1.$]
B3		U343 EY (2)	32.2	1.27	6.35E-01		
B4		U343 EY (2)	32.5	1.47	5.52E-01		
B5		U343 B-EY (2)	32.9	1.64	4.57E-01	33.1	[1.$,1.$]
B6		U343 B-EY (2)	31.8	1.59	7.66E-01		
B7		U343 B-EY (2)	34.6	1.66	2.06E-01		
B8		U343 B KD-EY (2)	34.8	1.62	1.87E-01	33.4	[1.$,1.$]
C1		U343 B KD-EY (2)	33.0	1.67	4.36E-01		
C2		U343 B KD-EY (2)	32.3	1.66	6.06E-01		

(Continued on next page)...

No.	Colour	Name	Take Off	Amplification	Comparative Conc.	Rep. Takeoff	Rep. Takeoff (95% CI)
G7		U343 EY (3)	26.0	1.66	1.17E+01	25.9	[1.$,1.$]
G8		U343 EY (3)	25.9	1.66	1.22E+01		
H1		U343 EY (3)	25.7	1.60	1.34E+01		
H2		U343 B-EY (3)	26.6	1.57	8.81E+00	26.4	[1.$,1.$]
H3		U343 B-EY (3)	26.4	1.47	9.68E+00		

H4		U343 B-EY (3)	26.3	1.37	1.01E+01		
H5		U343 B KD-EY (3)	26.8	1.67	8.02E+00	26.8	[1.$,1.$]
H6		U343 B KD-EY (3)	26.7	1.59	8.40E+00		
H7		U343 B KD-EY (3)	27.0	1.67	7.30E+00		
I8		H2O	10.1	0.00	2.04E+04	10.1	

Quality System™
Quality Endorsed Company
ISO 9001 Lic 21313
SAI Global

This report generated by Rotor-Gene Real-Time Analysis Software 6.1 (Build 93)
© Corbett Research 2005
® All Rights Reserved
ISO 9001:2000 (Reg. No. QEC21313)
