# Supplementary material for: Epigenetically silenced apoptosis-associated tyrosine kinase (AATK) facilitates a decreased expression of Cyclin D1 and WEE1, phosphorylates TP53 and reduces cell proliferation in a kinase-dependent manner
Source: Cancer Gene Ther. 2022 Jul 28;29(12):1975–87. doi: 10.1038/s41417-022-00513-x (PMC9750878; doi:10.1038/s41417-022-00513-x)
Supplement: Supplementary file 6 — Dataset original qPCR [file 41417_2022_513_MOESM6_ESM.zip › Epigen.edit_AATK_2.pdf]

14 Hilly Street Mortlake NSW 2137 Australia
T +61 2 9736 1320
F +61 2 9736 1364
W www.corbettlifescience.com

Comparative Quantitation Report

Experiment Information

Run Name	Run 2019-03-06_AATK_HEK_2.Epig.
Run Start	06.03.2019 12:07:38
Run Finish	06.03.2019 14:01:52
Operator	MW
Notes	AATK 2. Epig. ed. HEK triplicate
Run On Software Version	Rotor-Gene 6.1.93
Run Signature	The Run Signature is valid.
Gain FAM	8.
Gain ROX	9.33

Comparative Quantitation Information

Reaction Amplification	1.58
Reaction Amplification Std. Deviation	0.12
Sample Page	Page 1
Control Replicate	(1)

Take off Graph for Cycling A.FAM/Cycling A.ROX

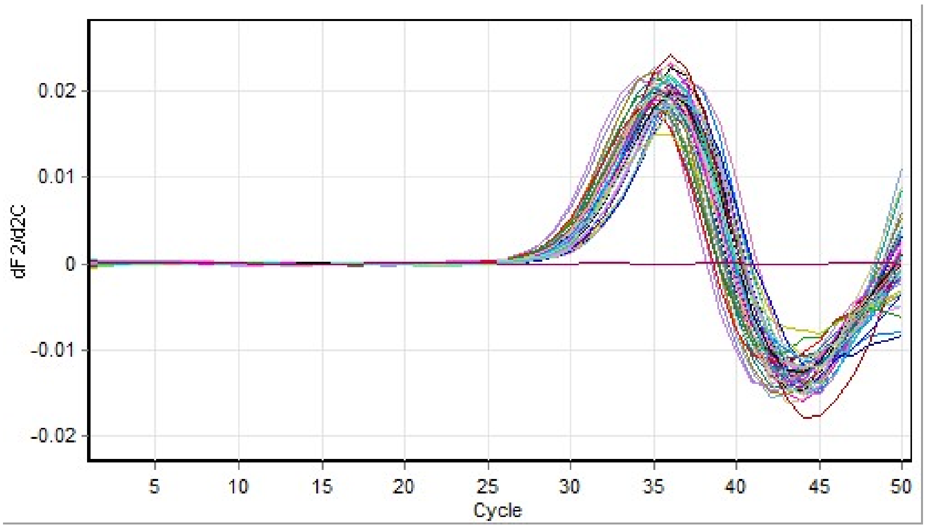

No.	Colour	Name	Take Off	Amplification	Comparative Conc.	Rep. Takeoff	Rep. Takeoff (95% CI)
A1		px459dCas9 pcDNA	30.7	1.54	1.36E+00	31.4	[1.$,1.$]
A2		px459dCas9 pcDNA	32.2	1.64	6.88E-01		
A3		px459dCas9 pcDNA	31.9	1.71	7.88E-01		
A4		Oligo Mix pcDNA	32.1	1.70	7.20E-01	30.9	[1.$,1.$]
A5		Oligo Mix pcDNA	31.8	1.63	8.25E-01		
A6		Oligo Mix pcDNA	32.7	1.57	5.48E-01		
A7		px459dCas9 p300	30.4	1.49	1.56E+00	30.5	[1.$,1.$]
A8		px459dCas9 p300	31.0	1.69	1.19E+00		
B1		px459dCas9 p300	30.2	1.71	1.71E+00		
B2		Oligo Mix p300	31.3	1.71	1.04E+00	31.6	[1.$,1.$]
B3		Oligo Mix p300	31.9	1.67	7.88E-01		
B4		Oligo Mix p300	31.6	1.73	9.04E-01		
B5		px459dCas9 pcDNA	31.7	1.57	8.64E-01		
B6		px459dCas9 pcDNA	31.0	1.67	1.19E+00		
B7		px459dCas9 pcDNA	31.2	1.71	1.08E+00		
B8		Oligo Mix pcDNA	30.6	1.77	1.42E+00		
C1		Oligo Mix pcDNA	29.8	1.60	2.05E+00		
C2		Oligo Mix pcDNA	29.7	1.60	2.15E+00		

(Continued on next page)...

No.	Colour	Name	Take Off	Amplification	Comparative Conc.	Rep. Takeoff	Rep. Takeoff (95% CI)
C3		px459dCas9 EZH2	32.6	1.52	5.73E-01	31.5	[1.$,1.$]
C4		px459dCas9 EZH2	31.7	1.54	8.64E-01		
C5		px459dCas9 EZH2	30.3	1.74	1.63E+00		
C6		Oligo Mix EZH2	31.6	1.50	9.04E-01	31.2	[1.$,1.$]
C7		Oligo Mix EZH2	31.4	1.42	9.90E-01		
C8		Oligo Mix EZH2	30.6	1.52	1.42E+00		
D1		px459dCas9 pcDNA	31.1	1.60	1.13E+00		
D2		px459dCas9 pcDNA	31.4	1.44	9.90E-01		

D3		px459dCas9 pcDNA	31.2	1.73	1.08E+00		
D4		Oligo Mix pcDNA3	30.8	1.64	1.30E+00	30.8	[1.$,1.$]
D5		Oligo Mix pcDNA3	31.2	1.68	1.08E+00		
D6		Oligo Mix pcDNA3	30.4	1.46	1.56E+00		
D7		px459 dCas9 DNMt3A	30.6	1.57	1.42E+00	30.2	[1.$,1.$]
D8		px459 dCas9 DNMt3A	30.3	1.44	1.63E+00		
E1		px459 dCas9 DNMt3A	29.8	1.61	2.05E+00		
E2		Oligo Mix DNMT3A	30.8	1.58	1.30E+00	31.5	[1.$,1.$]
E3		Oligo Mix DNMT3A	32.3	1.35	6.57E-01		
E4		Oligo Mix DNMT3A	31.5	1.44	9.46E-01		
E5		px459dCas9 pcDNA3	31.1	1.59	1.13E+00	30.9	[1.$,1.$]
E6		px459dCas9 pcDNA3	30.8	1.62	1.30E+00		
E7		px459dCas9 pcDNA3	30.7	1.56	1.36E+00		
E8		Oligo Mix pcDNA	30.7	1.62	1.36E+00		
F1		Oligo Mix pcDNA	30.0	1.22	1.87E+00		
F2		Oligo Mix pcDNA	30.6	1.52	1.42E+00		
F3		px459dCas9 DNMT3A+L	31.6	1.53	9.04E-01	31.2	[1.$,1.$]
F4		px459dCas9 DNMT3A+L	31.4	1.68	9.90E-01		
F5		px459dCas9 DNMT3A+L	30.7	1.53	1.36E+00		
F6		Oligo Mix DNMT3A+L	32.2	1.30	6.88E-01	31.8	[1.$,1.$]
F7		Oligo Mix DNMT3A+L	31.4	1.44	9.90E-01		
F8		Oligo Mix DNMT3A+L	31.8	1.56	8.25E-01		
H3		H2O	38.1	0.00	4.69E-02	38.1	

This report generated by Rotor-Gene Real-Time Analysis Software 6.1 (Build 93)
© Corbett Research 2005
® All Rights Reserved
ISO 9001:2000 (Reg. No. QEC21313)
