# Supplementary material for: Epigenetically silenced apoptosis-associated tyrosine kinase (AATK) facilitates a decreased expression of Cyclin D1 and WEE1, phosphorylates TP53 and reduces cell proliferation in a kinase-dependent manner
Source: Cancer Gene Ther. 2022 Jul 28;29(12):1975–87. doi: 10.1038/s41417-022-00513-x (PMC9750878; doi:10.1038/s41417-022-00513-x)
Supplement: Supplementary file 6 — Dataset original qPCR [file 41417_2022_513_MOESM6_ESM.zip › Epigen.edit_AATK_3.pdf]

corbett RESEARCH
A Division of Corbett Life Science

14 Hilly Street Mortlake NSW 2137 Australia
T +61 2 9736 1320
F +61 2 9736 1364
W www.corbettlifescience.com

Comparative Quantitation Report

Experiment Information

Run Name	Run 2019-03-07_AATK_HEK_3.Epig.
Run Start	07.03.2019 10:42:18
Run Finish	07.03.2019 12:35:06
Operator	MW
Notes	AATK 3. Epig. ed. HEK triplicate
Run On Software Version	Rotor-Gene 6.1.93
Run Signature	The Run Signature is valid.
Gain FAM	8.
Gain ROX	8.

Comparative Quantitation Information

Reaction Amplification	1.67
Reaction Amplification Std. Deviation	0.04
Sample Page	Page 1
Control Replicate	(1)

Take off Graph for Cycling A.FAM/Cycling A.ROX

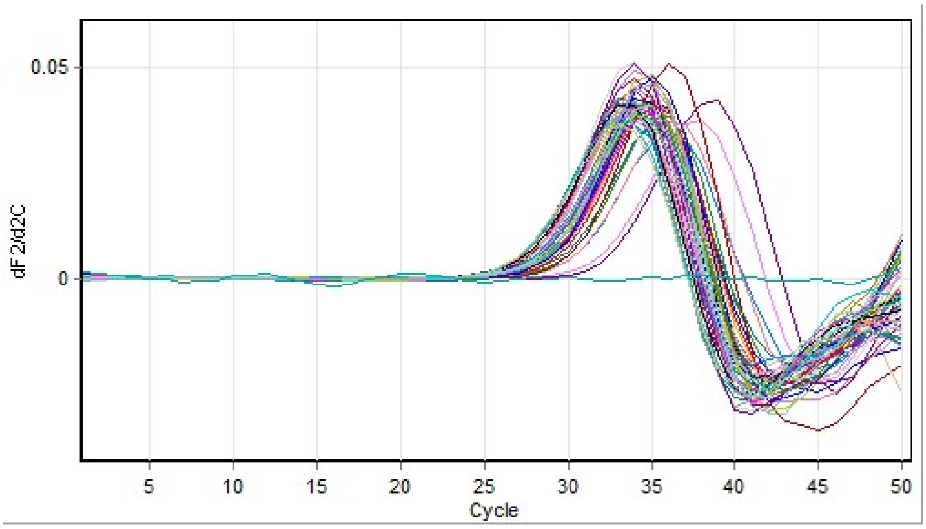

No.	Colour	Name	Take Off	Amplification	Comparative Conc.	Rep. Takeoff	Rep. Takeoff (95% CI)
A1		px459dCas9 pcDNA 1	30.4	1.65	1.02E+00	30.4	[1.$,1.$]
A2		px459dCas9 pcDNA 1	30.5	1.71	9.66E-01		
A3		px459dCas9 pcDNA 1	30.4	1.75	1.02E+00		
A4		Oligo Mix pcDNA 1	33.7	1.75	1.86E-01	32.7	[1.$,1.$]
A5		Oligo Mix pcDNA 1	33.1	1.64	2.53E-01		
A6		Oligo Mix pcDNA 1	31.3	1.76	6.40E-01		
A7		px459dCas9 p300	30.9	1.70	7.86E-01	31.2	[1.$,1.$]
A8		px459dCas9 p300	31.4	1.68	6.07E-01		
B1		px459dCas9 p300	31.2	1.70	6.73E-01		
B2		Oligo Mix p300	30.3	1.70	1.07E+00	30.1	[1.$,1.$]
B3		Oligo Mix p300	29.9	1.65	1.32E+00		
B4		Oligo Mix p300	30.2	1.72	1.13E+00		
B5		px459dCas9 pcDNA 2	30.4	1.72	1.02E+00	30.2	[1.$,1.$]
B6		px459dCas9 pcDNA 2	30.1	1.65	1.19E+00		
B7		px459dCas9 pcDNA 2	30.0	1.61	1.25E+00		
B8		Oligo Mix EZH2	28.9	1.63	2.21E+00	29.4	[1.$,1.$]
C1		Oligo Mix EZH2	29.4	1.66	1.70E+00		
C2		Oligo Mix EZH2	29.8	1.68	1.39E+00		

(Continued on next page)...

No.	Colour	Name	Take Off	Amplification	Comparative Conc.	Rep. Takeoff	Rep. Takeoff (95% CI)
C3		px459dCas9 EZH2	30.2	1.66	1.13E+00	30.7	[1.$,1.$]
C4		px459dCas9 EZH2	31.7	1.75	5.20E-01		
C5		px459dCas9 EZH2	30.3	1.65	1.07E+00		
C6		Oligo Mix pcDNA3	29.0	1.61	2.09E+00	29.0	[1.$,1.$]
C7		Oligo Mix pcDNA3	28.7	1.68	2.44E+00		
C8		Oligo Mix pcDNA3	28.7	1.72	2.44E+00		
D1		px459dCas9 pcDNA 3	29.9	1.61	1.32E+00	29.5	[1.$,1.$]
D2		px459dCas9 pcDNA 3	29.3	1.65	1.79E+00		

D3		px459dCas9 pcDNA 3	29.3	1.63	1.79E+00		
D4		Oligo Mix pcDNA3	29.4	1.66	1.70E+00		
D5		Oligo Mix pcDNA3	29.3	1.64	1.79E+00		
D6		Oligo Mix pcDNA3	28.7	1.64	2.44E+00		
D7		px459 dCas9 DNMt3A	29.7	1.73	1.46E+00	30.1	[1.$,1.$]
D8		px459 dCas9 DNMt3A	29.9	1.72	1.32E+00		
E1		px459 dCas9 DNMt3A	30.8	1.64	8.28E-01		
E2		Oligo Mix DNMT3A	30.2	1.68	1.13E+00	30.1	[1.$,1.$]
E3		Oligo Mix DNMT3A	30.6	1.71	9.18E-01		
E4		Oligo Mix DNMT3A	29.6	1.65	1.54E+00		
E5		px459dCas9 pcDNA3	29.5	1.66	1.62E+00	29.5	[1.$,1.$]
E6		px459dCas9 pcDNA3	29.3	1.70	1.79E+00		
E7		px459dCas9 pcDNA3	29.6	1.63	1.54E+00		
E8		Oligo Mix pcDNA	28.8	1.64	2.32E+00	29.5	[1.$,1.$]
F1		Oligo Mix pcDNA	30.0	1.78	1.25E+00		
F2		Oligo Mix pcDNA	29.6	1.67	1.54E+00		
F3		px459dCas9 DNMT3A+L	28.9	1.64	2.21E+00	28.5	[1.$,1.$]
F4		px459dCas9 DNMT3A+L	28.4	1.68	2.85E+00		
F5		px459dCas9 DNMT3A+L	28.2	1.62	3.16E+00		
F6		Oligo Mix DNMT3A+L	29.5	1.73	1.62E+00	29.9	[1.$,1.$]
F7		Oligo Mix DNMT3A+L	30.0	1.62	1.25E+00		
F8		Oligo Mix DNMT3A+L	30.3	1.63	1.07E+00		
G7		H2O	37.1	0.00	3.21E-02	37.1	

This report generated by Rotor-Gene Real-Time Analysis Software 6.1 (Build 93)
© Corbett Research 2005
® All Rights Reserved
ISO 9001:2000 (Reg. No. QEC21313)
