# Supplementary material for: Epigenetically silenced apoptosis-associated tyrosine kinase (AATK) facilitates a decreased expression of Cyclin D1 and WEE1, phosphorylates TP53 and reduces cell proliferation in a kinase-dependent manner
Source: Cancer Gene Ther. 2022 Jul 28;29(12):1975–87. doi: 10.1038/s41417-022-00513-x (PMC9750878; doi:10.1038/s41417-022-00513-x)
Supplement: Supplementary file 6 — Dataset original qPCR [file 41417_2022_513_MOESM6_ESM.zip › WEE1_clone pools.pdf]

corbett RESEARCH
A Division of Corbett Life Science

14 Hilly Street Mortlake NSW 2137 Australia
T +61 2 9736 1320
F +61 2 9736 1364
W www.corbettlifescience.com

Comparative Quantitation Report

Experiment Information

Run Name	Run 2020-10-05_WEE1_Affy cDNA_HeLa OE_b-Act_HeLa OE
Run Start	05.10.2020 12:06:54
Run Finish	05.10.2020 13:54:52
Operator	MW
Notes	Wee1 Affy cDNA (1ul); HeLa OE (1ul) b-Act HeLa OE triplicate
Run On Software Version	Rotor-Gene 6.1.93
Run Signature	The Run Signature is valid.
Gain FAM	8.
Gain ROX	9.33

Comparative Quantitation Information

Reaction Amplification	1.68
Reaction Amplification Std. Deviation	0.03
Sample Page	Page 1
Control Replicate	(1)

Take off Graph for Cycling A.FAM

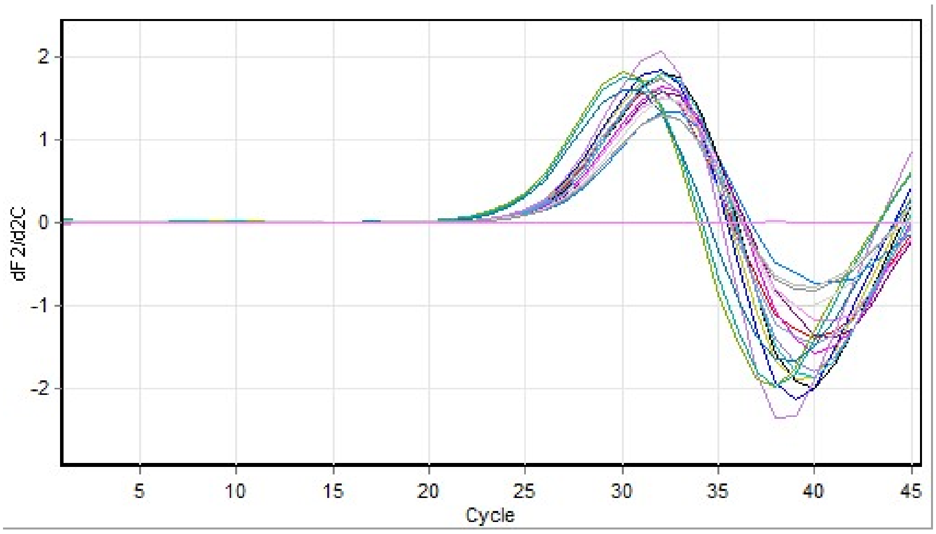

No.	Colour	Name	Take Off	Amplification	Comparative Conc.	Rep. Takeoff	Rep. Takeoff (95% CI)
A1		Control clone pool (1)	27.2	1.71	9.66E-01	27.1	[1.$,1.$]
A2		Control clone pool (1)	27.1	1.70	1.02E+00		
A3		Control clone pool (1)	27.1	1.66	1.02E+00		
A4		Control clone pool (2)	27.7	1.68	7.45E-01	27.7	[1.$,1.$]
A5		Control clone pool (2)	27.5	1.68	8.27E-01		
A6		Control clone pool (2)	27.8	1.66	7.08E-01		
B2		Clone pool AATK (1)	27.6	1.66	7.85E-01	27.6	[1.$,1.$]
B3		Clone pool AATK (1)	27.5	1.68	8.27E-01		
B4		Clone pool AATK (1)	27.7	1.70	7.45E-01		
B8		Clone pool AATK KD (1)	27.0	1.71	1.07E+00	27.2	[1.$,1.$]
C1		Clone pool AATK KD (1)	27.3	1.70	9.17E-01		
C2		Clone pool AATK KD (1)	27.3	1.67	9.17E-01		
C6		Clone pool AATK (2)	25.6	1.66	2.22E+00	25.7	[1.$,1.$]
C7		Clone pool AATK (2)	25.7	1.71	2.10E+00		
C8		Clone pool AATK (2)	25.7	1.68	2.10E+00		
D4		Clone pool AATK KD (2)	27.7	1.66	7.45E-01	27.6	[1.$,1.$]
D5		Clone pool AATK KD (2)	27.5	1.61	8.27E-01		
D6		Clone pool AATK KD (2)	27.6	1.70	7.85E-01		
I5		H2O	20.1	0.79	3.84E+01	20.1	

Quality System ™
Quality Endorsed Company
ISO 9001 Lic 21313
SAI Global

This report generated by Rotor-Gene Real-Time Analysis Software 6.1 (Build 93)
© Corbett Research 2005
® All Rights Reserved
ISO 9001:2000 (Reg. No. QEC21313)
