# Supplementary material for: Epigenetically silenced apoptosis-associated tyrosine kinase (AATK) facilitates a decreased expression of Cyclin D1 and WEE1, phosphorylates TP53 and reduces cell proliferation in a kinase-dependent manner
Source: Cancer Gene Ther. 2022 Jul 28;29(12):1975–87. doi: 10.1038/s41417-022-00513-x (PMC9750878; doi:10.1038/s41417-022-00513-x)
Supplement: Supplementary file 6 — Dataset original qPCR [file 41417_2022_513_MOESM6_ESM.zip › GAPDH_Capan1.pdf]

corbett RESEARCH
A Division of Corbett Life Science

14 Hilly Street Mortlake NSW 2137 Australia
T +61 2 9736 1320
F +61 2 9736 1364
W www.corbettlifescience.com

Comparative Quantitation Report

Experiment Information

Run Name	Run 2016-12-21_GAPDH_Aza_Pankreas
Run Start	21.12.2016 17:01:12
Run Finish	21.12.2016 18:13:45
Operator	MW
Notes	GAPDH Aza Pankeas triplicate
Run On Software Version	Rotor-Gene 6.1.93
Run Signature	The Run Signature is valid.
Gain FAM	8.
Gain ROX	8.

Comparative Quantitation Information

Reaction Amplification	1.77
Reaction Amplification Std. Deviation	0.03
Sample Page	Page 1
Control Replicate	(1)

Take off Graph for Cycling A.FAM/Cycling A.ROX

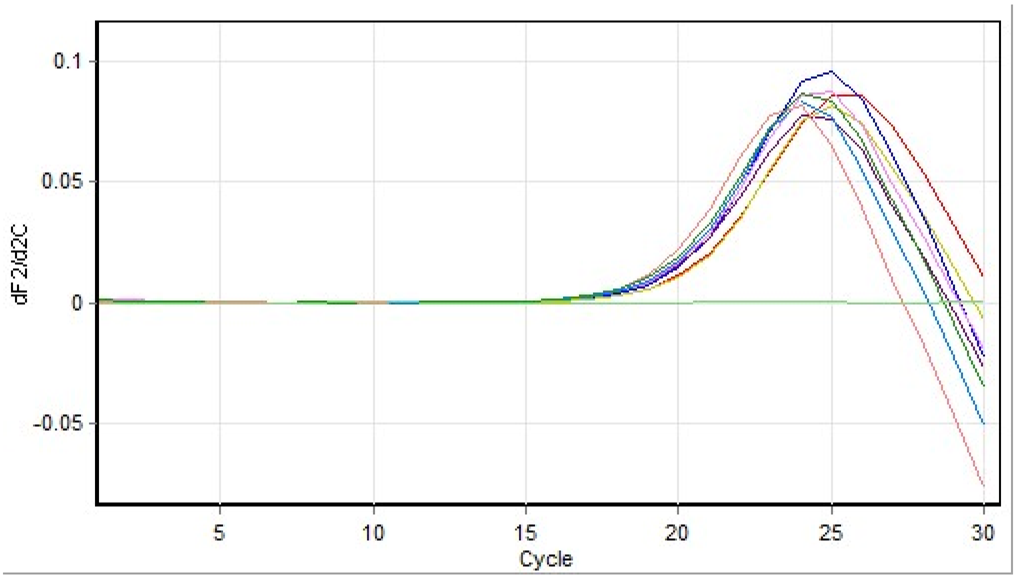

No.	Colour	Name	Take Off	Amplification	Comparative Conc.	Rep. Takeoff	Rep. Takeoff (95% CI)
A1		Capan1 0uM	21.3	1.76	9.44E-01	21.2	[1.$,1.$]
A2		Capan1 0uM	21.3	1.78	9.44E-01		
A3		Capan1 0uM	21.0	1.82	1.12E+00		
A4		Capan1 5uM	20.7	1.73	1.33E+00	20.7	[1.$,1.$]
A5		Capan1 5uM	20.9	1.75	1.19E+00		
A6		Capan1 5uM	20.6	1.77	1.41E+00		
A7		Capan1 10uM	20.2	1.77	1.77E+00	20.3	[1.$,1.$]
A8		Capan1 10uM	20.2	1.82	1.77E+00		
B1		Capan1 10uM	20.5	1.74	1.49E+00		
F6		H2O	23.8	0.00	2.26E-01	23.8	

This report generated by Rotor-Gene Real-Time Analysis Software 6.1 (Build 93)
© Corbett Research 2005
® All Rights Reserved
ISO 9001:2000 (Reg. No. QEC21313)

Quality System Quality Endorsed Company ISO 9001 Lic 21313 SAI Global
