# Supplementary material for: Epigenetically silenced apoptosis-associated tyrosine kinase (AATK) facilitates a decreased expression of Cyclin D1 and WEE1, phosphorylates TP53 and reduces cell proliferation in a kinase-dependent manner
Source: Cancer Gene Ther. 2022 Jul 28;29(12):1975–87. doi: 10.1038/s41417-022-00513-x (PMC9750878; doi:10.1038/s41417-022-00513-x)
Supplement: Supplementary file 6 — Dataset original qPCR [file 41417_2022_513_MOESM6_ESM.zip › U343_GAPDH.pdf]

14 Hilly Street Mortlake NSW 2137 Australia
T +61 2 9736 1320
F +61 2 9736 1364
W www.corbettlifescience.com

Comparative Quantitation Report

Experiment Information

Run Name	Run 2021-03-17_GAPDH_OE-EY_U343_U251_A549_A427
Run Start	17.03.2021 08:52:02
Run Finish	17.03.2021 10:17:21
Operator	MW
Notes	GAPDH OE U343 U251 A549 A427 triplicate
Run On Software Version	Rotor-Gene 6.1.93
Run Signature	The Run Signature is valid.
Gain FAM	8.
Gain ROX	9.33

Comparative Quantitation Information

Reaction Amplification	1.63
Reaction Amplification Std. Deviation	0.02
Sample Page	Page 1
Control Replicate	(1)

Take off Graph for Cycling A.FAM/Cycling A.ROX

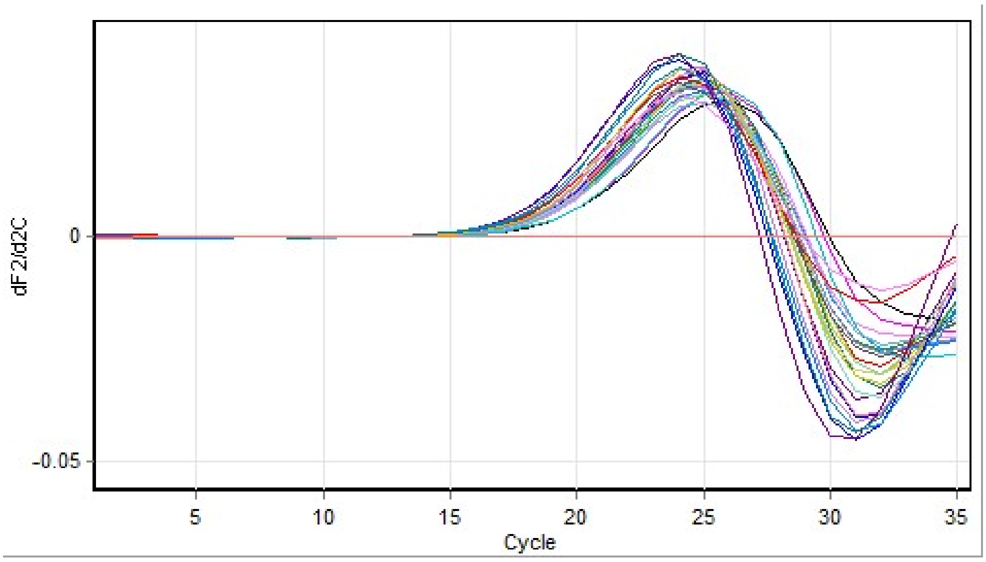

No.	Colour	Name	Take Off	Amplification	Comparative Conc.	Rep. Takeoff	Rep. Takeoff (95% CI)
A1		U343 EY (1)	20.0	1.62	1.03E+00	20.1	[1.$,1.$]
A2		U343 EY (1)	20.2	1.62	9.37E-01		
A3		U343 EY (1)	20.0	1.65	1.03E+00		
A4		U343 B-EY (1)	19.9	1.65	1.08E+00	20.1	[1.$,1.$]
A5		U343 B-EY (1)	20.2	1.59	9.37E-01		
A6		U343 B-EY (1)	20.1	1.61	9.84E-01		
A7		U343 B KD-EY (1)	20.1	1.65	9.84E-01	20.1	[1.$,1.$]
A8		U343 B KD-EY (1)	20.2	1.62	9.37E-01		
B1		U343 B KD-EY (1)	20.0	1.64	1.03E+00		
B2		U343 EY (2)	20.8	1.66	7.00E-01	20.7	[1.$,1.$]
B3		U343 EY (2)	20.6	1.64	7.71E-01		
B4		U343 EY (2)	20.8	1.62	7.00E-01		
B5		U343 B-EY (2)	19.9	1.63	1.08E+00	20.0	[1.$,1.$]
B6		U343 B-EY (2)	20.0	1.62	1.03E+00		
B7		U343 B-EY (2)	20.2	1.59	9.37E-01		
B8		U343 B KD-EY (2)	19.8	1.62	1.14E+00	19.8	[1.$,1.$]
C1		U343 B KD-EY (2)	19.7	1.66	1.20E+00		
C2		U343 B KD-EY (2)	19.8	1.63	1.14E+00		

(Continued on next page)...

No.	Colour	Name	Take Off	Amplification	Comparative Conc.	Rep. Takeoff	Rep. Takeoff (95% CI)
H8		U343 EY (3)	19.3	1.63	1.45E+00	19.5	[1.$,1.$]
I1		U343 EY (3)	19.4	1.63	1.38E+00		
I2		U343 EY (3)	19.7	1.65	1.20E+00		
I3		U343 B-EY (3)	19.1	1.64	1.60E+00	19.1	[1.$,1.$]
I4		U343 B-EY (3)	19.2	1.64	1.52E+00		

I5		U343 B-EY (3)	19.1	1.56	1.60E+00		
I6		U343 B KD-EY (3)	19.2	1.60	1.52E+00	19.4	
I7		U343 B KD-EY (3)	19.6	1.64	1.25E+00		
I8		H2O	24.0	0.00	1.48E-01	24.0	

This report generated by Rotor-Gene Real-Time Analysis Software 6.1 (Build 93)
© Corbett Research 2005
® All Rights Reserved
ISO 9001:2000 (Reg. No. QEC21313)

Quality System
Quality Endorsed Company
ISO 9001 Lic 21313
SAI Global
