# Supplementary material for: Epigenetically silenced apoptosis-associated tyrosine kinase (AATK) facilitates a decreased expression of Cyclin D1 and WEE1, phosphorylates TP53 and reduces cell proliferation in a kinase-dependent manner
Source: Cancer Gene Ther. 2022 Jul 28;29(12):1975–87. doi: 10.1038/s41417-022-00513-x (PMC9750878; doi:10.1038/s41417-022-00513-x)
Supplement: Supplementary file 6 — Dataset original qPCR [file 41417_2022_513_MOESM6_ESM.zip › CCND1_clone pools.pdf]

corbett RESEARCH
A Division of Corbett Life Science

14 Hilly Street Mortlake NSW 2137 Australia
T +61 2 9736 1320
F +61 2 9736 1364
W www.corbettlifescience.com

Comparative Quantitation Report

Experiment Information

Run Name	Run 2019-06-18_Affy_Nova1_CCND1
Run Start	18.06.2019 09:54:43
Run Finish	18.06.2019 11:50:05
Operator	MW
Notes	Affy cDNA Nova1 Ccnd1 Triplicate
Run On Software Version	Rotor-Gene 6.1.93
Run Signature	The Run Signature is valid.
Gain FAM	8.
Gain ROX	9.33

Comparative Quantitation Information

Reaction Amplification	1.63
Reaction Amplification Std. Deviation	0.03
Sample Page	Page 1
Control Replicate	(32)

Take off Graph for Cycling A.FAM

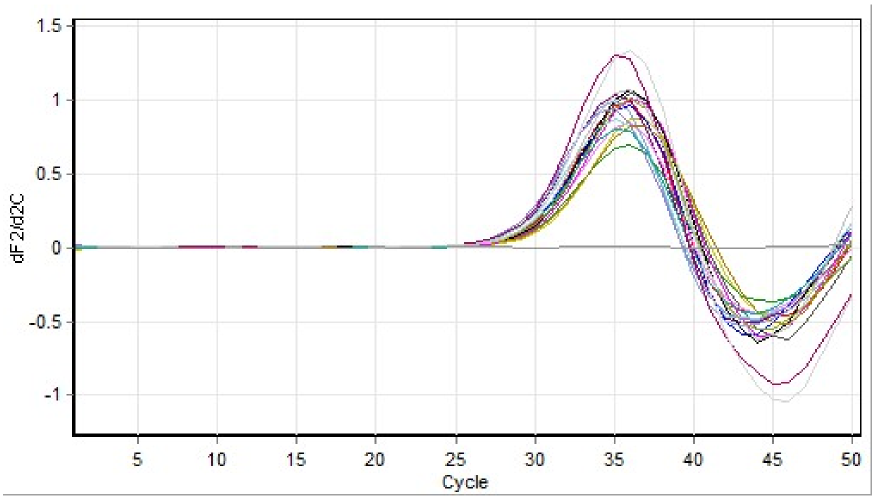

No.	Colour	Name	Take Off	Amplification	Comparative Conc.	Rep. Takeoff	Rep. Takeoff (95% CI)
D8		Control clone pool (1)_AATK	31.2	1.64	1.02E+00	31.2	[1.$,1.$]
E1		Control clone pool (1)_AATK	30.9	1.68	1.18E+00		
E2		Control clone pool (1)_AATK	31.6	1.64	8.36E-01		
E3		Control clone pool (2)_AATK	30.7	1.59	1.30E+00	30.7	[1.$,1.$]
E4		Control clone pool (2)_AATK	30.3	1.66	1.58E+00		
E5		Control clone pool (2)_AATK	31.0	1.63	1.12E+00		
F1		Clone pool AATK (1) _AATK	30.9	1.62	1.18E+00	31.1	[1.$,1.$]
F2		Clone pool AATK (1) _AATK	31.3	1.63	9.68E-01		
F3		Clone pool AATK (1) _AATK	31.2	1.61	1.02E+00		
F7		Clone pool AATK KD (1) _AATK	30.1	1.59	1.74E+00	30.0	[1.$,1.$]
F8		Clone pool AATK KD (1) _AATK	30.1	1.62	1.74E+00		
G1		Clone pool AATK KD (1) _AATK	29.9	1.66	1.91E+00		
G5		Clone pool AATK (2) _AATK	31.4	1.60	9.22E-01	30.9	[1.$,1.$]
G6		Clone pool AATK (2) _AATK	31.0	1.59	1.12E+00		
G7		Clone pool AATK (2) _AATK	30.4	1.62	1.50E+00		
H3		Clone pool AATK KD (2) _AATK	30.7	1.63	1.30E+00	30.8	[1.$,1.$]
H4		Clone pool AATK KD (2) _AATK	31.0	1.65	1.12E+00		
H5		Clone pool AATK KD (2) _AATK	30.8	1.65	1.24E+00		
H6		H2O CCND1	10.9	0.46	2.00E+04	10.9	

Quality System ™ Quality Endorsed Company ISO 9001 Lic 21313 SAI Global

This report generated by Rotor-Gene Real-Time Analysis Software 6.1 (Build 93)
© Corbett Research 2005
® All Rights Reserved
ISO 9001:2000 (Reg. No. QEC21313)
