# Supplementary material for: Epigenetically silenced apoptosis-associated tyrosine kinase (AATK) facilitates a decreased expression of Cyclin D1 and WEE1, phosphorylates TP53 and reduces cell proliferation in a kinase-dependent manner
Source: Cancer Gene Ther. 2022 Jul 28;29(12):1975–87. doi: 10.1038/s41417-022-00513-x (PMC9750878; doi:10.1038/s41417-022-00513-x)
Supplement: Supplementary file 6 — Dataset original qPCR [file 41417_2022_513_MOESM6_ESM.zip › Glioblastoma_AATK.pdf]

corbett RESEARCH
A Division of Corbett Life Science

14 Hilly Street Mortlake NSW 2137 Australia
T +61 2 9736 1320
F +61 2 9736 1364
W www.corbettlifescience.com

Comparative Quantitation Report

Experiment Information

Run Name	Run 2019-01-03_AATK_Glio-CL
Run Start	03.01.2019 11:34:12
Run Finish	03.01.2019 13:25:50
Operator	MW
Notes	AATK Glio-CL triplicate
Run On Software Version	Rotor-Gene 6.1.93
Run Signature	The Run Signature is valid.
Gain FAM	8.
Gain ROX	8.

Comparative Quantitation Information

Reaction Amplification	0.88
Reaction Amplification Std. Deviation	0.71
Sample Page	Page 1
Control Replicate	(1)

Take off Graph for Cycling A.FAM

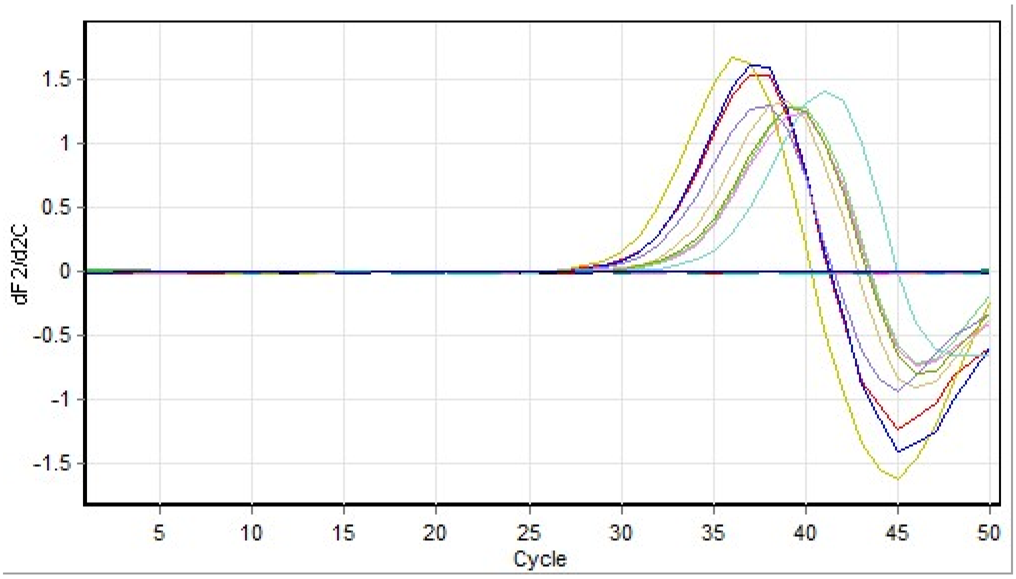

No.	Colour	Name	Take Off	Amplification	Comparative Conc.	Rep. Takeoff	Rep. Takeoff (95% CI)
A1		LN229	32.8	1.69	1.04E+00	32.5	[1.$,1.$]
A2		LN229	31.9	1.70	9.21E-01		
A3		LN229	32.9	1.72	1.05E+00		
A4		U343	26.3	-0.09	4.43E-01	33.7	[1.$,1.$]
A5		U343	34.8	1.67	1.34E+00		
A6		U343	40.1	0.63	2.68E+00		
A7		U118	23.3	0.21	3.00E-01	26.2	[1.$,1.$]
A8		U118	15.1	0.17	1.03E-01		
B1		U118	40.3	0.74	2.76E+00		
B2		U87MG	23.1	0.21	2.92E-01	19.5	[1.$,1.$]
B3		U87MG	29.3	0.52	6.56E-01		
B4		U87MG	6.0	0.17	3.13E-02		
B5		T98G	34.1	1.68	1.23E+00	35.1	[1.$,1.$]
B6		T98G	34.8	1.68	1.34E+00		
B7		T98G	36.5	1.68	1.68E+00		
B8		A172	36.1	-0.02	1.59E+00	31.4	[1.$,1.$]
C1		A172	33.0	1.67	1.06E+00		
C2		A172	25.1	1.56	3.79E-01		
C3		A764	9.4	0.35	4.88E-02	14.6	[1.$,1.$]
C4		A764	21.4	0.74	2.34E-01		
C5		A764	13.1	0.60	7.92E-02		
C6		SNB19	34.6	1.68	1.31E+00	37.6	[1.$,1.$]

(Continued on next page)...

C7		SNB19	40.3	0.17	2.76E+00		
C8		SNB19	37.9	-0.10	2.01E+00		
D1		H2O	21.6	0.91	2.40E-01	21.6	

This report generated by Rotor-Gene Real-Time Analysis Software 6.1 (Build 93)
© Corbett Research 2005
® All Rights Reserved
ISO 9001:2000 (Reg. No. QEC21313)

Quality System
Quality Endorsed Company
ISO 9001 Lic 21313
SAI Global
