# Supplementary material for: Epigenetically silenced apoptosis-associated tyrosine kinase (AATK) facilitates a decreased expression of Cyclin D1 and WEE1, phosphorylates TP53 and reduces cell proliferation in a kinase-dependent manner
Source: Cancer Gene Ther. 2022 Jul 28;29(12):1975–87. doi: 10.1038/s41417-022-00513-x (PMC9750878; doi:10.1038/s41417-022-00513-x)
Supplement: Supplementary file 6 — Dataset original qPCR [file 41417_2022_513_MOESM6_ESM.zip › Epigen.edit_GAPDH_1.pdf]

14 Hilly Street Mortlake NSW 2137 Australia
T + 61 2 9736 1320
F + 61 2 9736 1364
W www.corbettlifescience.com

Comparative Quantitation Report

Experiment Information

Run Name	Run 2019-02-28_GAPDH_HEK_1.Epig.
Run Start	27.02.2019 20:07:27
Run Finish	27.02.2019 22:01:26
Operator	MW
Notes	GAPDH 1. Epig. HEK triplicate
Run On Software Version	Rotor-Gene 6.1.93
Run Signature	The Run Signature is valid.
Gain FAM	8.
Gain ROX	9.33

Comparative Quantitation Information

Reaction Amplification	1.60
Reaction Amplification Std. Deviation	0.03
Sample Page	Page 1
Control Replicate	(4)

Take off Graph for Cycling A.FAM

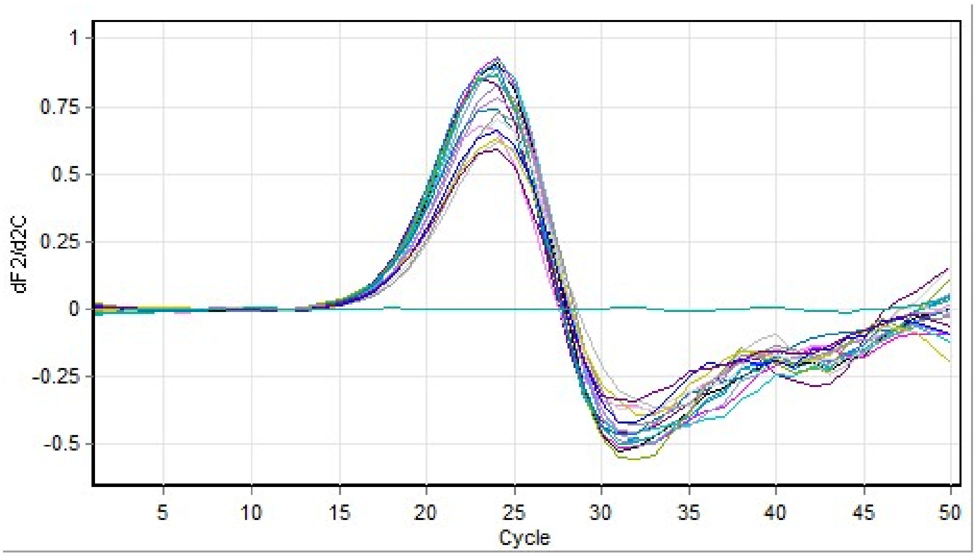

No.	Colour	Name	Take Off	Amplification	Comparative Conc.	Rep. Takeoff	Rep. Takeoff (95% CI)
A4		Oligo Mix pcDNA	18.4	1.57	1.15E+00	18.7	[1.$,1.$]
A5		Oligo Mix pcDNA	18.2	1.65	1.27E+00		
A6		Oligo Mix pcDNA	18.6	1.62	1.05E+00		
B2		Oligo Mix p300	18.9	1.63	9.10E-01	18.9	[1.$,1.$]
B3		Oligo Mix p300	18.8	1.64	9.54E-01		
B4		Oligo Mix p300	18.9	1.60	9.10E-01		
B8		Oligo Mix pcDNA	19.1	1.59	8.28E-01		
C1		Oligo Mix pcDNA	19.0	1.59	8.68E-01		
C2		Oligo Mix pcDNA	18.9	1.62	9.10E-01		
C6		Oligo Mix EZH2	18.7	1.63	1.00E+00	18.6	[1.$,1.$]
C7		Oligo Mix EZH2	18.7	1.61	1.00E+00		
C8		Oligo Mix EZH2	18.4	1.61	1.15E+00		
D4		Oligo Mix pcDNA3	19.1	1.60	8.28E-01	19.3	[1.$,1.$]
D5		Oligo Mix pcDNA3	19.2	1.57	7.90E-01		
D6		Oligo Mix pcDNA3	19.5	1.59	6.86E-01		
E2		Oligo Mix DNMT3A	18.6	1.56	1.05E+00	18.6	[1.$,1.$]
E3		Oligo Mix DNMT3A	18.7	1.57	1.00E+00		
E4		Oligo Mix DNMT3A	18.5	1.54	1.10E+00		
G7		H20	18.1	-0.29	1.33E+00	18.1	

This report generated by Rotor-Gene Real-Time Analysis Software 6.1 (Build 93)
© Corbett Research 2005
® All Rights Reserved
ISO 9001:2000 (Reg. No. QEC21313)
