# Supplementary material for: Epigenetically silenced apoptosis-associated tyrosine kinase (AATK) facilitates a decreased expression of Cyclin D1 and WEE1, phosphorylates TP53 and reduces cell proliferation in a kinase-dependent manner
Source: Cancer Gene Ther. 2022 Jul 28;29(12):1975–87. doi: 10.1038/s41417-022-00513-x (PMC9750878; doi:10.1038/s41417-022-00513-x)
Supplement: Supplementary file 6 — Dataset original qPCR [file 41417_2022_513_MOESM6_ESM.zip › U343_WEE1.pdf]

14 Hilly Street Mortlake NSW 2137 Australia
T +61 2 9736 1320
F +61 2 9736 1364
W www.corbettlifescience.com

Comparative Quantitation Report

Experiment Information

Run Name	Run 2020-10-16_WEE1_OE-U343_U251_A549_A427_(1)(2)
Run Start	16.10.2020 09:19:03
Run Finish	16.10.2020 11:04:07
Operator	MW
Notes	Wee1 OE u343 u251 a549 a427 (1)(2) triplicate
Run On Software Version	Rotor-Gene 6.1.93
Run Signature	The Run Signature is valid.
Gain FAM	8.
Gain ROX	8.

Comparative Quantitation Information

Reaction Amplification	1.65
Reaction Amplification Std. Deviation	0.03
Sample Page	Page 1
Control Replicate	(1)

Take off Graph for Cycling A.FAM/Cycling A.ROX

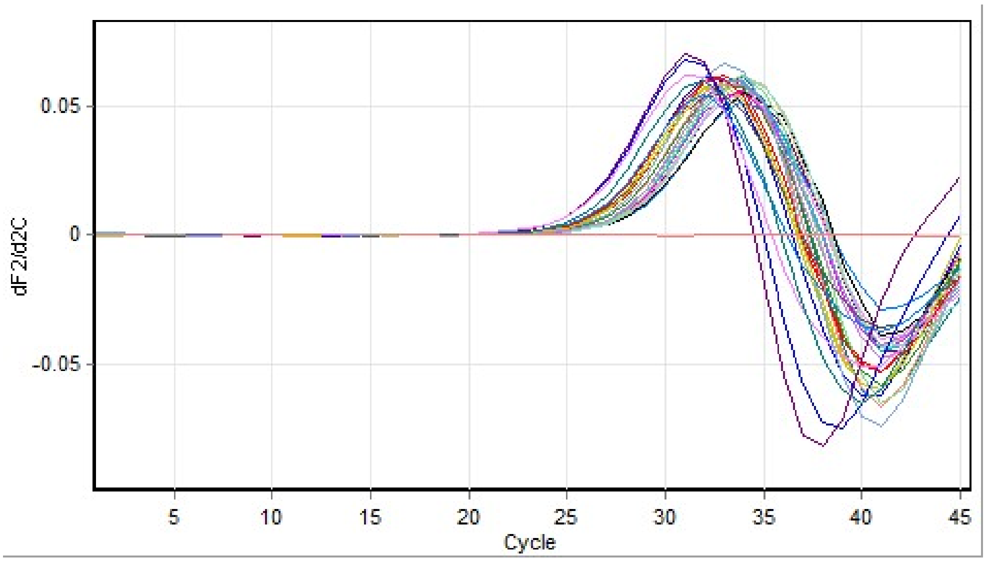

No.	Colour	Name	Take Off	Amplification	Comparative Conc.	Rep. Takeoff	Rep. Takeoff (95% CI)
A1		U343 EY (1)	28.3	1.61	8.61E-01	28.0	[1.$,1.$]
A2		U343 EY (1)	27.9	1.65	1.05E+00		
A3		U343 EY (1)	27.8	1.67	1.11E+00		
A4		U343 B-EY (1)	29.2	1.60	5.48E-01	29.3	[1.$,1.$]
A5		U343 B-EY (1)	29.0	1.62	6.06E-01		
A6		U343 B-EY (1)	29.6	1.64	4.49E-01		
A7		U343 B KD-EY (1)	28.8	1.67	6.70E-01	28.7	[1.$,1.$]
A8		U343 B KD-EY (1)	28.6	1.66	7.41E-01		
B1		U343 B KD-EY (1)	28.6	1.64	7.41E-01		
B2		U343 EY (2)	29.1	1.72	5.77E-01	29.2	[1.$,1.$]
B3		U343 EY (2)	29.5	1.62	4.72E-01		
B4		U343 EY (2)	29.0	1.70	6.06E-01		
B5		U343 B-EY (2)	29.4	1.62	4.96E-01	29.4	[1.$,1.$]
B6		U343 B-EY (2)	29.2	1.67	5.48E-01		
B7		U343 B-EY (2)	29.5	1.66	4.72E-01		
B8		U343 B KD-EY (2)	28.5	1.65	7.79E-01	28.7	[1.$,1.$]
C1		U343 B KD-EY (2)	28.9	1.64	6.37E-01		
C2		U343 B KD-EY (2)	28.8	1.64	6.70E-01		

(Continued on next page)...

No.	Colour	Name	Take Off	Amplification	Comparative Conc.	Rep. Takeoff	Rep. Takeoff (95% CI)
H8		U343 B-EY (3)	27.7	1.63	1.16E+00	27.7	
I1		U343 B-EY (3)	27.7	1.66	1.16E+00		
I2		U343 EY (3)	28.1	1.63	9.51E-01	27.2	[1.$,1.$]
I3		U343 EY (3)	26.8	1.64	1.82E+00		
I4		U343 EY (3)	26.8	1.67	1.82E+00		

I5		U343 B KD-EY (3)	26.7	1.66	1.92E+00	27.1	[1.$,1.$]
I6		U343 B KD-EY (3)	27.4	1.66	1.35E+00		
I7		U343 B KD-EY (3)	27.2	1.67	1.49E+00		
I8		H2O	24.3	0.00	6.37E+00	24.3	

Quality System
Quality Endorsed Company
ISO 9001 Lic 21313
SAI Global

This report generated by Rotor-Gene Real-Time Analysis Software 6.1 (Build 93)
© Corbett Research 2005
® All Rights Reserved
ISO 9001:2000 (Reg. No. QEC21313)
