# Supplementary material for: Epigenetically silenced apoptosis-associated tyrosine kinase (AATK) facilitates a decreased expression of Cyclin D1 and WEE1, phosphorylates TP53 and reduces cell proliferation in a kinase-dependent manner
Source: Cancer Gene Ther. 2022 Jul 28;29(12):1975–87. doi: 10.1038/s41417-022-00513-x (PMC9750878; doi:10.1038/s41417-022-00513-x)
Supplement: Supplementary file 6 — Dataset original qPCR [file 41417_2022_513_MOESM6_ESM.zip › Epigen.edit_GAPDH_3.pdf]

corbett
RESEARCH
A Division of Corbett Life Science

14 Hilly Street Mortlake NSW 2137 Australia
T +61 2 9736 1320
F +61 2 9736 1364
W www.corbettlifescience.com

Comparative Quantitation Report

Experiment Information

Run Name	Run 2019-03-06_GAPDH_HEK_3.Epig.
Run Start	06.03.2019 14:56:52
Run Finish	06.03.2019 16:51:14
Operator	MW
Notes	GAPDH 3. Epig. HEK triplicate
Run On Software Version	Rotor-Gene 6.1.93
Run Signature	The Run Signature is valid.
Gain FAM	8.
Gain ROX	9.33

Comparative Quantitation Information

Reaction Amplification	1.46
Reaction Amplification Std. Deviation	0.15
Sample Page	Page 1
Control Replicate	(1)

Take off Graph for Cycling A.FAM/Cycling A.ROX

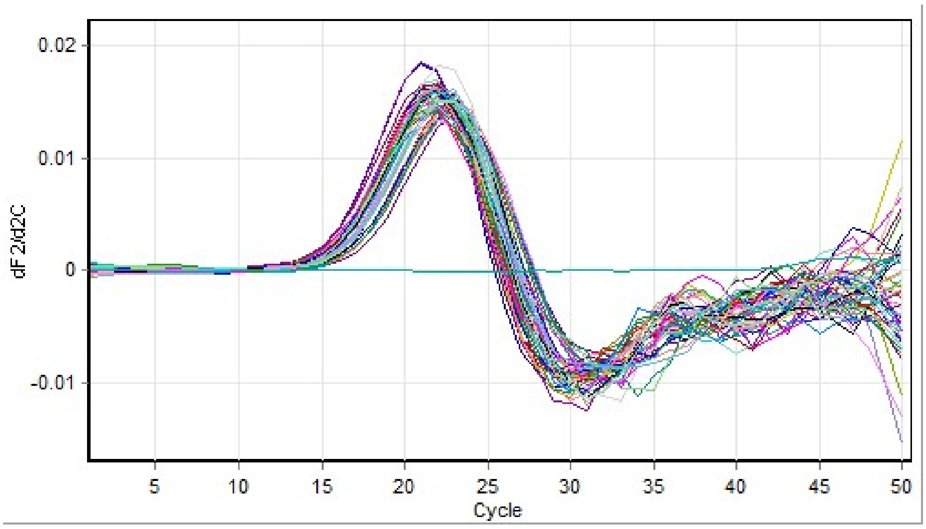

No.	Colour	Name	Take Off	Amplification	Comparative Conc.	Rep. Takeoff	Rep. Takeoff (95% CI)
A1		px459dCas9 pcDNA 1	17.8	1.52	1.03E+00	17.9	[1.$,1.$]
A2		px459dCas9 pcDNA 1	18.0	1.58	9.51E-01		
A3		px459dCas9 pcDNA 1	17.8	1.52	1.03E+00		
A4		Oligo Mix pcDNA 1	18.5	1.47	7.88E-01	18.3	[1.$,1.$]
A5		Oligo Mix pcDNA 1	18.2	1.24	8.82E-01		
A6		Oligo Mix pcDNA 1	18.1	1.29	9.16E-01		
A7		px459dCas9 p300	18.0	1.23	9.51E-01	18.1	[1.$,1.$]
A8		px459dCas9 p300	18.2	1.27	8.82E-01		
B1		px459dCas9 p300	18.1	1.20	9.16E-01		
B2		Oligo Mix p300	17.6	1.53	1.11E+00	17.6	[1.$,1.$]
B3		Oligo Mix p300	17.9	1.60	9.88E-01		
B4		Oligo Mix p300	17.4	1.37	1.19E+00		
B5		px459dCas9 pcDNA 2	16.9	1.59	1.44E+00	16.9	[1.$,1.$]
B6		px459dCas9 pcDNA 2	16.8	1.54	1.49E+00		
B7		px459dCas9 pcDNA 2	17.0	1.63	1.38E+00		
B8		Oligo Mix pcDNA 2	17.5	1.44	1.15E+00	17.4	[1.$,1.$]
C1		Oligo Mix pcDNA 2	17.4	1.42	1.19E+00		
C2		Oligo Mix pcDNA 2	17.4	1.33	1.19E+00		

(Continued on next page)...

No.	Colour	Name	Take Off	Amplification	Comparative Conc.	Rep. Takeoff	Rep. Takeoff (95% CI)
C3		px459dCas9 EZH2	17.1	1.63	1.33E+00	16.9	[1.$,1.$]
C4		px459dCas9 EZH2	16.8	1.52	1.49E+00		
C5		px459dCas9 EZH2	16.8	1.51	1.49E+00		
C6		Oligo Mix EZH2	17.1	1.64	1.33E+00	17.2	[1.$,1.$]
C7		Oligo Mix EZH2	17.3	1.30	1.24E+00		
C8		Oligo Mix EZH2	17.2	1.30	1.28E+00		
D1		px459dCas9 pcDNA 3	16.6	1.45	1.61E+00	16.6	[1.$,1.$]
D2		px459dCas9 pcDNA 3	16.7	1.55	1.55E+00		

D3		px459dCas9 pcDNA 3	16.5	1.41	1.67E+00		
D4		Oligo Mix pcDNA3	17.6	1.60	1.11E+00	17.3	[1.$,1.$]
D5		Oligo Mix pcDNA3	17.0	1.18	1.38E+00		
D6		Oligo Mix pcDNA3	17.4	1.69	1.19E+00		
D7		px459 dCas9 DNMt3A	16.9	1.60	1.44E+00	16.9	[1.$,1.$]
D8		px459 dCas9 DNMt3A	16.9	1.55	1.44E+00		
E1		px459 dCas9 DNMt3A	16.9	1.55	1.44E+00		
E2		Oligo Mix DNMT3A	16.7	1.53	1.55E+00	16.8	[1.$,1.$]
E3		Oligo Mix DNMT3A	16.9	1.57	1.44E+00		
E4		Oligo Mix DNMT3A	16.7	1.56	1.55E+00		
E5		px459dCas9 pcDNA3	17.1	1.26	1.33E+00	17.0	[1.$,1.$]
E6		px459dCas9 pcDNA3	16.9	1.20	1.44E+00		
E7		px459dCas9 pcDNA3	16.9	1.17	1.44E+00		
E8		Oligo Mix pcDNA	16.6	1.44	1.61E+00	16.4	[1.$,1.$]
F1		Oligo Mix pcDNA	16.3	1.29	1.80E+00		
F2		Oligo Mix pcDNA	16.4	1.34	1.73E+00		
F3		px459dCas9 DNMT3A+L	17.1	1.64	1.33E+00	17.0	[1.$,1.$]
F4		px459dCas9 DNMT3A+L	16.9	1.61	1.44E+00		
F5		px459dCas9 DNMT3A+L	16.9	1.53	1.44E+00		
F6		Oligo Mix DNMT3A+L	17.5	1.46	1.15E+00	17.6	[1.$,1.$]
F7		Oligo Mix DNMT3A+L	17.7	1.54	1.06E+00		
F8		Oligo Mix DNMT3A+L	17.7	1.48	1.06E+00		
G7		H2O	42.9	0.00	8.24E-05	42.9	

Quality System ™
Quality Endorsed Company
ISO 9001 Lic 21313
SAI Global
This report generated by Rotor-Gene Real-Time Analysis Software 6.1 (Build 93)
© Corbett Research 2005
® All Rights Reserved
ISO 9001:2000 (Reg. No. QEC21313)
