# Supplementary material for: Epigenetically silenced apoptosis-associated tyrosine kinase (AATK) facilitates a decreased expression of Cyclin D1 and WEE1, phosphorylates TP53 and reduces cell proliferation in a kinase-dependent manner
Source: Cancer Gene Ther. 2022 Jul 28;29(12):1975–87. doi: 10.1038/s41417-022-00513-x (PMC9750878; doi:10.1038/s41417-022-00513-x)
Supplement: Supplementary file 6 — Dataset original qPCR [file 41417_2022_513_MOESM6_ESM.zip › Epigen.edit_GAPDH_2.pdf]

corbett RESEARCH
A Division of Corbett Life Science

14 Hilly Street Mortlake NSW 2137 Australia
T +61 2 9736 1320
F +61 2 9736 1364
W www.corbettlifescience.com

Comparative Quantitation Report

Experiment Information

Run Name	Run 2019-03-06_GAPDH_HEK_2.Epig.
Run Start	06.03.2019 09:37:05
Run Finish	06.03.2019 11:29:27
Operator	MW
Notes	GAPDH 2. Epig. HEK triplicate
Run On Software Version	Rotor-Gene 6.1.93
Run Signature	The Run Signature is valid.
Gain FAM	8.
Gain ROX	8.

Comparative Quantitation Information

Reaction Amplification	1.60
Reaction Amplification Std. Deviation	0.03
Sample Page	Page 1
Control Replicate	(1)

Take off Graph for Cycling A.FAM/Cycling A.ROX

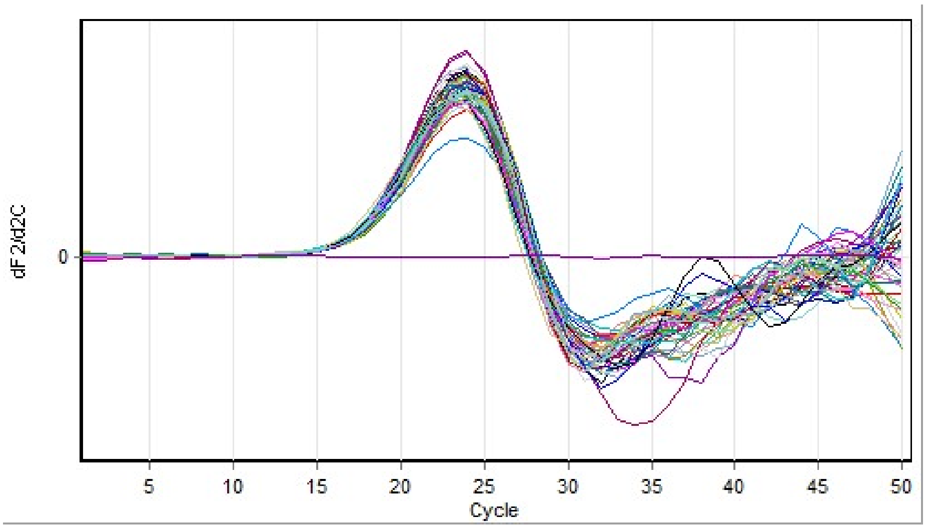

No.	Colour	Name	Take Off	Amplification	Comparative Conc.	Rep. Takeoff	Rep. Takeoff (95% CI)
A1		px459dCas9 pcDNA	18.8	1.62	1.02E+00	18.8	[1.$,1.$]
A2		px459dCas9 pcDNA	18.9	1.59	9.69E-01		
A3		px459dCas9 pcDNA	18.7	1.63	1.06E+00		
A4		Oligo Mix pcDNA	18.7	1.60	1.06E+00	18.7	[1.$,1.$]
A5		Oligo Mix pcDNA	18.8	1.59	1.02E+00		
A6		Oligo Mix pcDNA	18.7	1.60	1.06E+00		
A7		px459dCas9 p300	19.2	1.64	8.42E-01	19.3	[1.$,1.$]
A8		px459dCas9 p300	19.3	1.55	8.04E-01		
B1		px459dCas9 p300	19.4	1.60	7.67E-01		
B2		Oligo Mix p300	18.9	1.60	9.69E-01	19.0	[1.$,1.$]
B3		Oligo Mix p300	19.0	1.57	9.25E-01		
B4		Oligo Mix p300	19.1	1.58	8.83E-01		
B5		px459dCas9 pcDNA	18.7	1.59	1.06E+00		
B6		px459dCas9 pcDNA	18.8	1.58	1.02E+00		
B7		px459dCas9 pcDNA	18.9	1.61	9.69E-01		
B8		Oligo Mix pcDNA	18.8	1.62	1.02E+00		
C1		Oligo Mix pcDNA	18.6	1.56	1.12E+00		
C2		Oligo Mix pcDNA	18.7	1.64	1.06E+00		

(Continued on next page)...

No.	Colour	Name	Take Off	Amplification	Comparative Conc.	Rep. Takeoff	Rep. Takeoff (95% CI)
C3		px459dCas9 EZH2	18.9	1.61	9.69E-01	18.8	[1.$,1.$]
C4		px459dCas9 EZH2	19.0	1.60	9.25E-01		
C5		px459dCas9 EZH2	18.6	1.64	1.12E+00		
C6		Oligo Mix EZH2	19.0	1.58	9.25E-01	19.1	[1.$,1.$]
C7		Oligo Mix EZH2	19.2	1.63	8.42E-01		
C8		Oligo Mix EZH2	19.1	1.58	8.83E-01		
D1		px459dCas9 pcDNA	18.8	1.64	1.02E+00		
D2		px459dCas9 pcDNA	18.9	1.64	9.69E-01		

D3		px459dCas9 pcDNA	19.0	1.62	9.25E-01		
D4		Oligo Mix pcDNA3	18.8	1.57	1.02E+00	18.7	[1.$,1.$]
D5		Oligo Mix pcDNA3	18.6	1.62	1.12E+00		
D6		Oligo Mix pcDNA3	18.7	1.59	1.06E+00		
D7		px459 dCas9 DNMt3A	18.8	1.58	1.02E+00	18.7	[1.$,1.$]
D8		px459 dCas9 DNMt3A	18.8	1.57	1.02E+00		
E1		px459 dCas9 DNMt3A	18.6	1.57	1.12E+00		
E2		Oligo Mix DNMT3A	19.2	1.60	8.42E-01	19.1	[1.$,1.$]
E3		Oligo Mix DNMT3A	19.1	1.56	8.83E-01		
E4		Oligo Mix DNMT3A	19.1	1.64	8.83E-01		
E5		px459dCas9 pcDNA3	18.5	1.61	1.17E+00	18.5	[1.$,1.$]
E6		px459dCas9 pcDNA3	18.3	1.59	1.28E+00		
E7		px459dCas9 pcDNA3	18.6	1.58	1.12E+00		
E8		Oligo Mix pcDNA	18.5	1.59	1.17E+00		
F1		Oligo Mix pcDNA	18.8	1.56	1.02E+00		
F2		Oligo Mix pcDNA	18.6	1.56	1.12E+00		
F3		px459dCas9 DNMT3A+L	18.4	1.58	1.22E+00	18.3	[1.$,1.$]
F4		px459dCas9 DNMT3A+L	18.4	1.56	1.22E+00		
F5		px459dCas9 DNMT3A+L	18.1	1.59	1.41E+00		
F6		Oligo Mix DNMT3A+L	18.6	1.63	1.12E+00	18.6	[1.$,1.$]
F7		Oligo Mix DNMT3A+L	18.8	1.56	1.02E+00		
F8		Oligo Mix DNMT3A+L	18.5	1.60	1.17E+00		
H2		H2O	35.1	0.00	4.96E-04	35.1	

This report generated by Rotor-Gene Real-Time Analysis Software 6.1 (Build 93)
© Corbett Research 2005
® All Rights Reserved
ISO 9001:2000 (Reg. No. QEC21313)

Quality Endorsed Company
ISO 9001 Lic 21313
SAI Global
