# Supplementary material for: Epigenetically silenced apoptosis-associated tyrosine kinase (AATK) facilitates a decreased expression of Cyclin D1 and WEE1, phosphorylates TP53 and reduces cell proliferation in a kinase-dependent manner
Source: Cancer Gene Ther. 2022 Jul 28;29(12):1975–87. doi: 10.1038/s41417-022-00513-x (PMC9750878; doi:10.1038/s41417-022-00513-x)
Supplement: Supplementary file 6 — Dataset original qPCR [file 41417_2022_513_MOESM6_ESM.zip › AATK_U87.pdf]

14 Hilly Street Mortlake NSW 2137 Australia
T +61 2 9736 1320
F +61 2 9736 1364
W www.corbettlifescience.com

Comparative Quantitation Report

Experiment Information

Run Name	Run 2016-09-28_AATK(4ul)_Aza_Glio_AATK
Run Start	28.09.2016 15:08:11
Run Finish	28.09.2016 17:17:31
Operator	MW
Notes	AATK C81/MeWo<-p53_Noco_Cisp LZ308/U87<-Aza triplicate
Run On Software Version	Rotor-Gene 6.1.93
Run Signature	The Run Signature is valid.
Gain FAM	8.
Gain ROX	8.

Comparative Quantitation Information

Reaction Amplification	0.68
Reaction Amplification Std. Deviation	0.87
Sample Page	Page 1
Control Replicate	(52)

Take off Graph for Cycling A.FAM/Cycling A.ROX

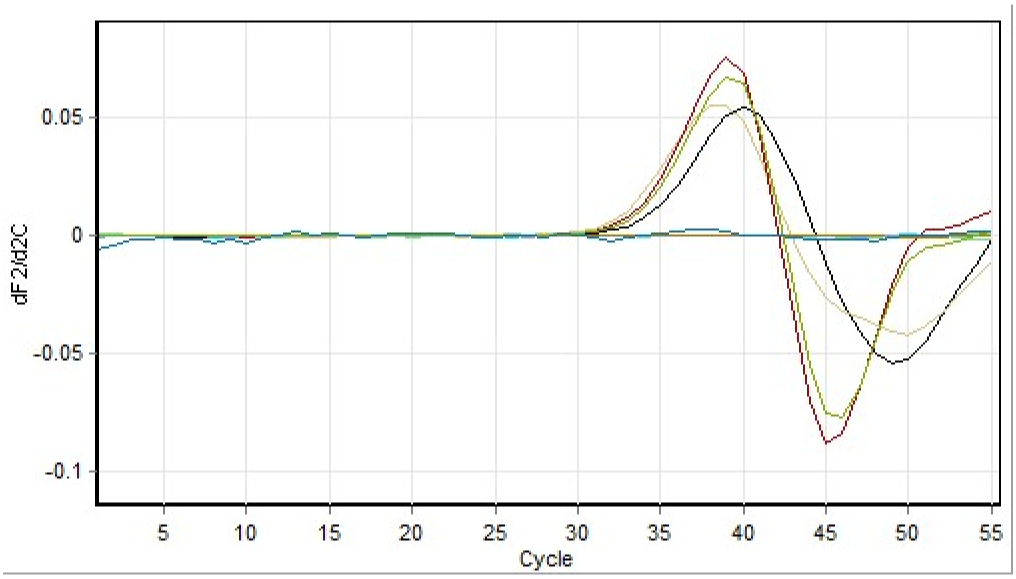

No.	Colour	Name	Take Off	Amplification	Comparative Conc.	Rep. Takeoff	Rep. Takeoff (95% CI)
F3		U87 5uM	35.2	1.66	7.11E-01	36.0	[1.$,1.$]
F4		U87 5uM	39.1	0.00	3.11E+00		
F5		U87 5uM	33.7	1.71	4.03E-01		
F6		U87 0uM	40.7	0.00	5.70E+00	33.0	[1.$,1.$]
F7		U87 0uM	37.3	0.00	1.57E+00		
F8		U87 0uM	21.1	0.00	3.42E-03		
G4		U87 10uM	34.8	1.71	6.11E-01	36.1	[1.$,1.$]
G5		U87 10uM	38.7	0.00	2.68E+00		
G6		U87 10uM	34.8	1.69	6.11E-01		
G8		H2O	11.4	0.07	8.71E-05	11.4	

Quality System™
Quality Endorsed Company
ISO 9001 Lic 21313
SAI Global

This report generated by Rotor-Gene Real-Time Analysis Software 6.1 (Build 93)
© Corbett Research 2005
® All Rights Reserved
ISO 9001:2000 (Reg. No. QEC21313)
