# Supplementary material for: Epigenetically silenced apoptosis-associated tyrosine kinase (AATK) facilitates a decreased expression of Cyclin D1 and WEE1, phosphorylates TP53 and reduces cell proliferation in a kinase-dependent manner
Source: Cancer Gene Ther. 2022 Jul 28;29(12):1975–87. doi: 10.1038/s41417-022-00513-x (PMC9750878; doi:10.1038/s41417-022-00513-x)
Supplement: Supplementary file 6 — Dataset original qPCR [file 41417_2022_513_MOESM6_ESM.zip › GAPDH_U87.pdf]

corbett RESEARCH
A Division of Corbett Life Science

14 Hilly Street Mortlake NSW 2137 Australia
T +61 2 9736 1320
F +61 2 9736 1364
W www.corbettlifescience.com

Comparative Quantitation Report

Experiment Information

Run Name	Run 2016-09-27_GAPDH_C81_MeWo_p53_Noco_Cisp_Aza_L_U
Run Start	27.09.2016 09:37:39
Run Finish	27.09.2016 11:14:42
Operator	MW
Notes	GAPDH C81/MeWo<-p53_Noco_Cisp LZ308/U87<-Aza triplicate
Run On Software Version	Rotor-Gene 6.1.93
Run Signature	The Run Signature is valid.
Gain FAM	8.
Gain ROX	8.

Comparative Quantitation Information

Reaction Amplification	1.66
Reaction Amplification Std. Deviation	0.02
Sample Page	Page 1
Control Replicate	(46)

Take off Graph for Cycling A.FAM

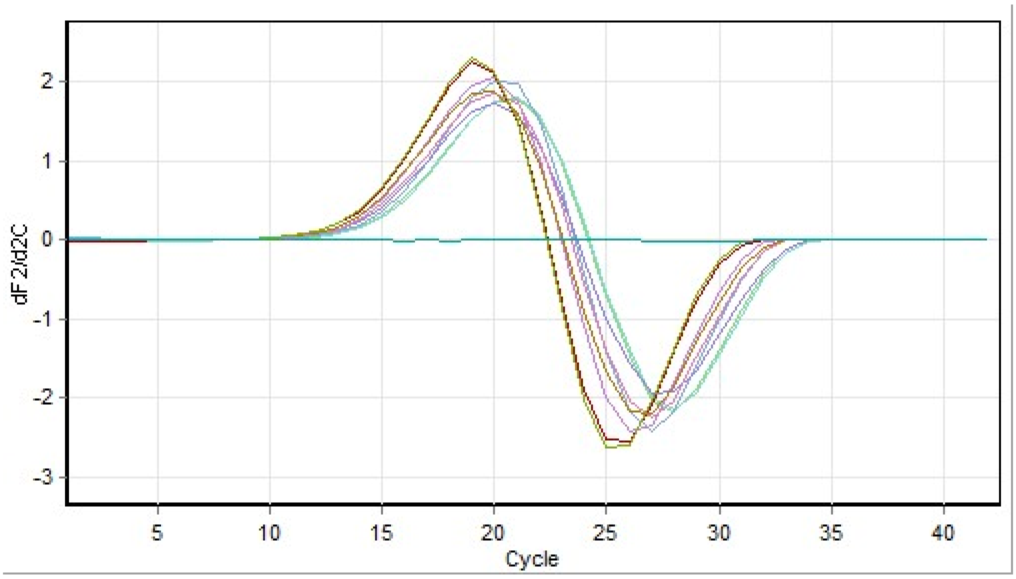

No.	Colour	Name	Take Off	Amplification	Comparative Conc.	Rep. Takeoff	Rep. Takeoff (95% CI)
F6		U87 0uM	15.8	1.65	1.00E+00	15.8	[1.$,1.$]
F7		U87 0uM	15.9	1.69	9.51E-01		
F8		U87 0uM	15.7	1.69	1.05E+00		
G1		U87 5uM	15.2	1.64	1.36E+00	15.1	[1.$,1.$]
G2		U87 5uM	15.1	1.66	1.43E+00		
G3		U87 5uM	15.1	1.63	1.43E+00		
G4		U87 10uM	15.0	1.67	1.50E+00	15.0	[1.$,1.$]
G5		U87 10uM	14.9	1.65	1.58E+00		
G6		U87 10uM	15.0	1.66	1.50E+00		
G7		H2O	19.3	0.86	1.69E-01	19.3	

Quality System™
Quality Endorsed Company
ISO 9001 Lic 21313
SAI Global

This report generated by Rotor-Gene Real-Time Analysis Software 6.1 (Build 93)
© Corbett Research 2005
® All Rights Reserved
ISO 9001:2000 (Reg. No. QEC21313)
