# Supplementary material for: Epigenetically silenced apoptosis-associated tyrosine kinase (AATK) facilitates a decreased expression of Cyclin D1 and WEE1, phosphorylates TP53 and reduces cell proliferation in a kinase-dependent manner
Source: Cancer Gene Ther. 2022 Jul 28;29(12):1975–87. doi: 10.1038/s41417-022-00513-x (PMC9750878; doi:10.1038/s41417-022-00513-x)
Supplement: Supplementary file 6 — Dataset original qPCR [file 41417_2022_513_MOESM6_ESM.zip › HCT116wt_CCND1.pdf]

corbett RESEARCH
A Division of Corbett Life Science

14 Hilly Street Mortlake NSW 2137 Australia
T + 61 2 9736 1320
F + 61 2 9736 1364
W www.corbettlifescience.com

Comparative Quantitation Report

Experiment Information

Run Name	Run 2019-07-05_CCND1_HCT-OE_starved
Run Start	05.07.2019 12:12:42
Run Finish	05.07.2019 14:09:22
Operator	MW
Notes	HCT OE starved Ccnd1 Triplicate
Run On Software Version	Rotor-Gene 6.1.93
Run Signature	The Run Signature is valid.
Gain FAM	8.
Gain ROX	9.33

Comparative Quantitation Information

Reaction Amplification	1.53
Reaction Amplification Std. Deviation	0.09
Sample Page	Page 1
Control Replicate	(1)

Take off Graph for Cycling A.FAM

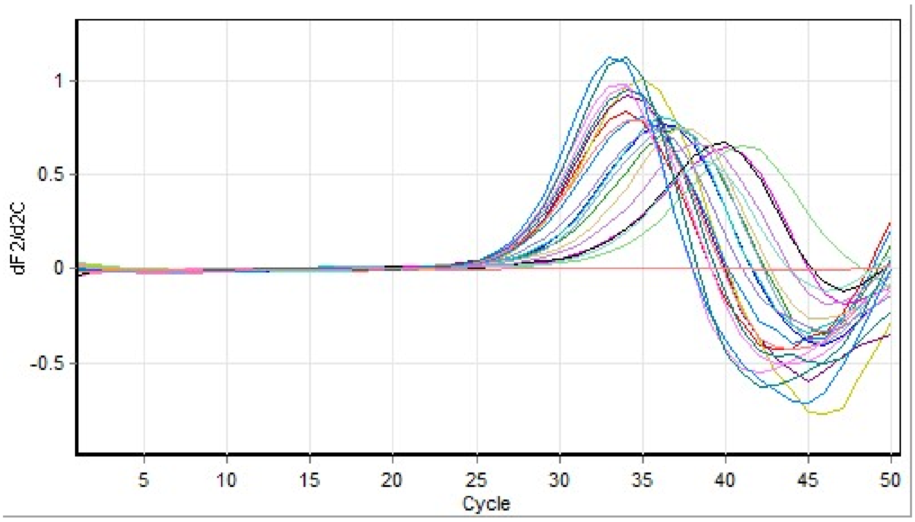

No.	Colour	Name	Take Off	Amplification	Comparative Conc.	Rep. Takeoff	Rep. Takeoff (95% CI)
A1		HCT116 wt p53 w/o	28.8	1.63	1.29E+00	29.4	[1.$,1.$]
A2		HCT116 wt p53 w/o	29.2	1.56	1.09E+00		
A3		HCT116 wt p53 w/o	30.2	1.49	7.13E-01		
A4		HCT116 wt p53 EYFP	28.8	1.64	1.29E+00	28.8	[1.$,1.$]
A5		HCT116 wt p53 EYFP	28.6	1.62	1.40E+00		
A6		HCT116 wt p53 EYFP	29.0	1.52	1.18E+00		
A7		HCT116 wt p53 AATKA	28.7	1.66	1.34E+00	29.3	[1.$,1.$]
A8		HCT116 wt p53 AATKA	28.5	1.59	1.46E+00		
B1		HCT116 wt p53 AATKA	30.8	1.42	5.53E-01		
B2		HCT116 wt p53 AATKA KD	33.2	1.39	2.00E-01	32.3	[1.$,1.$]
B3		HCT116 wt p53 AATKA KD	33.4	1.43	1.84E-01		
B4		HCT116 wt p53 AATKA KD	30.4	1.47	6.55E-01		
B5		HCT116 wt p53 AATKB	31.6	1.48	3.94E-01	33.2	[1.$,1.$]
B6		HCT116 wt p53 AATKB	34.9	1.39	9.75E-02		
B7		HCT116 wt p53 AATKB	33.2	1.53	2.00E-01		
B8		HCT116 wt p53 AATKB KD	30.7	1.53	5.77E-01	30.8	[1.$,1.$]
C1		HCT116 wt p53 AATKB KD	29.5	1.50	9.59E-01		
C2		HCT116 wt p53 AATKB KD	32.1	1.41	3.19E-01		

(Continued on next page)...

No.	Colour	Name	Take Off	Amplification	Comparative Conc.	Rep. Takeoff	Rep. Takeoff (95% CI)
E5		Mix	28.5	1.62	1.46E+00	28.6	[1.$,1.$]
E6		Mix	28.5	1.61	1.46E+00		
E7		Mix	28.9	1.60	1.24E+00		
E8		H2O	27.2	0.31	2.54E+00	27.2	

Quality System ™
Quality Endorsed Company
ISO 9001 Lic 21313
SAI Global

This report generated by Rotor-Gene Real-Time Analysis Software 6.1 (Build 93)
© Corbett Research 2005
® All Rights Reserved
ISO 9001:2000 (Reg. No. QEC21313)
