# Supplementary material for: Epigenetically silenced apoptosis-associated tyrosine kinase (AATK) facilitates a decreased expression of Cyclin D1 and WEE1, phosphorylates TP53 and reduces cell proliferation in a kinase-dependent manner
Source: Cancer Gene Ther. 2022 Jul 28;29(12):1975–87. doi: 10.1038/s41417-022-00513-x (PMC9750878; doi:10.1038/s41417-022-00513-x)
Supplement: Supplementary file 6 — Dataset original qPCR [file 41417_2022_513_MOESM6_ESM.zip › U251_ACTB.pdf]

corbett RESEARCH
A Division of Corbett Life Science

14 Hilly Street Mortlake NSW 2137 Australia
T +61 2 9736 1320
F +61 2 9736 1364
W www.corbettlifescience.com

Comparative Quantitation Report

Experiment Information

Run Name	Run 2020-09-18_b-Act_OE-EY_U343_U251_A549_A427_(1)(2)
Run Start	18.09.2020 08:47:00
Run Finish	18.09.2020 10:20:03
Operator	MW
Notes	b-Act OE EY (1)(2) U343 U251 A549 A427 triplicate
Run On Software Version	Rotor-Gene 6.1.93
Run Signature	The Run Signature is valid.
Gain FAM	8.
Gain ROX	9.33

Comparative Quantitation Information

Reaction Amplification	1.68
Reaction Amplification Std. Deviation	0.05
Sample Page	Page 1
Control Replicate	(19)

Take off Graph for Cycling A.FAM/Cycling A.ROX

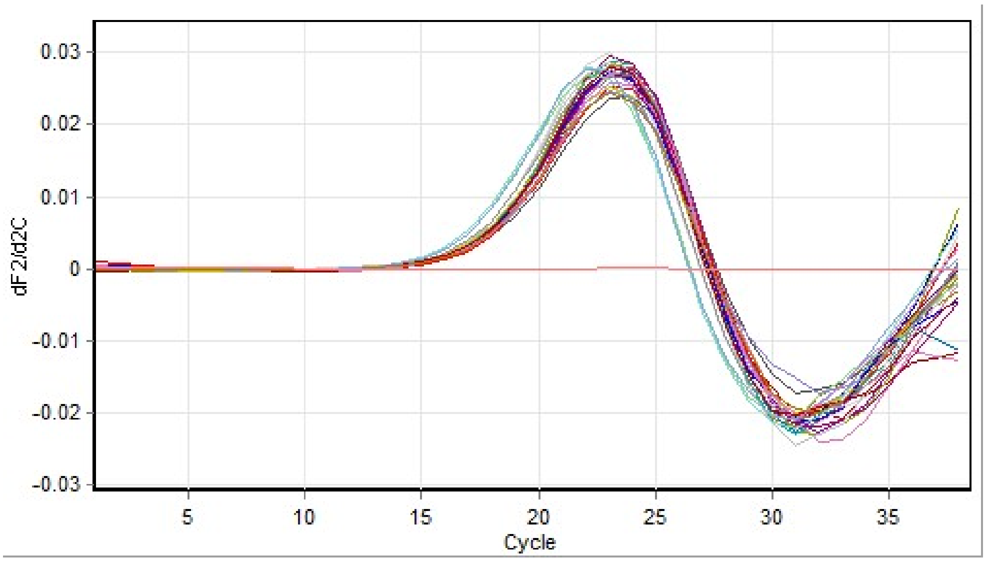

No.	Colour	Name	Take Off	Amplification	Comparative Conc.	Rep. Takeoff	Rep. Takeoff (95% CI)
C3		U251 EY (1)	18.6	1.71	1.02E+00	18.6	[1.$,1.$]
C4		U251 EY (1)	18.6	1.73	1.02E+00		
C5		U251 EY (1)	18.7	1.68	9.66E-01		
C6		U251 B-EY (1)	18.6	1.71	1.02E+00	18.6	[1.$,1.$]
C7		U251 B-EY (1)	18.6	1.73	1.02E+00		
C8		U251 B-EY (1)	18.6	1.71	1.02E+00		
D1		U251 B KD-EY (1)	18.7	1.72	9.66E-01	18.8	[1.$,1.$]
D2		U251 B KD-EY (1)	18.9	1.63	8.71E-01		
D3		U251 B KD-EY (1)	18.8	1.71	9.17E-01		
D4		U251 EY (2)	18.4	1.68	1.13E+00	18.4	[1.$,1.$]
D5		U251 EY (2)	18.4	1.74	1.13E+00		
D6		U251 EY (2)	18.3	1.70	1.19E+00		
D7		U251 B-EY (2)	18.9	1.64	8.71E-01	18.8	[1.$,1.$]
D8		U251 B-EY (2)	18.8	1.67	9.17E-01		
E1		U251 B-EY (2)	18.7	1.57	9.66E-01		
E2		U251 B KD-EY (2)	18.3	1.64	1.19E+00	18.5	[1.$,1.$]
E3		U251 B KD-EY (2)	18.7	1.66	9.66E-01		
E4		U251 B KD-EY (2)	18.5	1.69	1.07E+00		

(Continued on next page)...

No.	Colour	Name	Take Off	Amplification	Comparative Conc.	Rep. Takeoff	Rep. Takeoff (95% CI)
F6		U251 EY (3)	17.9	1.66	1.46E+00	17.9	[1.$,1.$]
F7		U251 EY (3)	17.8	1.64	1.54E+00		
F8		U251 EY (3)	17.9	1.67	1.46E+00		
G1		U251 B-EY (3)	18.7	1.57	9.66E-01	18.7	[1.$,1.$]
G2		U251 B-EY (3)	18.6	1.69	1.02E+00		

G3		U251 B-EY (3)	18.9	1.63	8.71E-01		
G4		U251 B KD-EY (3)	18.6	1.76	1.02E+00	18.6	
G5		U251 B KD-EY (3)	18.5	1.71	1.07E+00		
I8		H2O	21.6	0.00	2.15E-01	21.6	

Quality System
Quality Endorsed Company
ISO 9001 Lic 21313
SAI Global

™ This report generated by Rotor-Gene Real-Time Analysis Software 6.1 (Build 93)
© Corbett Research 2005
® All Rights Reserved
ISO 9001:2000 (Reg. No. QEC21313)
