# Supplementary material for: Epigenetically silenced apoptosis-associated tyrosine kinase (AATK) facilitates a decreased expression of Cyclin D1 and WEE1, phosphorylates TP53 and reduces cell proliferation in a kinase-dependent manner
Source: Cancer Gene Ther. 2022 Jul 28;29(12):1975–87. doi: 10.1038/s41417-022-00513-x (PMC9750878; doi:10.1038/s41417-022-00513-x)
Supplement: Supplementary file 6 — Dataset original qPCR [file 41417_2022_513_MOESM6_ESM.zip › AATK_PaTu-S.pdf]

corbett RESEARCH
A Division of Corbett Life Science

14 Hilly Street Mortlake NSW 2137 Australia
T +61 2 9736 1320
F +61 2 9736 1364
W www.corbettlifescience.com

Comparative Quantitation Report

Experiment Information

Run Name	Run 2017-01-19_AATK_Pankreas_Aza_all
Run Start	19.01.2017 14:18:51
Run Finish	19.01.2017 16:16:28
Operator	MW
Notes	AATK Aza Pankeas all triplicate
Run On Software Version	Rotor-Gene 6.1.93
Run Signature	The Run Signature is valid.
Gain FAM	8.
Gain ROX	9.33

Comparative Quantitation Information

Reaction Amplification	1.75
Reaction Amplification Std. Deviation	0.04
Sample Page	Page 1
Control Replicate	(31)

Take off Graph for Cycling A.FAM/Cycling A.ROX

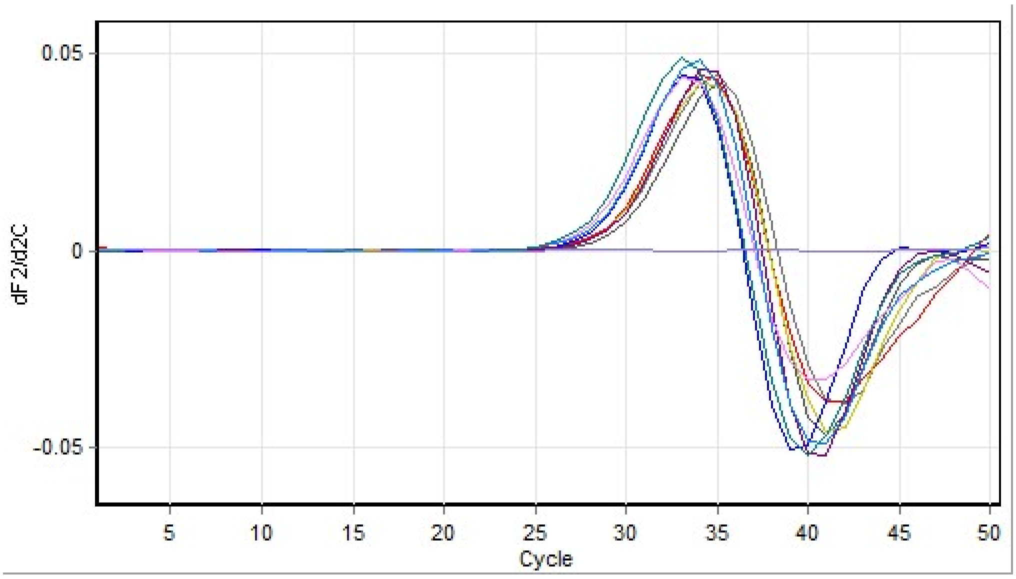

No.	Colour	Name	Take Off	Amplification	Comparative Conc.	Rep. Takeoff	Rep. Takeoff (95% CI)
D7		PaTu-S 0 uM	30.5	1.78	1.02E+00	30.5	[1.$,1.$]
D8		PaTu-S 0 uM	30.8	1.75	8.61E-01		
E1		PaTu-S 0 uM	30.3	1.74	1.14E+00		
E2		PaTu-S 5 uM	30.2	1.79	1.21E+00	30.2	[1.$,1.$]
E3		PaTu-S 5 uM	29.7	1.79	1.60E+00		
E4		PaTu-S 5 uM	30.6	1.78	9.63E-01		
E5		PaTu-S 10 uM	29.2	1.66	2.11E+00	29.3	[1.$,1.$]
E6		PaTu-S 10 uM	29.7	1.74	1.60E+00		
E7		PaTu-S 10 uM	29.1	1.74	2.24E+00		
G1		H2O	27.8	0.00	4.64E+00	27.8	

Quality System™
Quality Endorsed Company
ISO 9001 Lic 21313
SAI Global

This report generated by Rotor-Gene Real-Time Analysis Software 6.1 (Build 93)
© Corbett Research 2005
® All Rights Reserved
ISO 9001:2000 (Reg. No. QEC21313)
