# Supplementary material for: Epigenetically silenced apoptosis-associated tyrosine kinase (AATK) facilitates a decreased expression of Cyclin D1 and WEE1, phosphorylates TP53 and reduces cell proliferation in a kinase-dependent manner
Source: Cancer Gene Ther. 2022 Jul 28;29(12):1975–87. doi: 10.1038/s41417-022-00513-x (PMC9750878; doi:10.1038/s41417-022-00513-x)
Supplement: Supplementary file 6 — Dataset original qPCR [file 41417_2022_513_MOESM6_ESM.zip › U251_CCND1.pdf]

14 Hilly Street Mortlake NSW 2137 Australia
T +61 2 9736 1320
F +61 2 9736 1364
W www.corbettlifescience.com

Comparative Quantitation Report

Experiment Information

Run Name	Run 2020-09-18_CCND1_OE_(1)(2)_U343_U251_A549_A427
Run Start	18.09.2020 12:23:09
Run Finish	18.09.2020 14:19:08
Operator	MW
Notes	CCND1 OE EY (1) (2) U343 U251 A549 A427 triplicate
Run On Software Version	Rotor-Gene 6.1.93
Run Signature	The Run Signature is valid.
Gain FAM	8.
Gain ROX	9.33

Comparative Quantitation Information

Reaction Amplification	1.55
Reaction Amplification Std. Deviation	0.11
Sample Page	Page 1
Control Replicate	(19)

Take off Graph for Cycling A.FAM/Cycling A.ROX

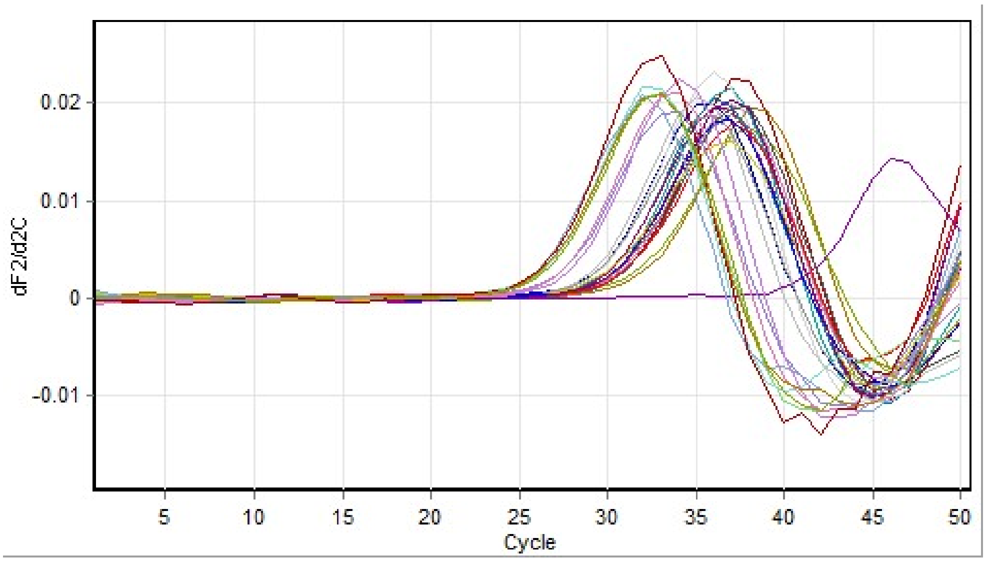

No.	Colour	Name	Take Off	Amplification	Comparative Conc.	Rep. Takeoff	Rep. Takeoff (95% CI)
C3		U251 EY (1)	31.9	1.59	1.36E+00	32.6	[1.$,1.$]
C4		U251 EY (1)	32.5	1.47	1.05E+00		
C5		U251 EY (1)	33.4	1.44	7.03E-01		
C6		U251 B KD-EY (1)	32.7	1.50	9.57E-01	31.9	[1.$,1.$]
C7		U251 B KD-EY (1)	31.7	1.60	1.49E+00		
C8		U251 B KD-EY (1)	31.4	1.71	1.70E+00		
D1		U251 B-EY (1)	30.8	1.60	2.21E+00	34.8	[1.$,1.$]
D2		U251 B-EY (1)	42.4	1.36	1.33E-02		
D3		U251 B-EY (1)	31.3	1.35	1.77E+00		
D4		U251 EY (2)	31.2	1.68	1.85E+00	30.8	[1.$,1.$]
D5		U251 EY (2)	30.5	1.46	2.52E+00		
D6		U251 EY (2)	30.6	1.48	2.41E+00		
D7		U251 B KD-EY (2)	31.7	1.53	1.49E+00	31.9	[1.$,1.$]
D8		U251 B KD-EY (2)	32.1	1.68	1.25E+00		
E1		U251 B KD-EY (2)	31.8	1.51	1.42E+00		
E2		U251 B-EY (2)	31.0	1.14	2.02E+00	31.5	[1.$,1.$]
E3		U251 B-EY (2)	31.6	1.47	1.55E+00		
E4		U251 B-EY (2)	31.8	1.59	1.42E+00		

(Continued on next page)...

No.	Colour	Name	Take Off	Amplification	Comparative Conc.	Rep. Takeoff	Rep. Takeoff (95% CI)
F6		A549 EY (2)	27.6	1.47	9.05E+00	27.6	[1.$,1.$]
F7		A549 EY (2)	27.9	1.60	7.93E+00		
F8		A549 EY (2)	27.4	1.70	9.89E+00		
G1		A549 B-EY (2)	28.6	1.48	5.83E+00	28.8	[1.$,1.$]
G2		A549 B-EY (2)	29.2	1.75	4.47E+00		

G3		A549 B-EY (2)	28.7	1.57	5.58E+00		
G4		A549 B KD-EY (2)	27.6	1.67	9.05E+00	27.7	[1.$,1.$]
G5		A549 B KD-EY (2)	27.7	1.52	8.66E+00		
G6		A549 B KD-EY (2)	27.9	1.61	7.93E+00		

Quality System
Quality Endorsed Company
ISO 9001 Lic 21313
SAI Global

This report generated by Rotor-Gene Real-Time Analysis Software 6.1 (Build 93)
© Corbett Research 2005
® All Rights Reserved
ISO 9001:2000 (Reg. No. QEC21313)
