# Supplementary material for: Epigenetically silenced apoptosis-associated tyrosine kinase (AATK) facilitates a decreased expression of Cyclin D1 and WEE1, phosphorylates TP53 and reduces cell proliferation in a kinase-dependent manner
Source: Cancer Gene Ther. 2022 Jul 28;29(12):1975–87. doi: 10.1038/s41417-022-00513-x (PMC9750878; doi:10.1038/s41417-022-00513-x)
Supplement: Supplementary file 6 — Dataset original qPCR [file 41417_2022_513_MOESM6_ESM.zip › RNAi_GAPDH_1.pdf]

corbett
RESEARCH
A Division of Corbett Life Science

14 Hilly Street Mortlake NSW 2137 Australia
T +61 2 9736 1320
F +61 2 9736 1364
W www.corbettlifescience.com

Comparative Quantitation Report

Experiment Information

Run Name	Run 2020-03-28_GAPDH_RNAi_div-CL
Run Start	28.03.2020 10:31:06
Run Finish	28.03.2020 12:22:39
Operator	MW
Notes	GAPDH RNAi div CL triplicate
Run On Software Version	Rotor-Gene 6.1.93
Run Signature	The Run Signature is valid.
Gain FAM	8.
Gain ROX	8.

Comparative Quantitation Information

Reaction Amplification	1.62
Reaction Amplification Std. Deviation	0.03
Sample Page	Page 1
Control Replicate	(7)

Take off Graph for Cycling A.FAM/Cycling A.ROX

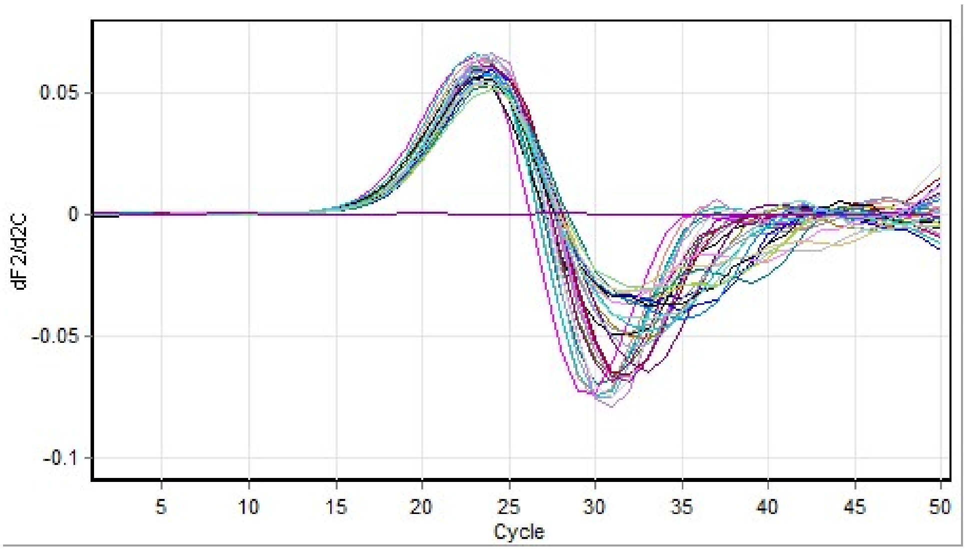

No.	Colour	Name	Take Off	Amplification	Comparative Conc.	Rep. Takeoff	Rep. Takeoff (95% CI)
A7		HEK siCtrl (1)	18.7	1.62	9.68E-01	18.6	[1.$,1.$]
A8		HEK siCtrl (1)	18.6	1.63	1.02E+00		
B1		HEK siCtrl (1)	18.6	1.66	1.02E+00		
B2		HEK siAATK (1)	18.1	1.62	1.29E+00	18.3	[1.$,1.$]
B3		HEK siAATK (1)	18.3	1.62	1.17E+00		
B4		HEK siAATK (1)	18.5	1.64	1.07E+00		
B8		HEK siCtrl (2)	19.2	1.64	7.61E-01	19.2	[1.$,1.$]
C1		HEK siCtrl (2)	19.1	1.63	7.98E-01		
C2		HEK siCtrl (2)	19.3	1.62	7.25E-01		
C3		HEK siAATK (2)	19.3	1.63	7.25E-01	19.3	[1.$,1.$]
C4		HEK siAATK (2)	19.3	1.64	7.25E-01		
C5		HEK siAATK (2)	19.3	1.58	7.25E-01		
D1		SkMel13 siCtrl (1)	18.9	1.58	8.79E-01	19.0	[1.$,1.$]
D2		SkMel13 siCtrl (1)	19.0	1.62	8.38E-01		
D3		SkMel13 siCtrl (1)	19.0	1.63	8.38E-01		
D4		SkMel13 siAATK (1)	18.9	1.64	8.79E-01	19.0	[1.$,1.$]
D5		SkMel13 siAATK (1)	18.9	1.60	8.79E-01		
D6		SkMel13 siAATK (1)	19.1	1.57	7.98E-01		

(Continued on next page)...

No.	Colour	Name	Take Off	Amplification	Comparative Conc.	Rep. Takeoff	Rep. Takeoff (95% CI)
E2		SkMel13 siCtrl (2)	19.0	1.62	8.38E-01	19.2	[1.$,1.$]
E3		SkMel13 siCtrl (2)	19.4	1.63	6.90E-01		
E4		SkMel13 siCtrl (2)	19.1	1.61	7.98E-01		
E5		SkMel13 siAATK (2)	19.0	1.61	8.38E-01	19.0	[1.$,1.$]
E6		SkMel13 siAATK (2)	19.0	1.62	8.38E-01		
E7		SkMel13 siAATK (2)	19.1	1.64	7.98E-01		

F3		MCF7 siCtrl (1)	18.9	1.68	8.79E-01	18.8	[1.$,1.$]
F4		MCF7 siCtrl (1)	18.9	1.65	8.79E-01		
F5		MCF7 siCtrl (1)	18.7	1.58	9.68E-01		
F6		MCF7 siAATK (1)	19.2	1.57	7.61E-01	19.0	[1.$,1.$]
F7		MCF7 siAATK (1)	18.8	1.60	9.23E-01		
F8		MCF7 siAATK (1)	19.0	1.62	8.38E-01		
H2		H2O	26.5	0.00	2.24E-02	26.5	

Quality System
Quality Endorsed Company
ISO 9001 Lic 21313
SAI Global
™ This report generated by Rotor-Gene Real-Time Analysis Software 6.1 (Build 93)
© Corbett Research 2005
® All Rights Reserved
ISO 9001:2000 (Reg. No. QEC21313)
