# Supplementary material for: Epigenetically silenced apoptosis-associated tyrosine kinase (AATK) facilitates a decreased expression of Cyclin D1 and WEE1, phosphorylates TP53 and reduces cell proliferation in a kinase-dependent manner
Source: Cancer Gene Ther. 2022 Jul 28;29(12):1975–87. doi: 10.1038/s41417-022-00513-x (PMC9750878; doi:10.1038/s41417-022-00513-x)
Supplement: Supplementary file 6 — Dataset original qPCR [file 41417_2022_513_MOESM6_ESM.zip › HEK_WEE1.pdf]

corbett
RESEARCH
A Division of Corbett Life Science

14 Hilly Street Mortlake NSW 2137 Australia
T +61 2 9736 1320
F +61 2 9736 1364
W www.corbettlifescience.com

Comparative Quantitation Report

Experiment Information

Run Name	Run 2021-03-18_Wee1_HEK-OE+UV_(1)(2)_SkMel13-OE_(1)-(3)
Run Start	18.03.2021 10:27:36
Run Finish	18.03.2021 12:14:11
Operator	MW
Notes	Wee1 HEK OE UV (1)(2)_Skmel13 OE (1)-(3) triplicate
Run On Software Version	Rotor-Gene 6.1.93
Run Signature	The Run Signature is valid.
Gain FAM	8.
Gain ROX	9.33

Comparative Quantitation Information

Reaction Amplification	1.64
Reaction Amplification Std. Deviation	0.04
Sample Page	Page 1
Control Replicate	(1)

Take off Graph for Cycling A.FAM/Cycling A.ROX

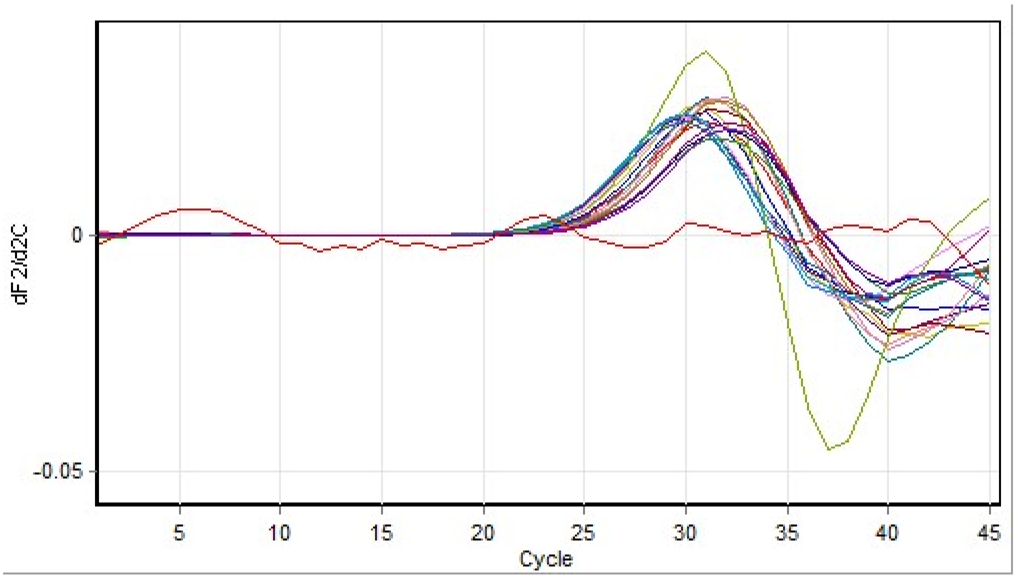

No.	Colour	Name	Take Off	Amplification	Comparative Conc.	Rep. Takeoff	Rep. Takeoff (95% CI)
A1		EY ohne (1)	26.0	1.59	1.00E+00	26.0	[1.$,1.$]
A2		EY ohne (1)	25.9	1.64	1.05E+00		
A3		EY ohne (1)	26.1	1.66	9.52E-01		
A4		B KD ohne (1)	25.3	1.68	1.41E+00	25.2	[1.$,1.$]
A5		B KD ohne (1)	25.2	1.69	1.49E+00		
A6		B KD ohne (1)	25.0	1.69	1.64E+00		
A7		B ohne (1)	26.6	1.64	7.43E-01	26.6	[1.$,1.$]
A8		B ohne (1)	26.6	1.67	7.43E-01		
B1		B ohne (1)	26.7	1.60	7.07E-01		
C3		EY ohne (2)	26.9	1.65	6.40E-01	26.9	[1.$,1.$]
C4		EY ohne (2)	26.8	1.67	6.73E-01		
C5		EY ohne (2)	27.0	1.64	6.09E-01		
C6		B KD ohne (2)	26.4	1.61	8.20E-01	25.4	[1.$,1.$]
C7		B KD ohne (2)	25.0	1.66	1.64E+00		
C8		B KD ohne (2)	24.9	1.68	1.72E+00		
D1		B ohne (2)	26.9	1.57	6.40E-01	27.0	
D2		B ohne (2)	27.1	1.60	5.80E-01		
D3		B ohne (2)	27.0	1.61	6.09E-01	27.0	
I1		H20	36.9	0.19	4.51E-03	36.9	

Quality System™
Quality Endorsed Company
ISO 9001 Lic 21313
SAI Global

This report generated by Rotor-Gene Real-Time Analysis Software 6.1 (Build 93)
© Corbett Research 2005
® All Rights Reserved
ISO 9001:2000 (Reg. No. QEC21313)
