# Supplementary material for: Epigenetically silenced apoptosis-associated tyrosine kinase (AATK) facilitates a decreased expression of Cyclin D1 and WEE1, phosphorylates TP53 and reduces cell proliferation in a kinase-dependent manner
Source: Cancer Gene Ther. 2022 Jul 28;29(12):1975–87. doi: 10.1038/s41417-022-00513-x (PMC9750878; doi:10.1038/s41417-022-00513-x)
Supplement: Supplementary file 6 — Dataset original qPCR [file 41417_2022_513_MOESM6_ESM.zip › ACTB_clone pools.pdf]

corbett RESEARCH
A Division of Corbett Life Science

14 Hilly Street Mortlake NSW 2137 Australia
T + 61 2 9736 1320
F + 61 2 9736 1364
W www.corbettlifescience.com

Comparative Quantitation Report

Experiment Information

Run Name	Run 2020-04-11_b-Act_Affy-cDNA
Run Start	11.04.2020 12:22:52
Run Finish	11.04.2020 13:58:31
Operator	MW
Notes	b-Act Affy cDNA triplicate
Run On Software Version	Rotor-Gene 6.1.93
Run Signature	The Run Signature is valid.
Gain FAM	8.
Gain ROX	8.

Comparative Quantitation Information

Reaction Amplification	1.69
Reaction Amplification Std. Deviation	0.02
Sample Page	Page 1
Control Replicate	(4)

Take off Graph for Cycling A.FAM/Cycling A.ROX

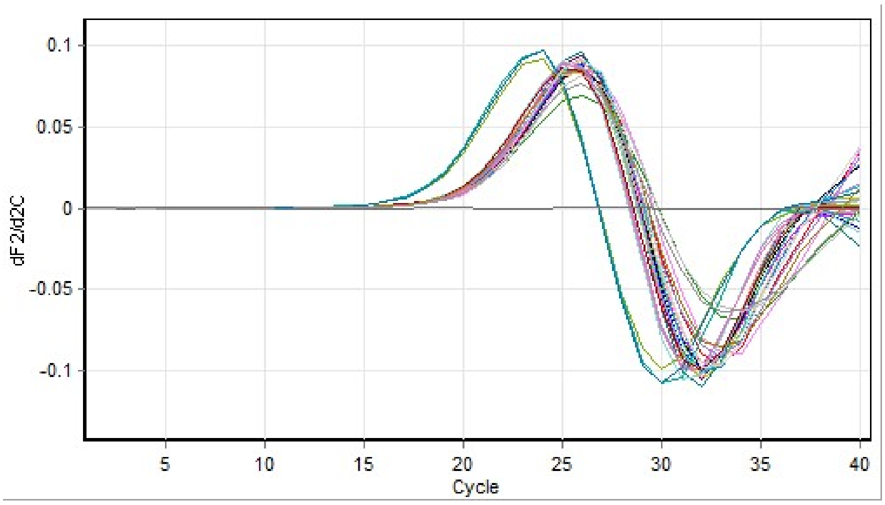

No.	Colour	Name	Take Off	Amplification	Comparative Conc.	Rep. Takeoff	Rep. Takeoff (95% CI)
A1		Control clone pool (1)	21.7	1.69	1.11E+00	21.7	[1.$,1.$]
A2		Control clone pool (1)	21.7	1.70	1.11E+00		
A3		Control clone pool (1)	21.6	1.70	1.17E+00		
A4		Control clone pool (2)	21.9	1.69	1.00E+00	21.9	[1.$,1.$]
A5		Control clone pool (2)	21.9	1.66	1.00E+00		
A6		Control clone pool (2)	21.9	1.67	1.00E+00		
A7		Clone pool AATK (1) unind	21.7	1.70	1.11E+00	21.6	[1.$,1.$]
A8		Clone pool AATK (1) unind	21.7	1.68	1.11E+00		
B1		Clone pool AATK (1) unind	21.3	1.66	1.37E+00		
B2		Clone pool AATK (1)	21.7	1.67	1.11E+00	21.7	[1.$,1.$]
B3		Clone pool AATK (1)	21.7	1.67	1.11E+00		
B4		Clone pool AATK (1)	21.8	1.70	1.05E+00		
B5		Clone pool AATK KD (1) unind	21.1	1.70	1.52E+00	21.2	[1.$,1.$]
B6		Clone pool AATK KD (1) unind	21.3	1.70	1.37E+00		
B7		Clone pool AATK KD (1) unind	21.1	1.70	1.52E+00		
B8		Clone pool AATK KD (1)	21.4	1.69	1.30E+00	21.4	[1.$,1.$]
C1		Clone pool AATK KD (1)	21.2	1.70	1.44E+00		
C2		Clone pool AATK KD (1)	21.5	1.71	1.23E+00		

(Continued on next page)...

No.	Colour	Name	Take Off	Amplification	Comparative Conc.	Rep. Takeoff	Rep. Takeoff (95% CI)
C3		Clone pool AATK (2) unind	21.4	1.69	1.30E+00	21.2	[1.$,1.$]
C4		Clone pool AATK (2) unind	21.0	1.71	1.60E+00		
C5		Clone pool AATK (2) unind	21.3	1.68	1.37E+00		
C6		Clone pool AATK (2)	19.5	1.66	3.51E+00	19.5	[1.$,1.$]
C7		Clone pool AATK (2)	19.4	1.69	3.70E+00		
C8		Clone pool AATK (2)	19.5	1.69	3.51E+00		
D4		Clone pool AATK KD (2)	21.7	1.70	1.11E+00	21.6	[1.$,1.$]
D5		Clone pool AATK KD (2)	21.7	1.68	1.11E+00		
D6		Clone pool AATK KD (2)	21.5	1.67	1.23E+00		
D7		H2O GAPDH	28.1	0.00	3.90E-02	28.1	

Quality System
Quality Endorsed Company
ISO 9001 Lic 21313
SAI Global

This report generated by Rotor-Gene Real-Time Analysis Software 6.1 (Build 93)
© Corbett Research 2005
® All Rights Reserved
ISO 9001:2000 (Reg. No. QEC21313)
