# Supplementary material for: Epigenetically silenced apoptosis-associated tyrosine kinase (AATK) facilitates a decreased expression of Cyclin D1 and WEE1, phosphorylates TP53 and reduces cell proliferation in a kinase-dependent manner
Source: Cancer Gene Ther. 2022 Jul 28;29(12):1975–87. doi: 10.1038/s41417-022-00513-x (PMC9750878; doi:10.1038/s41417-022-00513-x)
Supplement: Supplementary file 6 — Dataset original qPCR [file 41417_2022_513_MOESM6_ESM.zip › U251_GAPDH.pdf]

corbett RESEARCH
A Division of Corbett Life Science

14 Hilly Street Mortlake NSW 2137 Australia
T +61 2 9736 1320
F +61 2 9736 1364
W www.corbettlifescience.com

Comparative Quantitation Report

Experiment Information

Run Name	Run 2021-03-17_GAPDH_OE-EY_U343_U251_A549_A427
Run Start	17.03.2021 08:52:02
Run Finish	17.03.2021 10:17:21
Operator	MW
Notes	GAPDH OE U343 U251 A549 A427 triplicate
Run On Software Version	Rotor-Gene 6.1.93
Run Signature	The Run Signature is valid.
Gain FAM	8.
Gain ROX	9.33

Comparative Quantitation Information

Reaction Amplification	1.62
Reaction Amplification Std. Deviation	0.03
Sample Page	Page 1
Control Replicate	(19)

Take off Graph for Cycling A.FAM/Cycling A.ROX

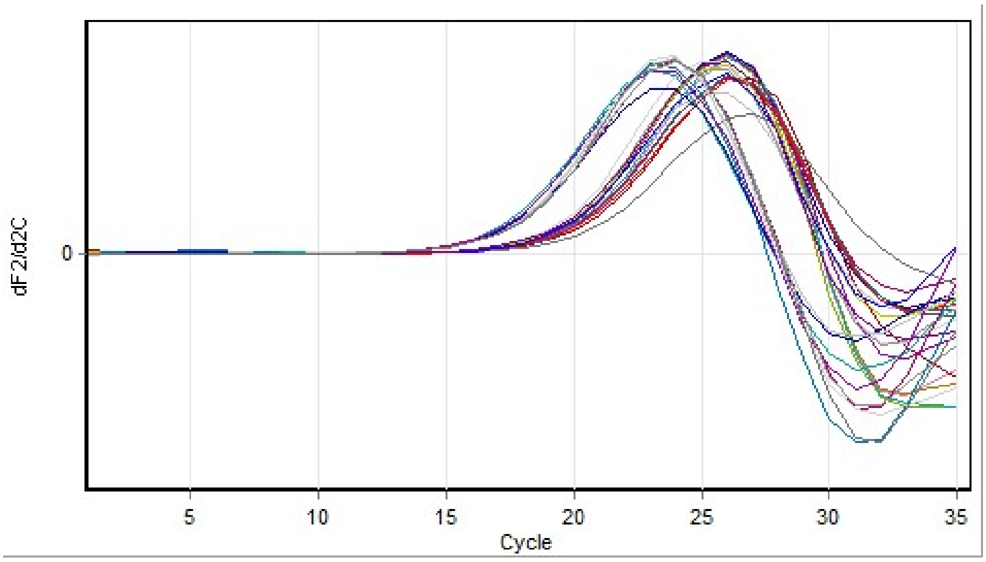

No.	Colour	Name	Take Off	Amplification	Comparative Conc.	Rep. Takeoff	Rep. Takeoff (95% CI)
C3		U251 EY (3)	21.4	1.66	9.53E-01	21.3	[1.$,1.$]
C4		U251 EY (3)	21.5	1.66	9.08E-01		
C5		U251 EY (3)	21.0	1.60	1.16E+00		
C6		U251 B-EY (3)	21.0	1.64	1.16E+00	21.2	[1.$,1.$]
C7		U251 B-EY (3)	21.3	1.60	1.00E+00		
C8		U251 B-EY (3)	21.3	1.65	1.00E+00		
D1		U251 B KD-EY (3)	21.0	1.63	1.16E+00	21.1	[1.$,1.$]
D2		U251 B KD-EY (3)	21.0	1.65	1.16E+00		
D3		U251 B KD-EY (3)	21.2	1.59	1.05E+00		
D4		U251 EY (2)	20.5	1.66	1.47E+00	20.5	[1.$,1.$]
D5		U251 EY (2)	20.4	1.62	1.55E+00		
D6		U251 EY (2)	20.7	1.63	1.34E+00		
D7		U251 B-EY (2)	21.6	1.59	8.65E-01	21.4	[1.$,1.$]
D8		U251 B-EY (2)	21.3	1.60	1.00E+00		
E1		U251 B-EY (2)	21.2	1.55	1.05E+00		
E2		U251 B KD-EY (2)	20.8	1.62	1.27E+00	20.8	[1.$,1.$]
E3		U251 B KD-EY (2)	20.8	1.63	1.27E+00		
E4		U251 B KD-EY (2)	20.7	1.57	1.34E+00		

(Continued on next page)...

No.	Colour	Name	Take Off	Amplification	Comparative Conc.	Rep. Takeoff	Rep. Takeoff (95% CI)
G7		U251 EY (1)	18.3	1.62	4.27E+00	18.5	[1.$,1.$]
G8		U251 EY (1)	18.7	1.62	3.52E+00		
H1		U251 EY (1)	18.5	1.59	3.88E+00		
H2		U251 B-EY 1)	18.5	1.68	3.88E+00	18.8	[1.$,1.$]
H3		U251 B-EY 1)	19.0	1.63	3.04E+00		

H4		U251 B-EY 1)	19.0	1.59	3.04E+00		
H5		U251 B KD-EY (1)	19.0	1.62	3.04E+00	19.0	[1.$,1.$]
H6		U251 B KD-EY (1)	19.0	1.57	3.04E+00		
H7		U251 B KD-EY (1)	19.0	1.66	3.04E+00		

™ This report generated by Rotor-Gene Real-Time Analysis Software 6.1 (Build 93)
© Corbett Research 2005
® All Rights Reserved
ISO 9001:2000 (Reg. No. QEC21313)

Quality System
Quality Endorsed Company
ISO 9001 Lic 21313
SAI Global
