# Supplementary material for: Epigenetically silenced apoptosis-associated tyrosine kinase (AATK) facilitates a decreased expression of Cyclin D1 and WEE1, phosphorylates TP53 and reduces cell proliferation in a kinase-dependent manner
Source: Cancer Gene Ther. 2022 Jul 28;29(12):1975–87. doi: 10.1038/s41417-022-00513-x (PMC9750878; doi:10.1038/s41417-022-00513-x)
Supplement: Supplementary file 6 — Dataset original qPCR [file 41417_2022_513_MOESM6_ESM.zip › U343_ACTB.pdf]

corbett RESEARCH
A Division of Corbett Life Science

14 Hilly Street Mortlake NSW 2137 Australia
T +61 2 9736 1320
F +61 2 9736 1364
W www.corbettlifescience.com

Comparative Quantitation Report

Experiment Information

Run Name	Run 2020-09-18_b-Act_OE-EY_U343_U251_A549_A427_(1)(2)
Run Start	18.09.2020 08:47:00
Run Finish	18.09.2020 10:20:03
Operator	MW
Notes	b-Act OE EY (1)(2) U343 U251 A549 A427 triplicate
Run On Software Version	Rotor-Gene 6.1.93
Run Signature	The Run Signature is valid.
Gain FAM	8.
Gain ROX	9.33

Comparative Quantitation Information

Reaction Amplification	1.68
Reaction Amplification Std. Deviation	0.05
Sample Page	Page 1
Control Replicate	(64)

Take off Graph for Cycling A.FAM/Cycling A.ROX

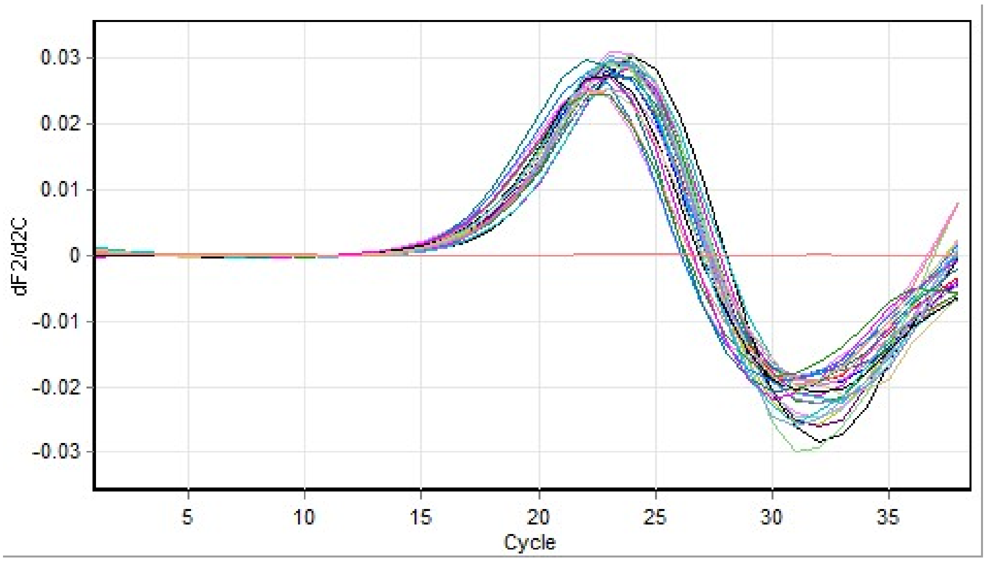

No.	Colour	Name	Take Off	Amplification	Comparative Conc.	Rep. Takeoff	Rep. Takeoff (95% CI)
A1		U343 EY (1)	18.6	1.67	6.32E-05	18.5	[1.$,1.$]
A2		U343 EY (1)	18.5	1.75	6.66E-05		
A3		U343 EY (1)	18.4	1.69	7.01E-05		
A4		U343 B-EY (1)	18.7	1.62	6.00E-05	18.7	[1.$,1.$]
A5		U343 B-EY (1)	18.8	1.66	5.70E-05		
A6		U343 B-EY (1)	18.6	1.70	6.32E-05		
A7		U343 B KD-EY (1)	18.9	1.68	5.41E-05	18.9	[1.$,1.$]
A8		U343 B KD-EY (1)	18.8	1.70	5.70E-05		
B1		U343 B KD-EY (1)	18.9	1.71	5.41E-05		
B2		U343 EY (2)	19.5	1.72	3.96E-05	19.4	[1.$,1.$]
B3		U343 EY (2)	19.4	1.72	4.17E-05		
B4		U343 EY (2)	19.3	1.73	4.39E-05		
B5		U343 B-EY (2)	18.7	1.70	6.00E-05	18.7	[1.$,1.$]
B6		U343 B-EY (2)	18.7	1.73	6.00E-05		
B7		U343 B-EY (2)	18.8	1.67	5.70E-05		
B8		U343 B KD-EY (2)	18.7	1.72	6.00E-05	18.6	[1.$,1.$]
C1		U343 B KD-EY (2)	18.8	1.72	5.70E-05		
C2		U343 B KD-EY (2)	18.4	1.74	7.01E-05		

(Continued on next page)...

No.	Colour	Name	Take Off	Amplification	Comparative Conc.	Rep. Takeoff	Rep. Takeoff (95% CI)
E5		U343 EY (3)	17.6	1.64	1.06E-04	17.7	[1.$,1.$]
E6		U343 EY (3)	17.8	1.66	9.58E-05		
E7		U343 EY (3)	17.7	1.68	1.01E-04		
E8		U343 B-EY (3)	17.8	1.60	9.58E-05	17.8	[1.$,1.$]
F1		U343 B-EY (3)	17.7	1.52	1.01E-04		

F2		U343 B-EY (3)	17.9	1.63	9.10E-05		
F3		U343 B KD-EY (3)	18.0	1.66	8.64E-05	18.2	[1.$,1.$]
F4		U343 B KD-EY (3)	18.3	1.67	7.39E-05		
F5		U343 B KD-EY (3)	18.2	1.72	7.78E-05		
I8		H2O	21.6	0.00	1.33E-05	21.6	

Quality System
Quality Endorsed Company
ISO 9001 Lic 21313
SAI Global

This report generated by Rotor-Gene Real-Time Analysis Software 6.1 (Build 93)
© Corbett Research 2005
® All Rights Reserved
ISO 9001:2000 (Reg. No. QEC21313)
