# Supplementary material for: Epigenetically silenced apoptosis-associated tyrosine kinase (AATK) facilitates a decreased expression of Cyclin D1 and WEE1, phosphorylates TP53 and reduces cell proliferation in a kinase-dependent manner
Source: Cancer Gene Ther. 2022 Jul 28;29(12):1975–87. doi: 10.1038/s41417-022-00513-x (PMC9750878; doi:10.1038/s41417-022-00513-x)
Supplement: Supplementary file 6 — Dataset original qPCR [file 41417_2022_513_MOESM6_ESM.zip › RNAi_WEE1_1.pdf]

corbett RESEARCH
A Division of Corbett Life Science

14 Hilly Street Mortlake NSW 2137 Australia
T +61 2 9736 1320
F +61 2 9736 1364
W www.corbettlifescience.com

Comparative Quantitation Report

Experiment Information

Run Name	Run 2020-10-06_WEE1_RNAi(1)_HEK_SkMel13_MCF7_A427
Run Start	06.10.2020 11:55:39
Run Finish	06.10.2020 13:42:25
Operator	MW
Notes	Wee1 RNAi(1) triplicate
Run On Software Version	Rotor-Gene 6.1.93
Run Signature	The Run Signature is valid.
Gain FAM	8.
Gain ROX	9.33

Comparative Quantitation Information

Reaction Amplification	1.70
Reaction Amplification Std. Deviation	0.03
Sample Page	Page 1
Control Replicate	(4)

Take off Graph for Cycling A.FAM/Cycling A.ROX

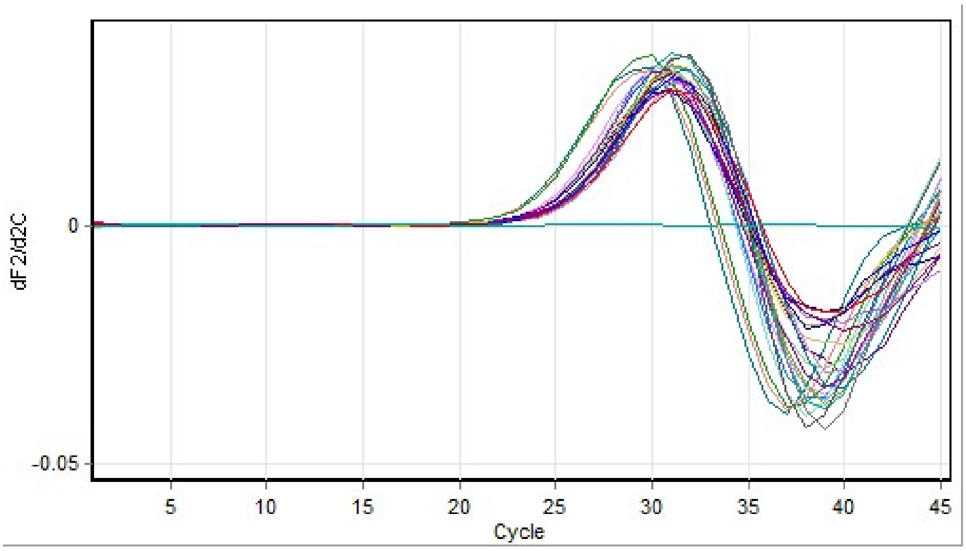

No.	Colour	Name	Take Off	Amplification	Comparative Conc.	Rep. Takeoff	Rep. Takeoff (95% CI)
A4		HEK siCtrl (1)	26.0	1.71	9.65E-01	25.9	[1.$,1.$]
A5		HEK siCtrl (1)	25.9	1.72	1.02E+00		
A6		HEK siCtrl (1)	25.9	1.72	1.02E+00		
A7		HEK siAATK (1)	24.6	1.72	2.03E+00	24.7	[1.$,1.$]
A8		HEK siAATK (1)	24.7	1.75	1.92E+00		
B1		HEK siAATK (1)	24.9	1.70	1.73E+00		
B5		HEK siAATK (2)	25.9	1.69	1.02E+00	26.0	[1.$,1.$]
B6		HEK siAATK (2)	26.0	1.67	9.65E-01		
B7		HEK siAATK (2)	26.0	1.72	9.65E-01		
B8		HEK siCtrl (2)	26.6	1.70	7.03E-01	26.6	[1.$,1.$]
C1		HEK siCtrl (2)	26.5	1.69	7.41E-01		
C2		HEK siCtrl (2)	26.8	1.65	6.32E-01		
C6		SkMel13 siCtrl (1)	26.6	1.66	7.03E-01	26.6	[1.$,1.$]
C7		SkMel13 siCtrl (1)	26.6	1.71	7.03E-01		
C8		SkMel13 siCtrl (1)	26.7	1.70	6.66E-01		
D1		SkMel13 siAATK (1)	26.0	1.64	9.65E-01	26.1	[1.$,1.$]
D2		SkMel13 siAATK (1)	26.0	1.69	9.65E-01		
D3		SkMel13 siAATK (1)	26.2	1.70	8.68E-01		

(Continued on next page)...

No.	Colour	Name	Take Off	Amplification	Comparative Conc.	Rep. Takeoff	Rep. Takeoff (95% CI)
D7		SkMel13 siCtrl (2)	27.0	1.65	5.69E-01	26.8	[1.$,1.$]
D8		SkMel13 siCtrl (2)	26.9	1.67	5.99E-01		
E1		SkMel13 siCtrl (2)	26.4	1.70	7.81E-01		
E2		SkMel13 siAATK (2)	26.3	1.75	8.24E-01	26.4	[1.$,1.$]
E3		SkMel13 siAATK (2)	26.5	1.72	7.41E-01		
E4		SkMel13 siAATK (2)	26.3	1.72	8.24E-01		

G7		H2O	15.1	0.00	3.09E+02	15.1	

This report generated by Rotor-Gene Real-Time Analysis Software 6.1 (Build 93)
© Corbett Research 2005
® All Rights Reserved
ISO 9001:2000 (Reg. No. QEC21313)
