# Supplementary material for: Epigenetically silenced apoptosis-associated tyrosine kinase (AATK) facilitates a decreased expression of Cyclin D1 and WEE1, phosphorylates TP53 and reduces cell proliferation in a kinase-dependent manner
Source: Cancer Gene Ther. 2022 Jul 28;29(12):1975–87. doi: 10.1038/s41417-022-00513-x (PMC9750878; doi:10.1038/s41417-022-00513-x)
Supplement: Supplementary file 6 — Dataset original qPCR [file 41417_2022_513_MOESM6_ESM.zip › RNAi_WEE1_3.pdf]

corbett RESEARCH
A Division of Corbett Life Science

14 Hilly Street Mortlake NSW 2137 Australia
T +61 2 9736 1320
F +61 2 9736 1364
W www.corbettlifescience.com

Comparative Quantitation Report

Experiment Information

Run Name	Run 2020-09-25_WEE1_NOXA_HEK-RNAi(2)(3)
Run Start	25.09.2020 11:12:05
Run Finish	25.09.2020 13:31:39
Operator	MW
Notes	Noxa HEK RNAi (2)(3) HEK OE EY UV (1)(2) triplicate
Run On Software Version	Rotor-Gene 6.1.93
Run Signature	The Run Signature is valid.
Gain FAM	8.
Gain ROX	9.33

Comparative Quantitation Information

Reaction Amplification	1.59
Reaction Amplification Std. Deviation	0.02
Sample Page	Page 1
Control Replicate	(7)

Take off Graph for Cycling A.FAM/Cycling A.ROX

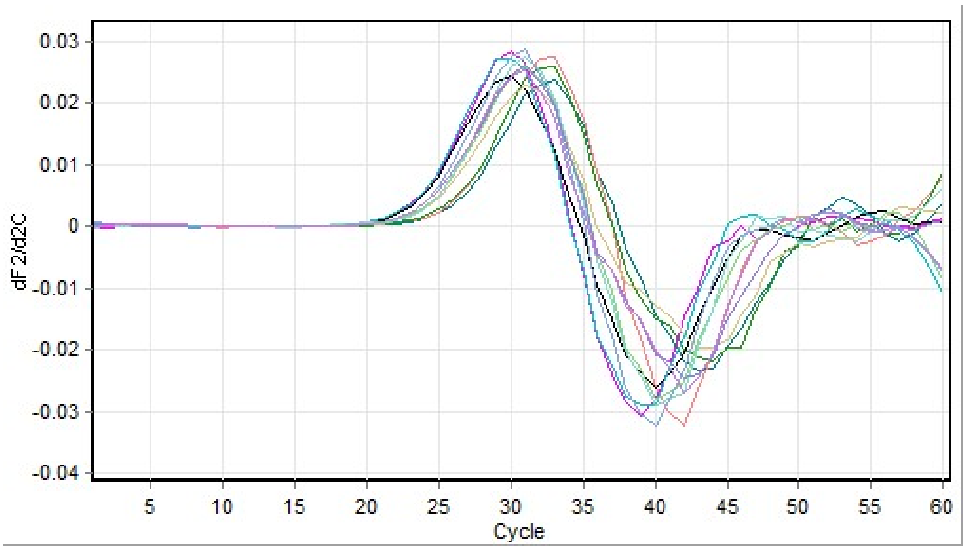

No.	Colour	Name	Take Off	Amplification	Comparative Conc.	Rep. Takeoff	Rep. Takeoff (95% CI)
A7		HEK siCtrl 24h (3)	27.3	1.60	9.69E-01	27.2	[1.$,1.$]
A8		HEK siCtrl 24h (3)	27.3	1.58	9.69E-01		
B1		HEK siCtrl 24h (3)	27.1	1.52	1.06E+00		
B2		HEK siAATK 24h (3)	24.8	1.54	3.10E+00	24.7	[1.$,1.$]
B3		HEK siAATK 24h (3)	24.5	1.62	3.56E+00		
B4		HEK siAATK 24h (3)	24.7	1.59	3.25E+00		
B5		HEK siCtrl 48h (3)	25.6	1.58	2.14E+00	25.7	[1.$,1.$]
B6		HEK siCtrl 48h (3)	25.7	1.59	2.04E+00		
B7		HEK siCtrl 48h (3)	25.8	1.60	1.95E+00		
B8		HEK siAATK 48h (3)	25.5	1.63	2.24E+00	25.4	[1.$,1.$]
C1		HEK siAATK 48h (3)	25.4	1.58	2.34E+00		
C2		HEK siAATK 48h (3)	25.3	1.59	2.46E+00		

Quality System
Quality Endorsed Company
ISO 9001 Lic 21313
SAI Global

™ This report generated by Rotor-Gene Real-Time Analysis Software 6.1 (Build 93)
© Corbett Research 2005
® All Rights Reserved
ISO 9001:2000 (Reg. No. QEC21313)
