# Supplementary material for: Epigenetically silenced apoptosis-associated tyrosine kinase (AATK) facilitates a decreased expression of Cyclin D1 and WEE1, phosphorylates TP53 and reduces cell proliferation in a kinase-dependent manner
Source: Cancer Gene Ther. 2022 Jul 28;29(12):1975–87. doi: 10.1038/s41417-022-00513-x (PMC9750878; doi:10.1038/s41417-022-00513-x)
Supplement: Supplementary file 6 — Dataset original qPCR [file 41417_2022_513_MOESM6_ESM.zip › GAPDH_PaTu-T.pdf]

14 Hilly Street Mortlake NSW 2137 Australia
T +61 2 9736 1320
F +61 2 9736 1364
W www.corbettlifescience.com

Comparative Quantitation Report

Experiment Information

Run Name	Run 2016-12-21_GAPDH_Aza_Pankreas
Run Start	21.12.2016 17:01:12
Run Finish	21.12.2016 18:13:45
Operator	MW
Notes	GAPDH Aza Pankeas triplicate
Run On Software Version	Rotor-Gene 6.1.93
Run Signature	The Run Signature is valid.
Gain FAM	8.
Gain ROX	8.

Comparative Quantitation Information

Reaction Amplification	1.76
Reaction Amplification Std. Deviation	0.02
Sample Page	Page 1
Control Replicate	(28)

Take off Graph for Cycling A.FAM/Cycling A.ROX

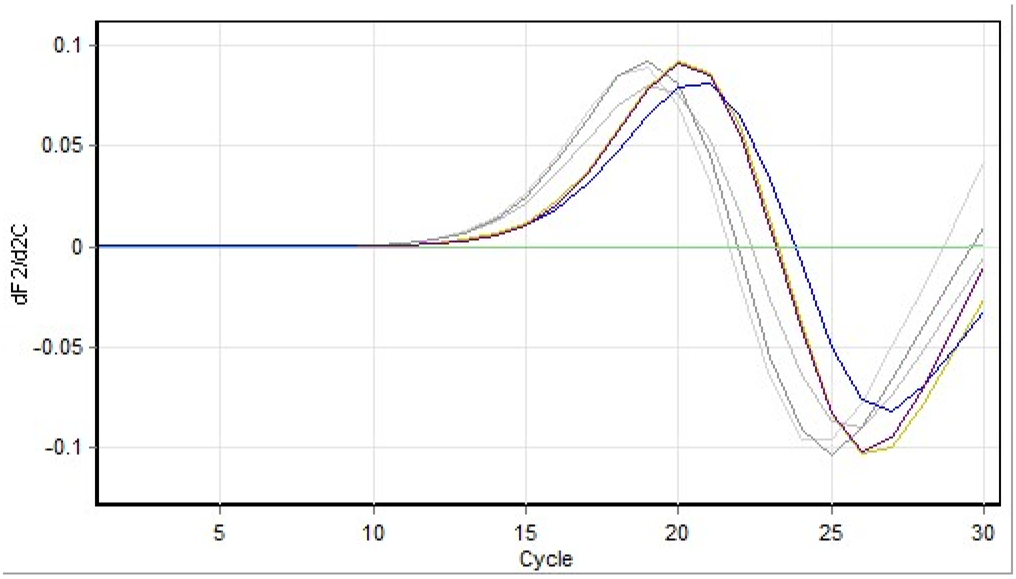

No.	Colour	Name	Take Off	Amplification	Comparative Conc.	Rep. Takeoff	Rep. Takeoff (95% CI)
D4		PaTu-T 0uM	15.0	1.79	1.04E+00	15.1	[1.$,1.$]
D5		PaTu-T 0uM	15.1	1.74	9.81E-01		
D6		PaTu-T 0uM	15.1	1.77	9.81E-01		
E2		PaTu-T 5uM	16.3	1.75	4.99E-01	16.4	[1.$,1.$]
E3		PaTu-T 5uM	16.4	1.72	4.72E-01		
E4		PaTu-T 5uM	16.4	1.76	4.72E-01		
F6		H2O	23.8	0.00	7.30E-03	23.8	

Quality System™
Quality Endorsed Company
ISO 9001 Lic 21313
SAI Global

This report generated by Rotor-Gene Real-Time Analysis Software 6.1 (Build 93)
© Corbett Research 2005
® All Rights Reserved
ISO 9001:2000 (Reg. No. QEC21313)
