# Supplementary material for: Epigenetically silenced apoptosis-associated tyrosine kinase (AATK) facilitates a decreased expression of Cyclin D1 and WEE1, phosphorylates TP53 and reduces cell proliferation in a kinase-dependent manner
Source: Cancer Gene Ther. 2022 Jul 28;29(12):1975–87. doi: 10.1038/s41417-022-00513-x (PMC9750878; doi:10.1038/s41417-022-00513-x)
Supplement: Supplementary file 6 — Dataset original qPCR [file 41417_2022_513_MOESM6_ESM.zip › ANXA1_clone pools.pdf]

corbett RESEARCH
A Division of Corbett Life Science

14 Hilly Street Mortlake NSW 2137 Australia
T +61 2 9736 1320
F +61 2 9736 1364
W www.corbettlifescience.com

Comparative Quantitation Report

Experiment Information

Run Name	Run 2020-04-04_ANXA1_AFFY_HCT-OE-starved
Run Start	04.04.2020 11:46:30
Run Finish	04.04.2020 13:31:16
Operator	MW
Notes	ANXA1 Affy cDNA HCT OE starved triplicate
Run On Software Version	Rotor-Gene 6.1.93
Run Signature	The Run Signature is valid.
Gain FAM	8.
Gain ROX	8.

Comparative Quantitation Information

Reaction Amplification	1.68
Reaction Amplification Std. Deviation	0.03
Sample Page	Page 1
Control Replicate	(1)

Take off Graph for Cycling A.FAM

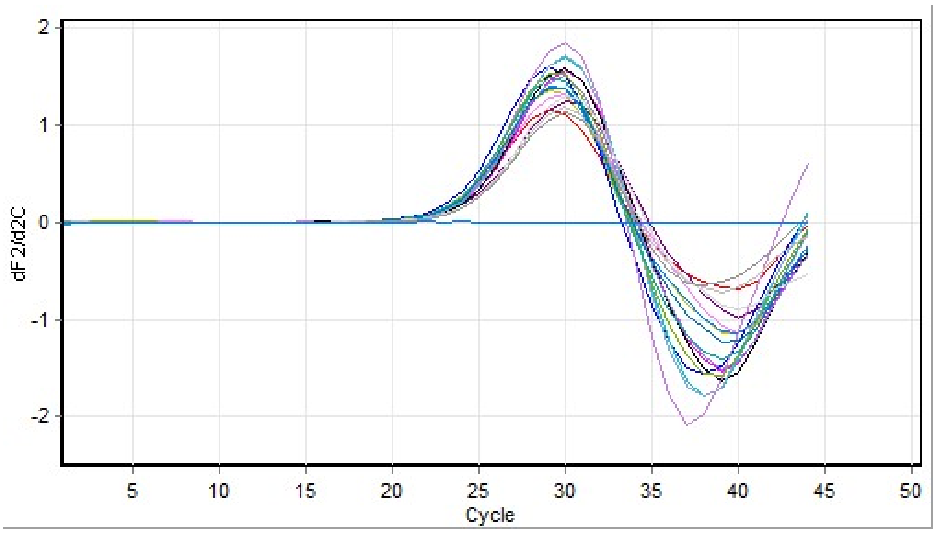

No.	Colour	Name	Take Off	Amplification	Comparative Conc.	Rep. Takeoff	Rep. Takeoff (95% CI)
A1		Control clone pool (1)	24.5	1.65	1.04E+00	24.6	[1.$,1.$]
A2		Control clone pool (1)	24.6	1.68	9.83E-01		
A3		Control clone pool (1)	24.6	1.66	9.83E-01		
A4		Control clone pool (2)	25.2	1.61	7.19E-01	25.0	[1.$,1.$]
A5		Control clone pool (2)	25.0	1.66	7.98E-01		
A6		Control clone pool (2)	24.9	1.71	8.40E-01		
B2		Clone pool AATK (1)	25.4	1.69	6.48E-01	25.4	[1.$,1.$]
B3		Clone pool AATK (1)	25.4	1.73	6.48E-01		
B4		Clone pool AATK (1)	25.3	1.72	6.82E-01		
B8		Clone pool AATK KD (1)	25.4	1.69	6.48E-01	25.3	[1.$,1.$]
C1		Clone pool AATK KD (1)	25.1	1.65	7.57E-01		
C2		Clone pool AATK KD (1)	25.3	1.71	6.82E-01		
C6		Clone pool AATK (2)	24.9	1.70	8.40E-01	24.8	[1.$,1.$]
C7		Clone pool AATK (2)	24.8	1.68	8.85E-01		
C8		Clone pool AATK (2)	24.8	1.66	8.85E-01		
D4		Clone pool AATK KD (2)	25.5	1.70	6.15E-01	25.4	[1.$,1.$]
D5		Clone pool AATK KD (2)	25.3	1.69	6.82E-01		
D6		Clone pool AATK KD (2)	25.3	1.72	6.82E-01		
I6		H2O	19.7	0.91	1.26E+01	19.7	

Quality System
Quality Endorsed Company
ISO 9001 Lic 21313
SAI Global

This report generated by Rotor-Gene Real-Time Analysis Software 6.1 (Build 93)
© Corbett Research 2005
® All Rights Reserved
ISO 9001:2000 (Reg. No. QEC21313)
