# Supplementary material for: Epigenetically silenced apoptosis-associated tyrosine kinase (AATK) facilitates a decreased expression of Cyclin D1 and WEE1, phosphorylates TP53 and reduces cell proliferation in a kinase-dependent manner
Source: Cancer Gene Ther. 2022 Jul 28;29(12):1975–87. doi: 10.1038/s41417-022-00513-x (PMC9750878; doi:10.1038/s41417-022-00513-x)
Supplement: Supplementary file 6 — Dataset original qPCR [file 41417_2022_513_MOESM6_ESM.zip › RNAi_WEE1_2.pdf]

14 Hilly Street Mortlake NSW 2137 Australia
T +61 2 9736 1320
F +61 2 9736 1364
W www.corbettlifescience.com

Comparative Quantitation Report

Experiment Information

Run Name	Run 2020-10-18_Wee1_RNAi(2)_EG mit Selektion für Lysate
Run Start	18.03.2021 08:11:28
Run Finish	18.03.2021 09:57:06
Operator	MW
Notes	Wee1 RNAi(2)_EG für Lysate triplicate
Run On Software Version	Rotor-Gene 6.1.93
Run Signature	The Run Signature is valid.
Gain FAM	8.
Gain ROX	9.33

Comparative Quantitation Information

Reaction Amplification	1.62
Reaction Amplification Std. Deviation	0.05
Sample Page	Page 1
Control Replicate	(4)

Take off Graph for Cycling A.FAM/Cycling A.ROX

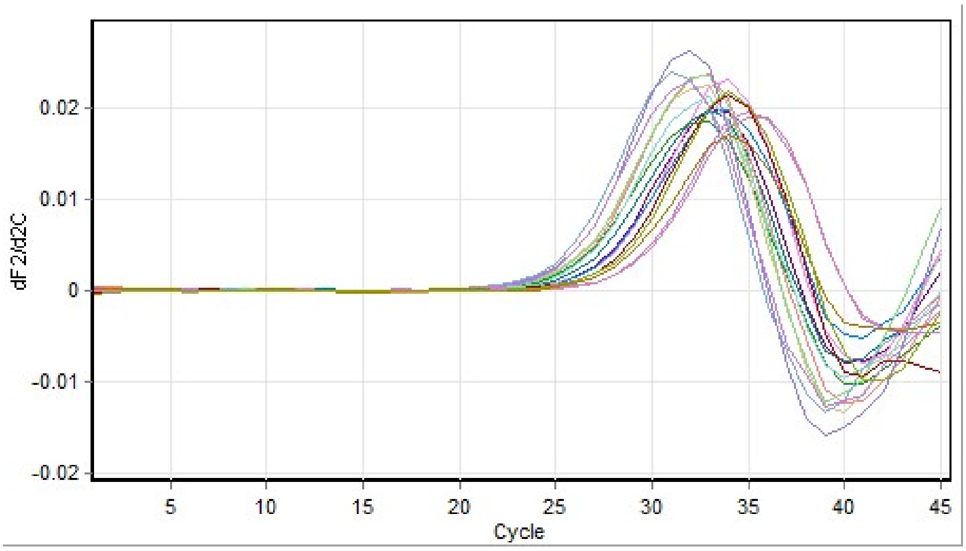

No.	Colour	Name	Take Off	Amplification	Comparative Conc.	Rep. Takeoff	Rep. Takeoff (95% CI)
A4		MCF-7 siCtrl (2)	28.5	1.41	1.12E+00	28.7	[1.$,1.$]
A5		MCF-7 siCtrl (2)	29.1	1.58	8.38E-01		
A6		MCF-7 siCtrl (2)	28.6	1.43	1.07E+00		
A7		MCF-7 siAATK (2)	27.8	1.54	1.57E+00	27.5	[1.$,1.$]
A8		MCF-7 siAATK (2)	27.5	1.66	1.81E+00		
B1		MCF-7 siAATK (2)	27.1	1.60	2.20E+00		
B5		MCF-7 siCtrl (3)	27.1	1.57	2.20E+00	27.3	[1.$,1.$]
B6		MCF-7 siCtrl (3)	27.4	1.64	1.90E+00		
B7		MCF-7 siCtrl (3)	27.5	1.69	1.81E+00		
B8		MCF-7 siAATK (3)	26.4	1.67	3.08E+00	26.7	[1.$,1.$]
C1		MCF-7 siAATK (3)	27.0	1.62	2.31E+00		
C2		MCF-7 siAATK (3)	26.8	1.53	2.54E+00		
G2		SkMel13 siCtrl (3)	30.0	1.64	5.43E-01	30.0	
G3		SkMel13 siCtrl (3)	30.0	1.63	5.43E-01		
G4		SkMel13 siAATK (3)	29.1	1.64	8.38E-01	29.3	[1.$,1.$]
G5		SkMel13 siAATK (3)	29.2	1.63	7.98E-01		
G6		SkMel13 siAATK (3)	29.5	1.67	6.91E-01		

(Continued on next page)...

Quality System
Quality Endorsed Company
ISO 9001 Lic 21313
SAI Global

™ This report generated by Rotor-Gene Real-Time Analysis Software 6.1 (Build 93)
© Corbett Research 2005
® All Rights Reserved
ISO 9001:2000 (Reg. No. QEC21313)
