# Supplementary material for: Epigenetically silenced apoptosis-associated tyrosine kinase (AATK) facilitates a decreased expression of Cyclin D1 and WEE1, phosphorylates TP53 and reduces cell proliferation in a kinase-dependent manner
Source: Cancer Gene Ther. 2022 Jul 28;29(12):1975–87. doi: 10.1038/s41417-022-00513-x (PMC9750878; doi:10.1038/s41417-022-00513-x)
Supplement: Supplementary file 6 — Dataset original qPCR [file 41417_2022_513_MOESM6_ESM.zip › AATK_LNZ308.pdf]

corbett RESEARCH
A Division of Corbett Life Science

14 Hilly Street Mortlake NSW 2137 Australia
T + 61 2 9736 1320
F + 61 2 9736 1364
W www.corbettlifescience.com

Comparative Quantitation Report

Experiment Information

Run Name	Run 2016-09-28_AATK(4ul)_Aza_Glio_AATK
Run Start	28.09.2016 15:08:11
Run Finish	28.09.2016 17:17:31
Operator	MW
Notes	AATK C81/MeWo<-p53_Noco_Cisp LZ308/U87<-Aza triplicate
Run On Software Version	Rotor-Gene 6.1.93
Run Signature	The Run Signature is valid.
Gain FAM	8.
Gain ROX	8.

Comparative Quantitation Information

Reaction Amplification	1.69
Reaction Amplification Std. Deviation	0.02
Sample Page	Page 1
Control Replicate	(1)

Take off Graph for Cycling A.FAM

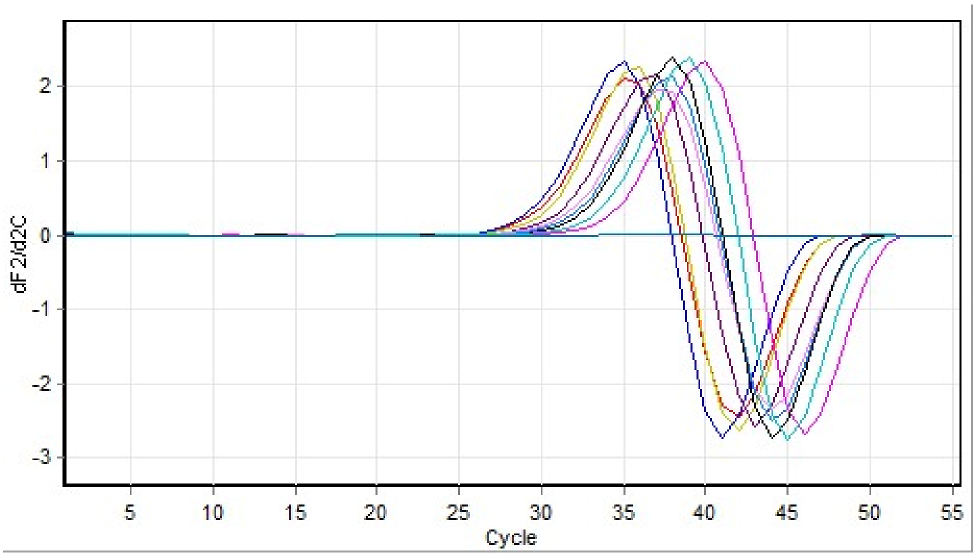

No.	Colour	Name	Take Off	Amplification	Comparative Conc.	Rep. Takeoff	Rep. Takeoff (95% CI)
A1		LNZ308 10 um Aza	30.9	1.68	1.05E+00	31.0	[1.$,1.$]
A2		LNZ308 10 um Aza	31.4	1.69	8.11E-01		
A3		LNZ308 10 um Aza	30.7	1.67	1.17E+00		
A4		LNZ308 5 um Aza	32.3	1.68	5.07E-01	32.8	[1.$,1.$]
A5		LNZ308 5 um Aza	32.8	1.64	3.90E-01		
A6		LNZ308 5 um Aza	33.3	1.71	3.00E-01		
B2		LNZ308 0 um Aza	35.7	1.72	8.56E-02	34.8	[1.$,1.$]
B3		LNZ308 0 um Aza	33.9	1.70	2.19E-01		
B4		LNZ308 0 um Aza	34.7	1.68	1.44E-01		
G8		H2O	35.9	0.03	7.71E-02	35.9	

Quality System
Quality Endorsed Company
ISO 9001 Lic 21313
SAI Global

™ This report generated by Rotor-Gene Real-Time Analysis Software 6.1 (Build 93)
© Corbett Research 2005
® All Rights Reserved
ISO 9001:2000 (Reg. No. QEC21313)
