# Supplementary material for: Epigenetically silenced apoptosis-associated tyrosine kinase (AATK) facilitates a decreased expression of Cyclin D1 and WEE1, phosphorylates TP53 and reduces cell proliferation in a kinase-dependent manner
Source: Cancer Gene Ther. 2022 Jul 28;29(12):1975–87. doi: 10.1038/s41417-022-00513-x (PMC9750878; doi:10.1038/s41417-022-00513-x)
Supplement: Supplementary file 6 — Dataset original qPCR [file 41417_2022_513_MOESM6_ESM.zip › RNAi_CCND1_3.pdf]

14 Hilly Street Mortlake NSW 2137 Australia
T +61 2 9736 1320
F +61 2 9736 1364
W www.corbettlifescience.com

Comparative Quantitation Report

Experiment Information

Run Name	Run 2020-06-03_CCND1_RNAi HEK(2)(3)_HIPK OE starved
Run Start	03.06.2020 15:30:38
Run Finish	03.06.2020 17:26:52
Operator	MW
Notes	CCND1 RNAi MCF-7,A427;SkMel13 (2) triplicate
Run On Software Version	Rotor-Gene 6.1.93
Run Signature	The Run Signature is valid.
Gain FAM	8.
Gain ROX	9.33

Comparative Quantitation Information

Reaction Amplification	1.70
Reaction Amplification Std. Deviation	0.03
Sample Page	Page 1
Control Replicate	(7)

Take off Graph for Cycling A.FAM/Cycling A.ROX

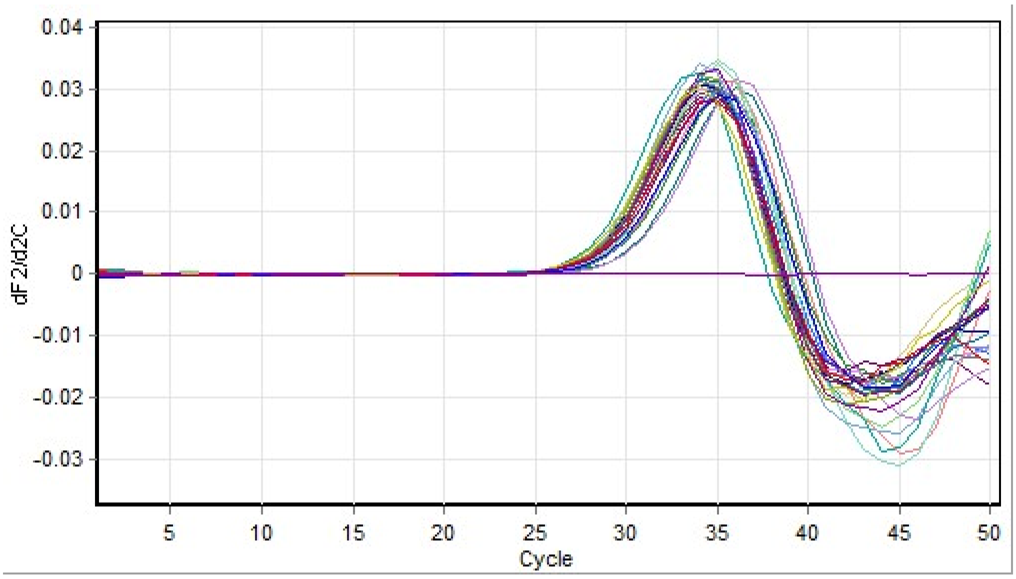

No.	Colour	Name	Take Off	Amplification	Comparative Conc.	Rep. Takeoff	Rep. Takeoff (95% CI)
A4		siAATK 24 h (2)	30.1	1.69	1.67E+00	30.1	[1.$,1.$]
A5		siAATK 24 h (2)	29.9	1.68	1.85E+00		
A6		siAATK 24 h (2)	30.3	1.71	1.50E+00		
A7		siCtrl 24 h (2)	31.4	1.70	8.38E-01	31.1	[1.$,1.$]
A8		siCtrl 24 h (2)	30.9	1.76	1.09E+00		
B1		siCtrl 24 h (2)	30.9	1.70	1.09E+00		
B5		siAATK 48 h (2)	30.1	1.64	1.67E+00	30.4	[1.$,1.$]
B6		siAATK 48 h (2)	30.6	1.73	1.28E+00		
B7		siAATK 48 h (2)	30.5	1.73	1.35E+00		
B8		siCtrl 48 h (2)	29.8	1.69	1.95E+00	30.8	[1.$,1.$]
C1		siCtrl 48 h (2)	30.8	1.70	1.15E+00		
C2		siCtrl 48 h (2)	31.8	1.68	6.79E-01		
C6		siAATK 24 h (3)	29.8	1.68	1.95E+00	29.7	[1.$,1.$]
C7		siAATK 24 h (3)	29.3	1.73	2.54E+00		
C8		siAATK 24 h (3)	30.0	1.74	1.76E+00		
D1		siCtrl 24 h (3)	29.9	1.65	1.85E+00	29.9	[1.$,1.$]
D2		siCtrl 24 h (3)	30.0	1.67	1.76E+00		
D3		siCtrl 24 h (3)	29.9	1.69	1.85E+00		

(Continued on next page)...

No.	Colour	Name	Take Off	Amplification	Comparative Conc.	Rep. Takeoff	Rep. Takeoff (95% CI)
D7		siAATK48 h (3)	29.8	1.72	1.95E+00	29.9	[1.$,1.$]
D8		siAATK48 h (3)	29.9	1.69	1.85E+00		
E1		siAATK48 h (3)	29.9	1.66	1.85E+00		
E2		siCtrl 48 h (3)	29.4	1.67	2.41E+00	30.0	[1.$,1.$]

E3		siCtrl 48 h (3)	30.5	1.70	1.35E+00		
E4		siCtrl 48 h (3)	30.1	1.71	1.67E+00		
H2		H2O	34.1	0.00	2.01E-01	34.1	

This report generated by Rotor-Gene Real-Time Analysis Software 6.1 (Build 93)
© Corbett Research 2005
® All Rights Reserved
ISO 9001:2000 (Reg. No. QEC21313)
