# Supplementary material for: Epigenetically silenced apoptosis-associated tyrosine kinase (AATK) facilitates a decreased expression of Cyclin D1 and WEE1, phosphorylates TP53 and reduces cell proliferation in a kinase-dependent manner
Source: Cancer Gene Ther. 2022 Jul 28;29(12):1975–87. doi: 10.1038/s41417-022-00513-x (PMC9750878; doi:10.1038/s41417-022-00513-x)
Supplement: Supplementary file 6 — Dataset original qPCR [file 41417_2022_513_MOESM6_ESM.zip › RNAi_ACTB_3.pdf]

corbett RESEARCH
A Division of Corbett Life Science

14 Hilly Street Mortlake NSW 2137 Australia
T +61 2 9736 1320
F +61 2 9736 1364
W www.corbettlifescience.com

Comparative Quantitation Report

Experiment Information

Run Name	Run 2020-06-07_b-Act_RNAi HEK (2)_(3);UV HEK (1)_(2)
Run Start	07.06.2020 13:11:27
Run Finish	07.06.2020 14:45:28
Operator	MW
Notes	b-Act RNAi HEK (2) (3), UV HEK (1) (2) triplicate
Run On Software Version	Rotor-Gene 6.1.93
Run Signature	The Run Signature is valid.
Gain FAM	8.
Gain ROX	9.33

Comparative Quantitation Information

Reaction Amplification	1.68
Reaction Amplification Std. Deviation	0.02
Sample Page	Page 1
Control Replicate	(4)

Take off Graph for Cycling A.FAM/Cycling A.ROX

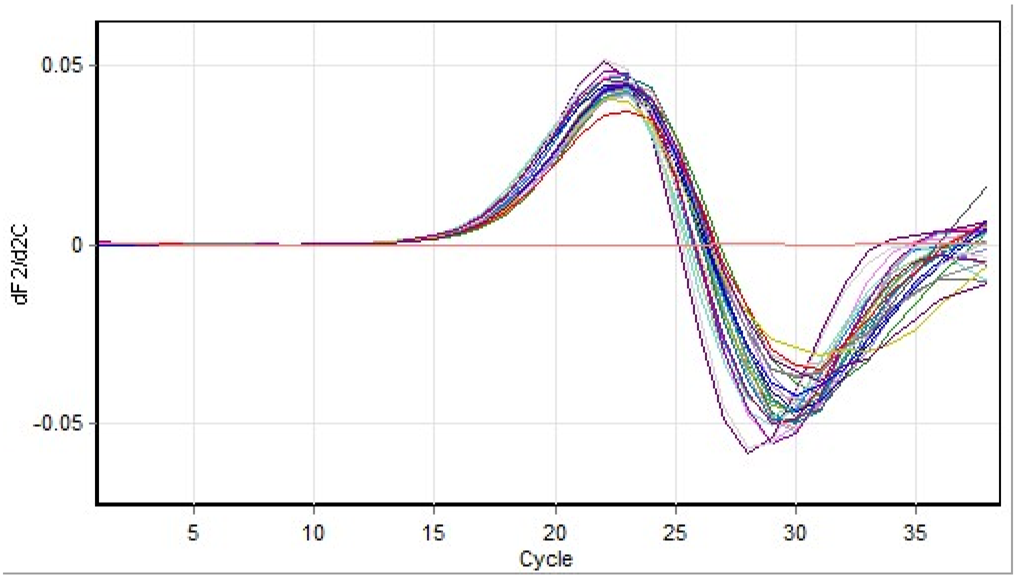

No.	Colour	Name	Take Off	Amplification	Comparative Conc.	Rep. Takeoff	Rep. Takeoff (95% CI)
A4		siCtrl 24 h (2)	18.2	1.71	9.83E-01	18.2	[1.$,1.$]
A5		siCtrl 24 h (2)	18.2	1.70	9.83E-01		
A6		siCtrl 24 h (2)	18.1	1.72	1.04E+00		
A7		siAATK 24 h (2)	18.8	1.70	7.20E-01	18.7	[1.$,1.$]
A8		siAATK 24 h (2)	18.7	1.69	7.58E-01		
B1		siAATK 24 h (2)	18.7	1.71	7.58E-01		
B5		siCtrl 48 h (2)	17.7	1.69	1.27E+00	17.7	[1.$,1.$]
B6		siCtrl 48 h (2)	17.8	1.68	1.21E+00		
B7		siCtrl 48 h (2)	17.7	1.68	1.27E+00		
B8		siAATK 48 h (2)	18.4	1.67	8.86E-01	18.4	[1.$,1.$]
C1		siAATK 48 h (2)	18.4	1.66	8.86E-01		
C2		siAATK 48 h (2)	18.4	1.70	8.86E-01		
C6		siCtrl 24 h (3)	18.2	1.64	9.83E-01	18.2	[1.$,1.$]
C7		siCtrl 24 h (3)	18.2	1.66	9.83E-01		
C8		siCtrl 24 h (3)	18.2	1.68	9.83E-01		
D1		siAATK 24 h (3)	17.8	1.73	1.21E+00	17.9	[1.$,1.$]
D2		siAATK 24 h (3)	17.9	1.70	1.15E+00		
D3		siAATK 24 h (3)	17.9	1.69	1.15E+00		

(Continued on next page)...

No.	Colour	Name	Take Off	Amplification	Comparative Conc.	Rep. Takeoff	Rep. Takeoff (95% CI)
D4		ohne 48 h (3)	18.3	1.69	9.33E-01	18.3	[1.$,1.$]
D5		ohne 48 h (3)	18.3	1.68	9.33E-01		
D6		ohne 48 h (3)	18.2	1.66	9.83E-01		
D7		siCtrl 48 h (3)	18.2	1.65	9.83E-01	18.3	[1.$,1.$]

D8		siCtrl 48 h (3)	18.4	1.67	8.86E-01		
E1		siCtrl 48 h (3)	18.2	1.64	9.83E-01		
E2		siAATK 48 h (3)	18.3	1.68	9.33E-01	18.3	[1.$,1.$]
E3		siAATK 48 h (3)	18.3	1.67	9.33E-01		
E4		siAATK 48 h (3)	18.4	1.66	8.86E-01		
I8		H2O	33.4	0.00	3.65E-04	33.4	

Quality System ™
Quality Endorsed Company
ISO 9001 Lic 21313
SAI Global

This report generated by Rotor-Gene Real-Time Analysis Software 6.1 (Build 93)
© Corbett Research 2005
® All Rights Reserved
ISO 9001:2000 (Reg. No. QEC21313)
