# Supplementary material for: Epigenetically silenced apoptosis-associated tyrosine kinase (AATK) facilitates a decreased expression of Cyclin D1 and WEE1, phosphorylates TP53 and reduces cell proliferation in a kinase-dependent manner
Source: Cancer Gene Ther. 2022 Jul 28;29(12):1975–87. doi: 10.1038/s41417-022-00513-x (PMC9750878; doi:10.1038/s41417-022-00513-x)
Supplement: Supplementary file 6 — Dataset original qPCR [file 41417_2022_513_MOESM6_ESM.zip › HEK_ANXA1.pdf]

corbett RESEARCH
A Division of Corbett Life Science

14 Hilly Street Mortlake NSW 2137 Australia
T +61 2 9736 1320
F +61 2 9736 1364
W www.corbettlifescience.com

Comparative Quantitation Report

Experiment Information

Run Name	Run 2020-06-07_ANXA1_RNAi HEK (2)_(3);UV HEK (1)_(2)
Run Start	07.06.2020 15:06:35
Run Finish	07.06.2020 17:06:45
Operator	MW
Notes	ANXA1 RNAi HEK (2) (3); UV (1) (2) triplicate
Run On Software Version	Rotor-Gene 6.1.93
Run Signature	The Run Signature is valid.
Gain FAM	8.
Gain ROX	9.33

Comparative Quantitation Information

Reaction Amplification	1.65
Reaction Amplification Std. Deviation	0.06
Sample Page	Page 1
Control Replicate	(37)

Take off Graph for Cycling A.FAM/Cycling A.ROX

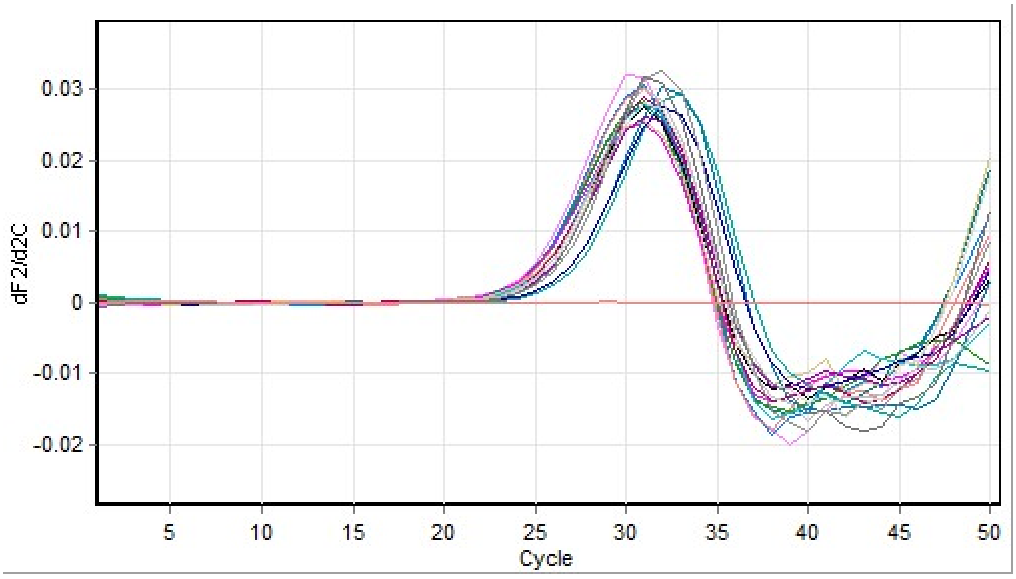

No.	Colour	Name	Take Off	Amplification	Comparative Conc.	Rep. Takeoff	Rep. Takeoff (95% CI)
E5		ohne EY (2)	26.0	1.64	9.35E-01	25.9	[1.$,1.$]
E6		ohne EY (2)	26.0	1.68	9.35E-01		
E7		ohne EY (2)	25.6	1.65	1.14E+00		
E8		ohne B (2)	26.0	1.63	9.35E-01	25.9	[1.$,1.$]
F1		ohne B (2)	26.1	1.64	8.89E-01		
F2		ohne B (2)	25.6	1.52	1.14E+00		
F3		ohne B KD (2)	26.2	1.60	8.46E-01	26.2	[1.$,1.$]
F4		ohne B KD (2)	26.2	1.73	8.46E-01		
F5		ohne B KD (2)	26.1	1.57	8.89E-01		
G7		ohne EY (3)	28.0	1.63	3.42E-01	27.9	[1.$,1.$]
G8		ohne EY (3)	27.9	1.61	3.59E-01		
H1		ohne EY (3)	27.8	1.65	3.78E-01		
H2		ohne B (3)	26.6	1.74	6.91E-01	26.5	[1.$,1.$]
H3		ohne B (3)	26.3	1.71	8.04E-01		
H4		ohne B (3)	26.7	1.67	6.57E-01		
H5		ohne B KD (3)	26.7	1.70	6.57E-01	27.0	[1.$,1.$]
H6		ohne B KD (3)	27.2	1.69	5.11E-01		
H7		ohne B KD (3)	27.1	1.72	5.38E-01		
I8		H2O	16.1	0.00	1.36E+02	16.1	

Quality System
Quality Endorsed Company
ISO 9001 Lic 21313
SAI Global

This report generated by Rotor-Gene Real-Time Analysis Software 6.1 (Build 93)
© Corbett Research 2005
® All Rights Reserved
ISO 9001:2000 (Reg. No. QEC21313)
