# Supplementary material for: Epigenetically silenced apoptosis-associated tyrosine kinase (AATK) facilitates a decreased expression of Cyclin D1 and WEE1, phosphorylates TP53 and reduces cell proliferation in a kinase-dependent manner
Source: Cancer Gene Ther. 2022 Jul 28;29(12):1975–87. doi: 10.1038/s41417-022-00513-x (PMC9750878; doi:10.1038/s41417-022-00513-x)
Supplement: Supplementary file 6 — Dataset original qPCR [file 41417_2022_513_MOESM6_ESM.zip › RNAi_ACTB_2.pdf]

corbett RESEARCH
A Division of Corbett Life Science

14 Hilly Street Mortlake NSW 2137 Australia
T +61 2 9736 1320
F +61 2 9736 1364
W www.corbettlifescience.com

Comparative Quantitation Report

Experiment Information

Run Name	Run 2020-06-04_b-Act_RNAi-MCF7,A427,SkMel13_(2)
Run Start	04.06.2020 12:58:53
Run Finish	04.06.2020 14:33:27
Operator	MW
Notes	b-Act RNAi MCF-7,A427,SkMel13 (2) triplicate
Run On Software Version	Rotor-Gene 6.1.93
Run Signature	The Run Signature is valid.
Gain FAM	8.
Gain ROX	9.33

Comparative Quantitation Information

Reaction Amplification	1.64
Reaction Amplification Std. Deviation	0.03
Sample Page	Page 1
Control Replicate	(38)

Take off Graph for Cycling A.FAM/Cycling A.ROX

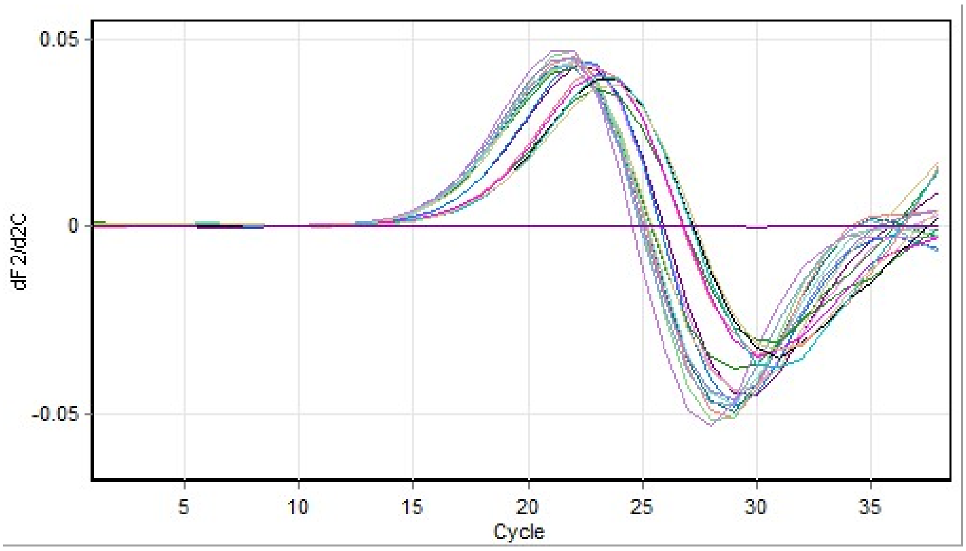

No.	Colour	Name	Take Off	Amplification	Comparative Conc.	Rep. Takeoff	Rep. Takeoff (95% CI)
A4		MCF-7 siCtrl (2)	17.9	1.65	1.51E-04	17.9	[1.$,1.$]
A5		MCF-7 siCtrl (2)	17.9	1.68	1.51E-04		
A6		MCF-7 siCtrl (2)	17.9	1.64	1.51E-04		
A7		MCF-7 siAATK (2)	17.2	1.65	2.13E-04	17.2	[1.$,1.$]
A8		MCF-7 siAATK (2)	17.2	1.64	2.13E-04		
B1		MCF-7 siAATK (2)	17.2	1.65	2.13E-04		
B5		MCF-7 siCtrl (3)	17.2	1.67	2.13E-04	17.2	[1.$,1.$]
B6		MCF-7 siCtrl (3)	17.2	1.65	2.13E-04		
B7		MCF-7 siCtrl (3)	17.1	1.62	2.23E-04		
B8		MCF-7 siAATK (3)	17.0	1.63	2.34E-04	17.0	[1.$,1.$]
C1		MCF-7 siAATK (3)	17.0	1.63	2.34E-04		
C2		MCF-7 siAATK (3)	17.0	1.70	2.34E-04		
E8		SkMel13 siCtrl (3)	18.6	1.61	1.07E-04	18.6	[1.$,1.$]
F1		SkMel13 siCtrl (3)	18.5	1.60	1.12E-04		
F2		SkMel13 siCtrl (3)	18.6	1.61	1.07E-04		
F3		SkMel13 siAATK (3)	18.9	1.63	9.21E-05	18.8	[1.$,1.$]
F4		SkMel13 siAATK (3)	19.0	1.59	8.77E-05		
F5		SkMel13 siAATK (3)	18.6	1.58	1.07E-04		
H2		H2O	19.1	0.00	8.35E-05	19.1	

Quality System ™
Quality Endorsed Company
ISO 9001 Lic 21313
SAI Global

This report generated by Rotor-Gene Real-Time Analysis Software 6.1 (Build 93)
© Corbett Research 2005
® All Rights Reserved
ISO 9001:2000 (Reg. No. QEC21313)
