# Supplementary material for: Epigenetically silenced apoptosis-associated tyrosine kinase (AATK) facilitates a decreased expression of Cyclin D1 and WEE1, phosphorylates TP53 and reduces cell proliferation in a kinase-dependent manner
Source: Cancer Gene Ther. 2022 Jul 28;29(12):1975–87. doi: 10.1038/s41417-022-00513-x (PMC9750878; doi:10.1038/s41417-022-00513-x)
Supplement: Supplementary file 6 — Dataset original qPCR [file 41417_2022_513_MOESM6_ESM.zip › RNAi_CCND1_2.pdf]

corbett
RESEARCH
A Division of Corbett Life Science

14 Hilly Street Mortlake NSW 2137 Australia
T +61 2 9736 1320
F +61 2 9736 1364
W www.corbettlifescience.com

Comparative Quantitation Report

Experiment Information

Run Name	Run 2020-06-03_CCND1_RNAi-MCF7,A427,SkMel13_(2)
Run Start	03.06.2020 12:40:41
Run Finish	03.06.2020 14:36:00
Operator	MW
Notes	CCND1 RNAi MCF-7,A427;SkMel13 (2) triplicate
Run On Software Version	Rotor-Gene 6.1.93
Run Signature	The Run Signature is valid.
Gain FAM	8.
Gain ROX	9.33

Comparative Quantitation Information

Reaction Amplification	1.70
Reaction Amplification Std. Deviation	0.03
Sample Page	Page 1
Control Replicate	(5)

Take off Graph for Cycling A.FAM/Cycling A.ROX

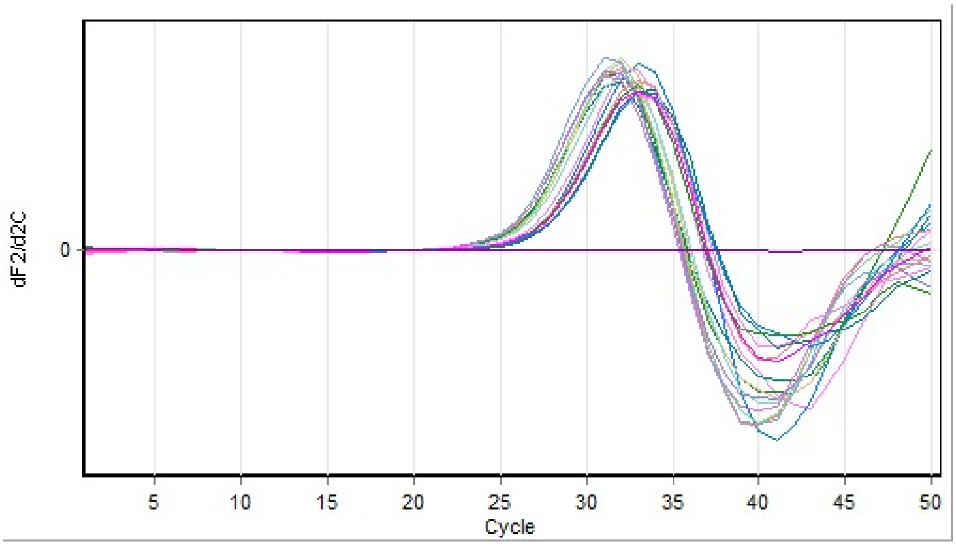

No.	Colour	Name	Take Off	Amplification	Comparative Conc.	Rep. Takeoff	Rep. Takeoff (95% CI)
A5		MCF-7 siCtrl (1)	28.3	1.69	1.05E+00	28.4	
A6		MCF-7 siCtrl (1)	28.5	1.77	9.48E-01		
A7		MCF-7 siAATK (1)	27.2	1.69	1.89E+00	27.2	[1.$,1.$]
A8		MCF-7 siAATK (1)	27.2	1.65	1.89E+00		
B1		MCF-7 siAATK (1)	27.3	1.67	1.79E+00		
B5		MCF-7 siCtrl (2)	27.7	1.68	1.45E+00	27.6	[1.$,1.$]
B6		MCF-7 siCtrl (2)	27.5	1.73	1.61E+00		
B7		MCF-7 siCtrl (2)	27.7	1.72	1.45E+00		
B8		MCF-7 siAATK (2)	27.1	1.74	1.99E+00	27.0	[1.$,1.$]
C1		MCF-7 siAATK (2)	27.0	1.67	2.10E+00		
C2		MCF-7 siAATK (2)	26.9	1.72	2.21E+00		
E5		SkMel13 siCtrl 24h (3)	28.6	1.72	9.00E-01	28.7	[1.$,1.$]
E6		SkMel13 siCtrl 24h (3)	28.8	1.66	8.09E-01		
E7		SkMel13 siCtrl 24h (3)	28.7	1.71	8.53E-01		
E8		SkMel13 siAATK(3)	28.5	1.64	9.48E-01	28.5	[1.$,1.$]
F1		SkMel13 siAATK(3)	28.5	1.67	9.48E-01		
F2		SkMel13 siAATK(3)	28.5	1.72	9.48E-01		
H2		H2O	45.0	0.00	1.53E-04	45.0	

Quality System ™ Quality Endorsed Company ISO 9001 Lic 21313 SAI Global

This report generated by Rotor-Gene Real-Time Analysis Software 6.1 (Build 93)
© Corbett Research 2005
® All Rights Reserved
ISO 9001:2000 (Reg. No. QEC21313)
