# Supplementary material for: Epigenetically silenced apoptosis-associated tyrosine kinase (AATK) facilitates a decreased expression of Cyclin D1 and WEE1, phosphorylates TP53 and reduces cell proliferation in a kinase-dependent manner
Source: Cancer Gene Ther. 2022 Jul 28;29(12):1975–87. doi: 10.1038/s41417-022-00513-x (PMC9750878; doi:10.1038/s41417-022-00513-x)
Supplement: Supplementary file 6 — Dataset original qPCR [file 41417_2022_513_MOESM6_ESM.zip › HCT116del_CCND1.pdf]

14 Hilly Street Mortlake NSW 2137 Australia
T +61 2 9736 1320
F +61 2 9736 1364
W www.corbettlifescience.com

Comparative Quantitation Report

Experiment Information

Run Name	Run 2019-07-05_CCND1_HCT-OE_starved
Run Start	05.07.2019 12:12:42
Run Finish	05.07.2019 14:09:22
Operator	MW
Notes	HCT OE starved Ccnd1 Triplicate
Run On Software Version	Rotor-Gene 6.1.93
Run Signature	The Run Signature is valid.
Gain FAM	8.
Gain ROX	9.33

Comparative Quantitation Information

Reaction Amplification	1.47
Reaction Amplification Std. Deviation	0.08
Sample Page	Page 1
Control Replicate	(19)

Take off Graph for Cycling A.FAM

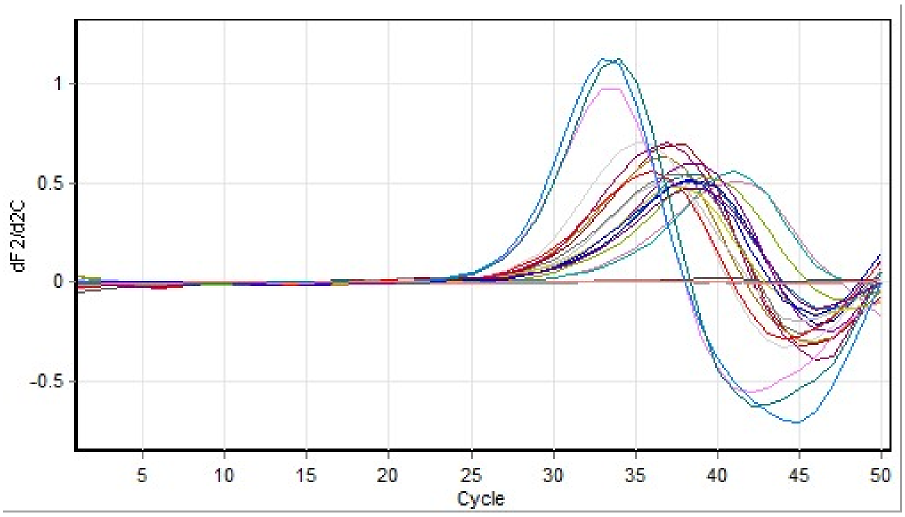

No.	Colour	Name	Take Off	Amplification	Comparative Conc.	Rep. Takeoff	Rep. Takeoff (95% CI)
C3		HCT116 del p53 w/o	34.1	1.41	4.03E-01	31.7	[1.$,1.$]
C4		HCT116 del p53 w/o	30.8	1.43	1.43E+00		
C5		HCT116 del p53 w/o	30.3	1.45	1.73E+00		
C6		HCT116 del p53 EYFP	33.0	1.47	6.15E-01	33.5	[1.$,1.$]
C7		HCT116 del p53 EYFP	35.0	1.41	2.85E-01		
C8		HCT116 del p53 EYFP	32.4	1.40	7.74E-01		
D1		HCT116 del p53 AATKA	31.9	1.41	9.38E-01	31.5	[1.$,1.$]
D2		HCT116 del p53 AATKA	32.1	1.39	8.69E-01		
D3		HCT116 del p53 AATKA	30.5	1.55	1.61E+00		
D4		HCT116 del p53 AATKA KD	29.5	1.56	2.36E+00	26.0	[1.$,1.$]
D5		HCT116 del p53 AATKA KD	31.0	1.46	1.33E+00		
D6		HCT116 del p53 AATKA KD	17.6	0.05	2.27E+02		
D7		HCT116 del p53 AATKB	30.9	1.40	1.38E+00	32.3	[1.$,1.$]
D8		HCT116 del p53 AATKB	36.6	1.43	1.54E-01		
E1		HCT116 del p53 AATKB	29.5	1.46	2.36E+00		
E2		HCT116 del p53 AATKB KD	31.2	1.45	1.23E+00	31.6	[1.$,1.$]
E3		HCT116 del p53 AATKB KD	31.5	1.45	1.09E+00		
E4		HCT116 del p53 AATKB KD	32.1	1.40	8.69E-01		

(Continued on next page)...

No.	Colour	Name	Take Off	Amplification	Comparative Conc.	Rep. Takeoff	Rep. Takeoff (95% CI)
E5		Mix	28.5	1.62	3.46E+00	28.6	[1.$,1.$]
E6		Mix	28.5	1.61	3.46E+00		
E7		Mix	28.9	1.60	2.97E+00		
E8		H2O	27.2	0.31	5.70E+00	27.2	

Quality System
Quality Endorsed Company
ISO 9001 Lic 21313
SAI Global

™ This report generated by Rotor-Gene Real-Time Analysis Software 6.1 (Build 93)
© Corbett Research 2005
® All Rights Reserved
ISO 9001:2000 (Reg. No. QEC21313)
