# Supplementary material for: Epigenetically silenced apoptosis-associated tyrosine kinase (AATK) facilitates a decreased expression of Cyclin D1 and WEE1, phosphorylates TP53 and reduces cell proliferation in a kinase-dependent manner
Source: Cancer Gene Ther. 2022 Jul 28;29(12):1975–87. doi: 10.1038/s41417-022-00513-x (PMC9750878; doi:10.1038/s41417-022-00513-x)
Supplement: Supplementary file 6 — Dataset original qPCR [file 41417_2022_513_MOESM6_ESM.zip › RNAi_ACTB_1.pdf]

corbett RESEARCH
A Division of Corbett Life Science

14 Hilly Street Mortlake NSW 2137 Australia
T +61 2 9736 1320
F +61 2 9736 1364
W www.corbettlifescience.com

Comparative Quantitation Report

Experiment Information

Run Name	Run 2020-04-19_b-Act_RNAi_div-CL
Run Start	19.04.2020 11:39:29
Run Finish	19.04.2020 13:16:53
Operator	MW
Notes	b-Act RNAi triplicate
Run On Software Version	Rotor-Gene 6.1.93
Run Signature	The Run Signature is valid.
Gain FAM	8.
Gain ROX	8.

Comparative Quantitation Information

Reaction Amplification	1.64
Reaction Amplification Std. Deviation	0.02
Sample Page	Page 1
Control Replicate	(7)

Take off Graph for Cycling A.FAM/Cycling A.ROX

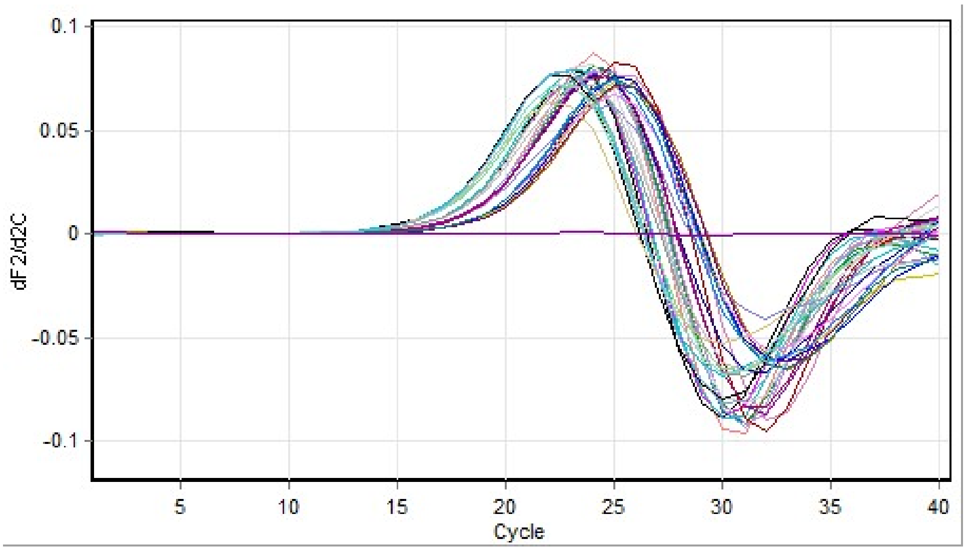

No.	Colour	Name	Take Off	Amplification	Comparative Conc.	Rep. Takeoff	Rep. Takeoff (95% CI)
A7		HEK siCtrl (1)	19.7	1.64	1.03E+00	19.8	[1.$,1.$]
A8		HEK siCtrl (1)	19.8	1.67	9.84E-01		
B1		HEK siCtrl (1)	19.8	1.66	9.84E-01		
B2		HEK siAATK (1)	18.9	1.67	1.54E+00	19.0	[1.$,1.$]
B3		HEK siAATK (1)	19.1	1.67	1.39E+00		
B4		HEK siAATK (1)	18.9	1.67	1.54E+00		
B8		HEK siCtrl (2)	19.7	1.66	1.03E+00	19.6	[1.$,1.$]
C1		HEK siCtrl (2)	19.4	1.62	1.20E+00		
C2		HEK siCtrl (2)	19.8	1.67	9.84E-01		
C3		HEK siAATK (2)	21.0	1.68	5.43E-01	20.9	[1.$,1.$]
C4		HEK siAATK (2)	21.0	1.65	5.43E-01		
C5		HEK siAATK (2)	20.8	1.63	5.99E-01		
D1		SkMel13 siCtrl (1)	19.8	1.60	9.84E-01	19.7	[1.$,1.$]
D2		SkMel13 siCtrl (1)	19.7	1.62	1.03E+00		
D3		SkMel13 siCtrl (1)	19.7	1.62	1.03E+00		
D4		SkMel13 siAATK (1)	19.2	1.67	1.32E+00	19.0	[1.$,1.$]
D5		SkMel13 siAATK (1)	18.9	1.65	1.54E+00		
D6		SkMel13 siAATK (1)	19.0	1.67	1.46E+00		

(Continued on next page)...

No.	Colour	Name	Take Off	Amplification	Comparative Conc.	Rep. Takeoff	Rep. Takeoff (95% CI)
E2		SkMel13 siCtrl (2)	20.7	1.63	6.30E-01	20.7	[1.$,1.$]
E3		SkMel13 siCtrl (2)	20.8	1.64	5.99E-01		
E4		SkMel13 siCtrl (2)	20.7	1.64	6.30E-01		
E5		SkMel13 siAATK (2)	20.2	1.64	8.07E-01	20.3	[1.$,1.$]
E6		SkMel13 siAATK (2)	20.4	1.62	7.31E-01		
E7		SkMel13 siAATK (2)	20.4	1.63	7.31E-01		

F3		MCF7 siCtrl (1)	18.0	1.63	2.40E+00	17.9	[1.$,1.$]
F4		MCF7 siCtrl (1)	18.1	1.63	2.28E+00		
F5		MCF7 siCtrl (1)	17.7	1.61	2.79E+00		
F6		MCF7 siAATK (1)	18.2	1.60	2.17E+00	18.2	[1.$,1.$]
F7		MCF7 siAATK (1)	18.2	1.63	2.17E+00		
F8		MCF7 siAATK (1)	18.2	1.64	2.17E+00		
H2		H2O	20.9	0.00	5.70E-01	20.9	

Quality System ™
Quality Endorsed Company
ISO 9001 Lic 21313
SAI Global
This report generated by Rotor-Gene Real-Time Analysis Software 6.1 (Build 93)
© Corbett Research 2005
® All Rights Reserved
ISO 9001:2000 (Reg. No. QEC21313)
