# Supplementary material for: Epigenetically silenced apoptosis-associated tyrosine kinase (AATK) facilitates a decreased expression of Cyclin D1 and WEE1, phosphorylates TP53 and reduces cell proliferation in a kinase-dependent manner
Source: Cancer Gene Ther. 2022 Jul 28;29(12):1975–87. doi: 10.1038/s41417-022-00513-x (PMC9750878; doi:10.1038/s41417-022-00513-x)
Supplement: Supplementary file 6 — Dataset original qPCR [file 41417_2022_513_MOESM6_ESM.zip › RNAi_CCND1_1.pdf]

corbett RESEARCH
A Division of Corbett Life Science

14 Hilly Street Mortlake NSW 2137 Australia
T +61 2 9736 1320
F +61 2 9736 1364
W www.corbettlifescience.com

Comparative Quantitation Report

Experiment Information

Run Name	Run 2020-03-29_CCND1_RNAi_div-CL
Run Start	29.03.2020 14:55:50
Run Finish	29.03.2020 16:48:17
Operator	MW
Notes	CCND1 RNAi div. CL triplicate
Run On Software Version	Rotor-Gene 6.1.93
Run Signature	The Run Signature is valid.
Gain FAM	8.
Gain ROX	9.33

Comparative Quantitation Information

Reaction Amplification	1.53
Reaction Amplification Std. Deviation	0.11
Sample Page	Page 1
Control Replicate	(7)

Take off Graph for Cycling A.FAM/Cycling A.ROX

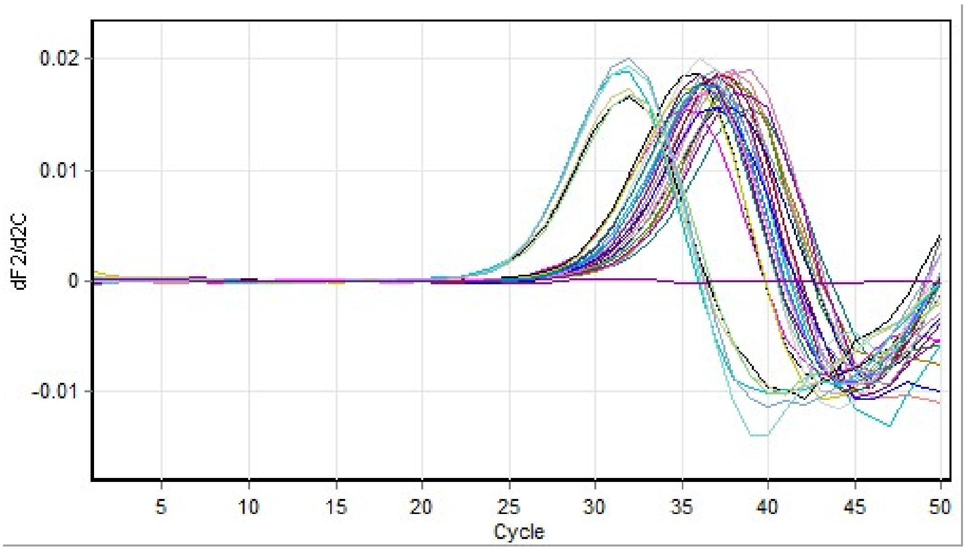

No.	Colour	Name	Take Off	Amplification	Comparative Conc.	Rep. Takeoff	Rep. Takeoff (95% CI)
A7		HEK siCtrl (1)	33.5	1.55	8.56E-01	33.1	[1.$,1.$]
A8		HEK siCtrl (1)	32.8	1.52	1.15E+00		
B1		HEK siCtrl (1)	33.1	1.56	1.01E+00		
B2		HEK siAATK (1)	30.1	1.60	3.64E+00	30.6	[1.$,1.$]
B3		HEK siAATK (1)	30.3	1.30	3.34E+00		
B4		HEK siAATK (1)	31.3	1.60	2.18E+00		
B8		HEK siCtrl (2)	31.9	1.52	1.69E+00	32.2	[1.$,1.$]
C1		HEK siCtrl (2)	32.0	1.57	1.62E+00		
C2		HEK siCtrl (2)	32.6	1.44	1.25E+00		
C3		HEK siAATK (2)	33.5	1.62	8.56E-01	33.0	[1.$,1.$]
C4		HEK siAATK (2)	32.3	1.64	1.43E+00		
C5		HEK siAATK (2)	33.2	1.62	9.72E-01		
D1		SkMel13 siCtrl (1)	32.5	1.36	1.31E+00	32.5	[1.$,1.$]
D2		SkMel13 siCtrl (1)	33.3	1.56	9.32E-01		
D3		SkMel13 siCtrl (1)	31.8	1.55	1.76E+00		
D4		SkMel13 siAATK (1)	31.2	1.63	2.28E+00	31.6	[1.$,1.$]
D5		SkMel13 siAATK (1)	31.0	1.59	2.48E+00		
D6		SkMel13 siAATK (1)	32.5	1.41	1.31E+00		

(Continued on next page)...

No.	Colour	Name	Take Off	Amplification	Comparative Conc.	Rep. Takeoff	Rep. Takeoff (95% CI)
D7		SkMel13 ohne 48h	31.5	1.39	2.00E+00	31.5	
E2		SkMel13 siCtrl (2)	30.4	1.33	3.20E+00	31.1	[1.$,1.$]
E3		SkMel13 siCtrl (2)	31.9	1.56	1.69E+00		
E4		SkMel13 siCtrl (2)	31.1	1.66	2.38E+00		
E5		SkMel13 siAATK (2)	31.4	1.59	2.09E+00	31.2	[1.$,1.$]
E6		SkMel13 siAATK (2)	31.5	1.59	2.00E+00		

E7		SkMel13 siAATK (2)	30.8	1.50	2.70E+00		
F3		MCF7 siCtrl (1)	26.9	1.57	1.42E+01	26.9	[1.$,1.$]
F4		MCF7 siCtrl (1)	26.8	1.60	1.48E+01		
F5		MCF7 siCtrl (1)	26.9	1.56	1.42E+01		
F6		MCF7 siAATK (1)	27.2	1.25	1.25E+01	26.9	[1.$,1.$]
F7		MCF7 siAATK (1)	26.8	1.61	1.48E+01		
F8		MCF7 siAATK (1)	26.8	1.58	1.48E+01		
H2		H2O	29.0	0.00	5.81E+00	29.0	

Quality System
Quality Endorsed Company
ISO 9001 Lic 21313
SAI Global

This report generated by Rotor-Gene Real-Time Analysis Software 6.1 (Build 93)
© Corbett Research 2005
® All Rights Reserved
ISO 9001:2000 (Reg. No. QEC21313)
