# Supplementary material for: Epigenetically silenced apoptosis-associated tyrosine kinase (AATK) facilitates a decreased expression of Cyclin D1 and WEE1, phosphorylates TP53 and reduces cell proliferation in a kinase-dependent manner
Source: Cancer Gene Ther. 2022 Jul 28;29(12):1975–87. doi: 10.1038/s41417-022-00513-x (PMC9750878; doi:10.1038/s41417-022-00513-x)
Supplement: Supplementary file 6 — Dataset original qPCR [file 41417_2022_513_MOESM6_ESM.zip › GAPDH_PaTu-S.pdf]

corbett RESEARCH
A Division of Corbett Life Science

14 Hilly Street Mortlake NSW 2137 Australia
T +61 2 9736 1320
F +61 2 9736 1364
W www.corbettlifescience.com

Comparative Quantitation Report

Experiment Information

Run Name	Run 2017-01-19_GAPDH_Pankreas_Aza_all
Run Start	19.01.2017 10:41:15
Run Finish	19.01.2017 11:53:35
Operator	MW
Notes	GAPDH Aza Pankeas all triplicate
Run On Software Version	Rotor-Gene 6.1.93
Run Signature	The Run Signature is valid.
Gain FAM	8.
Gain ROX	8.

Comparative Quantitation Information

Reaction Amplification	1.71
Reaction Amplification Std. Deviation	0.03
Sample Page	Page 1
Control Replicate	(31)

Take off Graph for Cycling A.FAM/Cycling A.ROX

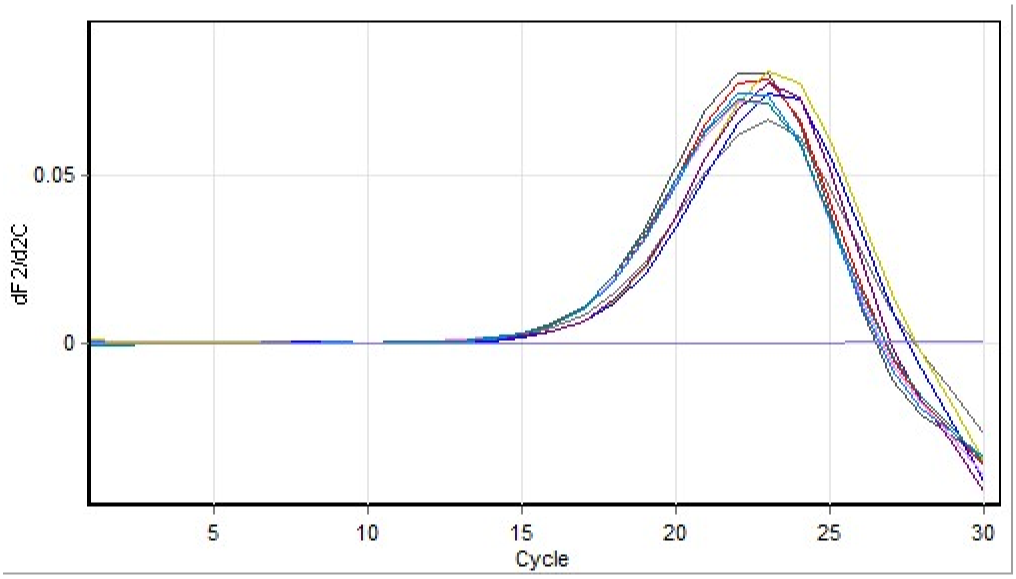

No.	Colour	Name	Take Off	Amplification	Comparative Conc.	Rep. Takeoff	Rep. Takeoff (95% CI)
D7		PaTu-S 0 uM	18.4	1.67	9.48E-01	18.3	[1.$,1.$]
D8		PaTu-S 0 uM	18.2	1.71	1.06E+00		
E1		PaTu-S 0 uM	18.3	1.71	1.00E+00		
E2		PaTu-S 5 uM	19.0	1.76	6.87E-01	19.0	[1.$,1.$]
E3		PaTu-S 5 uM	19.0	1.66	6.87E-01		
E4		PaTu-S 5 uM	18.9	1.73	7.25E-01		
E5		PaTu-S 10 uM	18.2	1.75	1.06E+00	18.2	[1.$,1.$]
E6		PaTu-S 10 uM	18.3	1.69	1.00E+00		
E7		PaTu-S 10 uM	18.1	1.70	1.11E+00		
G1		H2O	25.1	0.00	2.62E-02	25.1	

Quality System™
Quality Endorsed Company
ISO 9001 Lic 21313
SAI Global

This report generated by Rotor-Gene Real-Time Analysis Software 6.1 (Build 93)
© Corbett Research 2005
® All Rights Reserved
ISO 9001:2000 (Reg. No. QEC21313)
