# Supplementary material for: Epigenetically silenced apoptosis-associated tyrosine kinase (AATK) facilitates a decreased expression of Cyclin D1 and WEE1, phosphorylates TP53 and reduces cell proliferation in a kinase-dependent manner
Source: Cancer Gene Ther. 2022 Jul 28;29(12):1975–87. doi: 10.1038/s41417-022-00513-x (PMC9750878; doi:10.1038/s41417-022-00513-x)
Supplement: Supplementary file 6 — Dataset original qPCR [file 41417_2022_513_MOESM6_ESM.zip › Glioblastoma_GAPDH.pdf]

corbett RESEARCH
A Division of Corbett Life Science

14 Hilly Street Mortlake NSW 2137 Australia
T +61 2 9736 1320
F +61 2 9736 1364
W www.corbettlifescience.com

Comparative Quantitation Report

Experiment Information

Run Name	Run 2019-01-03_GAPDH_Glio-CL
Run Start	03.01.2019 09:32:57
Run Finish	03.01.2019 11:23:51
Operator	MW
Notes	GAPDH Glio CL triplicate
Run On Software Version	Rotor-Gene 6.1.93
Run Signature	The Run Signature is valid.
Gain FAM	8.
Gain ROX	8.

Comparative Quantitation Information

Reaction Amplification	1.66
Reaction Amplification Std. Deviation	0.03
Sample Page	Page 1
Control Replicate	(26)

Take off Graph for Cycling A.FAM

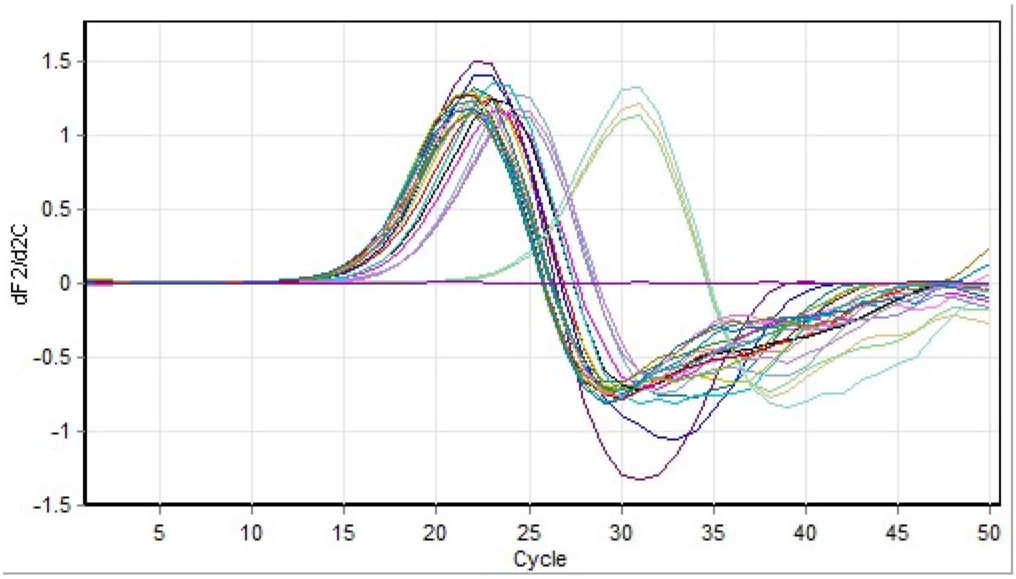

No.	Colour	Name	Take Off	Amplification	Comparative Conc.	Rep. Takeoff	Rep. Takeoff (95% CI)
A1		LN229	17.9	1.66	3.05E+00	17.9	[1.$,1.$]
A2		LN229	17.9	1.65	3.05E+00		
A3		LN229	18.0	1.67	2.90E+00		
A4		U343	17.5	1.67	3.74E+00	17.3	[1.$,1.$]
A5		U343	17.3	1.64	4.13E+00		
A6		U343	17.2	1.72	4.35E+00		
A7		U118	17.5	1.69	3.74E+00	17.3	[1.$,1.$]
A8		U118	17.3	1.63	4.13E+00		
B1		U118	17.2	1.68	4.35E+00		
B2		U87MG	18.8	1.65	1.93E+00	18.7	[1.$,1.$]
B3		U87MG	18.6	1.65	2.14E+00		
B4		U87MG	18.6	1.64	2.14E+00		
B5		T98G	26.1	1.63	4.78E-02	26.0	[1.$,1.$]
B6		T98G	25.9	1.64	5.29E-02		
B7		T98G	26.1	1.64	4.78E-02		
B8		A172	19.7	1.65	1.22E+00	19.6	[1.$,1.$]
C1		A172	19.6	1.61	1.29E+00		
C2		A172	19.6	1.63	1.29E+00		

(Continued on next page)...

No.	Colour	Name	Take Off	Amplification	Comparative Conc.	Rep. Takeoff	Rep. Takeoff (95% CI)
C3		A764	16.9	1.68	5.06E+00	17.0	[1.$,1.$]
C4		A764	17.0	1.69	4.81E+00		
C5		A764	17.0	1.65	4.81E+00		
C6		SNB19	17.0	1.69	4.81E+00	16.9	[1.$,1.$]

C7		SNB19	16.9	1.69	5.06E+00		
C8		SNB19	16.8	1.66	5.33E+00		
D2		H_2O	20.1	0.23	1.00E+00	20.1	

Quality System
Quality Endorsed Company
ISO 9001 Lic 21313
SAI Global

™ This report generated by Rotor-Gene Real-Time Analysis Software 6.1 (Build 93)
© Corbett Research 2005
® All Rights Reserved
ISO 9001:2000 (Reg. No. QEC21313)
