# Supplementary material for: Epigenetically silenced apoptosis-associated tyrosine kinase (AATK) facilitates a decreased expression of Cyclin D1 and WEE1, phosphorylates TP53 and reduces cell proliferation in a kinase-dependent manner
Source: Cancer Gene Ther. 2022 Jul 28;29(12):1975–87. doi: 10.1038/s41417-022-00513-x (PMC9750878; doi:10.1038/s41417-022-00513-x)
Supplement: Supplementary file 6 — Dataset original qPCR [file 41417_2022_513_MOESM6_ESM.zip › NOVA1_clone pools.pdf]

corbett RESEARCH
A Division of Corbett Life Science

14 Hilly Street Mortlake NSW 2137 Australia
T + 61 2 9736 1320
F + 61 2 9736 1364
W www.corbettlifescience.com

Comparative Quantitation Report

Experiment Information

Run Name	Run 2019-06-18_Affy_Nova1_CCND1
Run Start	18.06.2019 09:54:43
Run Finish	18.06.2019 11:50:05
Operator	MW
Notes	Affy cDNA Nova1 Ccnd1 Triplicate
Run On Software Version	Rotor-Gene 6.1.93
Run Signature	The Run Signature is valid.
Gain FAM	8.
Gain ROX	9.33

Comparative Quantitation Information

Reaction Amplification	1.69
Reaction Amplification Std. Deviation	0.02
Sample Page	Page 1
Control Replicate	(1)

Take off Graph for Cycling A.FAM

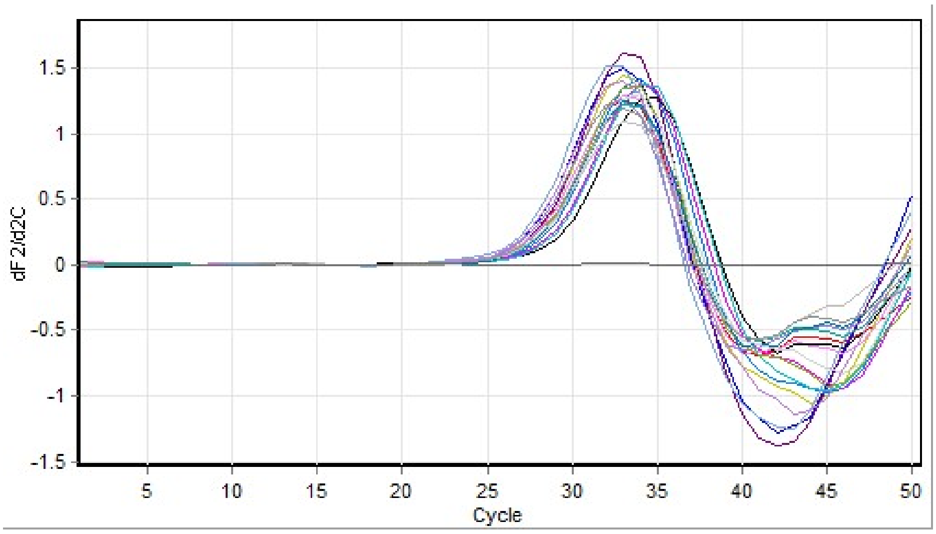

No.	Colour	Name	Take Off	Amplification	Comparative Conc.	Rep. Takeoff	Rep. Takeoff (95% CI)
A1		Control clone pool (1)	28.5	1.69	1.00E+00	28.5	[1.$,1.$]
A2		Control clone pool (1)	28.6	1.70	9.49E-01		
A3		Control clone pool (1)	28.4	1.68	1.05E+00		
A4		Control clone pool (2)	28.9	1.71	8.10E-01	28.9	[1.$,1.$]
A5		Control clone pool (2)	28.5	1.68	1.00E+00		
A6		Control clone pool (2)	29.3	1.72	6.57E-01		
B2		Clone pool AATK (1)	29.7	1.68	5.32E-01	29.9	[1.$,1.$]
B3		Clone pool AATK (1)	30.1	1.70	4.31E-01		
B4		Clone pool AATK (1)	29.9	1.70	4.79E-01		
B8		Clone pool AATK KD (1)	28.1	1.73	1.23E+00	28.2	[1.$,1.$]
C1		Clone pool AATK KD (1)	28.2	1.66	1.17E+00		
C2		Clone pool AATK KD (1)	28.2	1.67	1.17E+00		
C6		Clone pool AATK (2)	28.9	1.68	8.10E-01	28.9	[1.$,1.$]
C7		Clone pool AATK (2)	28.8	1.70	8.54E-01		
C8		Clone pool AATK (2)	28.9	1.70	8.10E-01		
D4		Clone pool AATK KD (2)	28.9	1.67	8.10E-01	28.7	[1.$,1.$]
D5		Clone pool AATK KD (2)	28.6	1.69	9.49E-01		
D6		Clone pool AATK KD (2)	28.5	1.68	1.00E+00		
D7		H2O NOVA1	31.1	1.56	2.55E-01	31.1	

Quality System
Quality Endorsed Company
ISO 9001 Lic 21313
SAI Global

This report generated by Rotor-Gene Real-Time Analysis Software 6.1 (Build 93)
© Corbett Research 2005
® All Rights Reserved
ISO 9001:2000 (Reg. No. QEC21313)
